# Design, Synthesis, and Biological Evaluation of Novel ML-167 Analogues as Therapeutic Candidates Against Sarcopenia-Induced Muscle Wasting

**DOI:** 10.3390/ph19091442

**Published:** 2026-09-11

**Authors:** Issam Ameziane El Hassani, Shabnam Alizadeh, Sajda Ashraf, N. Ceren Suer, Aybuke Ozturk, Woonghee Kim, Muhammed Melik Saracoglu, Edanur Yildiz, Seymanur Baycelebi, Gonca Candan, Gizem Bati Ayaz, Mustafa Kara, Murat Ozdemir, Busra Turan, Neaz Ahmed, Onur Ceylan, Sevilay Ozmen, Fatih Isik, Eda Sahin, Fatih Alper, Hasan Turkez, Adil Mardinoglu

**Affiliations:** 1 Trustlife Labs, Drug Research & Development Center, Istanbul 34774, Türkiye; i.amezianeelhassani@gmail.com (I.A.E.H.); shabnam.alizadeh@trustlifelabs.com (S.A.); ceren.suer@trustlifelabs.com (N.C.S.); murat.ozdemir@trustlifelabs.com (M.O.);; 2 Science for Life Laboratory, KTH-Royal Institute of Technology, SE-17165 Stockholm, Sweden; 3 Institute of Science, Atatürk University, Erzurum 25240, Türkiye; 4 Institute of Science, Erzurum Technical University, Erzurum 25240, Türkiye; 5 Department of Pathology, Faculty of Medicine, Atatürk University, Erzurum 25240, Türkiye; 6 Department of Radiology, Faculty of Medicine, Atatürk University, Erzurum 25240, Türkiye; 7 Department of Medical Biology, Faculty of Medicine, Atatürk University, Erzurum 25240, Türkiye; hasanturkez@gmail.com; 8 Centre for Host-Microbiome Interactions, Faculty of Dentistry, Oral & Craniofacial Sciences, King’s College London, London SE1 9RT, UK

**Keywords:** sarcopenia, muscle atrophy, myogenic differentiation, myogenin (MyoG), myosin heavy chain 3 (MyH3), preclinical efficacy, in vivo rat model

## Abstract

**Background**: Sarcopenia is an aging-related syndrome characterized by loss of skeletal muscle mass, strength, and function, with no approved pharmacological treatment. This study aimed to design, synthesize, and evaluate novel ML-167 derivatives for sarcopenia-associated muscle wasting. **Methods**: ML-167 derivatives were synthesized and characterized by ^1^H NMR, ^13^C NMR, and mass spectrometry. Biological activity was evaluated in C2C12 myoblasts using MTT and Western blot assays. Selected compounds were evaluated in a dexamethasone-induced muscle wasting model. Muscle strength, mass, biochemical parameters, and histopathology were assessed. Structure–activity relationships were analysed using cell viability, molecular docking, and ligand efficiency data, alongside physicochemical properties. **Results**: Compounds **6b** (116%), **3a** (115%), **8d** (114%), **4c** (112%), **3m** (112%), and **3c** (111%) showed the highest C2C12 viability at 1 μM. Compounds **3a**, **3c**, and **6b** increased MyH3 expression by approximately 1.6–1.7-fold, while MyoG remained near basal levels. In vivo, these compounds improved muscle strength, mass, and biochemical parameters without overt hepatic or renal abnormalities based on assessed serum and histopathological findings. Among the evaluated derivatives, compound **6b** emerged as the most promising overall lead candidate, showing the greatest improvement in skeletal muscle mass and metabolic parameters, whereas compound **3c** demonstrated the strongest histopathological protection and favourable effects on muscle strength with favourable physicochemical properties. Molecular docking supported the SAR trends, although did not consistently correlate with cellular activity. **Conclusions**: The findings identify **3a**, **3c**, and **6b** as promising lead compounds and support further investigation of ML-167 derivatives as potential pharmacological approaches for sarcopenia-associated muscle wasting.

## 1. Introduction

Sarcopenia is an age-related disease characterized by the progressive loss of skeletal muscle mass and function, leading to increased weakness, disability, hospitalization and mortality rates [1,2]. Despite the increasing clinical burden, current treatment relies primarily on exercise, nutritional support, hormone therapy, and emerging biotherapy methods while there is no approved pharmacological treatment specifically indicated for sarcopenia [3,4]. Sarcopenia is a multifactorial disorder involving chronic inflammation, oxidative stress, mitochondrial dysfunction, and impaired muscle proteostasis [5,6]. Age-related mitochondrial dysfunction disrupts redox homeostasis, leading to increased reactive oxygen species (ROS), lipid peroxidation, DNA damage, and muscle wasting [7]. Increased inflammatory cytokines (TNF-α, IL-1, IL-6, and IL-8) activate NF-κB-mediated catabolic pathways, leading to protein breakdown and impaired muscle regeneration [8]. Therefore, targeting oxidative stress and inflammation is a promising treatment strategy for sarcopenia.

Myoblasts differentiate into myotubes that mature into striated muscle fibers [9,10]. Myogenin (MyoG) is a key myogenic regulatory factor involved in terminal muscle differentiation, whereas embryonic myosin heavy chain (MyH3) is an early marker of myogenic differentiation and muscle maturation [11,12]. Our previous systems biology study identified key hub genes and pathways involved in sarcopenia. Accordingly, C2C12 myoblasts were used as an in vitro model of myogenesis. MyoG and MyH3 were selected as myogenic markers because of their roles in muscle differentiation and sarcopenia, and their modulation by the synthesized compounds supported their utility as indicators of myogenic status [13].

**Figure 1:**
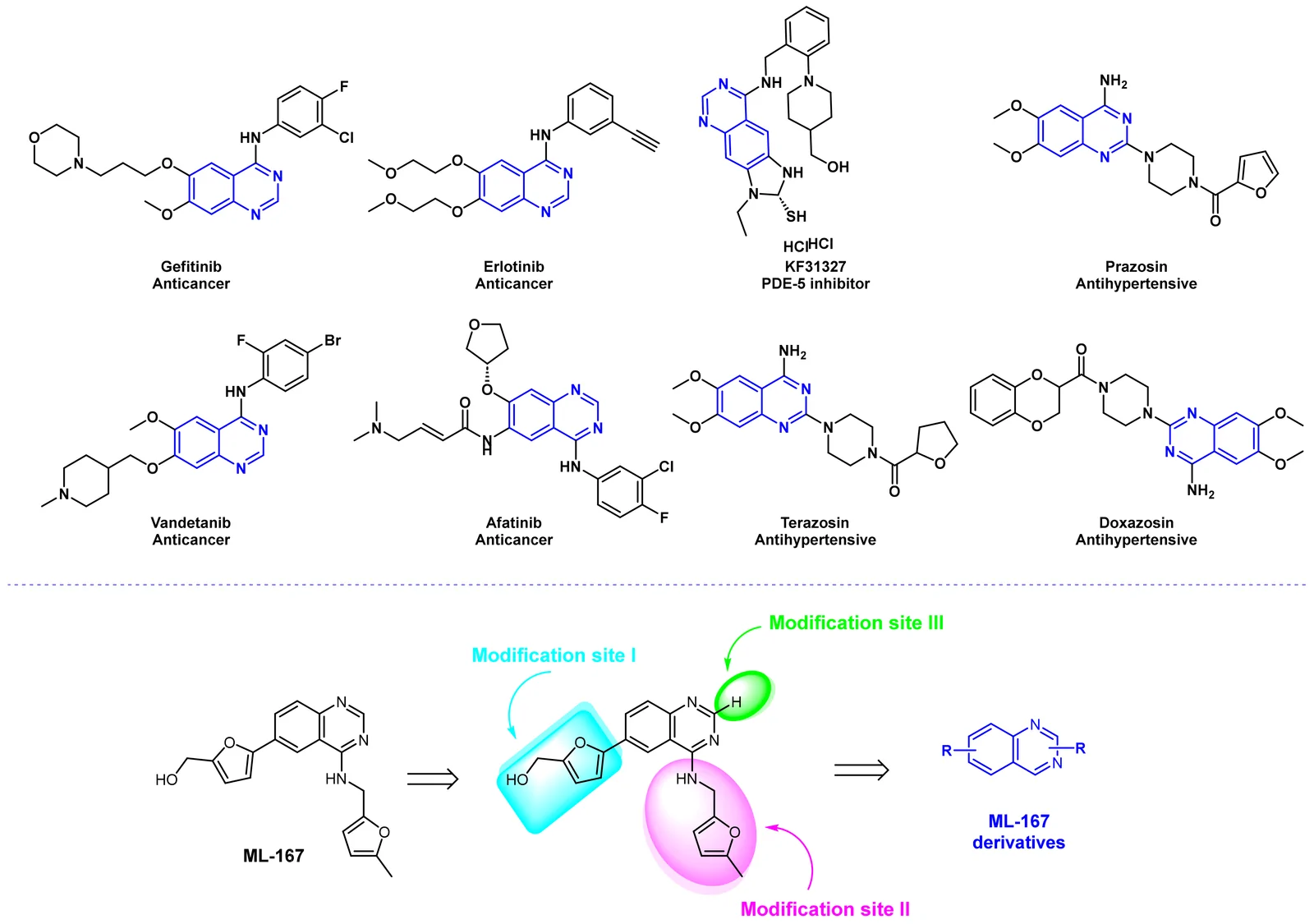
Some quinazoline clinical drugs and rational design of ML-167.

Quinazoline scaffolds, a prominent class of nitrogen-containing heterocycles, have attracted significant attention in synthetic and medicinal chemistry due to their presence in biologically active compounds and pharmaceutical agents [14,15,16]. Quinazoline derivatives exhibited a diverse range of pharmacological activities, including antibacterial, anticancer, antidiabetic, anticonvulsant, anti-inflammatory, and antihypertensive effects [16,17,18,19,20]. Notably, several clinically approved drugs, such as gefitinib, erlotinib, vandetanib, afatinib, terazosin, prazosin, and doxazosin, contain the quinazoline core and are used in the treatment of diverse diseases [21] (Figure 1).

ML-167, a quinazoline-based small molecule, has previously been investigated by our group through comprehensive structural and computational studies, including spectroscopic characterization, single-crystal X-ray diffraction, density functional theory (DFT) calculations, and molecular docking analysis. These studies provided detailed structural insights into the ML-167 scaffold [22]. In the present study, we explored ML-167 as a scaffold for drug repurposing and designed and synthesized a library of structurally modified derivatives to investigate their biological activity and structure–activity relationships (Figure 1). The compounds were characterized by ^1^H NMR, ^13^C NMR, and mass spectrometry, and evaluated in C2C12 myoblasts using the MTT assay and by measuring MyoG and MyH3 expression. Molecular docking was performed to rationalize structure–activity relationships and compound–target interactions. Finally, the most promising compounds were evaluated in a dexamethasone-induced sarcopenia-like muscle wasting model to assess their in vivo therapeutic potential.

## 2. Results and Discussion

### 2.1. Chemistry

**Scheme 1:**
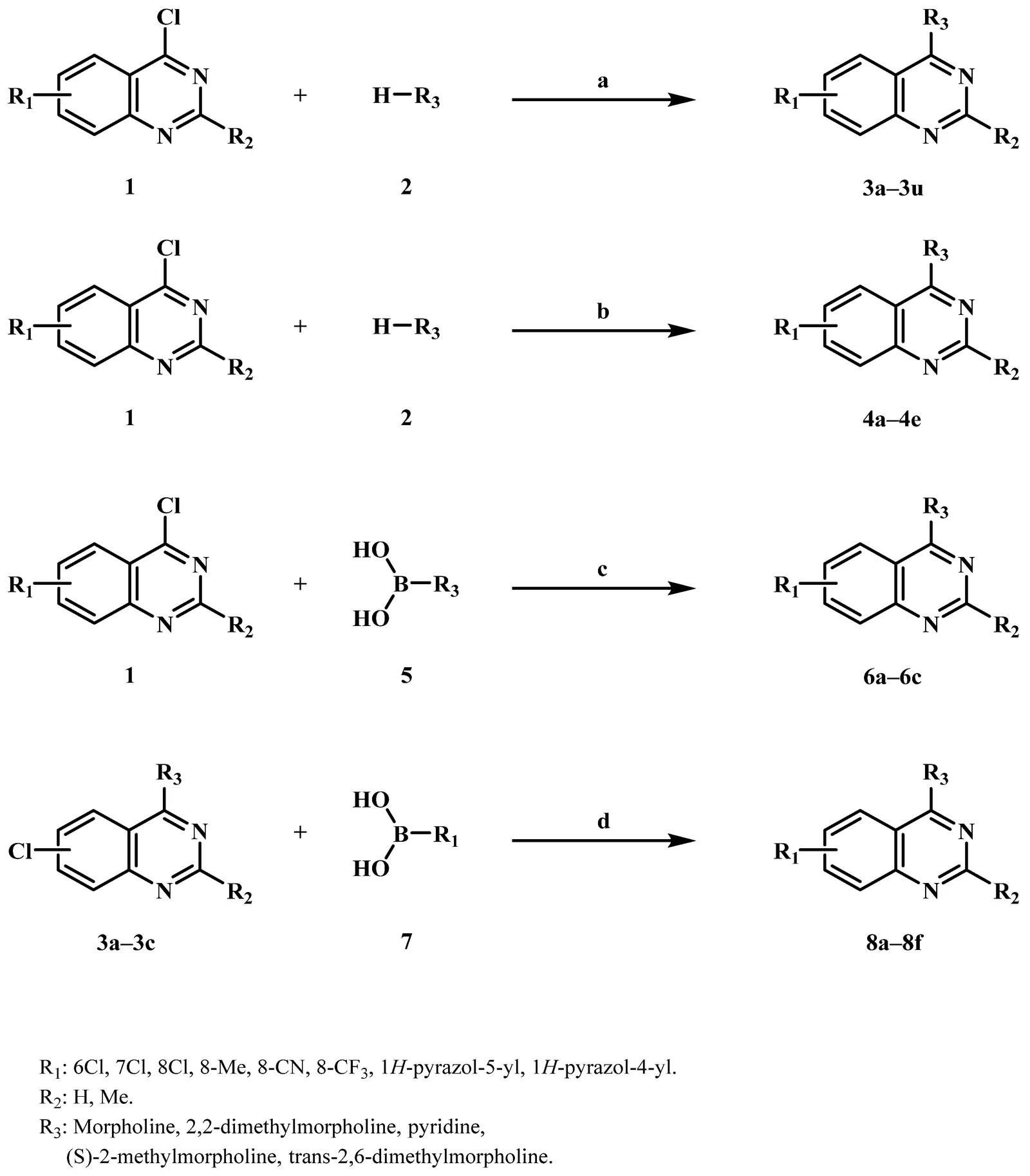
Synthesis of ML-167 derivatives (**3a**–**3u**), (**4a**–**4e**), (**6a**–**6c**) and (**8a**–**8f**): Reagents and conditions: (**a**) DCM, 0 °C, 2 h; (**b**) Cs_2_CO_3_, MeCN, 85 °C, reflux, 12 h; (**c**) Cs_2_CO_3_, Pd(dppf)Cl_2_, THF, 160 °C, MW, 0.5 h; (**d**) Cs_2_CO_3_, Pd(dppf)Cl_2_, DMF/Water, 160 °C, MW, 1.5 h.

The ML-167 derivatives (**3a**–**3u**), (**4a**–**4e**), (**6a**–**6c**), and (**8a**–**8f**), summarized in Table 1, were synthesized through a series of selective reactions, as outlined in Scheme 1. In the first approach, commercially available quinazoline derivative **1** underwent nucleophilic substitution with a series of morpholine derivatives **2** in dichloromethane at 0 °C, affording compounds (**3a**–**3u**). In the second approach, caesium carbonate was employed as the base in refluxing acetonitrile to yield compounds (**4a**–**4e**). Compounds (**6a**–**6c**) were synthesized by reacting quinazoline **1** with various boronic acid derivatives **5** under standard Suzuki–Miyaura coupling conditions, while compounds (**8a**–**8f**) were obtained under the same conditions using boronic acid derivatives **7** and intermediates (**3a**–**3g**). All compounds were isolated in good to excellent yields. The structures of the newly synthesized compounds (**3a**–**3u**), (**4a**–**4e**), (**6a**–**6c**), and (**8a**–**8f**), were confirmed through spectroscopic techniques, including ^1^H NMR (Figures S1a–S35a), 13C NMR (Figures S1b–S35b), and ESI-MS (Figures S1c–S35c).

In particular, the ^1^H NMR spectrum of compound **3d** displayed a singlet at 8.74 ppm, attributable to the proton at the 2-position of the quinazoline moiety. The methyl protons at the 8-position of the quinazoline ring appeared as a singlet at 2.66 ppm. The methylene (–CH_2_–) protons at the 2,6-positions of the morpholine ring were observed as a triplet at 3.83 ppm, while those at the 3,5-positions appeared as a triplet at 3.71 ppm. The aromatic protons resonated in the range of 7.46–7.90 ppm. The ^13^C NMR spectrum of compound **3d** exhibited a characteristic signal for the carbon at the 4-position of the quinazoline moiety at 164.86 ppm. The methyl carbon at the 8-position of the quinazoline ring resonated at 17.97 ppm. The methylene (–CH_2_–) carbons at the 2,6-positions of the morpholine ring were observed at 66.48 ppm, whereas those at the 3,5-positions appeared at 50.45 ppm. The carbon at the 2-position of the quinazoline moiety was detected at 153.31 ppm, and the aromatic carbons resonated within the range of 116.09–150.66 ppm. The ESI-MS spectrum of compound **3d** showed a molecular ion peak at *m*/*z* 230.09, corresponding to [*M*+*H*]^+^ for C_13_H_15_N_3_O (calculated *m*/*z* 230.12). As another example, the ^1^H NMR spectrum of compound **4d** showed a singlet at 8.69 ppm, assigned to the proton at the 2-position of the quinazoline moiety. The methyl protons at the 8-position of the quinazoline ring resonated as a singlet at 2.62 ppm. The methylene (–CH_2_–) protons at the 2-position of the morpholine ring appeared as a triplet at 3.85 ppm, whereas those at the 3-position appeared as a triplet at 3.63 ppm, and those at the 5-position as a singlet at 3.53 ppm. The two methyl groups at the 6-position of the morpholine moiety gave rise to a singlet at 1.23 ppm. The aromatic protons resonated within the range of 7.42–7.85 ppm. The ^13^C NMR spectrum of compound **4d** displayed a characteristic signal for the carbon at the 4-position of the quinazoline moiety at 165.11 ppm, while the carbon at the 2-position was observed at 153.32 ppm. The methyl carbon at the 8-position of the quinazoline ring resonated at 18.01 ppm. The methylene (–CH_2_–) carbon at the 2-position of the morpholine ring appeared at 58.82 ppm, whereas those at the 3-, 5-, and 6-positions were observed at 49.76, 60.30, and 71.64 ppm, respectively. The carbons of the two methyl groups at the 6-position of the morpholine moiety resonated at 24.77 ppm. The aromatic carbons appeared within the range of 116.01–150.70 ppm. The ESI-MS spectrum of compound **4d** exhibited a molecular ion peak at *m*/*z* 258.11, corresponding to [M+H]^+^ for C_15_H_19_N_3_O (calculated *m*/*z* 258.16).

### 2.2. In Vitro Effects on Myogenic Markers and Cell Viability

The effects of the synthesized compounds on C2C12 cell viability were evaluated using the MTT assay following treatment at 1 µM and 5 µM concentrations for 48 h. Table 1 presents the in vitro viability percentages of the tested ML-167 derivatives.

**Figure 2:**
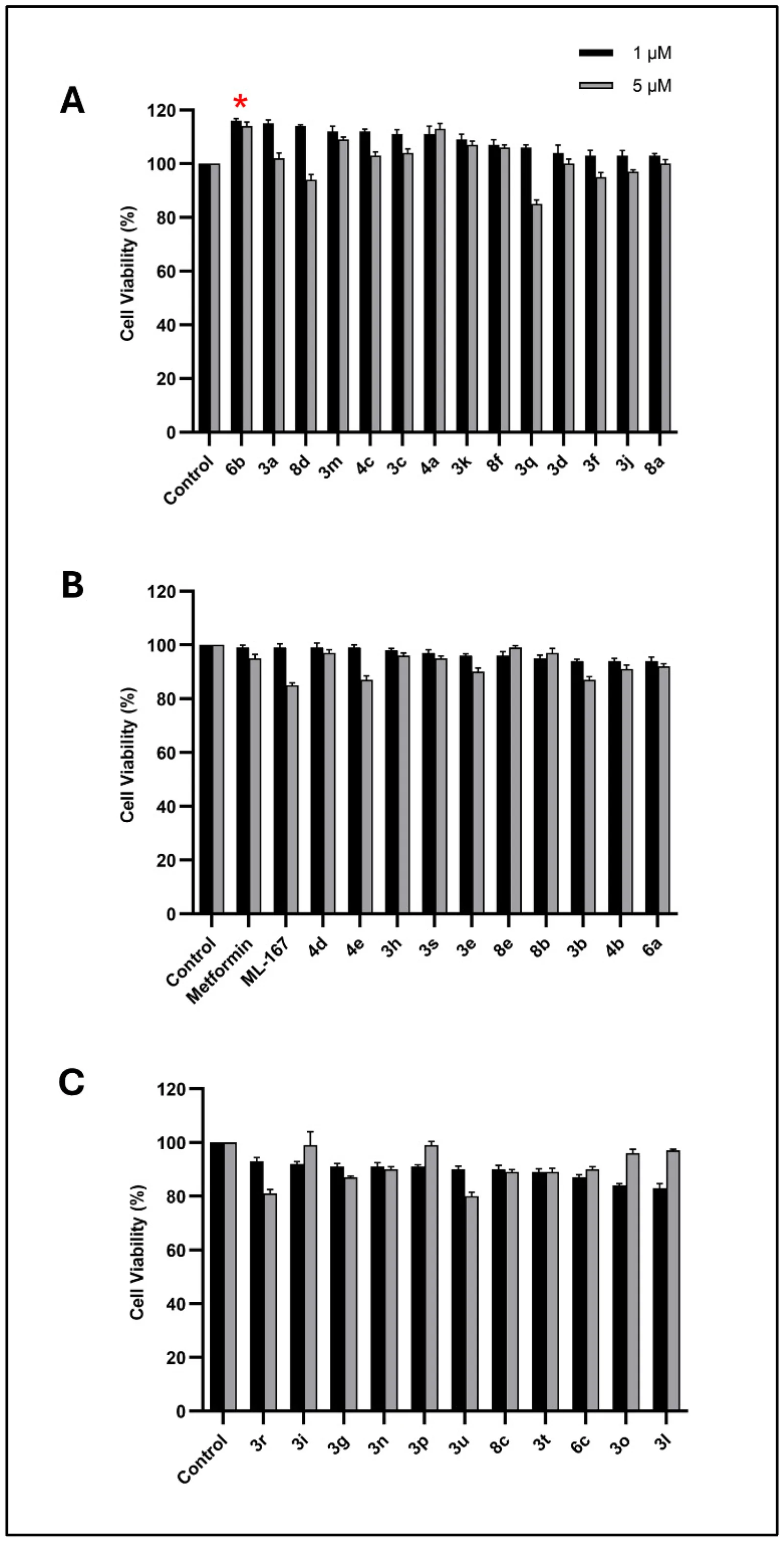
Effects of synthesized compounds on C2C12 cell proliferation. (**A**–**C**) Cell proliferation of C2C12 myoblasts was evaluated by MTT assay following treatment with synthesized compounds at 1 µM and 5 µM concentrations for 48 h. Data are expressed as percentage of the control group and presented as mean ± SD of three independent experiments (*n* = 3). A gradual decrease in cell proliferation values was observed from panel A to panel C. Black bars indicate 1 µM and gray bars indicate 5 µM concentrations. Statistical significance was determined by one-way ANOVA followed by Dunnett’s multiple comparisons test (* *p* < 0.05 vs. control).

The MTT assay result showed that various derivatives increased C2C12 cell survival at 1 μM relative to the control group, with a subset of compounds providing increases between 11% and 16%. Notably, compounds **3a**, **3c**, **3m**, **4a**, **4c**, **8a**, **8f** and **6b** exhibited consistent improvements in cell viability, and some maintained stable cell viability profiles at 5 µM, indicating acceptable tolerability across concentrations. Conversely, ML-167 compound as parent compound did not improve cell viability under same conditions, underscoring the benefit of structural optimization. Based on cell viability data, compounds exhibiting acceptable cell viability profiles were further evaluated for their effects on myogenic differentiation (Figure 2).

**Figure 3:**
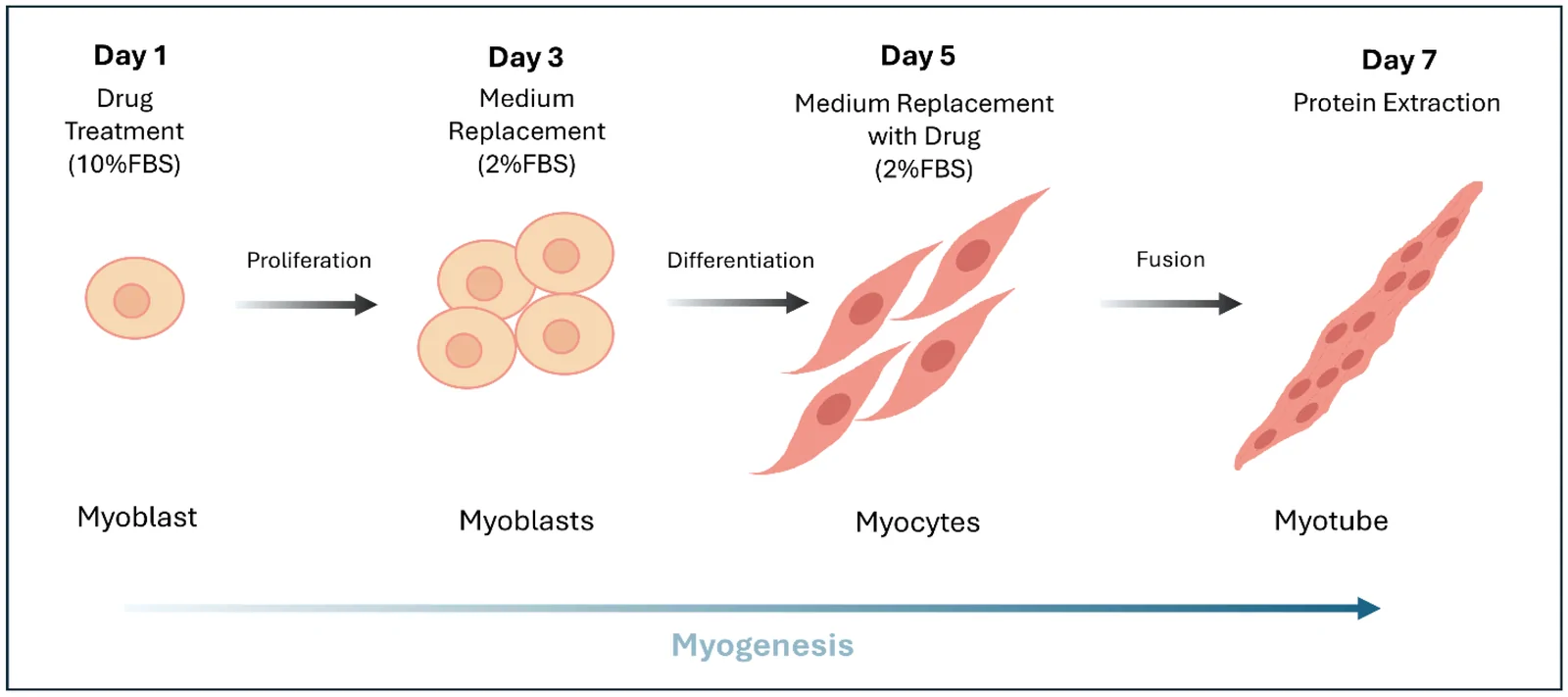
Experimental set-up for the differentiation of C2C12 cells in Western blot assay.

To evaluate the promyogenic potential of the synthesized compounds, an in vitro screening assay was established using differentiated C2C12 myotubes. The expression of the myogenic markers MyoG and MYH3 was analysed by Western blotting after 4 days of differentiation (Figure 3).

**Figure 4:**
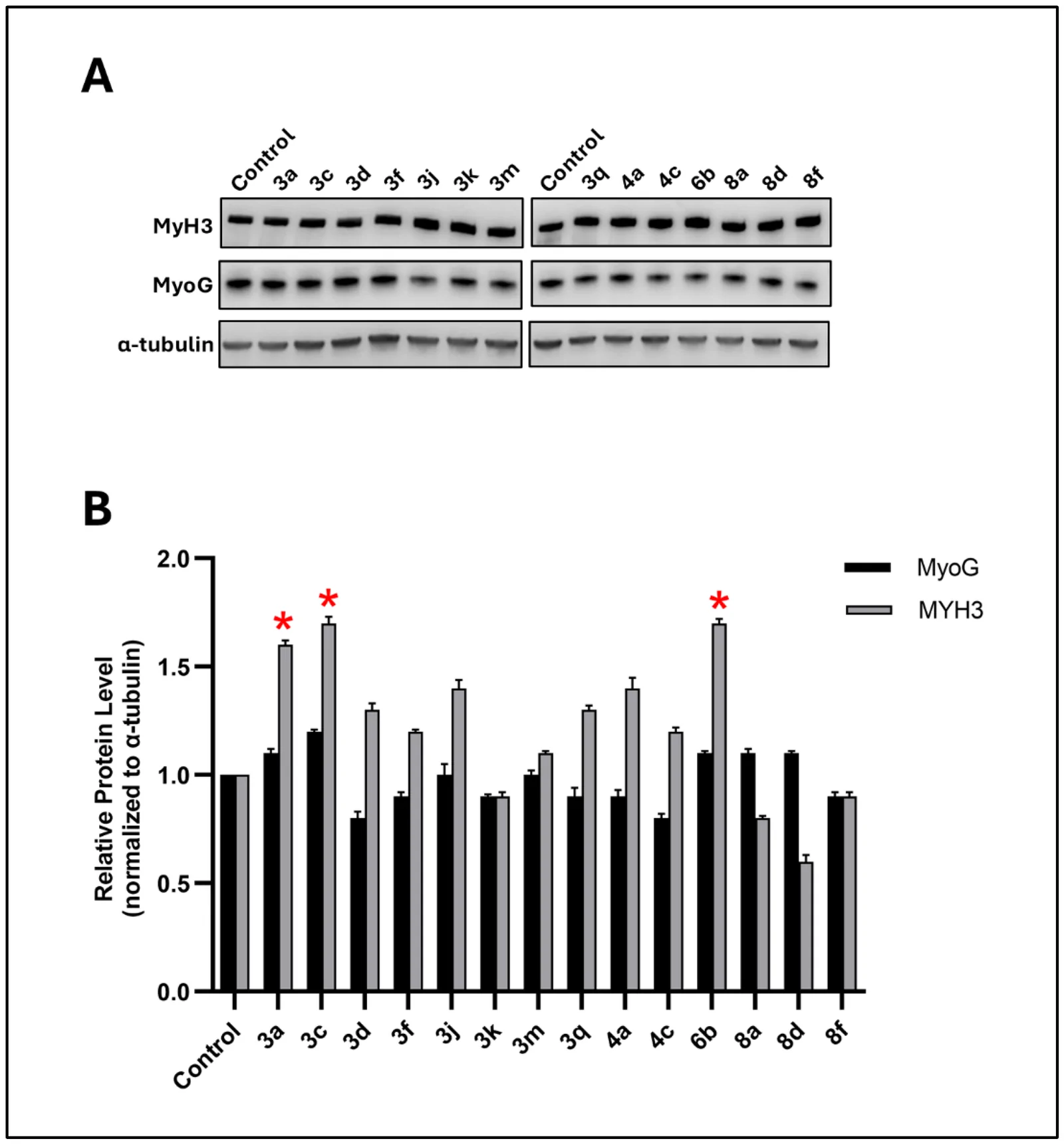
Myogenic differentiation effect of ML-167 compounds on C2C12. (**A**) Representative immunoblot images showing MyoG and MYH3 expression in differentiated C2C12 cells treated with selected compounds. (**B**) Relative expression levels of differentiation markers, MyoG and MYH3, indicating enhanced expression with selected ML-167 derived compounds (**3a**, **3c**, **3d**, **3f**, **3j**, **3k**, **3m**, **3q**, **4a**, **4c**, **6b**, **8a**, **8d**, **8f**). Quantification represents the mean ± SD of three independent experiments (*n* = 3). Statistical significance was determined using an unpaired two-tailed Student’s *t*-test, with * *p* < 0.05 considered statistically significant.

Western blot analysis revealed that compounds **3a**, **3c**, and **6b** significantly increased MYH3 expression (1.6–1.7-fold relative to control), indicating enhanced late-stage myotube maturation and muscle fiber formation. Compounds **3c** and **6b** produced the strongest induction of MYH3, suggesting robust promyogenic activity. In contrast, MyoG expression remained relatively stable, at or slightly above control levels, implying that these compounds primarily promote terminal myogenic differentiation while maintaining early myogenic signalling (Figure 4). The marked MYH3 induction observed with **6b**, together with its favourable C2C12 viability profile, provided additional support for its subsequent in vivo evaluation. This finding is consistent with previous reports showing that small-molecule modulation of muscle-regulatory pathways can promote myogenic differentiation and attenuate muscle atrophy. For example, the miR-29b inhibitor TGP-29b-066 was reported to promote muscle regeneration and attenuate dexamethasone-, angiotensin II-, and TNF-α-induced muscle atrophy [23]. In the present study, the increase in MYH3 expression further supports the potential of ML-167 derivatives to influence the myogenic component of muscle wasting. Mechanistically, the observed increase in MYH3 expression may indicate that ML-167 derivatives promote or preserve the myogenic differentiation program, thereby supporting myotube maturation and muscle fiber maintenance. MYH3 encodes the embryonic myosin heavy chain, a skeletal muscle-specific contractile protein that is expressed during skeletal muscle development and regeneration and is closely associated with myogenic differentiation [24]. Experimental evidence further indicates that MYH3 plays an important role in skeletal muscle differentiation, with its loss affecting muscle fiber development and the expression of genes involved in myogenic differentiation [25]. Skeletal muscle regeneration requires coordinated myoblast differentiation, myofiber formation, and maturation, which are essential for restoration and maintenance of muscle structure and function [26]. In the present study, the increased MYH3 expression observed following treatment with **3a**, **3c**, and **6b** was accompanied by improved muscle strength and muscle volume in vivo, suggesting that enhanced myogenic activity may contribute to the observed muscle-protective effects. Furthermore, the favourable metabolic effects observed particularly with **6b** may additionally support muscle preservation by improving the metabolic environment associated with muscle wasting. Taken together, these findings suggest that the muscle-protective effects of ML-167 derivatives may involve enhanced myogenic differentiation together with improved metabolic homeostasis.

### 2.3. Structure-Activity Relationship (SAR) Studies

For SAR evaluation, the MTT assay was performed at 1 µM, as this concentration reflects better intrinsic compound-target interactions with fewer nonspecific effects. This interpretation focused on cell viability data together with molecular docking scores and ligand efficiency values. Among the 4-morpholino quinazoline derivatives, compounds **3a**–**3c** with substitution at the 6 and 8-positions showed better activity compared to compound **3b**, the 7-chloro analogue, which exhibited lower activity. Within the 4-series (**4a**–**4e**), a similar positional effect was observed, where compounds **4a** and **4c** displayed higher cell viability than compound **4b**. Substitutional change at the C-8 position suggested that balanced hydrophobic groups usually improved the activity, while modification with strongly electron-withdrawing groups was less favourable. Further analysis indicates that replacement of the morpholine ring with a pyridin-4-yl group changed the SAR profile, with **6b** emerging as the most active compound within this series based on the observed C2C12 viability profile. Among the pyrazole derivatives, compound **8d** showed the highest activity. Collectively, these results demonstrate that biological activity is governed by the substitution pattern as well as the chemical nature of the attached heterocyclic moiety. The most active compounds of the series at 1 µM were **6b** (116%), **3a** (115%), **8d** (114%), **4c** (112%), **3m** (112%), and **3c** (111%). Molecular docking generally supported the observed trends; however, docking scores did not consistently correlate with cell viability, indicating that cellular activity is influenced by additional biological and physicochemical factors. Detailed SAR analysis together with docking interactions and binding energies is provided in Table S1 and Figure S1d.

### 2.4. Physicochemical Properties

The physicochemical properties of the synthesized compounds are summarized in Table S2. The molecular weights (MWs) of the synthesized derivatives ranged from 229.28 to 358.34 g/mol, indicating relatively low molecular sizes that are generally favourable for small-molecule drug development. The experimental solubility values ranged from 105.66 to 316.20 μM, demonstrating measurable aqueous solubility for all synthesized derivatives. The LogD values ranged from 0.91 to 2.94, reflecting a moderate degree of lipophilicity across the series. This range suggests a balanced hydrophilic–lipophilic character that may support membrane permeability while avoiding excessive lipophilicity, which can negatively affect aqueous solubility and other drug-like properties. Most of the synthesized derivatives exhibited a TPSA of 38.25 Å^2^, whereas the nitrile-containing analogues showed a higher TPSA of 62.04 Å^2^, consistent with the additional polar contribution of the nitrile-containing structures [27,28]. The higher TPSA values observed for these analogues may increase their polarity and potential for hydrogen-bonding interactions, although this may also influence membrane permeability and aqueous solubility. Overall, the compounds displayed physicochemical profiles within ranges commonly associated with drug-like small molecules. Their molecular weights, moderate LogD values, and relatively low-to-moderate TPSA values were broadly consistent with the physicochemical requirements described by Lipinski’s Rule of Five [29]. The observed balance between lipophilicity and polarity, together with measurable aqueous solubility, is particularly relevant for compounds intended to exert intracellular biological effects, as both adequate membrane permeability and sufficient aqueous compatibility are important considerations during early drug discovery [30,31]. Collectively, these physicochemical characteristics provide a rational basis for the subsequent biological evaluation of the synthesized derivatives.

### 2.5. In Vivo Validation of Lead Compounds in a Rat Model of Sarcopenia

Dexamethasone is considered an important model compound in preclinical studies examining the effects of glucocorticoids on muscle physiology. Previous experimental studies have demonstrated that Dexamethasone (DEX) treatment for a duration of two to four weeks induces degenerative changes in muscle and bone tissues [32,33,34,35]. Following disease model confirmation, animals in the treatment groups received the respective test compounds at a dose of 15 mg/kg once daily for 42 consecutive days according to the experimental design. The experimental groups, dosing regimens, and routes of administration are summarized in Table 2.

#### 2.5.1. Body Weight

**Figure 5:**
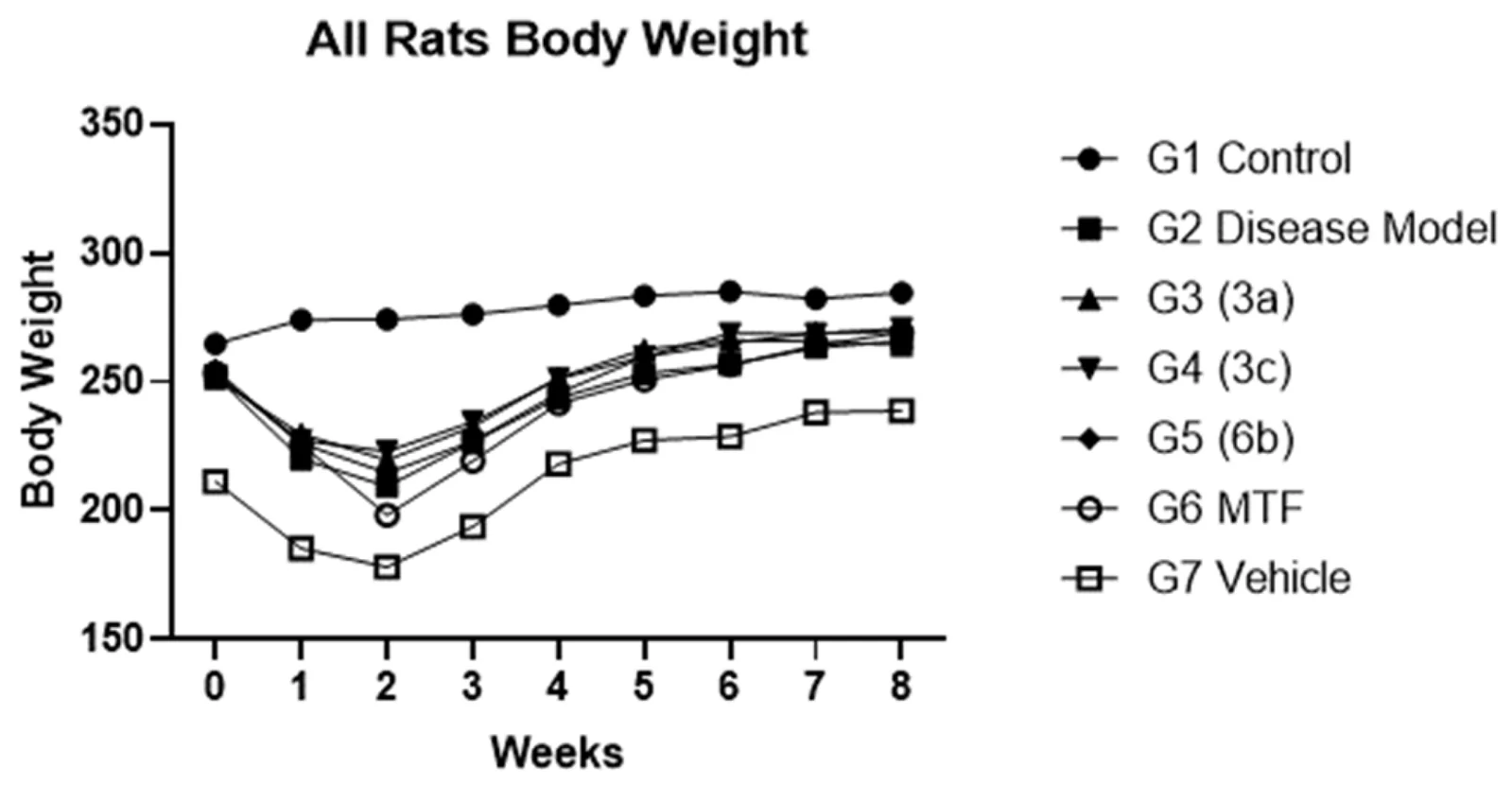
Mean body weight of rats in experimental groups.

Daily body weight tracking was considered an important parameter for evaluating the metabolic and physiological effects of the administered treatments. Similarly, previous studies investigating glucocorticoid or pharmacological interventions have also reported daily monitoring of body weight during the experimental period as a standard practice. This approach allows for a more accurate assessment and comparison of potential weight fluctuations among experimental groups [36]. During the two-week dexamethasone administration period used to induce sarcopenia, all groups exhibited a reduction in body weight. The observed reduction in body weight confirmed the successful establishment of the dexamethasone-induced sarcopenia-like muscle wasting model. The healthy control group (G1), body weight continued to increase, maintaining a normal growth trajectory. As shown in the results, After the induction phase of the model was completed, a gradual increase in body weight was observed in all groups, reflecting the effects of the treatment and/or recovery process [33] (Table 3 and Figure 5).

#### 2.5.2. Behavioural Analysis

##### Hanging Grid Graphics

**Figure 6:**
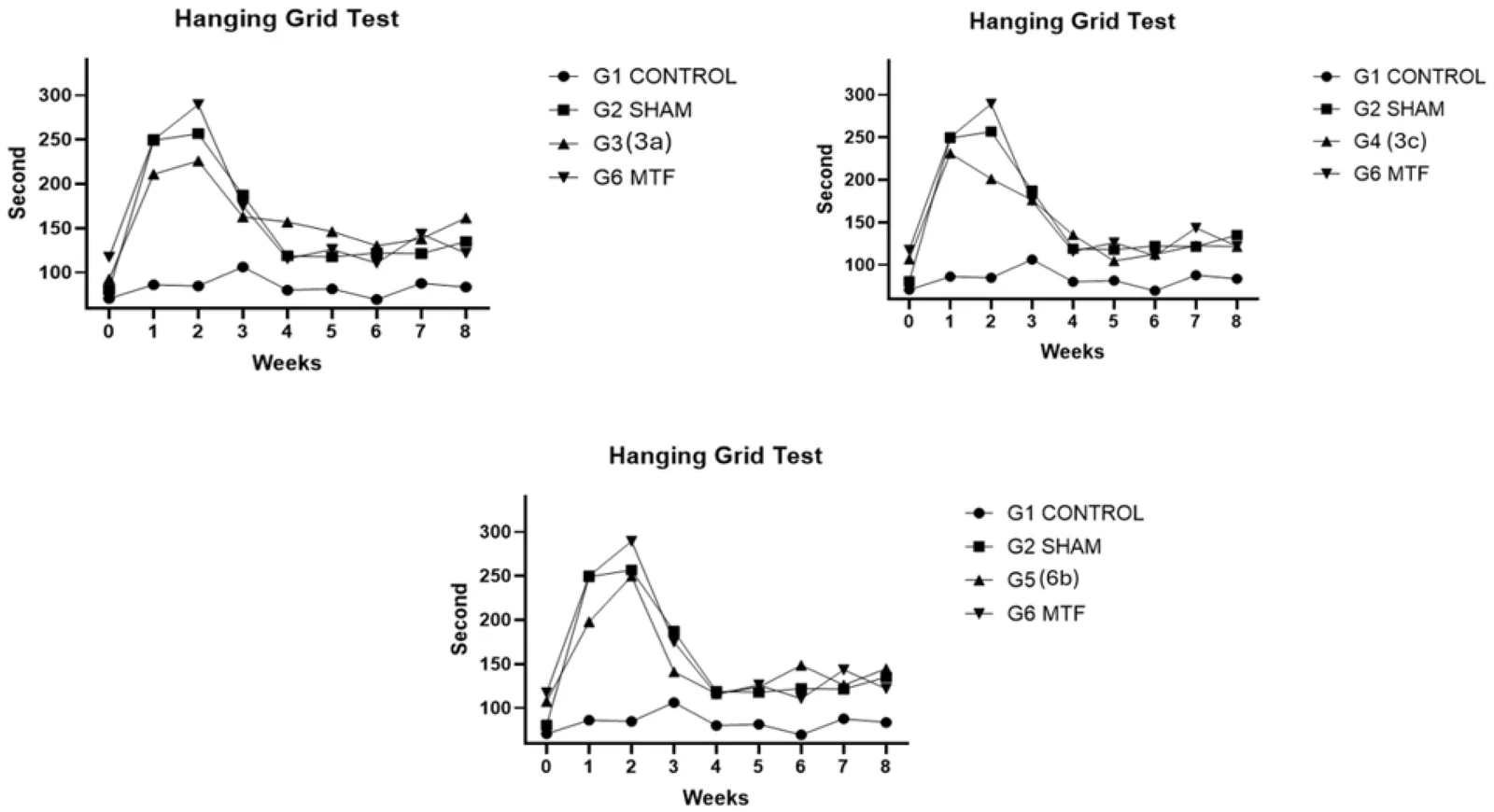
Effects of treatments on hanging grid test performance.

During the two-week dexamethasone administration period used for disease model induction, after completion of the model induction phase, the initial measurement was defined as 100%, and values obtained at the end of week 6 were expressed as a ratio relative to this baseline. The hanging time (in seconds) increased in all dex-treated groups, as also illustrated in the corresponding graph. Following disease model establishment, a decline in hanging duration was observed across all groups. The percentage decrease in hanging time by the end of the experimental period was 1.47% in the healthy control group (G1), 47.36% in the diseased control group (G2), 28.44% in the **3a** group (G3), 39.69% in the **3c** group (G4), 42.02% in the group **6b** (G5), 57.85% in the metformin-treated group (G6), and 64.14% in the vehicle group (G7) Figure 6. However, compared to the disease model group (G2), the SML compound groups (G3, G4, and G5) demonstrated superior performance, indicating a partial improvement in muscle endurance. These findings suggest that compounds exhibited muscle-protective effects comparable to, and in some cases exceeding, those of metformin, which is considered one of the most effective reference drugs for muscle preservation (Figure 6).

##### Forelimb Grip Strength Measurement

**Figure 7:**
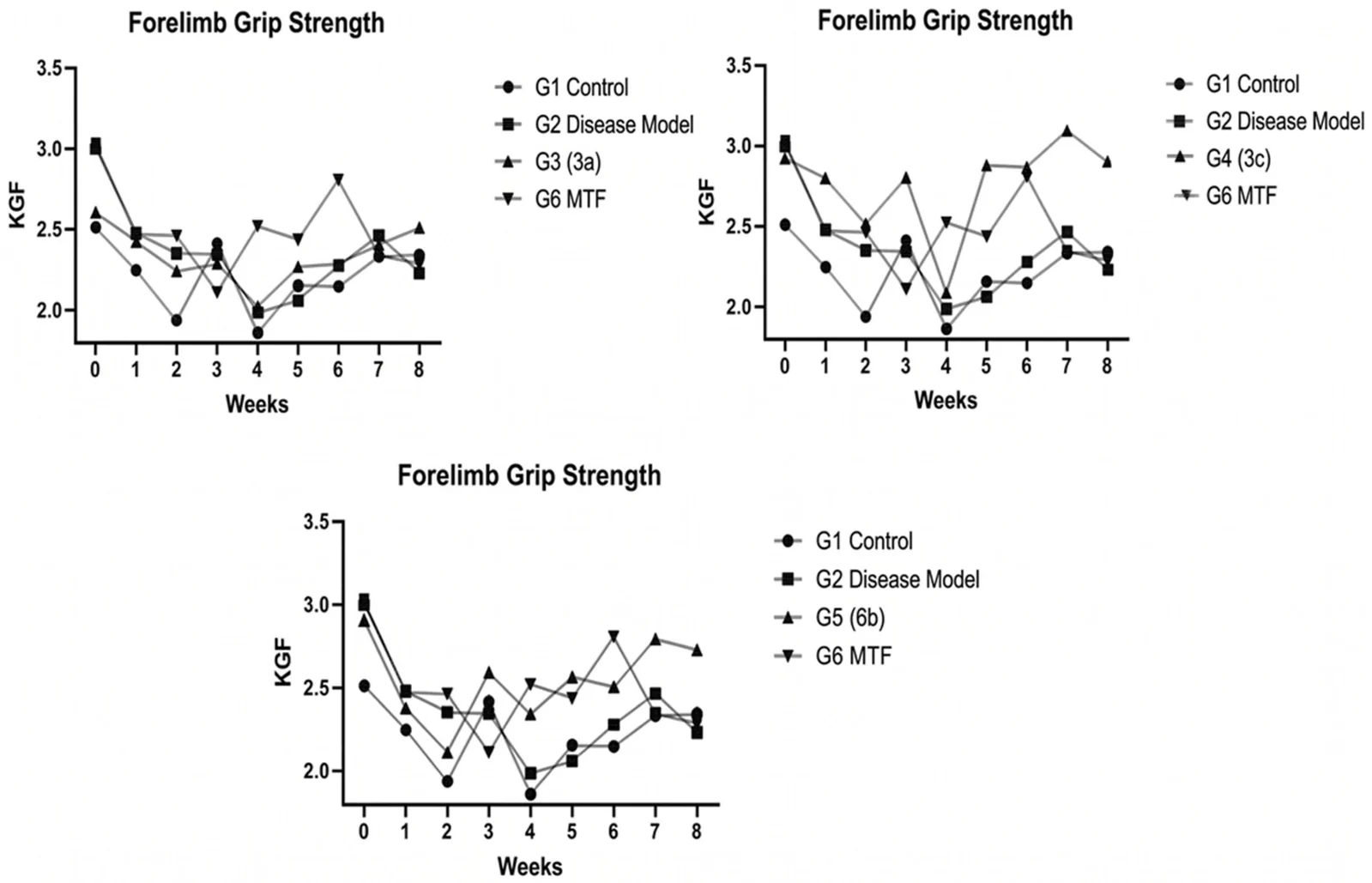
Effects of treatments on forelimb grip strength performance.

The Forelimb Grip Strength Test was performed using an AMF-20 digital force meter to evaluation muscle strength. For minimize the effects of inter-individual variability, the measured grip strength values were normalized to each animal’s body weight to account. Analysis of weekly measurements revealed that compound **3c** showed the greatest improvement in grip strength by week 8, followed by compounds **6b** and **3a**, respectively. Although **3c** showed the greatest improvement in grip strength, the overall efficacy profile of **6b** was supported by its more pronounced effects on skeletal muscle volume and metabolic parameters. These findings demonstrate a significant improvement in muscle function in the ML derivative-treated groups compared to the control group, suggesting that ML compounds may have a protective or restorative effect on muscle strength in a dexamethasone-induced sarcopenia-like muscle wasting model (Figure 7). These findings are in line with the growing evidence that pharmacological modulation of muscle catabolic pathways can improve muscle function. For instance, small-molecule targeting of MuRF1 has been reported to improve skeletal muscle contractility in a preclinical model [37]. Although the molecular targets and experimental models differ, the functional improvement observed with the ML-167 derivatives supports their potential to preserve muscle performance in sarcopenia.

#### 2.5.3. MRI Analysis of Muscle Volumes

MRI analysis of skeletal muscle mass showed a markedly decrease in skeletal muscle mass in the sarcopenia model group compared to the control group. Treatment with compounds, particularly **6b** compound, as well as metformin (MTF), effectively reversed sarcopenia-induced muscle loss. MRI measurements of the gluteus maximus, quadriceps femoris, and longissimus dorsi revealed that **6b** and MTF achieved the greatest restoration of individual muscle mass, exceeding or reaching control values. Consistently, total muscle mass assessed by MRI was significantly improved in the **6b** and MTF groups, whereas **3c** and **3a** showed moderate recovery and the vehicle group remained below control levels. Overall, MRI analysis identified **6b** as the most effective compound for restoring skeletal muscle volume among the tested ML-167 derivatives (Tables S3–S5 and Figure S1e). This pronounced effect on muscle volume, together with the favourable metabolic profile observed in the biochemical analyses, provided a strong basis for prioritizing **6b** as the overall lead candidate. This finding is comparable in direction to previous observations with small-molecule inhibition of 15-PGDH, which increased skeletal muscle mass, strength, and exercise performance in aged mice [38]. However, the present study evaluated muscle volume in a dexamethasone-induced sarcopenia model, and therefore direct quantitative comparison between the two approaches is not possible.

#### 2.5.4. Blood and Biochemical Analysis

Serum biochemical parameters were evaluated in the healthy control, sarcopenia disease model, **3a**, **3c**, **6b**, metformin, and vehicle groups (Table S6). CK and CK-MB were elevated in the sarcopenia disease model, whereas treatment with **3c** and **6b** reduced their levels, with moderate improvement observed for **3a** and metformin. Triglycerides were increased, whereas total cholesterol and HDL were reduced in the sarcopenia disease model. Treatment with **3c** and **6b** reduced triglycerides, with **6b** additionally improving HDL and total cholesterol. Total protein and albumin were decreased in the sarcopenia model and partially restored by **3a** and **3c**. Fasting glucose was elevated in the sarcopenia model, with the greatest reduction observed in the **6b** group, followed by **3c** and **3a**. Taken together, the biochemical findings further support **6b** as the overall lead candidate, particularly because its effects extended beyond muscle-associated biomarkers to metabolic parameters. Iron and zinc levels were reduced in the sarcopenia model and partially restored following treatment.

#### 2.5.5. Haematological Parameters

Platelets contribute to haemostasis and tissue regeneration through the release of growth factors and cytokines involved in muscle repair [39]. In the present study, platelet counts were reduced in the disease group (964.50 vs. 820.43), whereas treatment with the selected compounds partially restored platelet levels, with compounds **6b** (900.50) and **3a** (894.00) showing the greatest recovery toward the healthy control values (Table S7). These findings can be interpreted differently depending on the pathophysiological stage of the model. During chronic muscle injury with active inflammation and regeneration, platelet counts may be slightly increased [39,40]. In contrast, advanced muscle atrophy with mitochondrial dysfunction and impaired anabolic activity may result in reduced platelet counts [40,41].

#### 2.5.6. Histopathological Findings

Histopathological analysis revealed marked differences among the experimental groups. The highest pathology score (3.0) was observed in the disease model and vehicle groups, indicating severe muscle degeneration. In contrast, minimal histopathological changes were observed in the control group (score: 0.125). Treatment with compounds and MTF substantially reduced muscle pathology compared with the disease model. Among the treatments, **3c** demonstrated the lowest mean pathology score (1.375), suggesting the most consistent histological improvement. Thus, although **3c** demonstrated the strongest histopathological protection, the broader in vivo profile of **6b**, particularly its effects on muscle volume and metabolic parameters, supported its prioritization as the overall lead compound. **3a** and **6b** also showed significant reductions in muscle damage (1.5 each), while MTF produced a moderate protective effect (1.71). Overall, these findings indicate that these compounds, particularly **3c**, effectively attenuate sarcopenia induced muscle pathology, whereas vehicle treatment fails to confer histological protection (Table S8 and Figure S2e).

## 3. Materials and Methods

### 3.1. General Methods

All reagents and solvents were purchased commercially from Sigma-Aldrich and BLD Pharm and used without further purification. HPLC- or LC–MS-grade methanol, acetonitrile, and formic acid, as appropriate, were purchased from Merck (Darmstadt, Germany). Reaction progress was monitored either by thin-layer chromatography (TLC) on silica gel plates using a CAMAG TLC system (CAMAG, Muttenz, Switzerland) or by LC–MS using a Dionex UltiMate 3000 UHPLC system coupled to a TSQ Quantum Access MAX triple-quadrupole mass spectrometer (Thermo Fisher Scientific, Waltham, MA, USA). Compounds were purified by automated flash column chromatography using puriFlash® 5.020 and puriFlash® 5.250 systems (Interchim, Montluçon, France). The purity of the isolated compounds was assessed by LC–UV–MS using the aforementioned UHPLC–MS system equipped with a Vanquish UV detector (Thermo Fisher Scientific, Waltham, MA, USA). 1H and 13C NMR spectra were recorded in DMSO-*d6* using a Bruker Avance 500 MHz spectrometer (Bruker BioSpin, Rheinstetten, Germany). Chemical shifts (δ) are reported in parts per million (ppm) relative to tetramethylsilane (TMS).

### 3.2. General Synthesis of ML-167 Derivatives

#### 3.2.1. General Synthesis of ML-167 Derivatives (**3a**–**3u**)

The appropriate quinazoline derivative 1 (5 mmol) and amine derivative 2 (5 mmol) were stirred in dichloromethane (10 mL) at 0 °C for 2 h. Reaction progress was monitored by TLC or LC–MS. After completion, the reaction mixture was extracted with dichloromethane, dried over MgSO_4_, concentrated under reduced pressure, and purified by flash chromatography (hexane/ethyl acetate, 1:1). Compounds **3a**–**3u** were obtained in 67–84% yields.

##### 4-(6-Chloroquinazolin-4-yl)morpholine (**3a**)

White solid, Yield: 79%, ^1^H NMR (500 MHz, DMSO) δ 9.60 (s, 1H), 8.65 (s, 1H), 8.03–7.95 (m, 1H), 7.84 (d, *J* = 2.0 Hz, 1H), 3.80–3.78 (m, 4H), 3.06–3.03 (m, 4H). ^13^C NMR (126 MHz, DMSO) δ 163.28, 154.42, 150.48, 133.79, 130.74, 130.05, 124.60, 117.00, 63.63, 43.02. (ESI-MS): *m*/*z* 250.04 [M+H]^+^.

##### 4-(7-Chloroquinazolin-4-yl)morpholine (**3b**)

White solid, Yield: 75%, ^1^H NMR (500 MHz, DMSO) δ 8.64 (s, 1H), 8.05 (d, *J* = 9.0 Hz, 1H), 7.85 (d, *J* = 2.3 Hz, 1H), 7.54 (dd, *J* = 8.9, 2.3 Hz, 1H), 3.91–3.61 (m, 4H), 3.09–3.03 (m, 4H). ^13^C NMR (126 MHz, DMSO) δ 163.66, 155.04, 152.62, 137.60, 127.92, 127.00, 126.24, 114.61, 66.32, 49.89. (ESI-MS): *m*/*z* 250.01 [M+H]^+^.

##### 4-(8-Chloroquinazolin-4-yl)morpholine (**3c**)

White solid, Yield: 80%, ^1^H NMR (500 MHz, DMSO) δ 9.30 (s, 1H), 8.72 (s, 1H), 8.00 (dd, *J* = 8.0, 2.7 Hz, 1H), 7.51 (t, *J* = 8.0 Hz, 1H), 3.77 (t, *J* = 5.9 Hz, 4H), 3.07 (t, *J* = 5.0 Hz, 4H). ^13^C NMR (126 MHz, DMSO) δ 164.25, 154.63, 148.19, 133.25, 131.92, 126.04, 125.13, 117.40, 66.44, 50.29. (ESI-MS): *m*/*z* 250.03 [M+H]^+^.

##### 4-(8-Methylquinazolin-4-yl)morpholine (**3d**)

White solid, Yield: 74%, ^1^H NMR (500 MHz, DMSO) δ 8.74 (s, 1H), 7.89 (d, *J* = 8.4 Hz, 1H), 7.73 (d, *J* = 7.0 Hz, 1H), 7.47 (dd, *J* = 8.4, 7.1 Hz, 1H), 3.86–3.80 (m, 4H), 3.74–3.69 (m, 4H), 2.66 (s, 3H); ^13^C NMR (126 MHz, DMSO) δ 164.86, 153.31, 150.66, 136.44, 133.11, 125.62, 123.30, 116.09, 66.48, 50.45, 17.97. (ESI-MS): *m*/*z* 230.09 [M+H]^+^.

##### 4-Morpholinoquinazoline-8-carbonitrile (**3e**)

White solid, Yield: 75%, ^1^H NMR (500 MHz, DMSO) δ 8.73 (s, 1H), 8.37 (d, *J* = 8.2 Hz, 1H), 8.33 (d, *J* = 8.5 Hz, 1H), 7.63 (dd, *J* = 8.3, 8.1 Hz, 1H), 3.92–3.80 (m, 4H), 3.80–3.69 (m, 4H). 13C NMR (126 MHz, DMSO) δ 163.47, 155.73, 152.16, 139.24, 131.44, 125.49, 117.39, 115.99, 110.83, 66.44, 49.92. ESI-MS): *m*/*z* 241.07 [M+H]^+^.

##### 4-(8-(Trifluoromethyl)quinazolin-4-yl)morpholine (**3f**)

White solid, Yield: 71%, ^1^H NMR (500 MHz, DMSO) δ 8.74 (s, 1H), 8.31 (d, *J* = 8.4 Hz, 1H), 8.22 (d, *J* = 7.4 Hz, 1H), 7.65 (t, *J* = 7.9 Hz, 1H), 3.79 (q, *J* = 3.0 Hz, 8H). ^13^C NMR (126 MHz, DMSO) δ 163.93, 154.69, 148.83, 131.62, 131.58, 131.54, 131.49, 130.82, 125.76, 125.53, 125.41, 125.30, 124.82, 123.24, 116.43, 66.44, 50.11. (ESI-MS): *m*/*z* 283.85 [M+H]^+^.

##### 4-(8-Chloro-2-methylquinazolin-4-yl)morpholine (**3g**)

White solid, Yield: 70%, ^1^H NMR (500 MHz, DMSO) δ 7.87 (d, *J* = 8.0 Hz, 2H), 7.35 (t, *J* = 8.0 Hz, 1H), 3.71 (dd, *J* = 5.7, 3.6 Hz, 4H), 3.64 (dd, *J* = 5.7, 3.6 Hz, 4H), 2.51 (s, 3H). 13C NMR (126 MHz, DMSO) δ 164.55, 163.47, 148.70, 133.08, 131.42, 125.09, 124.93, 115.70, 66.44, 50.34, 26.78. (ESI-MS): *m*/*z* 264.04 [M+H]^+^.

##### (S)-4-(6-Chloroquinazolin-4-yl)-2-methylmorpholine (**3h**)

White solid, Yield: 75%, ^1^H NMR (500 MHz, DMSO) δ 8.71 (s, 1H), 8.00 (d, *J* = 1.6 Hz, 1H), 7.99 (d, *J* = 3.1 Hz, 1H), 7.50 (d, *J* = 8.4 Hz, 1H), 4.24–4.13 (m, 2H), 3.94–3.87 (m, 1H), 3.77–3.64 (m, 2H), 3.31–2.93 (m, 2H), 1.15 (d, *J* = 6.4 Hz, 3H). ^13^C NMR (126 MHz, DMSO) δ 163.72, 154.33, 148.01, 132.88, 131.65, 125.69, 124.82, 117.12, 71.16, 65.81, 55.18, 18.70. (ESI-MS): *m*/*z* 263.97 [M+H]^+^.

##### (S)-4-(7-Chloroquinazolin-4-yl)-2-methylmorpholine (**3i**)

White solid, Yield: 68%, ^1^H NMR (500 MHz, DMSO) δ 8.70 (s, 1H), 7.99 (d, *J* = 1.6 Hz, 1H), 7.98 (d, *J* = 3.1 Hz, 1H), 7.49 (d, *J* = 8.4 Hz, 1H), 4.23–4.12 (m, 2H), 3.93–3.86 (m, 1H), 3.76–3.63 (m, 2H), 3.30–2.94 (m, 2H), 1.14 (d, *J* = 6.4 Hz, 3H). ^13^C NMR (126 MHz, DMSO) δ 163.22, 153.83, 147.50, 132.37, 131.14, 125.19, 124.32, 116.62, 70.65, 65.30, 54.68, 48.87, 18.20. (ESI-MS): *m*/*z* 264.06 [M+H]^+^.

##### (S)-4-(8-Chloroquinazolin-4-yl)-2-methylmorpholine (**3j**)

White solid, Yield: 77%, ^1^H NMR (500 MHz, DMSO) δ 8.71 (s, 1H), 8.02–8.00 (m, 1H), 7.99 (d, *J* = 3.1 Hz, 1H), 7.51 (t, *J* = 8.0 Hz, 1H), 4.24–4.13 (m, 2H), 3.94–3.87 (m, 1H), 3.77–3.64 (m, 2H), 3.32–2.93 (m, 2H), 1.15 (d, *J* = 6.4 Hz, 3H); ^13^C NMR (126 MHz, DMSO) δ 164.04, 154.65, 148.32, 133.19, 131.96, 126.01, 125.14, 117.44, 71.47, 66.12, 55.49, 49.69, 19.02. (ESI-MS): *m*/*z* 264.06 [M+H]^+^.

##### (S)-2-Methyl-4-(8-methylquinazolin-4-yl)morpholine (**3k**)

White solid, Yield: 71%, ^1^H NMR (500 MHz, DMSO) δ 8.67 (s, 1H), 7.91 (d, *J* = 8.3 Hz, 1H), 7.76 (d, *J* = 7.2 Hz, 1H), 7.50 (t, *J* = 7.8 Hz, 1H), 4.40 (s, 2H), 3.89 (d, *J* = 10.4 Hz, 1H), 3.58 (d, *J* = 11.6 Hz, 2H), 3.13–3.02 (m, 2H), 2.56 (s, 3H), 1.11 (d, *J* = 6.2 Hz, 3H). ^13^C NMR (126 MHz, DMSO) δ 163.56, 150.50, 144.93, 135.66, 135.17, 126.68, 124.71, 123.97, 71.66, 66.00, 47.83, 42.26, 18.94, 18.00. (ESI-MS): *m*/*z* 244.10 [M+H]^+^.

##### (S)-4-(2-Methylmorpholino)quinazoline-8-carbonitrile (**3l**)

White solid, Yield: 84%, ^1^H NMR (500 MHz, DMSO) δ 8.71 (s, 1H), 8.37 (d, *J* = 7.2 Hz, 1H), 8.34 (d, *J* = 8.4 Hz, 1H), 7.63 (t, *J* = 7.9 Hz, 1H), 4.28 (ddt, *J* = 22.5, 13.1, 2.2 Hz, 2H), 3.95–3.88 (m, 1H), 3.76–3.62 (m, 2H), 3.41 (ddd, *J* = 14.6, 11.7, 3.3 Hz, 1H), 3.07 (dd, *J* = 13.1, 10.3 Hz, 1H), 1.16 (d, *J* = 6.2 Hz, 3H). ^13^C NMR (126 MHz, DMSO) δ 163.27, 155.69, 152.15, 139.22, 131.50, 125.50, 117.39, 116.00, 110.75, 71.53, 66.11, 55.09, 49.34, 18.99. (ESI-MS): *m*/*z* 255.04 [M+H]^+^.

##### (S)-2-Methyl-4-(8-(trifluoromethyl)quinazolin-4-yl)morpholine (**3m**)

White solid, Yield: 78%, ^1^H NMR (500 MHz, DMSO) δ 8.72 (s, 1H), 8.31 (d, *J* = 8.5 Hz, 1H), 8.22 (d, *J* = 7.9 Hz, 1H), 7.65 (t, *J* = 7.9 Hz, 1H), 4.28–4.16 (m, 2H), 3.91 (dd, *J* = 11.7, 3.4, 1.6 Hz, 1H), 3.77–3.64 (m, 2H), 3.42–3.33 (m, 1H), 3.04 (dd, *J* = 13.1, 10.3 Hz, 1H), 1.15 (d, *J* = 6.3 Hz, 3H). 13C NMR (126 MHz, DMSO) δ 163.70, 154.67, 148.89, 131.54, 131.50, 131.45, 130.85, 127.58, 125.97, 125.74, 125.50, 125.41, 125.27, 124.80, 123.24, 121.07, 116.44, 71.49, 66.12, 55.28, 49.54, 18.99. (ESI-MS): *m*/*z* 298.02 [M+H]^+^.

##### (S)-4-(8-Chloro-2-methylquinazolin-4-yl)-2-methylmorpholine (**3n**)

White solid, Yield: 74%, ^1^H NMR (500 MHz, DMSO) δ 7.95 (s, 1H), 7.93 (s, 1H), 7.43 (t, *J* = 8.0 Hz, 1H), 4.17–4.07 (m, 2H), 3.91 (dd, *J* = 11.6, 3.4, 1.6 Hz, 1H), 3.77–3.66 (m, 2H), 3.25 (dd, *J* = 13.3, 11.5, 3.3 Hz, 1H), 2.94 (dd, *J* = 13.2, 10.2 Hz, 1H), 2.57 (s, 3H), 1.15 (d, *J* = 6.3 Hz, 3H). 13C NMR (126 MHz, DMSO) δ 164.38, 163.46, 148.74, 133.05, 131.40, 125.09, 124.96, 115.73, 71.46, 66.11, 55.64, 49.74, 26.79, 19.05. (ESI-MS): *m*/*z* 278.07 [M+H]^+^.

##### (2R,6R)-4-(6-Chloroquinazolin-4-yl)-2,6-dimethylmorpholine (**3o**)

White solid, Yield: 67%, ^1^H NMR (500 MHz, DMSO) δ 8.69 (s, 1H), 8.01–7.98 (m, 1H), 7.98–7.96 (m, 1H), 7.53–7.46 (m, 1H), 4.21 (d, *J* = 12.9 Hz, 2H), 3.73 (dqd, *J* = 12.3, 6.1, 1.9 Hz, 2H), 2.93 (dd, *J* = 13.0, 10.4 Hz, 2H), 1.13 (dd, *J* = 6.2, 1.0 Hz, 6H). ^13^C NMR (126 MHz, DMSO) δ 163.00, 153.81, 147.54, 132.34, 131.12, 125.18, 124.35, 116.63, 70.56, 54.14, 18.18. (ESI-MS): *m*/*z* 278.05 [M+H]^+^.

##### (2R,6R)-4-(7-Chloroquinazolin-4-yl)-2,6-dimethylmorpholine (**3p**)

White solid, Yield: 73%, ^1^H NMR (500 MHz, DMSO) δ 8.70 (s, 1H), 8.01–7.99 (m, 1H), 7.99–7.97 (m, 1H), 7.54–7.47 (m, 1H), 4.21 (d, *J* = 12.9 Hz, 2H), 3.74 (dqd, *J* = 12.3, 6.1, 1.9 Hz, 2H), 2.94 (dd, *J* = 13.0, 10.4 Hz, 2H), 1.14 (dd, *J* = 6.2, 1.0 Hz, 6H). ^13^C NMR (126 MHz, DMSO) δ 163.41, 154.22, 147.96, 132.75, 131.53, 125.59, 124.76, 117.04, 70.97, 54.55, 18.60. (ESI-MS): *m*/*z* 278.08 [M+H]^+^.

##### (2R,6R)-4-(8-Chloroquinazolin-4-yl)-2,6-dimethylmorpholine (**3q**)

White solid, Yield: 68%, ^1^H NMR (500 MHz, DMSO) δ 8.63 (d, *J* = 1.0 Hz, 1H), 7.95–7.89 (m, 2H), 7.47–7.40 (m, 1H), 4.15 (d, *J* = 12.9 Hz, 2H), 3.67 (dqd, *J* = 12.3, 6.1, 1.9 Hz, 2H), 2.87 (dd, *J* = 13.0, 10.4 Hz, 2H), 1.07 (dd, *J* = 6.2, 1.0 Hz, 6H). ^13^C NMR (126 MHz, DMSO) δ 163.82, 154.63, 148.37, 133.16, 131.94, 126.00, 125.17, 117.45, 71.38, 54.96, 19.00. (ESI-MS): *m*/*z* 278.07 [M+H]^+^.

##### (2R,6R)-2,6-Dimethyl-4-(8-methylquinazolin-4-yl)morpholine (**3r**)

White solid, Yield: 74%, ^1^H NMR (500 MHz, DMSO) δ 8.67 (s, 1H), 7.84 (d, *J* = 8.1 Hz, 1H), 7.68 (dt, *J* = 7.0, 1.2 Hz, 1H), 7.43 (dd, *J* = 8.4, 7.1 Hz, 1H), 4.15 (pd, J = 6.4, 3.0 Hz, 2H), 3.77 (dd, *J* = 13.0, 3.2 Hz, 2H), 3.46 (dd, *J* = 13.0, 6.7 Hz, 2H), 2.61 (s, 3H), 1.20 (s, 3H), 1.19 (s, 3H). 13C NMR (126 MHz, DMSO) δ 164.94, 153.31, 150.71, 136.36, 133.03, 125.45, 123.18, 115.89, 66.14, 54.39, 18.29, 18.03. (ESI-MS): *m*/*z* 258.02 [M+H]^+^.

##### 4-((2R,6R)-2,6-Dimethylmorpholino)quinazoline-8-carbonitrile (**3s**)

Cream solid, Yield: 70%, ^1^H NMR (500 MHz, DMSO) δ 8.69 (s, 1H), 8.40–8.30 (m, 2H), 7.63 (dd, *J* = 8.5, 7.3 Hz, 1H), 4.16 (ddt, *J* = 10.1, 6.5, 3.4 Hz, 2H), 3.92 (dd, *J* = 13.0, 3.2 Hz, 2H), 3.65 (dd, *J* = 13.1, 7.2 Hz, 2H), 1.18 (s, 3H), 1.17 (s, 3H). 13C NMR (126 MHz, DMSO) δ 163.44, 155.73, 152.23, 139.13, 131.46, 125.36, 117.45, 115.80, 110.70, 66.29, 53.71, 18.35. (ESI-MS): *m*/*z* 269.04 [M+H]^+^.

##### (2R,6R)-2,6-Dimethyl-4-(8-(trifluoromethyl)quinazolin-4-yl)morpholine (**3t**)

Yellowish solid, Yield: 73%, ^1^H NMR (500 MHz, DMSO) δ 8.70 (s, 1H), 8.31 (dd, *J* = 8.6, 1.4 Hz, 1H), 8.21 (d, *J* = 7.3 Hz, 1H), 7.65 (t, *J* = 7.9 Hz, 1H), 4.16 (td, *J* = 6.6, 3.1 Hz, 2H), 3.88 (dd, *J* = 13.0, 3.2 Hz, 2H), 3.61 (dd, *J* = 13.1, 7.0 Hz, 2H), 1.19 (s, 3H), 1.18 (s, 3H). 13C NMR (126 MHz, DMSO) δ 163.92, 154.70, 148.92, 131.49, 131.09, 130.80, 125.65, 124.65, 123.26, 121.08, 116.21, 66.24, 53.91, 18.32. (ESI-MS): *m*/*z* 312.09 [M+H]^+^.

##### (2R,6R)-4-(8-Chloro-2-methylquinazolin-4-yl)-2,6-dimethylmorpholine (**3u**)

White solid, Yield: 71%, ^1^H NMR (500 MHz, DMSO) δ 7.98–7.91 (m, 2H), 7.43 (t, *J* = 8.0 Hz, 1H), 4.16 (td, *J* = 6.6, 3.1 Hz, 2H), 3.77 (dd, *J* = 13.0, 3.1 Hz, 2H), 3.50 (dd, *J* = 13.0, 6.9 Hz, 2H), 2.57 (s, 3H), 1.21 (s, 3H), 1.20 (s, 3H). 13C NMR (126 MHz, DMSO) δ 164.63, 163.45, 148.73, 133.02, 131.34, 124.97, 124.83, 115.56, 66.23, 54.27, 26.78, 18.34. (ESI-MS): *m*/*z* 278.04 [M+H]^+^.

#### 3.2.2. General Synthesis of ML-167 Derivatives (**4a**–**4e**)

The appropriate quinazoline derivative 1 (3 mmol), amine derivative 2 (3 mmol), and Cs_2_CO_3_ (6 mmol) were refluxed in acetonitrile (10 mL) for 12 h. Reaction progress was monitored by TLC or LC–MS. After completion, the reaction mixture was cooled, extracted with dichloromethane, dried over MgSO_4_, concentrated under reduced pressure, and purified by flash chromatography (hexane/ethyl acetate, 1:1) to afford compounds **4a**–**4e** in 64–73% yields.

##### 4-(6-Chloroquinazolin-4-yl)-2,2-dimethylmorpholine (**4a**)

White solid, Yield: 66%, ^1^H NMR (500 MHz, DMSO) δ 8.70 (s, 1H), 8.04 (d, *J* = 1.8 Hz, 1H), 7.90 (d, *J* = 2.3 Hz, 2H), 3.89 (t, *J* = 3.8 Hz, 2H), 3.76 (t, *J* = 4.9 Hz, 2H), 3.66 (s, 2H), 1.27 (s, 6H). ^13^C NMR (126 MHz, DMSO) δ 163.59, 154.46, 150.55, 133.74, 130.72, 129.94, 124.56, 116.89, 71.78, 60.19, 58.15, 49.41, 24.79. (ESI-MS): *m*/*z* 278.03 [M+H]^+^.

##### 4-(7-Chloroquinazolin-4-yl)-2,2-dimethylmorpholine (**4b**)

White solid, Yield: 70%, ^1^H NMR (500 MHz, DMSO) δ 8.63 (s, 1H), 7.98 (d, *J* = 1.8 Hz, 1H), 7.86–7.84 (m, 1H), 7.83 (s, 1H), 3.82 (dd, *J* = 6.2, 3.8 Hz, 2H), 3.70 (t, *J* = 4.9 Hz, 2H), 3.60 (s, 2H), 1.21 (s, 6H). ^13^C NMR (126 MHz, DMSO) δ 162.85, 153.72, 149.81, 133.00, 129.99, 129.20, 123.82, 116.15, 71.04, 59.45, 57.41, 24.05. (ESI-MS): *m*/*z* 278.04 [M+H]^+^.

##### 4-(8-Chloroquinazolin-4-yl)-2,2-dimethylmorpholine (**4c**)

White solid, Yield: 64%, ^1^H NMR (500 MHz, DMSO) δ 8.70 (s, 1H), 7.99 (ddd, *J* = 10.2, 8.1, 1.2 Hz, 2H), 7.52 (t, *J* = 8.0 Hz, 1H), 3.84 (t, *J* = 3.8 Hz, 2H), 3.72 (t, *J* = 4.9 Hz, 2H), 3.63 (s, 2H), 1.22 (s, 6H). ^13^C NMR (126 MHz, DMSO) δ 164.50, 154.67, 148.32, 133.20, 131.97, 126.00, 124.96, 117.30, 71.77, 60.21, 58.38, 49.68, 24.72. (ESI-MS): *m*/*z* 278.05 [M+H]^+^.

##### 2,2-Dimethyl-4-(8-methylquinazolin-4-yl)morpholine (**4d**)

White solid, Yield: 73%, ^1^H NMR (500 MHz, DMSO) δ 8.69 (s, 1H), 7.84 (dd, *J* = 8.6, 1.3 Hz, 1H), 7.71–7.66 (m, 1H), 7.44 (d, *J* = 1.3 Hz, 1H), 3.85 (dd, *J* = 5.9, 4.0 Hz, 2H), 3.63 (t, *J* = 5.0 Hz, 2H), 3.53 (s, 2H), 2.62 (s, 3H), 1.23 (s, 6H). ^13^C NMR (126 MHz, DMSO) δ 165.11, 153.32, 150.70, 136.43, 133.08, 125.60, 123.12, 116.01, 71.64, 60.30, 58.82, 49.76, 24.77, 18.01. (ESI-MS): *m*/*z* 258.11 [M+H]^+^.

##### 4-(8-Chloro-2-methylquinazolin-4-yl)-2,2-dimethylmorpholine (**4e**)

White solid, Yield: 67%, ^1^H NMR (500 MHz, DMSO) δ 7.94 (dd, *J* = 8.4, 5.6, 1.3 Hz, 2H), 7.43 (t, *J* = 7.9 Hz, 1H), 3.84 (dd, *J* = 5.9, 4.0 Hz, 2H), 3.65 (dd, *J* = 6.0, 3.9 Hz, 2H), 3.55 (s, 2H), 2.57 (s, 3H), 1.24 (s, 6H). 13C NMR (126 MHz, DMSO) δ 164.87, 163.48, 148.72, 133.07, 131.42, 125.12, 124.75, 115.65, 71.70, 60.28, 58.48, 49.93, 26.77, 24.77. (ESI-MS): *m*/*z* 292.04 [M+H]^+^.

#### 3.2.3. General Synthesis of ML-167 Derivatives (**6a**–**6c**)

An appropriate quinazoline derivative 1 (1 mmol), pyridin-4-ylboronic acid 5 (1 mmol), Cs_2_CO_3_ (2 mmol), and Pd(dppf)Cl_2_ (0.2 mmol) were reacted in THF (3 mL) under a nitrogen atmosphere using microwave irradiation at 160 °C for 10 min. After cooling, the reaction mixture was extracted with chloroform, dried over MgSO_4_, concentrated under reduced pressure, and purified by flash chromatography (hexane/ethyl acetate, 1:1) to afford compounds **6a**–**6c** in 63–69% yields [42].

##### 6-Chloro-4-(pyridin-4-yl)quinazoline (**6a**)

White solid, Yield: 63%, ^1^H NMR (500 MHz, DMSO) δ 9.44 (s, 1H), 8.89 (s, 2H), 8.24 (d, *J* = 2.1 Hz, 1H), 8.07 (d, *J* = 9.0 Hz, 1H), 7.84–7.81 (m, 2H), 7.79 (d, *J* = 2.1 Hz, 1H). ^13^C NMR (126 MHz, DMSO) δ 165.54, 155.35, 150.89, 149.66, 143.85, 139.32, 129.38, 128.53, 127.22, 124.30, 120.68. (ESI-MS): *m*/*z* 242.01 [M+H]^+^.

##### 7-Chloro-4-(pyridin-4-yl)quinazoline (**6b**)

White solid, Yield: 65%, ^1^H NMR (500 MHz, DMSO) δ 9.36 (s, 1H), 8.81 (s, 2H), 8.15 (d, *J* = 2.1 Hz, 1H), 7.99 (d, *J* = 9.0 Hz, 1H), 7.75–7.73 (m, 2H), 7.71 (d, *J* = 2.1 Hz, 1H). ^13^C NMR (126 MHz, DMSO) δ 166.09, 155.91, 151.44, 150.22, 144.41, 139.88, 129.94, 129.08, 127.78, 124.86, 121.24. (ESI-MS): *m*/*z* 241.97 [M+H]^+^.

##### 8-Chloro-4-(pyridin-4-yl)quinazoline (**6c**)

White solid, Yield: 69%, ^1^H NMR (500 MHz, DMSO) δ 9.46 (s, 1H), 8.91 (s, 2H), 8.25 (d, *J* = 2.1 Hz, 1H), 8.09 (d, *J* = 9.0 Hz, 1H), 7.85–7.83 (m, 2H), 7.81 (d, *J* = 2.1 Hz, 1H). ^13^C NMR (126 MHz, DMSO) δ 165.13, 154.94, 150.48, 149.25, 143.44, 138.91, 128.97, 128.12, 126.81, 123.89, 120.27. (ESI-MS): *m*/*z* 241.95 [M+H]^+^.

#### 3.2.4. General Synthesis of ML-167 Derivatives (**8a**–**8f**)

An appropriate ML-167 derivative (**3a**–**3c**) (1 mmol), boronic acid derivative 7 (1 mmol), Cs_2_CO_3_ (2 mmol), and Pd(dppf)Cl_2_ (0.2 mmol) were reacted in DMF/H_2_O (3:1, 8 mL) under a nitrogen atmosphere using microwave irradiation at 160 °C for 90 min. After cooling, the reaction mixture was extracted with chloroform, dried over MgSO_4_, concentrated under reduced pressure, and purified by flash chromatography (hexane/ethyl acetate, 1:1) to afford compounds **8a**–**8f** in 60–70% yields [42].

##### 4-(6-(1*H*-Pyrazol-5-yl)quinazolin-4-yl)morpholine (**8a**)

White solid, Yield: 64%, ^1^H NMR (500 MHz, DMSO) δ 13.04 (s, 1H), 8.64 (s, 1H), 8.35 (s, 1H), 8.30 (d, *J* = 8.6 Hz, 1H), 7.86 (d, *J* = 8.4 Hz, 2H), 6.90 (d, *J* = 2.3 Hz, 1H), 3.82 (dd, *J* = 5.8, 3.5 Hz, 4H), 3.78 (dd, *J* = 5.8, 3.5 Hz, 4H). ^13^C NMR (126 MHz, DMSO) δ 164.28, 161.45, 153.83, 151.28, 149.63, 131.60, 130.93, 129.01, 120.62, 116.52, 102.85, 66.45, 50.26. (ESI-MS): *m*/*z* 282.08 [M+H]^+^.

##### 4-(7-(1*H*-Pyrazol-5-yl)quinazolin-4-yl)morpholine (**8b**)

White solid, Yield: 60%, ^1^H NMR (500 MHz, DMSO) δ 13.03 (s, 1H), 8.63 (s, 1H), 8.33 (s, 1H), 8.28 (d, *J* = 8.6 Hz, 1H), 7.85 (d, *J* = 8.4 Hz, 2H), 6.88 (d, *J* = 2.3 Hz, 1H), 3.81 (dd, *J* = 5.8, 3.5 Hz, 4H), 3.77 (dd, *J* = 5.8, 3.5 Hz, 4H). ^13^C NMR (126 MHz, DMSO) δ 163.38, 160.55, 152.93, 150.37, 148.73, 130.70, 130.02, 128.10, 119.72, 115.61, 101.94, 65.55, 49.35. (ESI-MS): *m*/*z* 282.07 [M+H]^+^.

##### 4-(8-(1*H*-Pyrazol-5-yl)quinazolin-4-yl)morpholine (**8c**)

White solid, Yield: 63%, ^1^H NMR (500 MHz, DMSO) δ 12.96 (s, 1H), 8.73 (s, 1H), 8.58 (s, 1H), 8.23 (s, 1H), 8.10 (d, *J* = 7.4 Hz, 1H), 7.83 (d, *J* = 8.3 Hz, 1H), 7.52 (t, *J* = 7.8 Hz, 1H), 3.79 (t, *J* = 4.6 Hz, 4H), 3.68 (t, *J* = 4.6 Hz, 4H). ^13^C NMR (126 MHz, DMSO) δ 164.17, 152.52, 147.66, 138.31, 130.45, 129.27, 128.44, 125.15, 122.31, 117.15, 115.92, 65.59, 49.61. (ESI-MS): *m*/*z* 282.06 [M+H]^+^.

##### 4-(6-(1*H*-Pyrazol-4-yl)quinazolin-4-yl)morpholine (**8d**)

White solid, Yield: 67%, ^1^H NMR (500 MHz, DMSO) δ 13.09 (s, 1H), 8.61 (s, 1H), 8.39 (s, 1H), 8.11 (d, *J* = 1.6 Hz, 1H), 8.10 (t, *J* = 1.8 Hz, 1H), 8.08–8.03 (m, 1H), 7.82 (d, *J* = 8.6 Hz, 1H), 3.82 (q, *J* = 3.7 Hz, 4H), 3.76 (q, *J* = 3.6 Hz, 4H); ^13^C NMR (126 MHz, DMSO) δ 164.07, 153.38, 150.38, 137.14, 131.49, 130.98, 129.11, 126.78, 120.90, 119.92, 116.80, 66.44, 50.24. (ESI-MS): *m*/*z* 282.05 [M+H]^+^.

##### 4-(7-(1*H*-Pyrazol-4-yl)quinazolin-4-yl)morpholine (**8e**)

White solid, Yield: 68%, ^1^H NMR (500 MHz, DMSO) δ 13.07 (s, 1H), 8.59 (s, 1H), 8.38 (s, 1H), 8.10 (d, *J* = 1.6 Hz, 1H), 8.08 (t, *J* = 1.8 Hz, 1H), 8.06–8.03 (m, 1H), 7.80 (d, *J* = 8.6 Hz, 1H), 3.80 (q, *J* = 3.7 Hz, 4H), 3.75 (q, *J* = 3.6 Hz, 4H). ^13^C NMR (126 MHz, DMSO) δ 163.16, 152.48, 149.47, 136.23, 130.58, 130.07, 128.20, 125.87, 119.99, 119.01, 115.90, 65.53, 49.33. (ESI-MS): *m*/*z* 282.06 [M+H]^+^.

##### 4-(8-(1*H*-Pyrazol-4-yl)quinazolin-4-yl)morpholine (**8f**)

White solid, Yield: 70%, ^1^H NMR (500 MHz, DMSO) δ 12.98 (s, 1H), 8.75 (s, 1H), 8.60 (s, 1H), 8.25 (s, 1H), 8.12 (d, *J* = 7.4 Hz, 1H), 7.85 (d, *J* = 8.3 Hz, 1H), 7.54 (t, *J* = 7.8 Hz, 1H), 3.81 (t, *J* = 4.6 Hz, 4H), 3.70 (t, *J* = 4.6 Hz, 4H); ^13^C NMR (126 MHz, DMSO) δ 165.07, 153.43, 148.57, 139.21, 131.36, 130.18, 129.35, 126.05, 123.21, 118.06, 116.83, 66.49, 50.52. (ESI-MS): *m*/*z* 282.08 [M+H]^+^.

### 3.3. Molecular Docking

Molecular docking was performed using the AlphaFold structure of P08574 (CY1_HUMAN) in the Schrödinger Maestro suite (2023-3) [43]. The Protein preparation was carried out with the Protein Preparation Wizard, including correction of bond orders, formal charges, disulfide bonds, terminal residues, addition of hydrogen atoms, protonation state assignment, and energy minimization using the OPLS4 force field [44]. Protonation states were adjusted to match the experimental pH conditions, and the OPLS4 force field was used to optimize the structure. For predicting the binding site, Schrödinger’s Binding Site Identification module was used. The two-dimensional chemical structures of the compounds were sketched in ChemDraw (22.2.0.3300) and saved in. mol format, then converted into 3D structures using the LigPrep module. These compounds were minimized using the OPLS4 force field [45]. Additionally, compounds were processed to produce tautomeric and relevant ionization states under physiological pH conditions (7.0 ± 2.0). A grid box generated using the Glide module and placed on the predicted binding site to define the docking region. For docking, standard precision (SP) mode with flexible ligand conformations was used, and docking outcomes were assessed using the Glide score.

### 3.4. Physicochemical Properties Experimental Procedures

#### 3.4.1. Chemicals, Instruments and Equipment

Diclofenac, progesterone, nicardipine, and desipramine hydrochloride were obtained from Sigma-Aldrich (St. Louis, MO, USA). Ammonium formate, sodium dihydrogen phosphate monohydrate (NaH_2_PO_4_·H_2_O), disodium hydrogen phosphate heptahydrate (Na_2_HPO_4_·7H_2_O), formic acid, and 1-octanol were obtained from Merck (Darmstadt, Germany). Dimethyl sulfoxide (DMSO), acetonitrile, and methanol were purchased from Merck (Darmstadt, Germany) or Isolab (Eschau, Germany). The acetonitrile and methanol used for chromatographic analyses were of LC–MS grade. Unless otherwise specified, all other chemicals and reagents were of analytical grade or higher and were used without further purification.

UHPLC–MS/MS analyses were performed using an Agilent 1290 Infinity II UHPLC system coupled to an Agilent 6475 triple-quadrupole mass spectrometer (Agilent Tech-nologies, Santa Clara, CA, USA). Chromatographic separation was achieved using a ZORBAX RRHD C18 analytical column (2.1 × 50 mm, 1.8 µm; Agilent Technologies).

Supporting laboratory equipment included an Eppendorf ThermoMixer Comfort, a Heidolph vortex mixer, a Heidolph magnetic stirrer, a DKsonic ultrasonic water bath, a Mettler Toledo SevenDirect SD20 pH meter, a Nüve NF 800R refrigerated centrifuge, a Radwag MYA 5.5Y microbalance, and a Sartorius Entris 224I-1S analytical balance. Standard laboratory glassware, adjustable-volume Eppendorf micropipettes, and 0.22 µm PTFE syringe filters were used for sample preparation. Samples and reagents were stored, as appropriate, in an Arçelik K78550NE refrigerator/freezer or a B Medical Systems U501 ultra-low-temperature freezer.

#### 3.4.2. Kinetic Aqueous Solubility

Kinetic solubility was determined by the shake-flask method. Briefly, 15 μL of each 10 mM DMSO stock solution was mixed with 485 μL PBS (100 mM, pH 7.4), incubated at 25 °C with shaking (1100 rpm) for 2 h, then centrifuged, filtered, diluted with internal standard, and analysed by UPLC–MS/MS. [46,47]. Progesterone and diclofenac served as low- and high-solubility reference compounds [48,49]. Experiments were performed in duplicate, and kinetic solubility was calculated from calibration curves.

#### 3.4.3. Lipophilicity (LogD) Determination

LogD was determined by the shake-flask *n*-octanol/PBS (1:1, *v*/*v*) method. Test solutions (1 mM) were equilibrated at 25 °C (2000 rpm, 2 h), centrifuged, and aliquots from each phase were diluted with internal standard (desipramine, 100 ng/mL in H_2_O/acetonitrile, 1:1, *v*/*v*) before UPLC–MS/MS analysis (5 μL injection). LogD values were calculated from dilution-corrected analyte peak-area ratios using Equation (1) [50,51,52]:

logD = log_10_((AREA_Oct_ × DF)/(AREA_Buf_ × DF)) (1)

Experiments were performed in duplicate using nicardipine as the reference compound.

### 3.5. In Vitro Experimental Procedures

#### 3.5.1. Cell Culture

The C2C12 mouse myoblast cell line (CRL-1772) was obtained from ATCC (Manassas, VA, USA). Cells were cultured in high-glucose DMEM (Gibco, Thermo Fisher Scientific, Waltham, MA, USA) supplemented with 10% fetal bovine serum (FBS; Gibco) and 1% penicillin–streptomycin (Gibco). Cultures were maintained at 37 °C in a humidified incubator with 5% CO_2_ and grown in 75 cm^2^ culture flasks.

#### 3.5.2. Cell Viability Assay

Cell viability was assessed using the MTT assay (SERVA Electrophoresis GmbH, Heidelberg, Germany). C2C12 cells were plated in 96-well plates at a density of 7 × 10^4^ cells per well and incubated for 24 h. The cells were then treated with the test compounds at final concentrations of 1 and 5 μM for 48 h, while control cells received culture medium alone. Subsequently, 10 μL of MTT solution (5 mg/mL in PBS; Gibco) was added to each well and incubated for 2 h at 37 °C. The medium was removed, and the resulting formazan crystals were dissolved in 100 μL of DMSO (AppliChem GmbH, Darmstadt, Germany). Absorbance was measured at 570 nm using a microplate reader (Agilent Technologies, Winooski, VT, USA). Cell viability was expressed as a percentage of the untreated control.

#### 3.5.3. Cell Lysis and Protein Extraction

For protein expression analysis, C2C12 cells were seeded into 6-well plates at a density of 3 × 10^5^ cells per well. After 24 h, the cells were treated with the test compounds at final concentrations of 1 and 5 μM for 48 h. For differentiation studies, cells at approximately 80% confluence were switched to differentiation medium consisting of high-glucose DMEM supplemented with 2% horse serum (Gibco) and 1% penicillin–streptomycin. The differentiation medium was refreshed every other day, and myotube formation was evaluated after 4 days. Cells were lysed using Cell Lytic M (Sigma-Aldrich, Burlington, MA, USA) reagent supplemented with a protease inhibitor cocktail (Abbkine, Wuhan, China). Lysates were centrifuged at 12,000× *g* for 20 min at 4 °C, and protein concentrations were determined using the Bradford protein assay (Bio-Rad, Hercules, CA, USA).

#### 3.5.4. Western Blot Analysis

Equal amounts of protein (25 μg) were mixed with 4× Laemmli buffer (Bio-Rad), heated at 95 °C for 5 min, and separated on 4–20% SDS–PAGE gels (Bio-Rad). Proteins were transferred to polyvinylidene difluoride (PVDF) membranes (Bio-Rad) and blocked in 5% non-fat milk in TBS-T (Tris-buffered saline with 0.2% Tween 20) for 30 min at room temperature. Membranes were incubated overnight at 4 °C with a 1:1250 dilution of anti-MyoG monoclonal antibody (ab124800, Abcam, Cambridge, UK) and MYH3 monoclonal antibody at a concentration of 1 μg/mL (ab124205, Abcam). For loading control, membranes were incubated for 1 h with anti-alpha-tubulin antibody (1:5000; ab52866). After washing with TBS-T, membranes were incubated with HRP-conjugated anti-rabbit secondary antibody (1:20,000; ab97051, Abcam). Protein bands were visualized using Clarity Western ECL substrate (Bio-Rad) and detected with a ChemiDoc imaging system (Bio-Rad). Band intensities were quantified using ImageJ 1.54f software).

#### 3.5.5. Statistical Analysis

All data were presented as mean ± standard deviation (SD). Statistical analyses were performed using GraphPad Prism 10.3.1 (GraphPad Software, San Diego, CA, USA). Differences between control and treated groups were analysed using Student’s *t*-test, and *p* < 0.05 was considered statistically significant.

### 3.6. In Vivo Experimental Procedures

#### 3.6.1. Ethical Approval

All experimental procedures were conducted in accordance with Directive 2010/63/EU for the protection of animals used for scientific purposes and were performed in compliance with the ARRIVE 2.0 guidelines. The study protocol was reviewed and approved by the Atatürk University Animal Experiments Local Ethics Committee (Approval No. 2200366213).

The experimental protocol including the study objectives, experimental design and statistical analysis plan was established before the initiation of the study as part of the animal ethics committee application. The protocol was developed based on an extensive review of the relevant literature and previous experience with the experimental model.

#### 3.6.2. Experimental Animals

Female Sprague–Dawley rats (*n* = 49), aged 12 weeks and weighing 252 ± 25 g, were obtained from the Atatürk University Medical Experimental Application and Research Center (ATADEM). Only clinically healthy animals were included in the study. Before the start of the experiment, animals underwent a 10-day acclimatization period under standard laboratory conditions.

Animals were housed in polycarbonate cages, with each experimental group distributed into two cages (4 and 3 animals per cage) to maintain social housing conditions. Wood shavings were used as bedding and nesting material. Rats had ad libitum access to standard laboratory chow (Bayramoğlu Yem, Erzurum, Turkiye) and drinking water. The animal room was maintained at 22 ± 2 °C with a relative humidity of 50–60% under a 12 h light/12 h dark cycle throughout the study.

#### 3.6.3. Study Design

Rats were randomly allocated to seven experimental groups (*n* = 7 per group) while ensuring comparable baseline body weights among groups. The groups were defined as follows; G1: Control; G2: Disease model; G3: Compound **3a**; G4: Compound **3c**; G5: Compound **6b**; G6: Metformin (MTF); G7: Vehicle.

The experimental unit was an individual rat, as all treatments, injections, behavioural assessments, blood collections, and tissue analyses were performed separately for each animal.

#### 3.6.4. Sample Size

The sample size was determined based on previous experience with the same experimental sarcopenia model in our laboratory and was considered sufficient to detect biologically relevant differences while minimizing the number of animals used in accordance with the principles of Replacement, Reduction, and Refinement (3Rs). A total of 49 animals were used, with seven animals allocated to each experimental group.

#### 3.6.5. Inclusion and Exclusion Criteria

Inclusion criteria were defined a priori and included clinically healthy rats of appropriate age and body weight. Before enrolment all animals underwent a general health examination including assessment of body condition, fur appearance, eyes, salivation, nasal discharge, defecation, skin lesions, ears and paws.

Animals showing signs of illness, abnormal clinical findings, or inadequate body weight were excluded before the experiment. During the study, animals were to be excluded if they developed severe clinical abnormalities, impaired defecation, marked distress, or other predefined health problems. No animals met the exclusion criteria during the experimental period; therefore, no exclusions were made from the analyses.

#### 3.6.6. Randomisation and Blinding

Animals were randomly assigned to experimental groups while balancing baseline body weights across groups. Control and treatment groups were handled in the same manner throughout the study to minimize potential confounding effects. The order of behavioural assessments and sample collection was kept consistent across groups.

Investigators responsible for compound administration were blinded to the identity of the test compounds. Investigators performing behavioural assessments, magnetic resonance imaging (MRI) evaluations and image analyses, histopathological examinations and scoring, haematological analyses, serum biochemical analyses, and data analyses were blinded to the experimental group allocation. Blinding was maintained throughout the respective assessment and analysis procedures to minimize potential observer bias.

#### 3.6.7. Experimental Procedures

Sarcopenia-like muscle wasting model was induced by daily intraperitoneal injections of dexamethasone (DEX) at a dose of 1 mg/kg for 14 consecutive days in groups G2–G7. DEX (MedChemExpress, Monmouth Junction, NJ, USA (Cat. No. HY-14648)) was dissolved in physiological saline containing 5% DMSO immediately before administration. Injection volume was adjusted according to individual body weight to provide 1 mL per 250 g rat.

Following DEX administration, oral treatments were continued for a total experimental duration of 42 days (6 weeks). The treatment groups received **3a** (15 mg/kg), **3c** (15 mg/kg), **6b** (15 mg/kg), or metformin (15 mg/kg) by oral gavage once daily at approximately 09:00 each morning. Compounds were synthesized by Career Henan Chemical Co. (Zhengzhou City, Henan Province, China). and formulated in Maisine CC or PEG 400 as vehicle components. The vehicle group received the corresponding vehicle solution in the same volume, whereas the control and disease model groups received an equal volume of physiological saline to ensure identical handling conditions.

Forelimb grip strength and hanging grid tests were performed on day 0 and subsequently once weekly on the same day of the week and at approximately the same time of day throughout the study.

Magnetic resonance imaging was performed on the final experimental day immediately before euthanasia. Blood samples were collected under anaesthesia immediately before euthanasia. Animals were anesthetized with ketamine/xylazine, and the depth of anaesthesia was confirmed by the absence of reflex responses. Blood was collected by intracardiac puncture after which animals were euthanized by cervical dislocation while under deep anaesthesia.

After blood collection, the gastrocnemius muscle from one hind limb was excised and fixed in formaldehyde for histological evaluation. Liver, heart, brain, skeletal muscle, femur, kidney, adipose tissue, pancreas, uterus and ovaries were rapidly frozen in liquid nitrogen and stored at −80 °C for subsequent molecular analyses.

#### 3.6.8. Outcome Measures

The primary outcome measures were forelimb grip strength and hanging grid test performance. Secondary outcome measures included haematological parameters, serum biochemical parameters, histopathological findings and magnetic resonance imaging outcomes.

#### 3.6.9. Body Weight Monitoring

Daily body weight monitoring was done throughout the experiment under consistent environmental conditions.

#### 3.6.10. Behavioural Assessment

Muscle function was evaluated weekly using forelimb grip strength and hanging tests. Grip strength was measured over three trials in which each rat was gently pulled backward by its tail while holding a grip strength measuring device. The highest and lowest values were removed, and the remaining values were normalized according to body weight. Relative grip strength (g/g) was calculated as follows: grip strength (kgf)/body weight (g) × 1000 [53,54]. Muscle endurance was assessed using the hanging grid test. A 45 cm × 45 cm wire grid apparatus was used for the experiment. Each rat was placed on a 45 × 45 cm wire, allowed to grasp the mesh with all four limbs, and the grid was then carefully inverted to allow the animal to hang in an upside-down position. Latency to fall was recorded in two trials separated by a 15-min rest interval, and the mean hanging time was used for analysis as a measure of muscle strength and endurance [55].

#### 3.6.11. Magnetic Resonance Imaging (MRI)

Anaesthetics was administered via intraperitoneal route on animals fixed on the supine position with parallel hind limbs. MRI was performed using a 3 Tesla clinical scanner (Magnetom Skyra; Siemens Healthineers, Erlangen, Germany). Rats were placed in the supine position with their hind legs extended backwards. T2-space (Sampling Perfection with Application optimized Contrast using different flip angle Evolution) sequence was obtained to view the bilateral quadriceps, adductor and iliopsoas muscles in coronal plane (TR: 5000 ms, TE: 396 ms, voxel size: 0.5 × 0.5 × 1 mm, 112 slices, slice spacing: 1.8 mm). The measurement of volumes of aforementioned muscles was performed upon manual segmentation method on Syngo via Workstation using freehand volume of interest (VOI).

#### 3.6.12. Histological Analysis

Muscle tissues were fixed in 10% formaldehyde for 48 h, processed routinely, embedded in paraffin, and sectioned at 4 μm. Sections were stained with haematoxylin and eosin (H&E) and examined under a light microscope (Olympus Optical Co. Ltd., Tokyo, Japan). The degree of muscle fiber atrophy was assessed using a semi-quantitative scoring system ranging from 0 to 3, based on the extent and severity of histomorphological alterations observed in the muscle tissue. Score 0 (no atrophy) was assigned to muscle tissue showing predominantly uniform and regularly arranged myofibers, with no evident fiber shrinkage or appreciable degenerative changes. Score 1 (mild atrophy) was assigned when most myofibers were preserved but occasional fibers showed mild reduction in size and/or focal mild hyalinization. Score 2 (moderate atrophy) was assigned when clear variation in myofiber size, moderate fiber shrinkage, and/or multifocal hyalinization and degenerative changes were observed. Score 3 (severe atrophy) was assigned when marked and widespread myofiber atrophy was accompanied by extensive hyalinization, fragmentation, and/or de-generative changes. The final score for each specimen was determined according to the overall extent and severity of the observed histomorphological alterations.

#### 3.6.13. Animal Care and Monitoring

Humane endpoint criteria were established before the study. Animals showing signs of severe pain, distress, marked weight loss, impaired mobility, or any condition compromising animal welfare would have been humanely euthanized. However, no animals met these criteria during the study.

#### 3.6.14. Statistical Analysis

All data are presented as mean ± standard deviation (SD). Statistical analyses were performed using GraphPad Prism 10.3.1 (GraphPad Software, San Diego, CA, USA). For comparisons involving more than two experimental groups one-way analysis of variance (ANOVA) followed by Dunnett’s multiple-comparisons test was used when treatment groups were compared with a common control group. For longitudinal measurements involving treatment and time two-way repeated-measures ANOVA followed by an ap-propriate multiple-comparisons test was applied. Given the significant differences in baseline body weight among the experimental groups, Week 0 body weight was included as a covariate in the statistical analysis to adjust for baseline imbalance when evaluating treatment-related changes. Student’s *t*-test was used only for comparisons between two independent groups where applicable. A *p*-value < 0.05 was considered statistically significant.

## 4. Conclusions

The present study identified novel ML-167 derivatives with promising anti-sarcopenic activity. Several compounds, including **3a**, **3c**, and **6b**, demonstrated a favourable biological profile by enhancing C2C12 myoblast viability and promoting a myogenic phenotype, accompanied by a 1.6–1.7-fold increase in MYH3 expression. Physicochemical and molecular modelling analyses further supported the drug-like properties of the most promising derivatives. In a dexamethasone-induced sarcopenia-like muscle wasting rat model, these compounds improved skeletal muscle mass, endurance, muscle strength, and histopathological integrity without evidence of renal or hepatic toxicity. Serum AST, ALT, ALP, GGT, creatinine, urea, and BUN levels, together with histopathological findings, were assessed as indicators of potential hepatic and renal toxicity, with no overt abnormalities observed within the parameters evaluated. Among the evaluated derivatives, compound **6b** demonstrated the most pronounced restoration of skeletal muscle volume by MRI, with an efficacy comparable to metformin, whereas compound **3c** provided the strongest histopathological protection. Taken together, the pronounced effects of **6b** on skeletal muscle volume and metabolic parameters, together with its favourable in vitro promyogenic profile, support its prioritization as the overall lead candidate among the evaluated ML-167 derivatives. Although the DEX-induced sarcopenia-like muscle wasting model does not fully recapitulate the complexity of age-related sarcopenia in humans, it is a well-established preclinical model for evaluating skeletal muscle dysfunction and potential therapeutic interventions. Given the glucocorticoid-induced nature of the experimental model, the present findings particularly support the potential application of these compounds in glucocorticoid-associated muscle wasting. More broadly, their combined effects on skeletal muscle volume, muscle strength, histopathological integrity, and metabolic parameters suggest potential applicability to conditions characterized by muscle loss and functional decline. Compared with approaches that primarily target a single aspect of muscle dysfunction, the present compounds may offer a potentially broader pharmacological profile by combining promyogenic activity with effects on muscle mass and metabolic homeostasis. However, direct comparative studies with established and emerging pharmacological interventions will be required to determine whether this profile provides a meaningful therapeutic advantage. While the identified lead candidate and other promising derivatives showed encouraging efficacy in the current in vivo study, their therapeutic potential remains to be confirmed through further investigation in complementary preclinical models and subsequent well-designed clinical studies. Therefore, the findings of the present study may contribute to the preclinical evaluation of novel pharmacological candidates for sarcopenia-associated muscle wasting. Collectively, these findings identify compound **6b** as the most promising overall lead candidate among the evaluated ML-167 derivatives for further investigation in glucocorticoid-associated muscle wasting, based on its combined effects on skeletal muscle volume, metabolic parameters, and promyogenic activity.

## Figures and Tables

**Table 1:** In vitro viability % activities of the tested synthesized ML-167 derivatives.

Entry	R_1_	R_2_	R_3_	Cell Viability (%)	
				1 μM	5 μM
Metformin	-	-	-	99	95
ML-167	-	-	-	99	85
(**3a**)	6-Cl	H	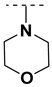	115	102
(**3b**)	7-Cl	H	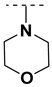	94	87
(**3c**)	8-Cl	H	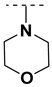	111	104
(**3d**)	8-Me	H	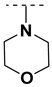	104	100
(**3e**)	8-CN	H	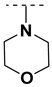	96	90
(**3f**)	8-CF_3_	H	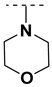	103	95
(**3g**)	8-Cl	Me	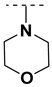	91	87
(**3h**)	6-Cl	H	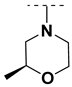	98	96
(**3i**)	7-Cl	H	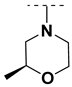	92	99
(**3j**)	8-Cl	H	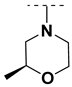	103	97
(**3k**)	8-Me	H	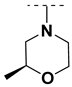	109	107
(**3l**)	8-CN	H	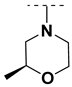	83	97
(**3m**)	8-CF_3_	H	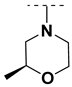	112	109
(**3n**)	8-Cl	Me	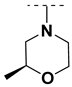	91	90
(**3o**)	6-Cl	H	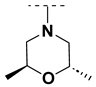	84	96
(**3p**)	7-Cl	H	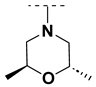	91	99
(**3q**)	8-Cl	H	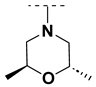	106	85
(**3r**)	8-Me	H	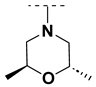	93	81
(**3s**)	8-CN	H	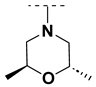	97	95
(**3t**)	8-CF_3_	H	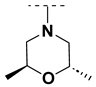	89	89
(**3u**)	8-Cl	Me	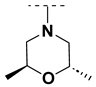	90	80
(**4a**)	6-Cl	H	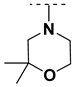	111	113
(**4b**)	7-Cl	H	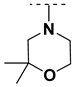	94	91
(**4c**)	8-Cl	H	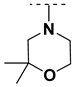	112	103
(**4d**)	8-Me	H	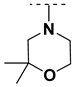	99	97
(**4e**)	8-Cl	Me	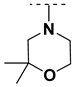	99	87
(**6a**)	6-Cl	H	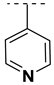	94	92
(**6b**)	7-Cl	H	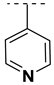	116	114
(**6c**)	8-Cl	H	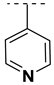	87	90
(**8a**)	6 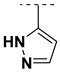	H	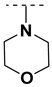	103	100
(**8b**)	7 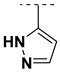	H	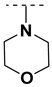	95	97
(**8c**)	8 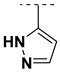	H	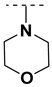	90	89
(**8d**)	6 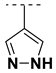	H	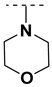	114	94
(**8e**)	7 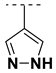	H	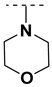	96	99
(**8f**)	8 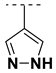	H	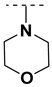	107	106

**Table 2:** The experimental design and treatment groups in the dexamethasone-induced sarcopenia model.

Group No.	Group Description	No. of Animals	Induction Method/Dose	Test Compound Dose	Route of Administration
1	G1 (Control)	7 F	None	0	—
2	G2 (Disease Model Group; DEX)	7 F	DEX 1 mg/kg (14 days)	0	i.p.
3	G3 (**3a**)	7 F	DEX 1 mg/kg (14 days)	15 mg/kg	i.p. (DEX)/as designed
4	G4 (**3c**)	7 F	DEX 1 mg/kg (14 days)	15 mg/kg	i.p. (DEX)/as designed
5	G5 (**6b**)	7 F	DEX 1 mg/kg (14 days)	15 mg/kg	i.p. (DEX)/as designed
6	G6 MTF (Metformin)	7 F	DEX 1 mg/kg (14 days)	15 mg/kg	i.p. (DEX)/as designed
7	G7 (Vehicle)	7 F	DEX 1 mg/kg (14 days)	0	i.p.

**Table 3:** The body weight of the rats in experimental groups.

Group No.	Group Description	Mean Body Weight (As Gram)				
		Day 0	Day 14	Day 28	Day 42	Day 56
1	G1 Control	264.75	272.50	276.75	283.75	284.50
2	G2 (Disease Model Group; DEX)	252.33	240.75	232.50	216.08	265.92
3	G3 (**3a**)	251.25	244.10	238.50	228.00	264.25
4	G4 (**3c**)	252.50	244.75	234.75	230.75	270.75
5	G5 (**6b**)	254.75	246.25	234.75	228.75	269.50
6	G6 MTF (Metformin)	253.42	238.67	239.25	211.17	269.33
7	G7 (Vehicle)	211.33	202.20	196.00	189.33	238.67

## Data Availability

The original contributions presented in this study are included in the article/Supplementary Materials. Further inquiries can be directed to the corresponding author(s).

## Supplementary Materials

The following supporting information can be downloaded at: https://www.mdpi.com/article/10.3390/ph19091442/s1. ^1^H and ^13^C NMR spectra of compounds (Figures S1a,b–S35a,b); mass spectra of compounds (Figures S1c–S35c); computational studies including 2D ligand–protein interaction diagrams and docking results (Figure S1d; Table S1); physicochemical properties of the synthesized ML-167 derivatives (Table S2); representative MRI and histopathological images from the in vivo study (Figures S1e–S2e); and detailed in vivo data, including skeletal muscle mass, biochemical parameters, haematological parameters, and histopathological scores (Tables S3–S8).

## Abbreviations

The following abbreviations are used in this manuscript:

ADME	Absorption, Distribution, Metabolism, and Excretion
C2C12	Mouse C2C12 Myoblast Cell Line
DEX	Dexamethasone
HDL	High-Density Lipoprotein
MRI	Magnetic Resonance Imaging
MS	Mass Spectrometry
MTF	Metformin
MTT	3-(4,5-Dimethylthiazol-2-yl)-2,5-diphenyltetrazolium Bromide
MyH3	Myosin Heavy Chain 3
MyoG	Myogenin
NMR	Nuclear Magnetic Resonance
ROS	Reactive Oxygen Species
