# Supplementary material for: Design, Synthesis, and Biological Evaluation of Novel ML-167 Analogues as Therapeutic Candidates Against Sarcopenia-Induced Muscle Wasting
Source: Pharmaceuticals (Basel). 2026 Sep 11;19(9):1442. doi: 10.3390/ph19091442 (PMC13610714; doi:10.3390/ph19091442)
Supplement: Supplementary file 1 [file pharmaceuticals-19-01442-s001.zip › pharmaceuticals-4497965-supplementary.pdf]

Design, Synthesis, and Biological Evaluation of Novel ML-167 Analogues as Therapeutic Candidates Against Sarcopenia-Induced Muscle Wasting

^{1}H and ^{13}C NMR of compounds (3a–3u), (4a–4e), (6a–6c), and (8a–8f). 6

Figure SM 1a: ^{1}H NMR spectrum of (3a). 6

Figure SM 1b: ^{13}C NMR spectrum of (3a). 6

Figure SM 2a: ^{1}H NMR spectrum of (3b). 7

Figure SM 2b: ^{13}C NMR spectrum of (3b). 7

Figure SM 3a: ^{1}H NMR spectrum of (3c). 8

Figure SM 3b: ^{13}C NMR spectrum of (3c). 8

Figure SM 4a: ^{1}H NMR spectrum of (3d). 9

Figure SM 5a: ^{1}H NMR spectrum of (3e). 10

Figure SM 5b: ^{13}C NMR spectrum of (3e). 10

Figure SM 6b: ^{13}C NMR spectrum of (3f). 11

Figure SM 7a: ^{1}H NMR spectrum of (3g). 12

Figure SM 7b: ^{13}C NMR spectrum of (3g). 12

Figure SM 8a: ^{1}H NMR spectrum of (3h). 13

Figure SM 8b: ^{13}C NMR spectrum of (3h). 13

Figure SM 9a: ^{1}H NMR spectrum of (3i). 14

Figure SM 9b: ^{13}C NMR spectrum of (3i). 14

Figure SM 10a: ^{1}H NMR spectrum of (3j). 15

Figure SM 10b: ^{13}C NMR spectrum of (3j). 15

Figure SM 11b: ^{13}C NMR spectrum of (3k). 16

Figure SM 12b: ^{13}C NMR spectrum of (3l). 17

Figure SM 13a: ^{1}H NMR spectrum of (3m). 18

Figure SM 13b: ^{13}C NMR spectrum of (3m). 18

Figure SM 14a: ^{1}H NMR spectrum of (3n). 19

Figure SM 14b: ^{13}C NMR spectrum of (3n). 19

Figure SM 15a: ^{1}H NMR spectrum of (3o). 20

Figure SM 15b: ^{13}C NMR spectrum of (3o). 20

Figure SM 16a: ^{1}H NMR spectrum of (3p). 21
Figure SM 16b: ^{13}C NMR spectrum of (3p). 21
Figure SM 17a: ^{1}H NMR spectrum of (3q). 22
Figure SM 17b: ^{13}C NMR spectrum of (3q). 22
Figure SM 18a: ^{1}H NMR spectrum of (3r). 23
Figure SM 18b: ^{13}C NMR spectrum of (3r). 23
Figure SM 19a: ^{1}H NMR spectrum of (3s). 24
Figure SM 19b: 1H NMR spectrum of (3s) 24
Figure SM 20a: ^{1}H NMR spectrum of (3t). 25
Figure SM 20b: 1H NMR spectrum of (3t). 25
Figure SM 21a: ^{1}H NMR spectrum of (3u). 26
Figure SM 21b: ^{13}C NMR spectrum of (3u). 26
Figure SM 22a: ^{1}H NMR spectrum of (4a). 27
Figure SM 22b: ^{13}C NMR spectrum of (4a). 27
Figure SM 23a: ^{1}H NMR spectrum of (4b). 28
Figure 23b: ^{13}C NMR spectrum of (4b) 28
Figure SM 24a: ^{1}H NMR spectrum of (4c). 29
Figure SM 24b: ^{13}C NMR spectrum of (4c). 29
Figure SM 25b: ^{13}C NMR spectrum of (4d). 30
Figure SM 26a: ^{1}H NMR spectrum of (4e). 31
Figure SM 26b: ^{13}C NMR spectrum of (4e). 31
Figure SM 27a: ^{1}H NMR spectrum of (6a). 32
Figure SM 27b: ^{13}C NMR spectrum of (6a). 32
Figure SM 28a: ^{1}H NMR spectrum of (6b). 33
Figure SM 28b: ^{13}C NMR spectrum of (6b). 33
Figure SM 29a: ^{1}H NMR spectrum of (6c). 34
Figure SM 29b: ^{13}C NMR spectrum of (6c). 34
Figure SM 30a: ^{1}H NMR spectrum of (8a). 35
Figure SM 30b: ^{13}C NMR spectrum of (8a). 35
Figure SM 31a: ^{1}H NMR spectrum of (8b). 36
Figure SM 31b: ^{13}C NMR spectrum of (8b). 36
Figure SM 32a: ^{1}H NMR spectrum of (8c). 37

Figure SM 32b: ^{13}C NMR spectrum of (8c). 37
Figure SM 33b: ^{13}C NMR spectrum of (8d). 38
Figure SM 34a: ^{1}H NMR spectrum of (8e). 39
Figure SM 34b: ^{13}C NMR spectrum of (8e). 39
Figure SM 35a: ^{1}H NMR spectrum of (8f). 40
Figure SM 35b: ^{13}C NMR spectrum of (8f). 40
Mass spectrum of compounds (3a–3u), **(4a–4e)**, **(6a–6c)**, and **(8a–8f)**. 41
Figure SM 1c: MS spectrum of (3a). 41
Figure SM 2c: MS spectrum of (3b). 41
Figure SM 3c: MS spectrum of (3c). 42
Figure SM 4c: MS spectrum of (3d). 42
Figure SM 5c: MS spectrum of (3e). 43
Figure SM 6c: MS spectrum of (3f). 43
Figure SM 7c: MS spectrum of (3g). 44
Figure SM 8c: MS spectrum of (3h). 44
Figure SM 9c: MS spectrum of (3i). 45
Figure SM 10c: MS spectrum of (3j). 45
Figure SM 11c: MS spectrum of (3k). 46
Figure SM 12c: MS spectrum of (3l). 46
Figure SM 13c: MS spectrum of (3m). 47
Figure SM 14c: MS spectrum of (3n). 47
Figure SM 15c: MS spectrum of (3o). 48
Figure SM 16c: MS spectrum of (3p). 48
Figure SM 17c: MS spectrum of (3q). 49
Figure SM 18c: MS spectrum of (3r). 49
Figure SM 19c: MS spectrum of (3s). 50
Figure SM 20c: MS spectrum of (3t). 50
Figure SM 21c: MS spectrum of (3u). 51
Figure SM 22c: MS spectrum of (4a). 51
Figure SM 23c: MS spectrum of (4b). 52
Figure SM 24c: MS spectrum of (4c). 52
Figure SM 25c: MS spectrum of (4d). 53

Figure SM 26c: MS spectrum of (4e). 53
Figure SM 27c: MS spectrum of (6a). 54
Figure SM 28c: MS spectrum of (6b). 54
Figure SM 29c: MS spectrum of (6c). 55
Figure SM 30c: MS spectrum of (8a). 55
Figure SM 31c: MS spectrum of (8b). 56
Figure SM 32c: MS spectrum of (8c). 56
Figure SM 33c: MS spectrum of (8d). 57
Figure SM 34c: MS spectrum of (8e). 57
Figure SM 35c: MS spectrum of (8f). 58

Computational Data: 59

Figure SM 1d: 2D representation of the binding interactions between compounds 3a, 6b, 4c, 8d, 3c, and 3m of the ML-167 derivatives with Cyc1 59

Table SM 1: Details of biological activity, docking scores, ligand efficiency, and key hydrophobic and hydrophilic interactions of ML-167 derivatives. 60

Physicochemical properties : 61

Table SM 2. Physicochemical parameters of the tested synthesized ML-167 derivatives. 61

***In vivo* Data:** 63

Figure SM 1e. Representative MRI images of skeletal muscles in experimental groups. Magnetic resonance imaging (MRI) of the quadriceps femoris, gluteus maximus, and longissimus dorsi muscles in the control, dexamethasone-induced sarcopenia model, G4 (3c), and metformin (MTF) groups. Highlighted regions indicate the analyzed muscle areas used for volumetric and signal intensity assessments, illustrating treatment-related changes in muscle morphology. 63

Figure SM 2e. Representative histopathological images of skeletal muscle tissues in the experimental groups. Sections from the Control, Sarcopenia model, G3 (3a), G4 (3c), G5 (6b), Metformin (MTF), and Vehicle groups demonstrate treatment-related atrophy and morphological changes. 64

Table SM 3. The Right and left skeletal muscle mass (mean ± SD) of the Gluteus Maximus, Quadriceps Femoris, and Longissimus Dorsi in experimental groups. 65

Table SM 4. The average skeletal muscle mass (mean ± SD) of Gluteus Maximus, Quadriceps Femoris, and Longissimus Dorsi in experimental groups. 65

Table SM 5. The total skeletal muscle mass (mean ± SD) in experimental groups. 66

Table SM 6. Combined biochemical parameters in experimental groups (Mean ± SD). 66
Table SM 7. Haematological parameters in experimental groups (Ort ± SD). 67
Table SM 8. Histopathological Scores of Skeletal Muscle (Ort ± SD). 68

^{1}H and ^{13}C NMR of compounds (3a–3u), (4a–4e), (6a–6c), and (8a–8f).

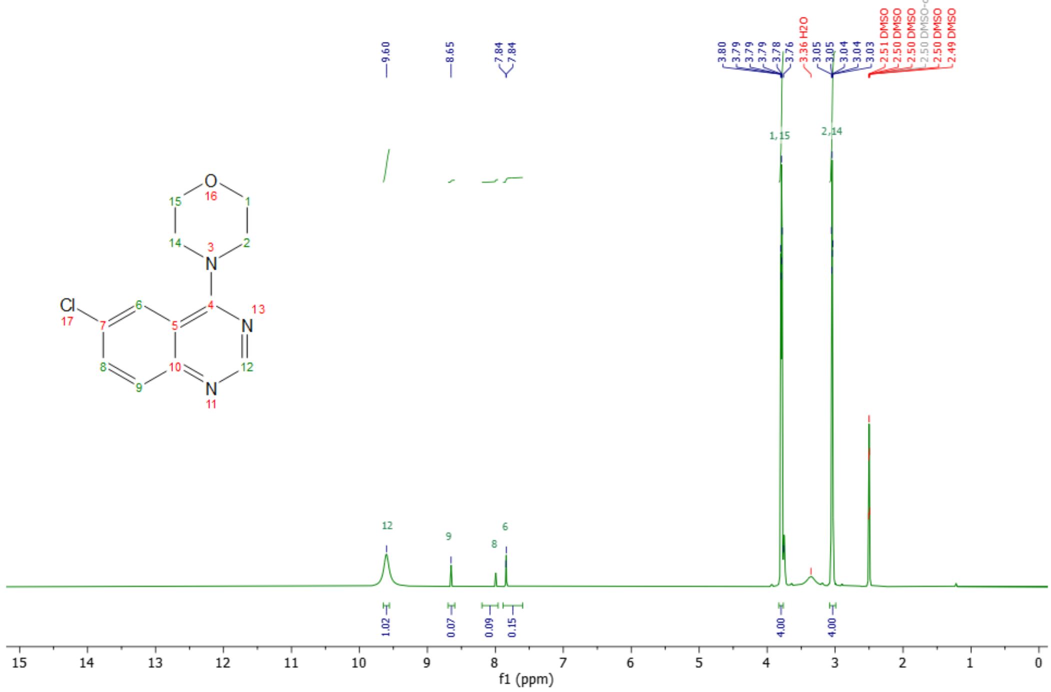

Figure S1a: ^{1}H NMR spectrum of (3a).

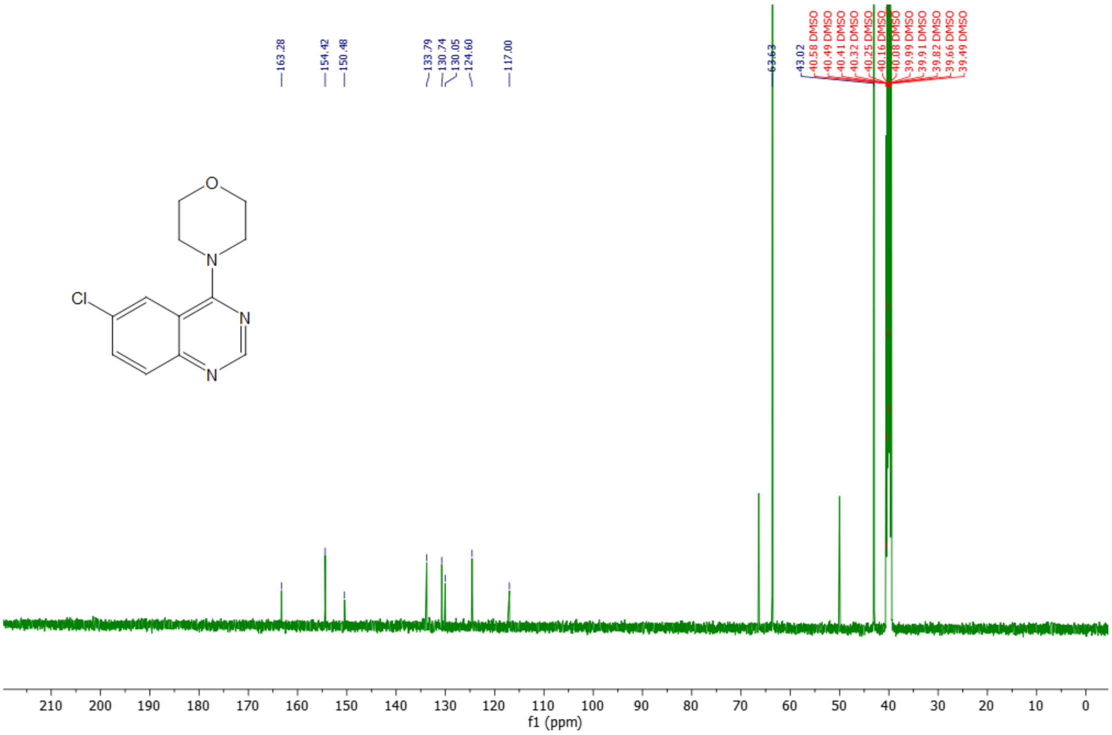

Figure S1b: ^{13}C NMR spectrum of (3a).

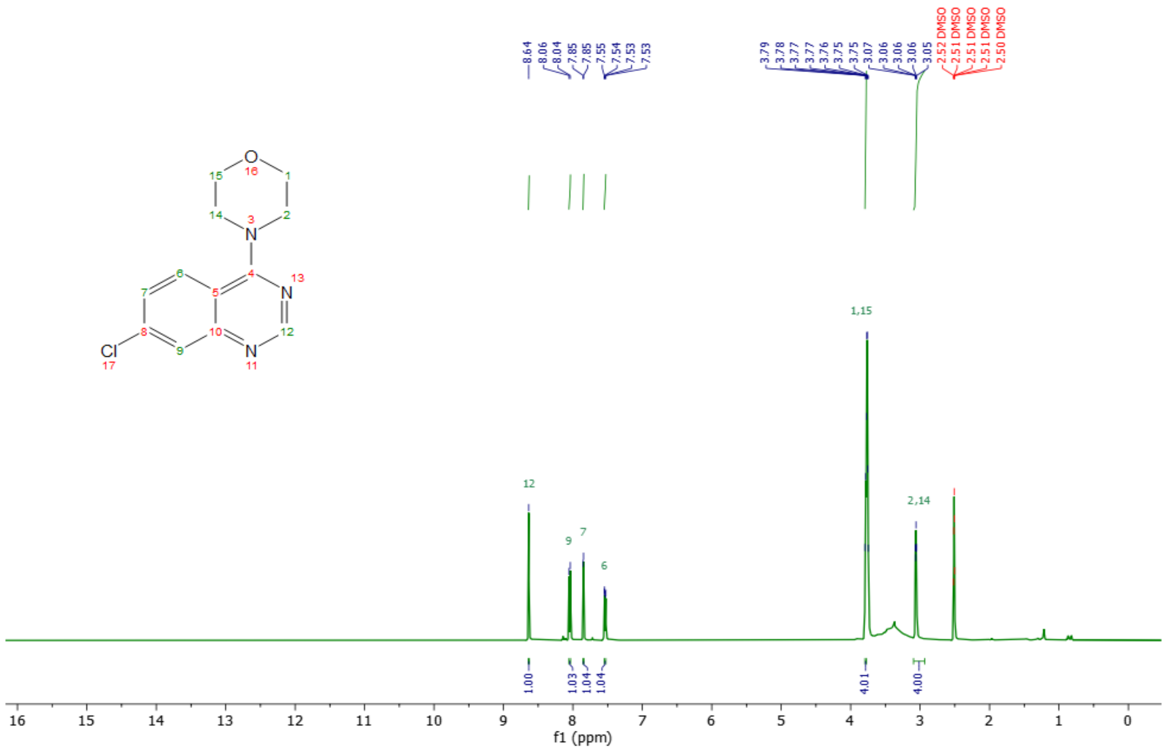

Figure S2a: ^{1}H NMR spectrum of (3b).

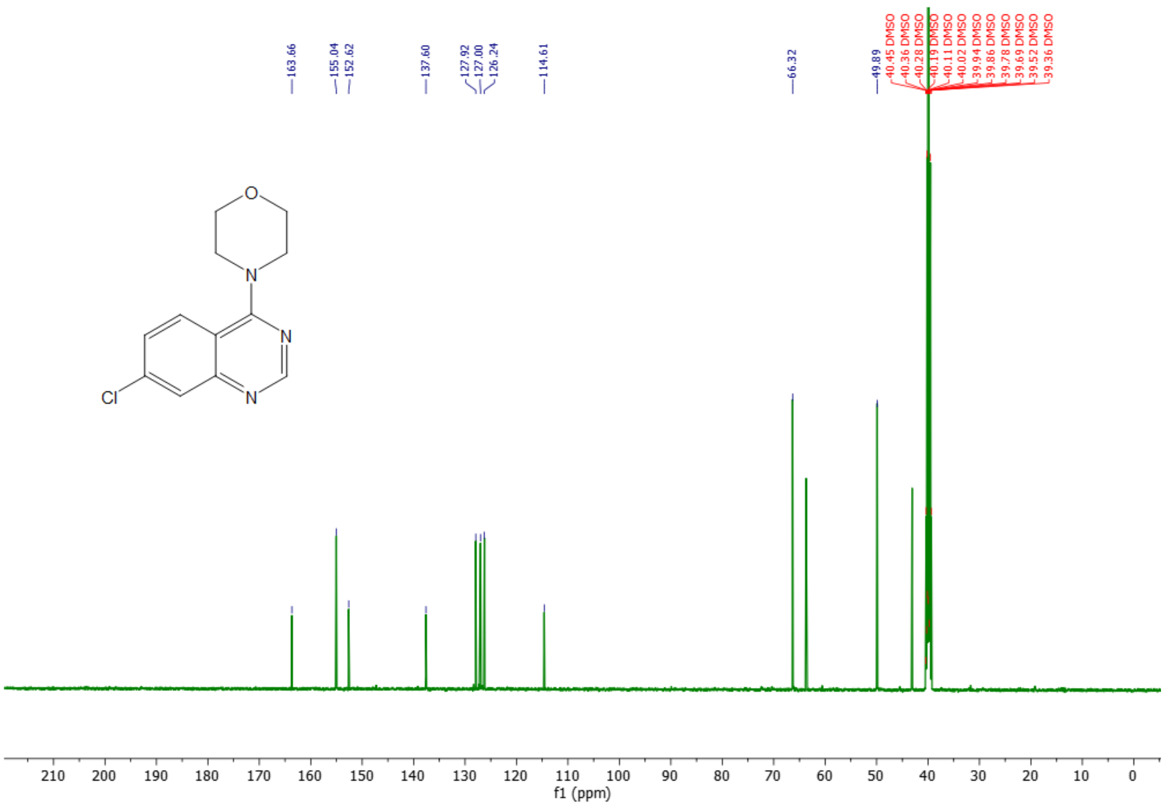

Figure S2b: ^{13}C NMR spectrum of (3b).

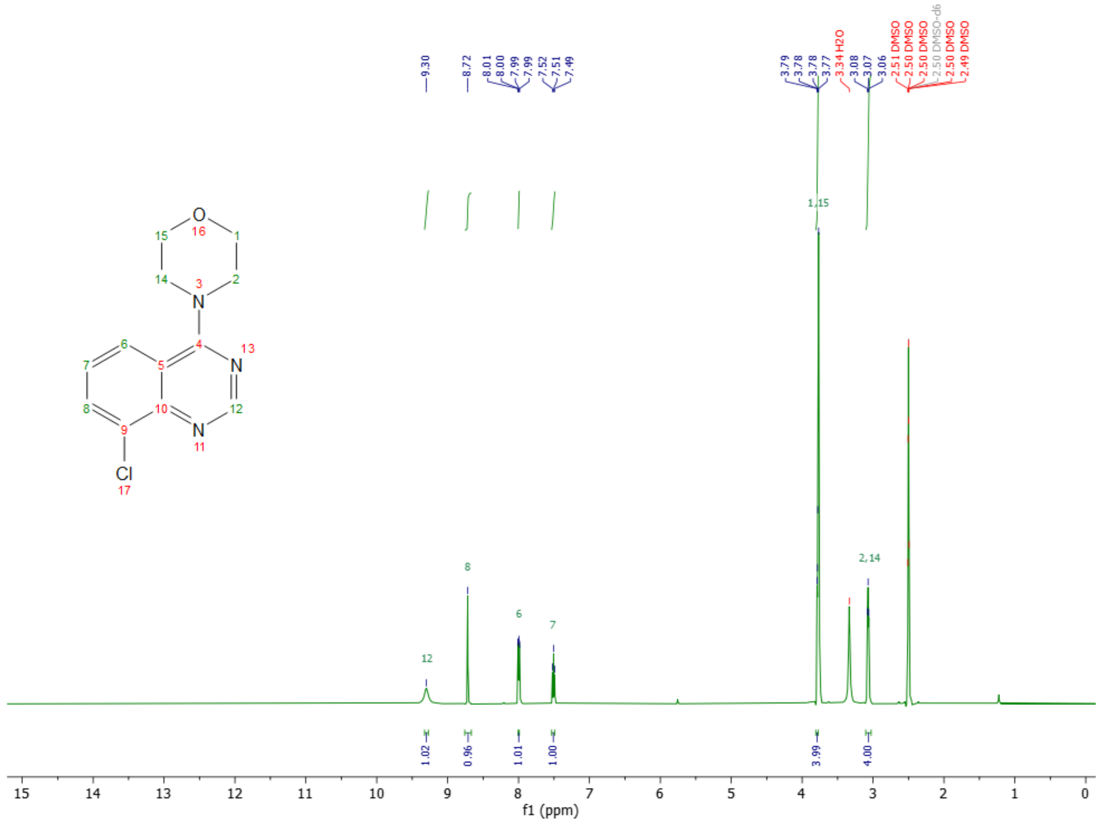

Figure S3a: ^{1}H NMR spectrum of (3c).

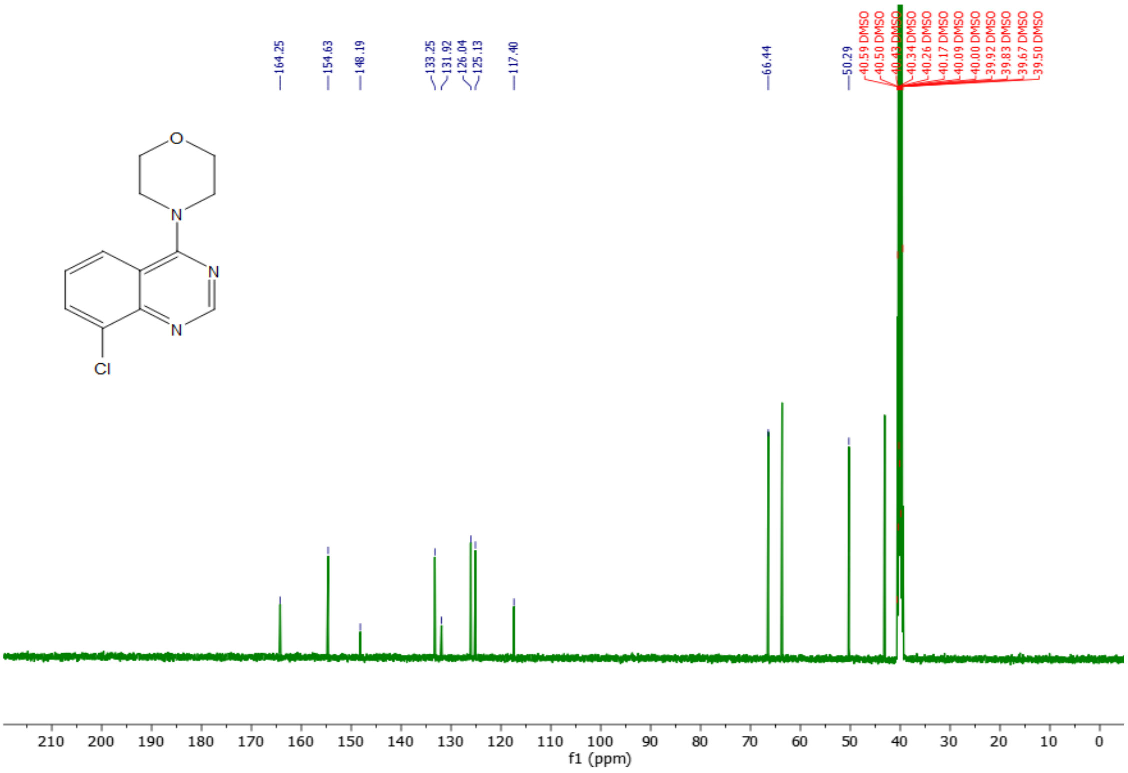

Figure S3b: ^{13}C NMR spectrum of (3c).

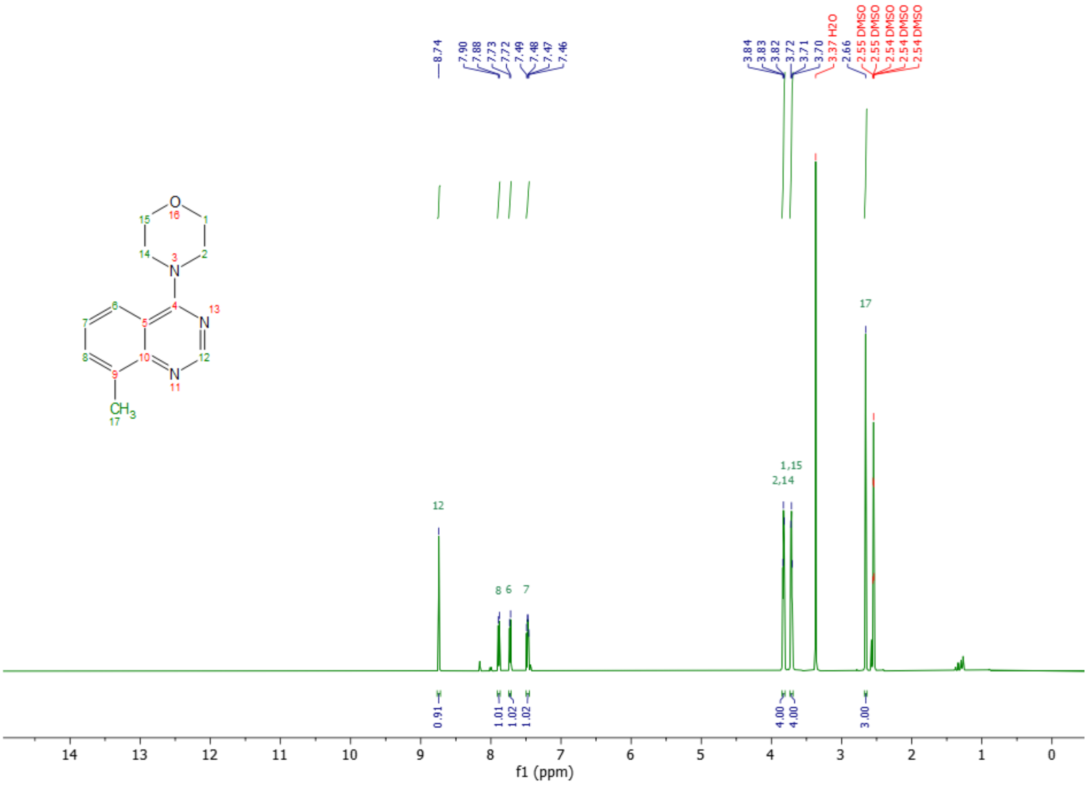

Figure S4a: ^{1}H NMR spectrum of (3d).

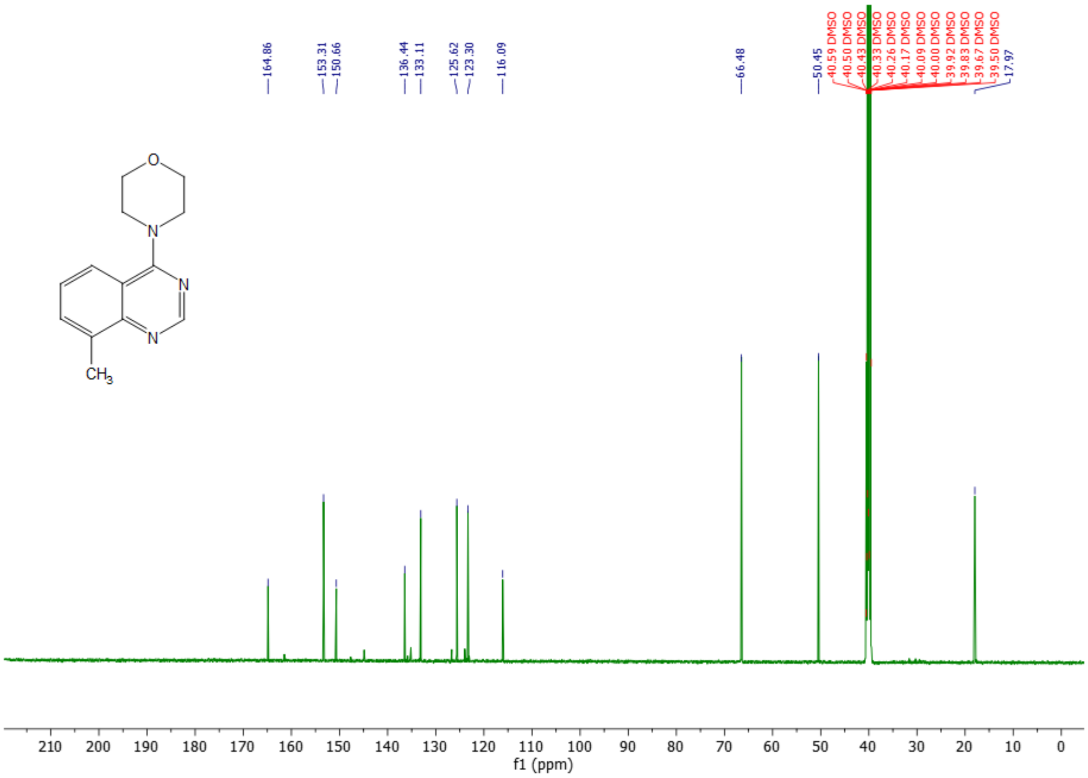

Figure S4b: 13C NMR spectrum of (3d).

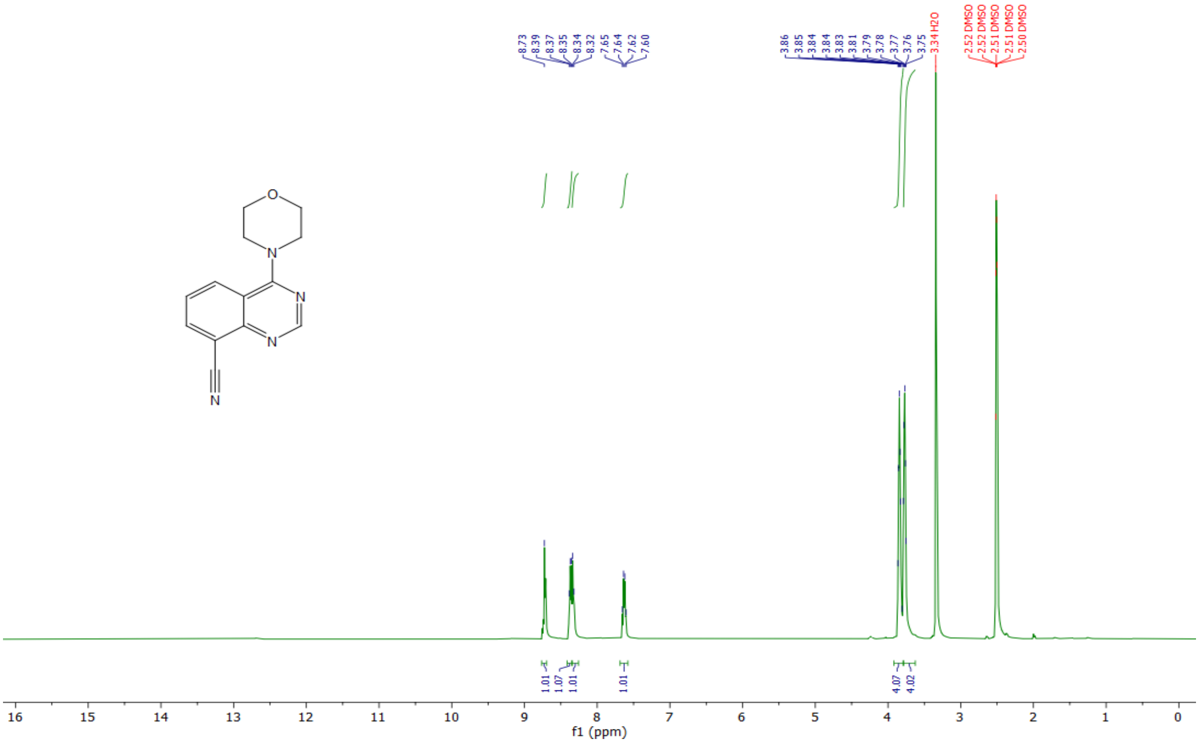

Figure S5a: ^{1}H NMR spectrum of (3e).

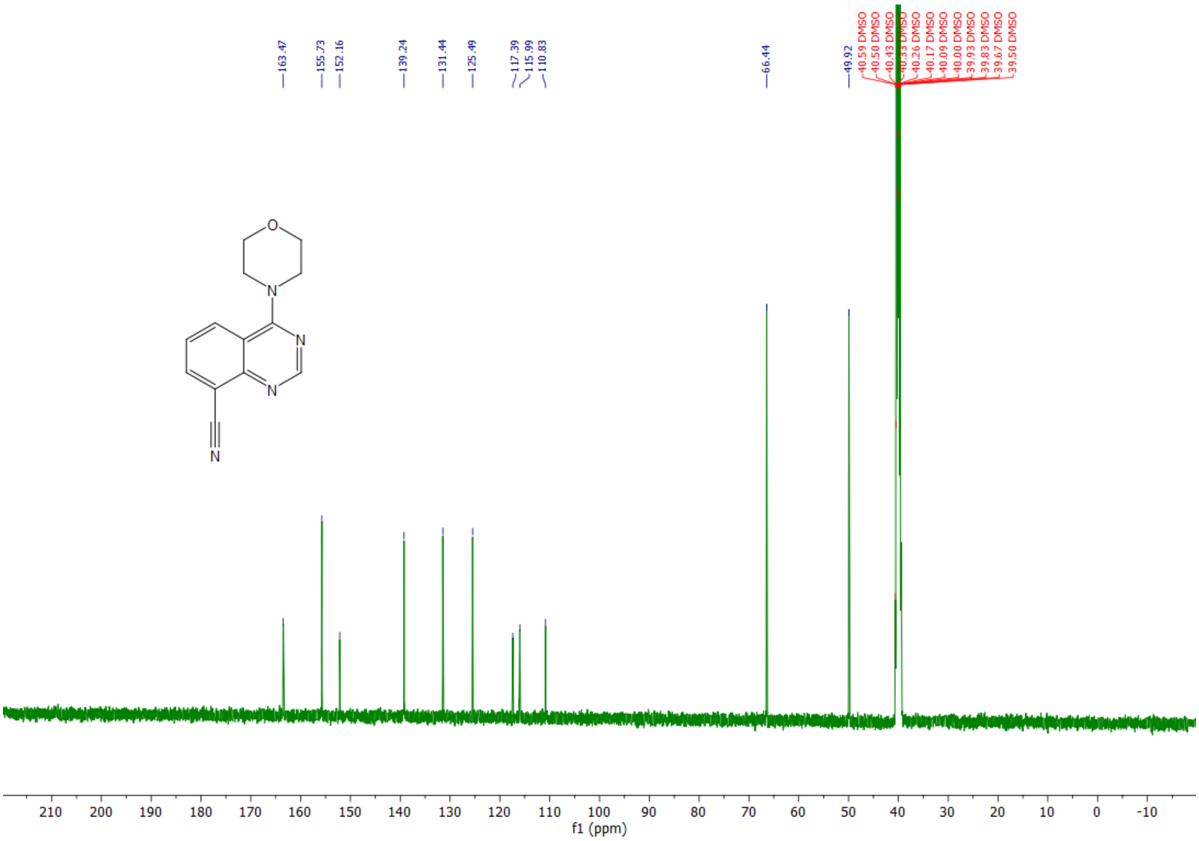

Figure S5b: ^{13}C NMR spectrum of (3e).

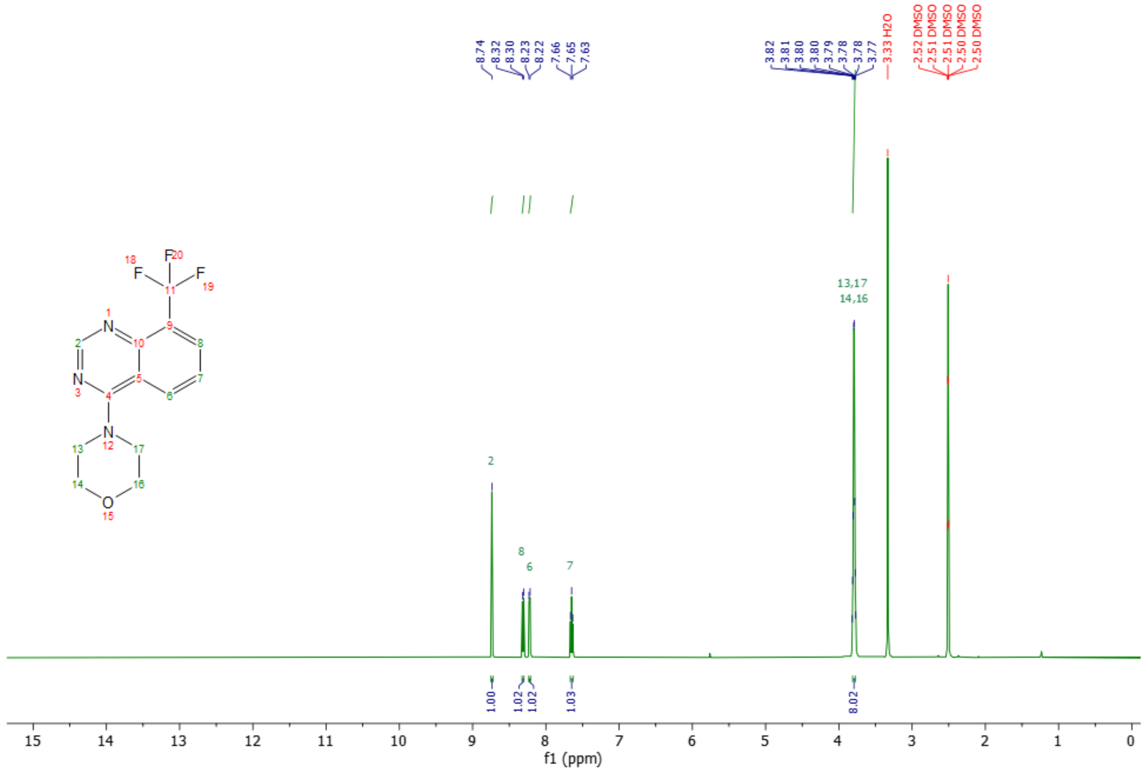

Figure S6a: 1H NMR spectrum of (3f).

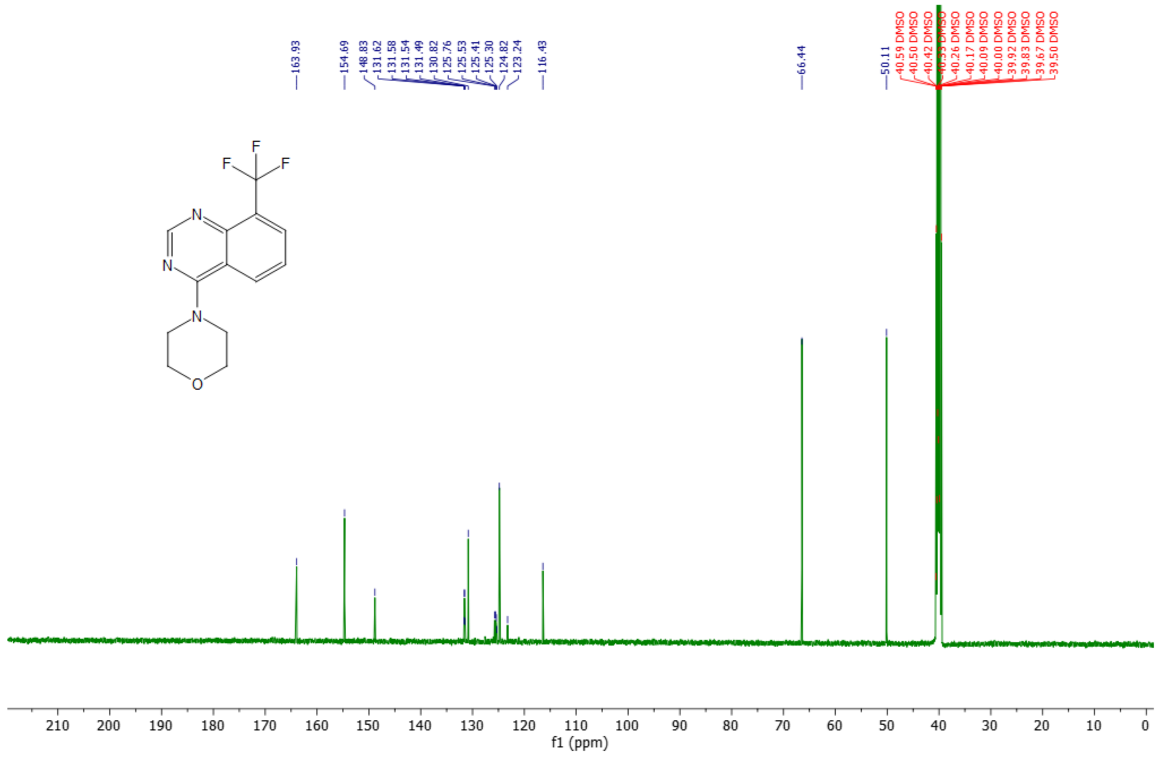

Figure S6b: ^{13}C NMR spectrum of (3f).

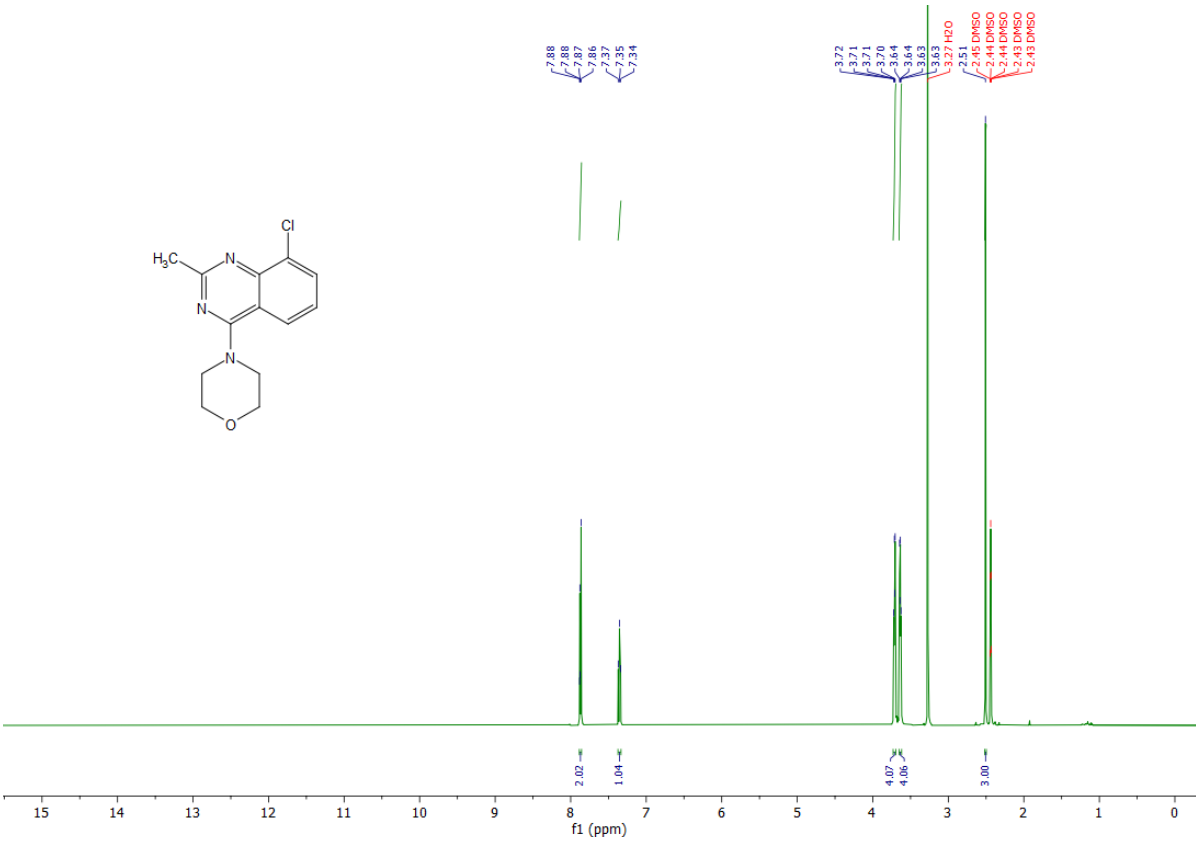

Figure S7a: ^{1}H NMR spectrum of (3g).

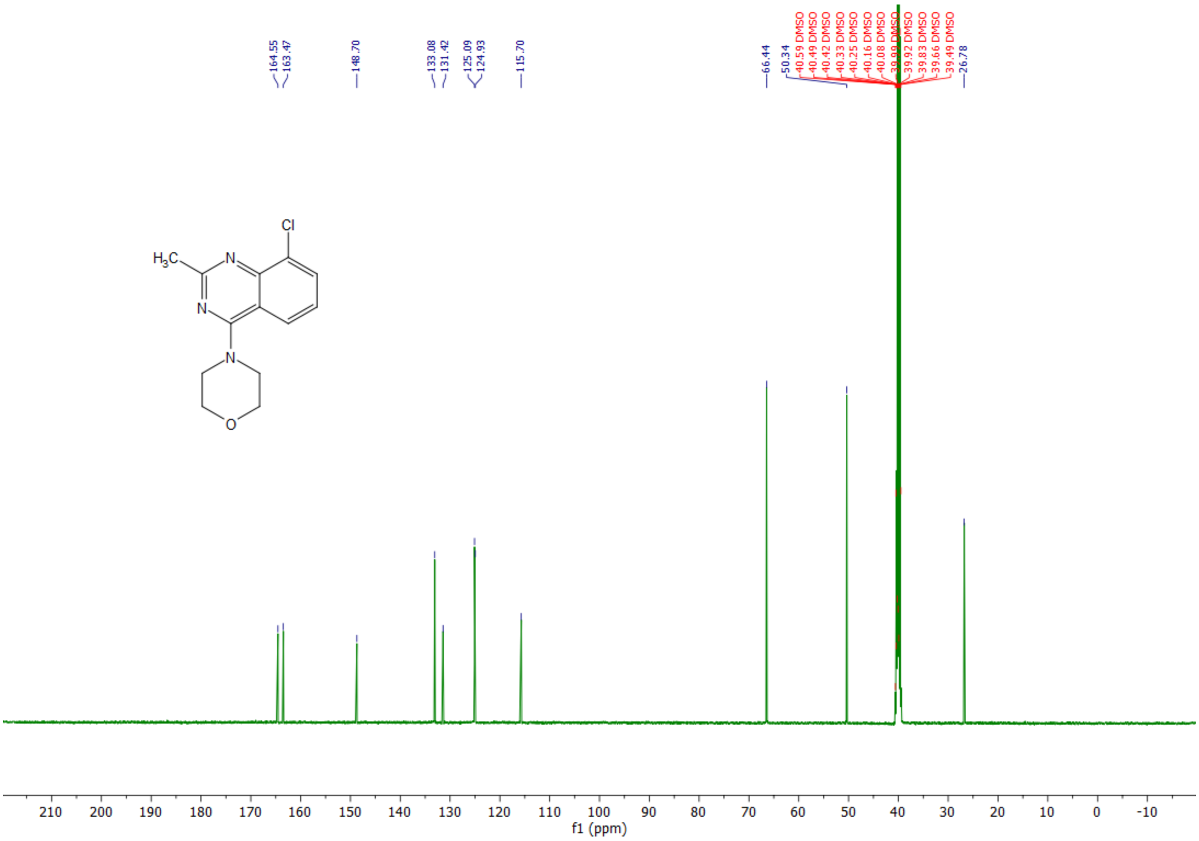

Figure S7b: ^{13}C NMR spectrum of (3g).

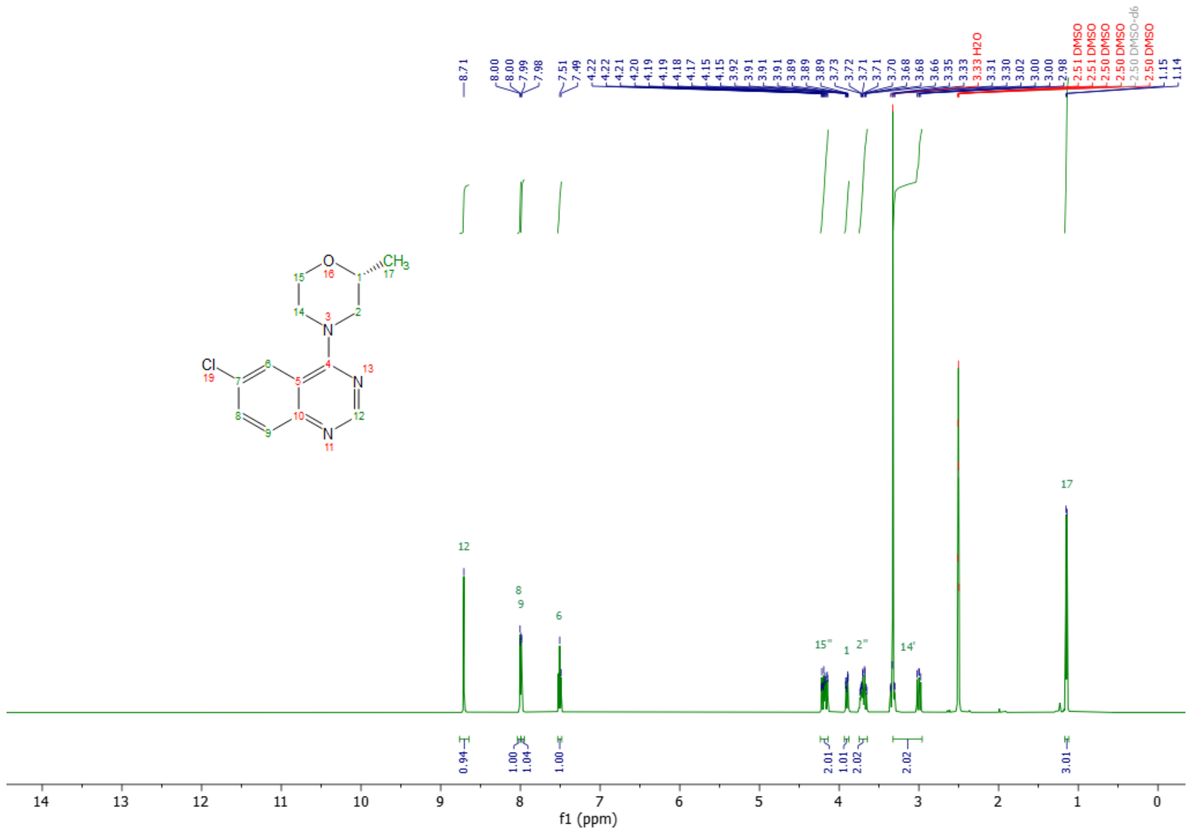

Figure S8a: ^{1}H NMR spectrum of (3h).

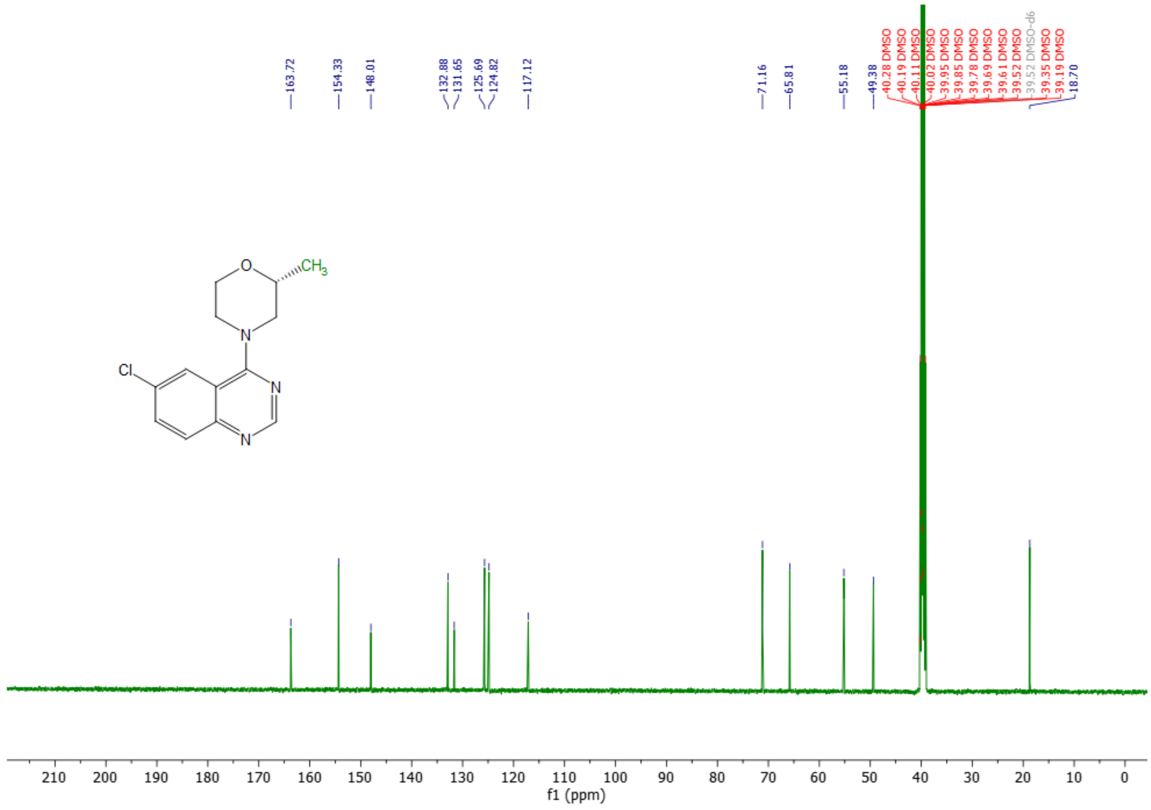

Figure S8b: ^{13}C NMR spectrum of (3h).

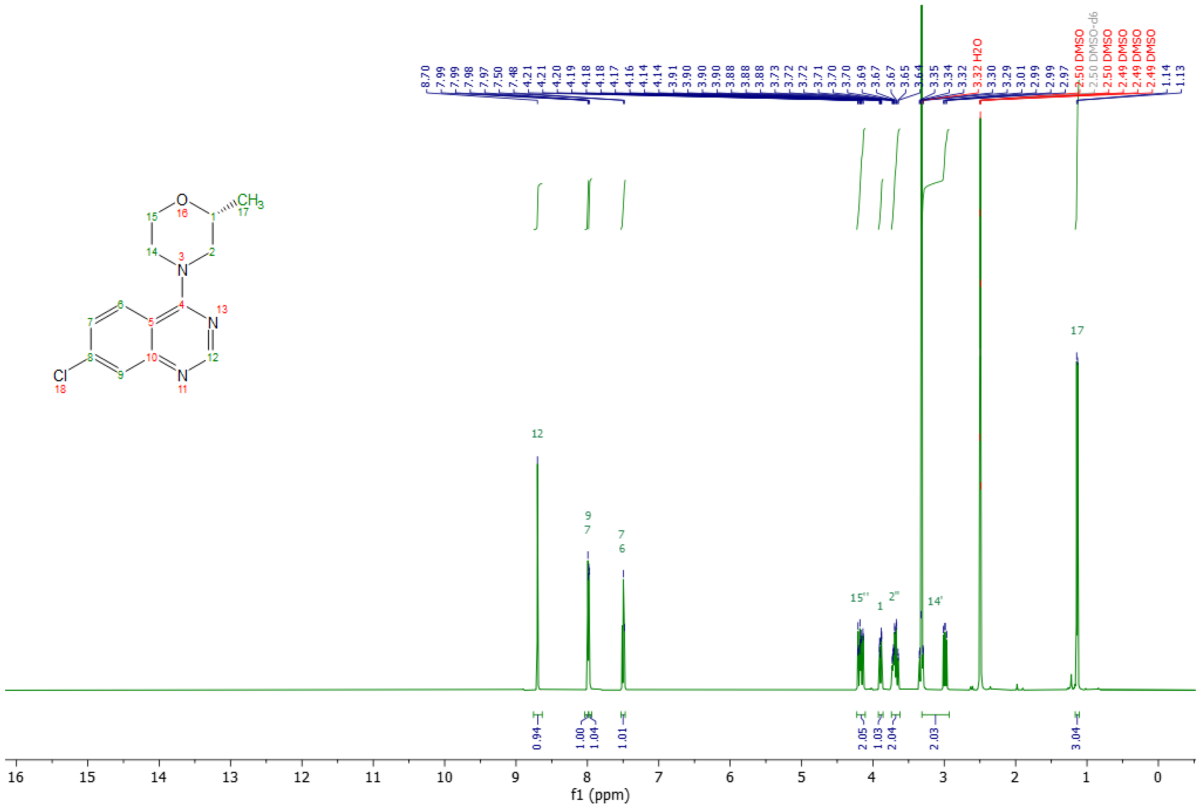

Figure S9a: ^{1}H NMR spectrum of (3i).

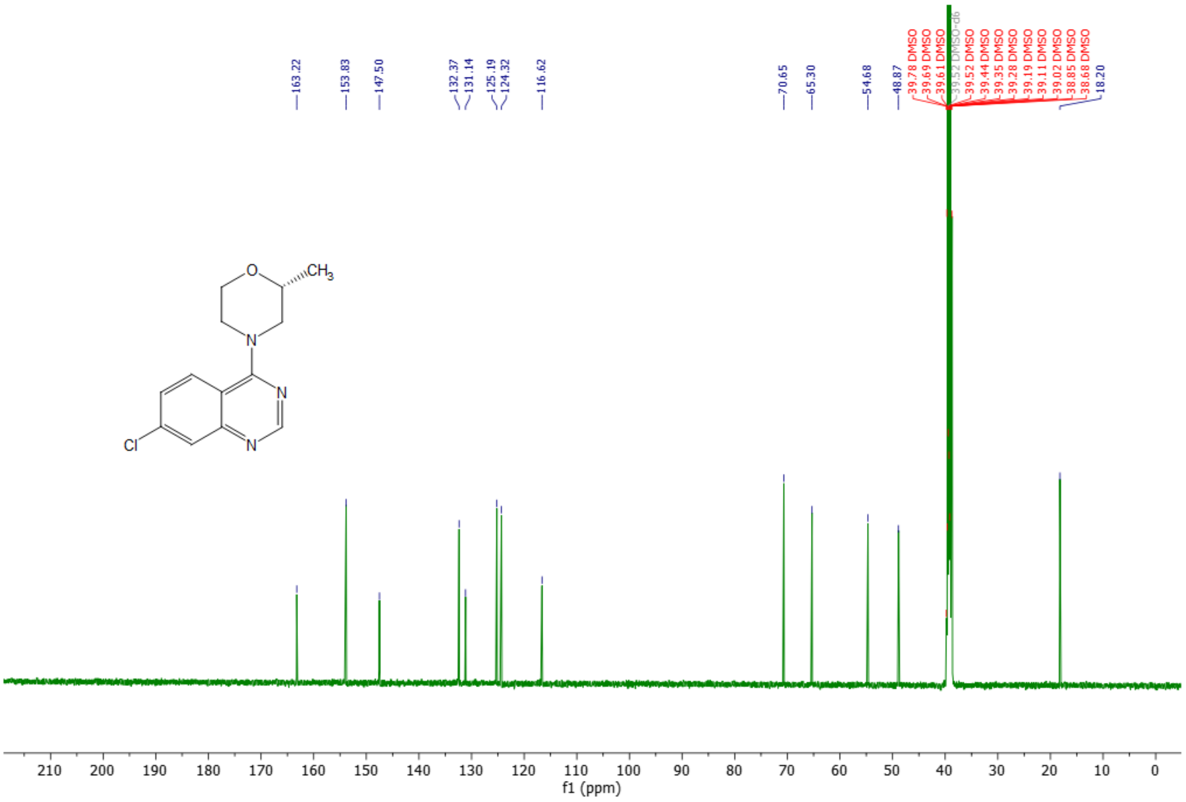

Figure S9b: ^{13}C NMR spectrum of (3i).

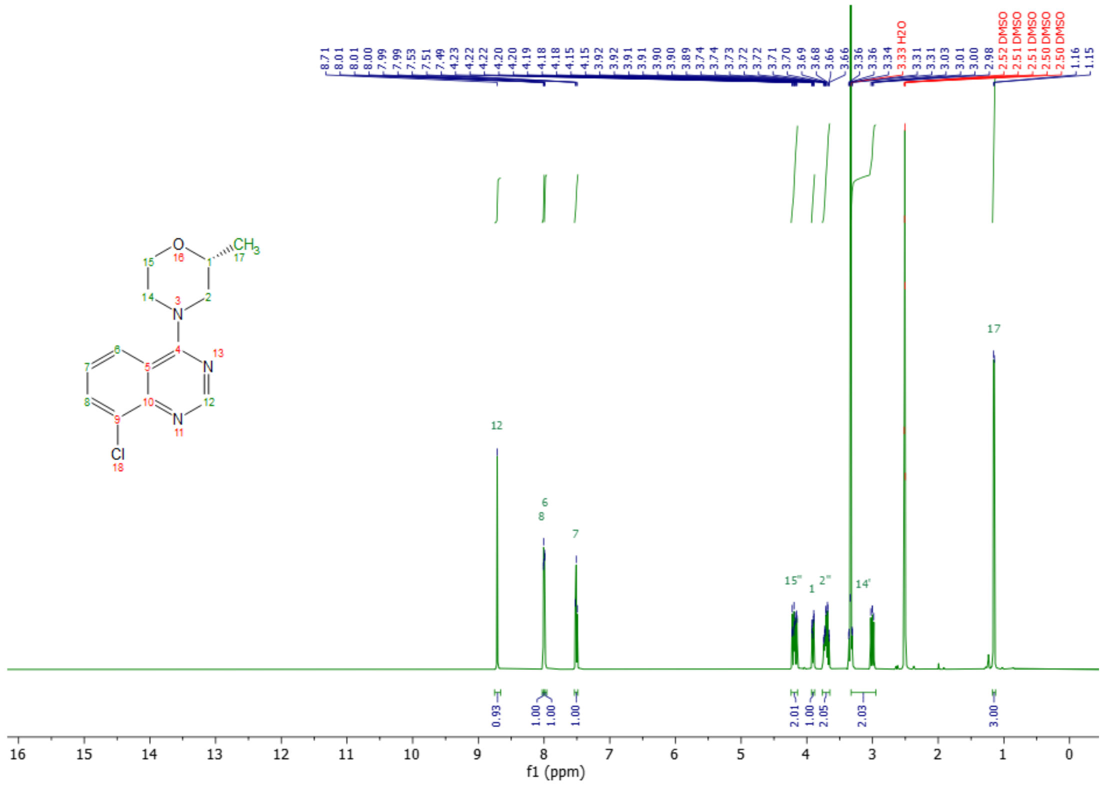

Figure S10a: ^{1}H NMR spectrum of (3j).

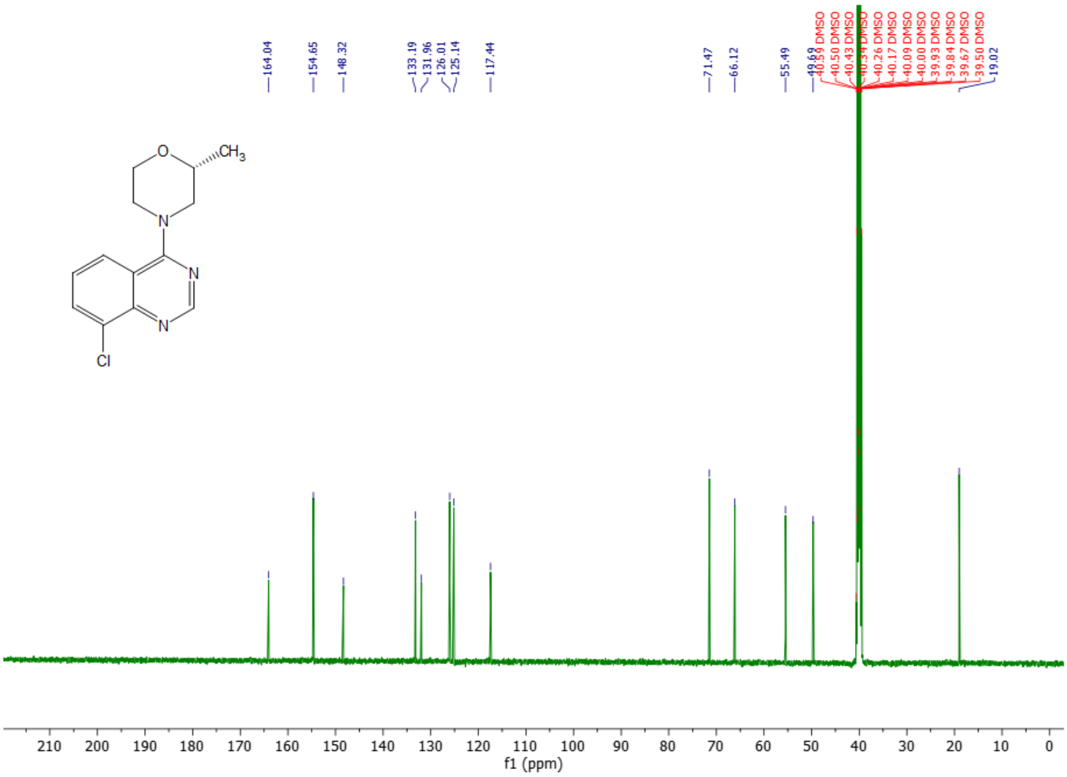

Figure S10b: ^{13}C NMR spectrum of (3j).

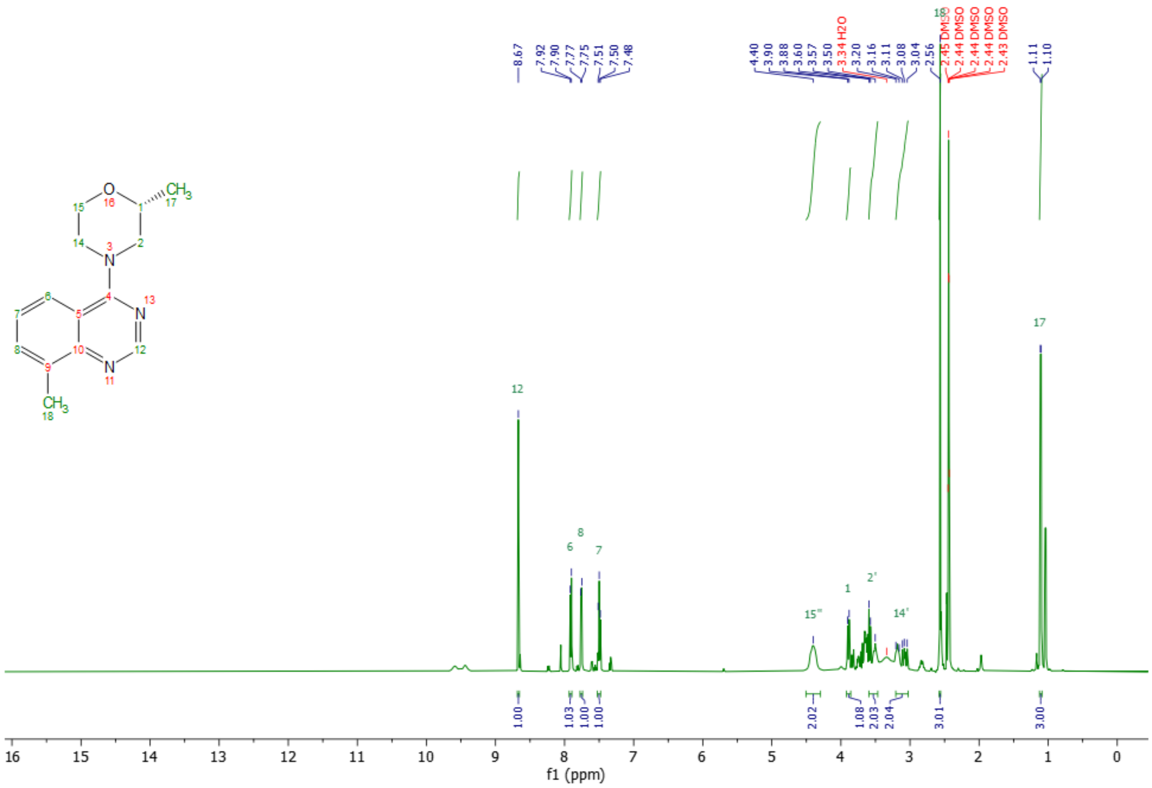

Figure S11a: 1H NMR spectrum of (3k).

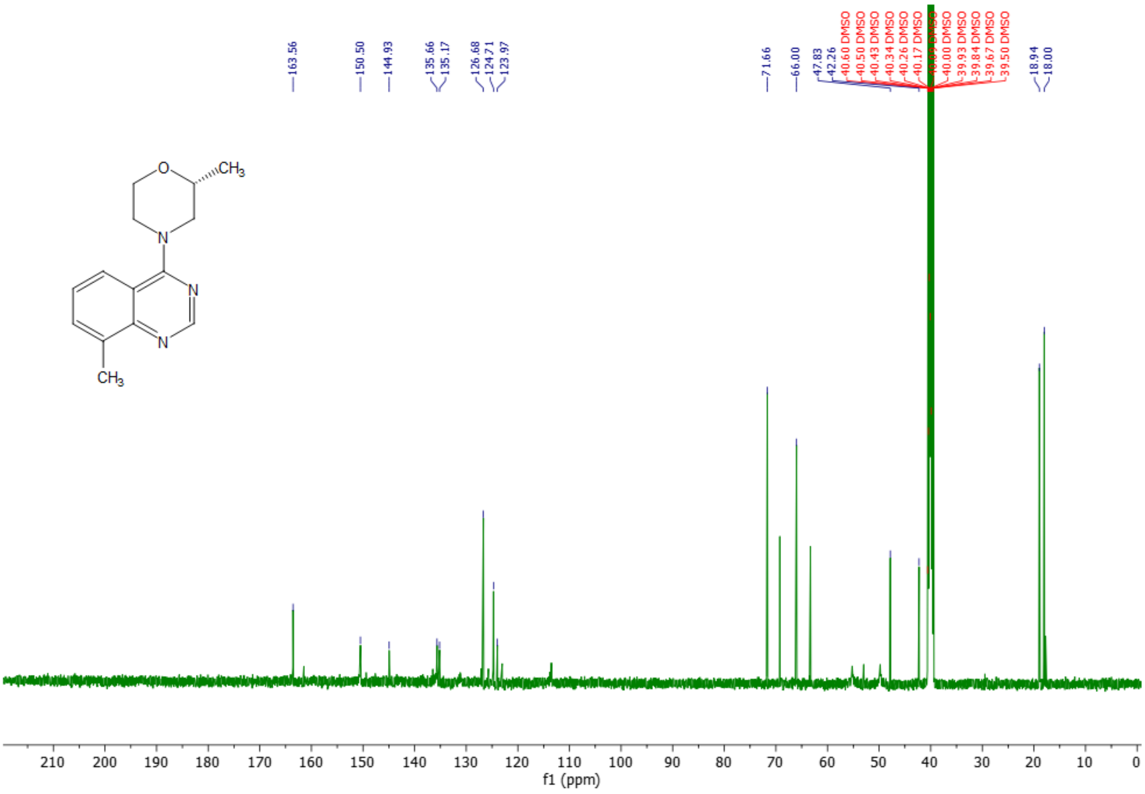

Figure S11b: ^{13}C NMR spectrum of (3k).

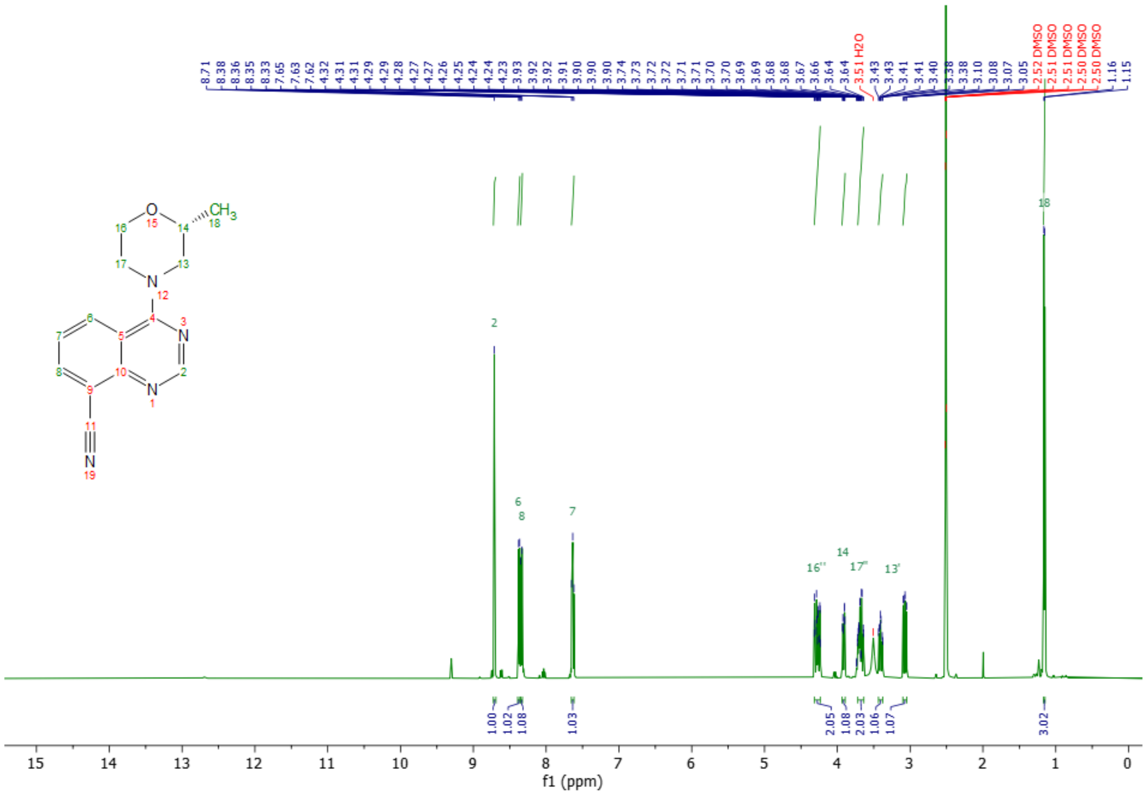

Figure S12a: 1H NMR spectrum of (3l).

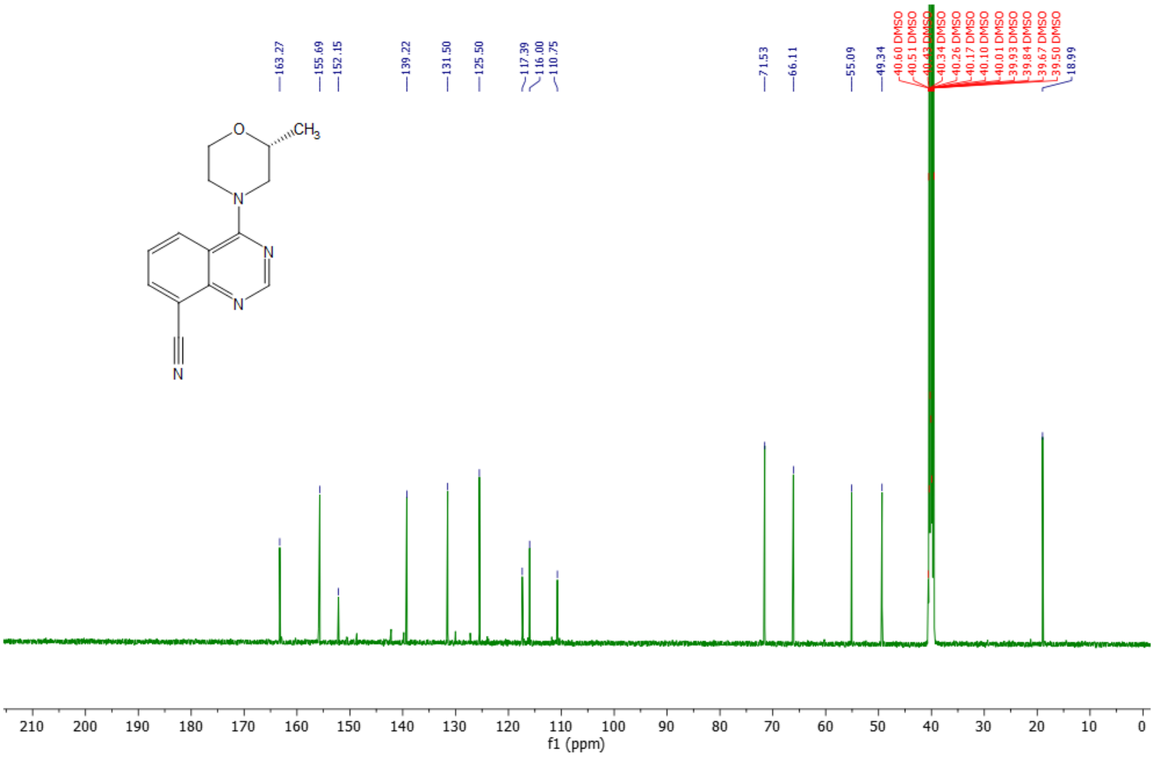

Figure S12b: ^{13}C NMR spectrum of (3l).

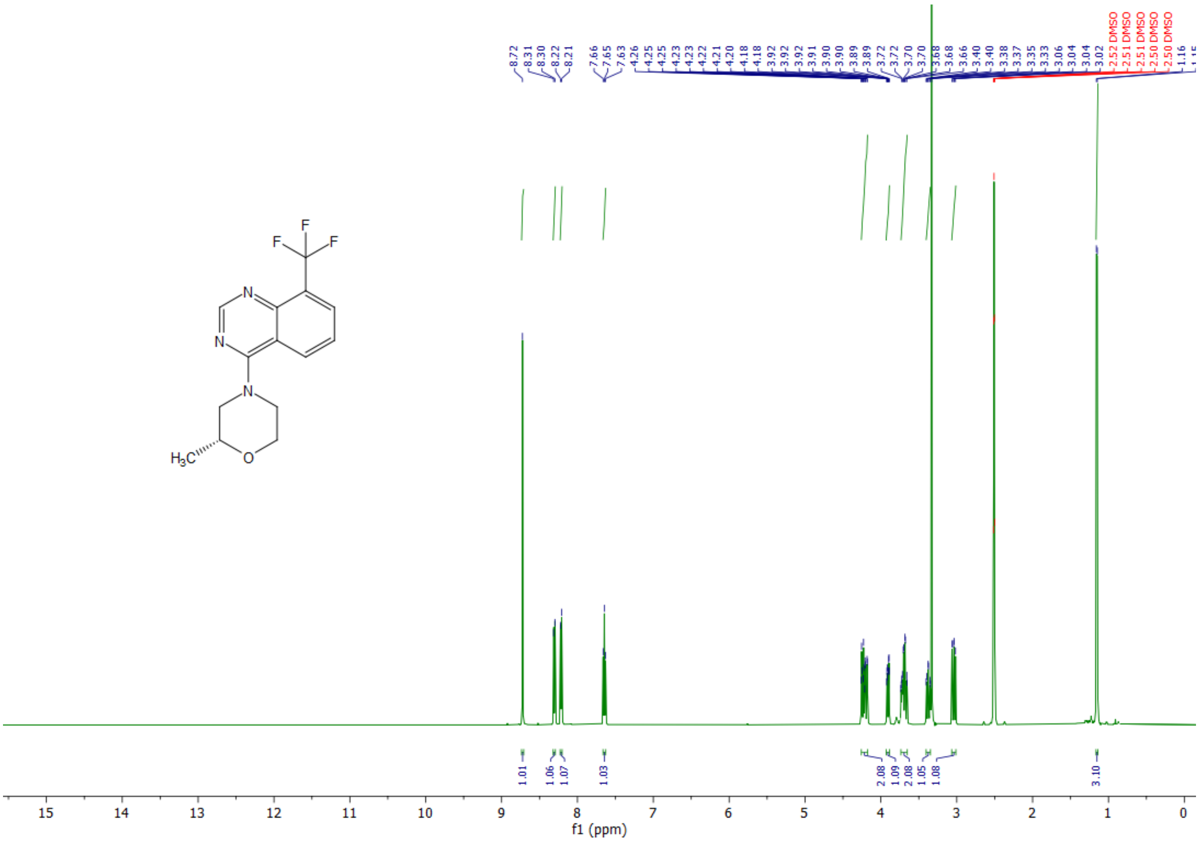

Figure S13a: ^{1}H NMR spectrum of (3m).

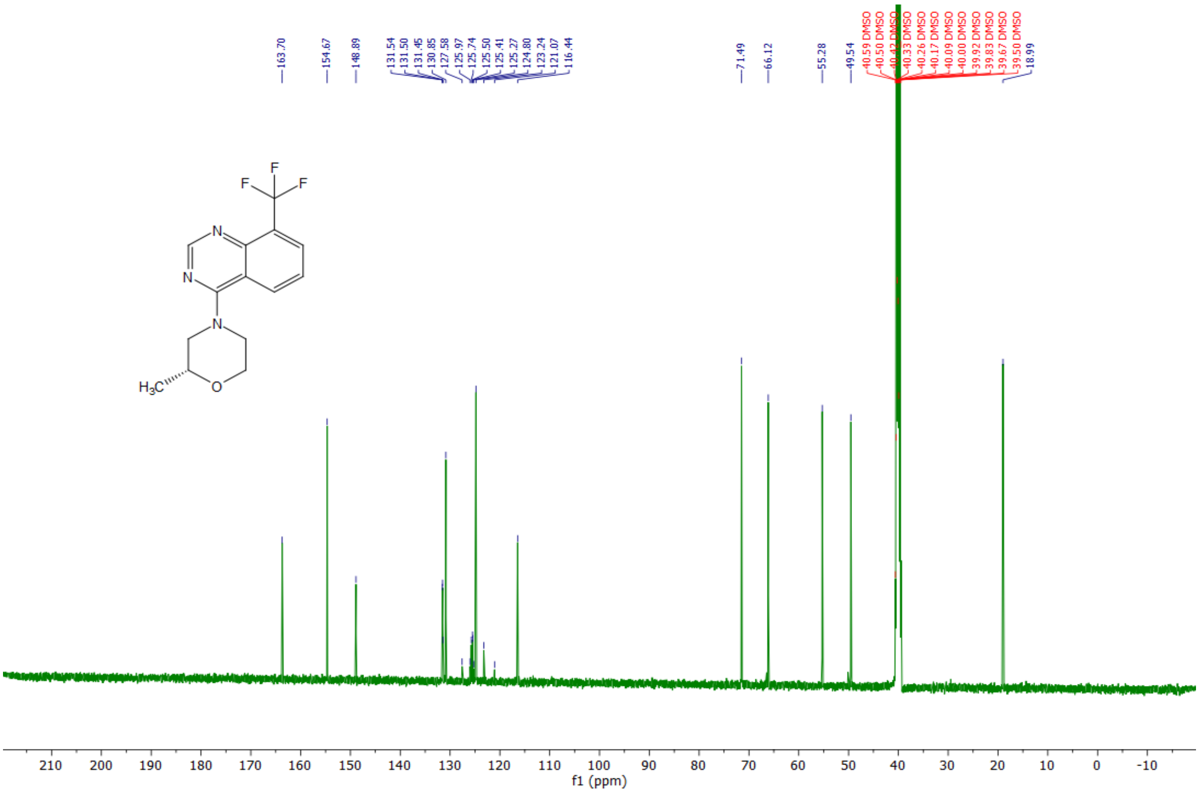

Figure S13b: ^{13}C NMR spectrum of (3m).

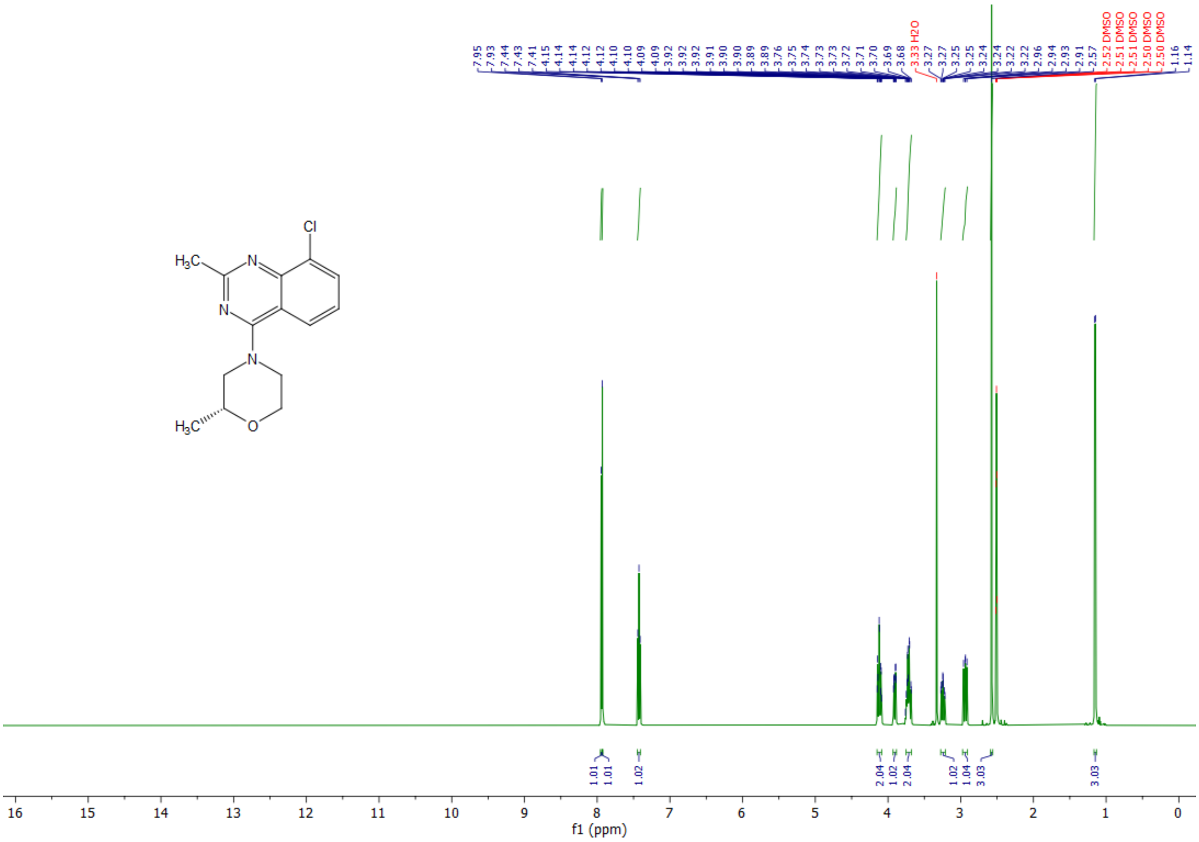

Figure S14a: ^{1}H NMR spectrum of (3n).

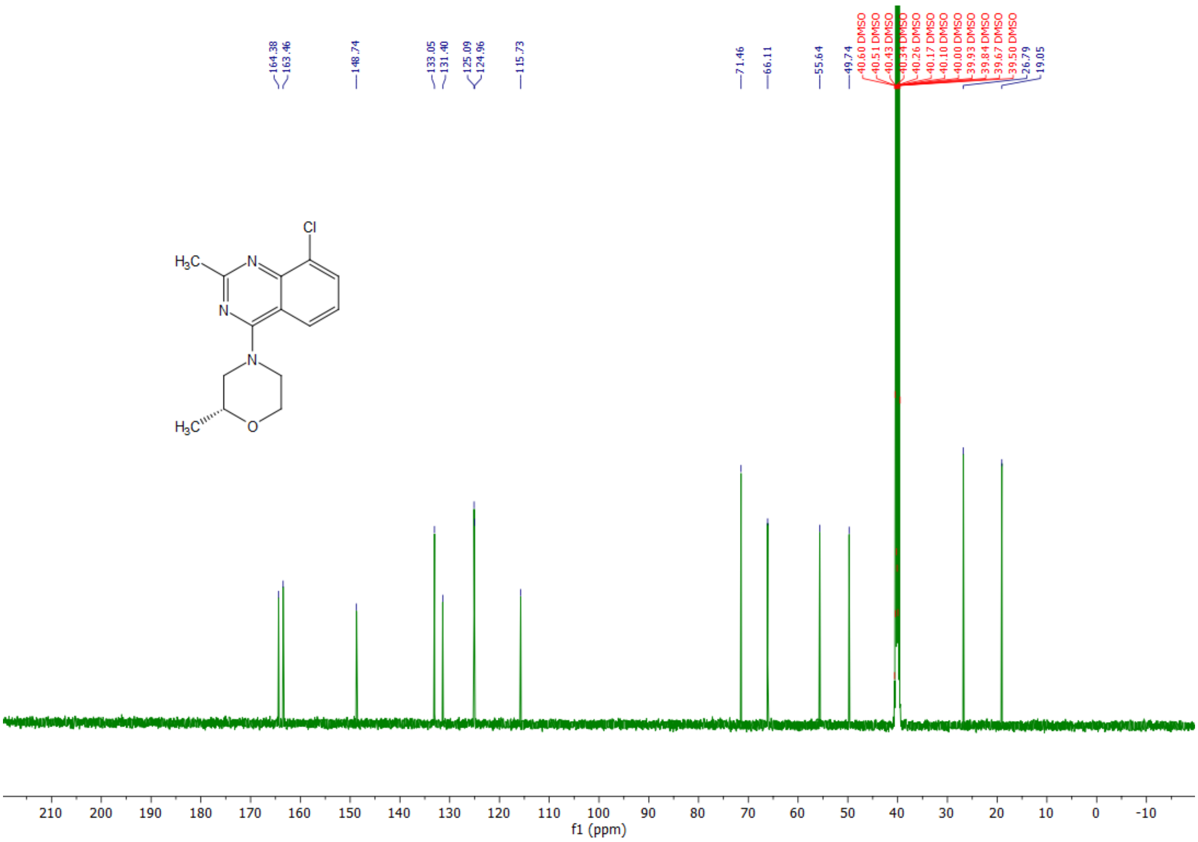

Figure S14b: ^{13}C NMR spectrum of (3n).

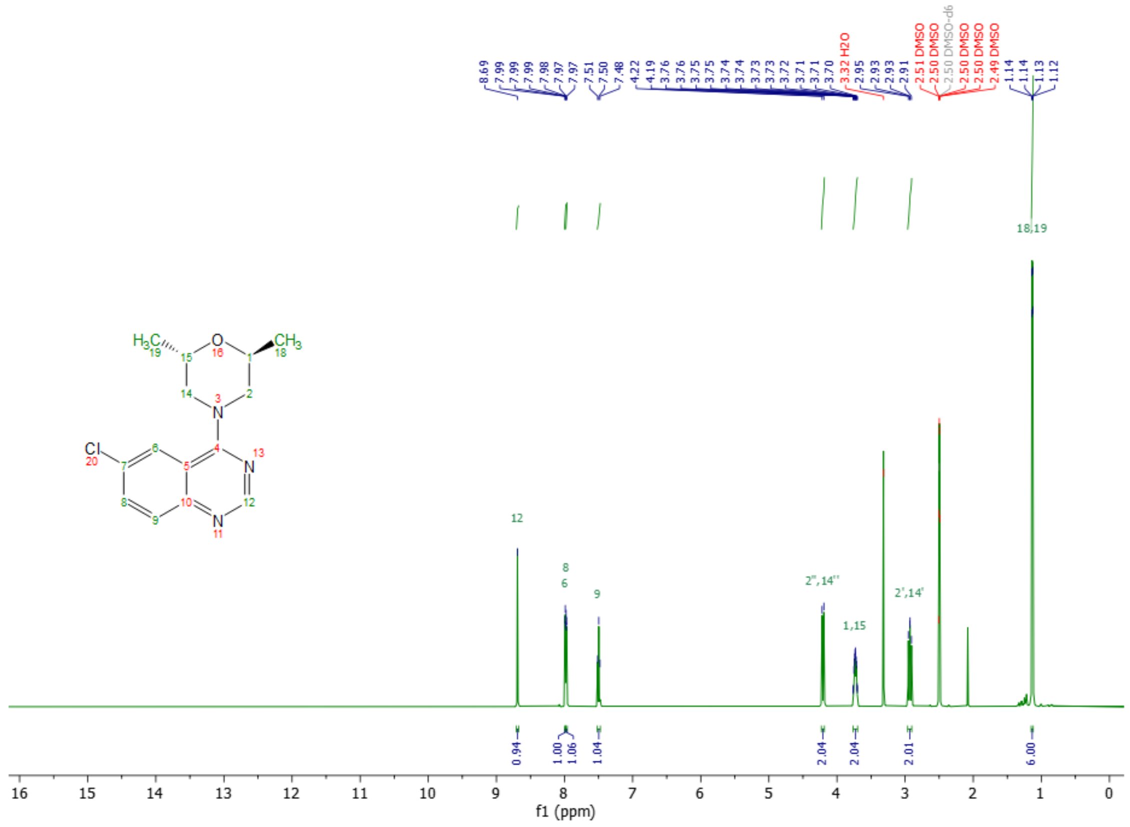

Figure S15a: ^{1}H NMR spectrum of (3o).

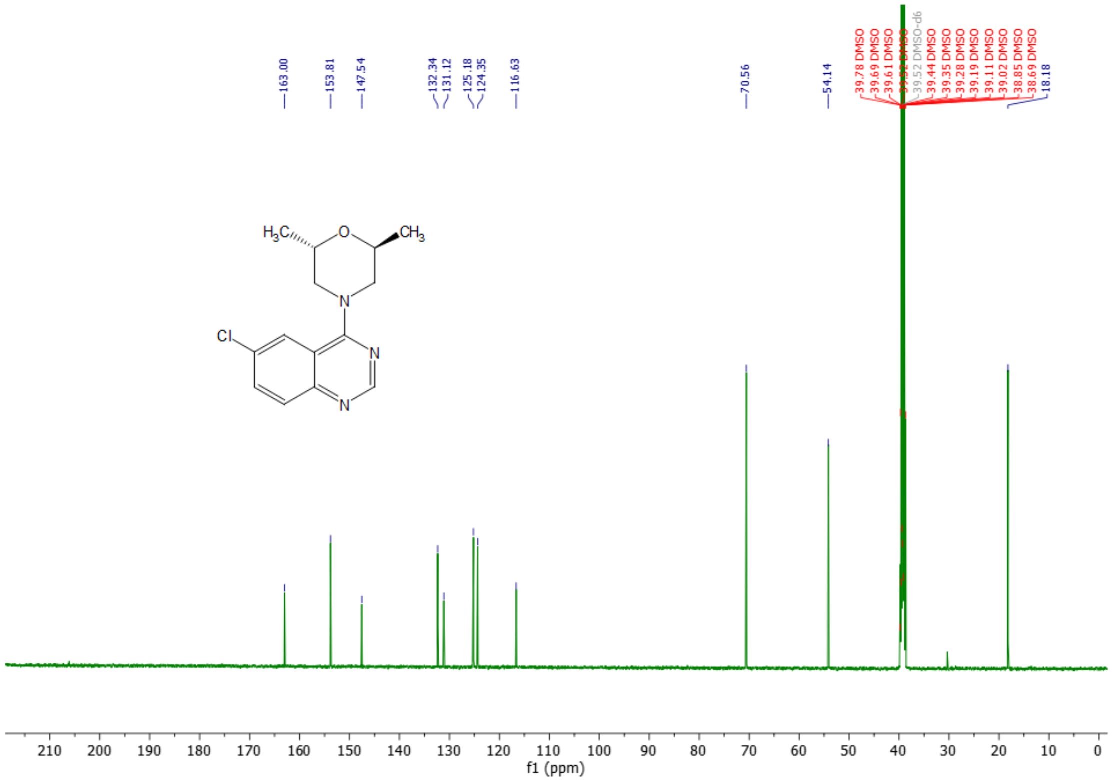

Figure S15b: ^{13}C NMR spectrum of (3o).

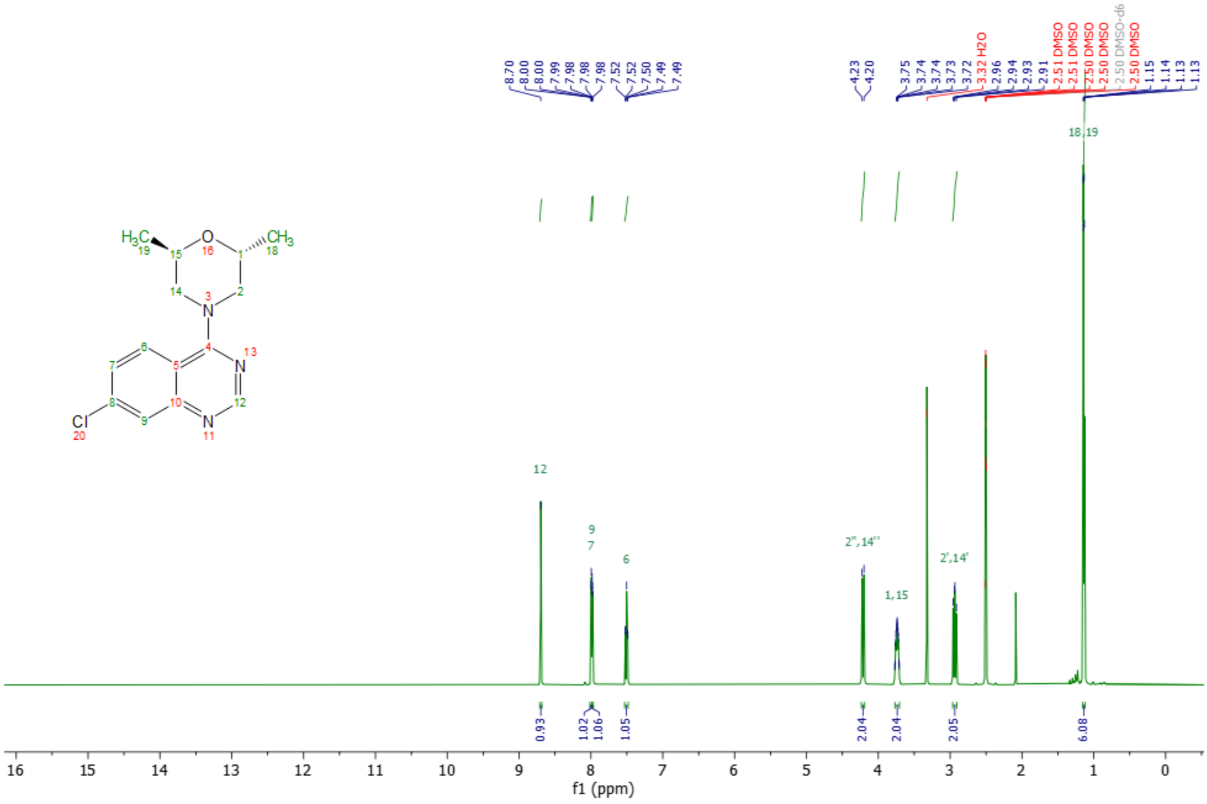

Figure S16a: ^{1}H NMR spectrum of (3p).

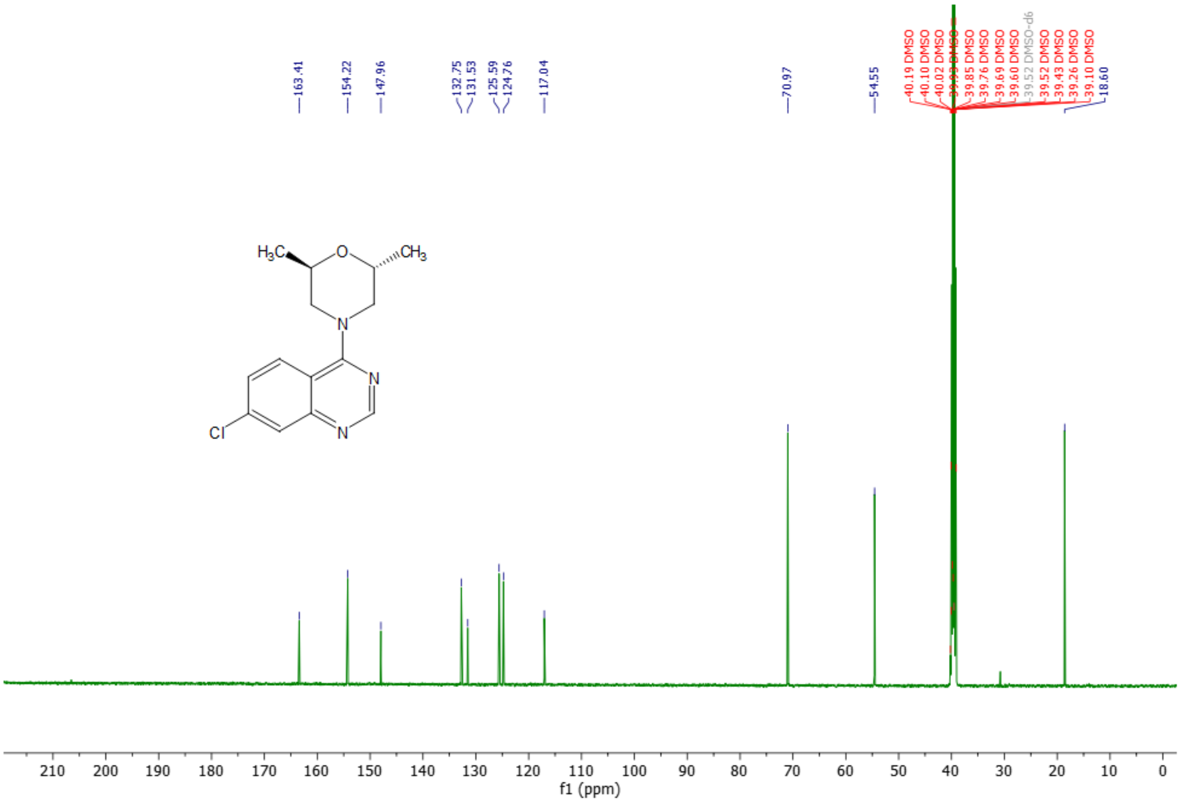

Figure S16b: ^{13}C NMR spectrum of (3p).

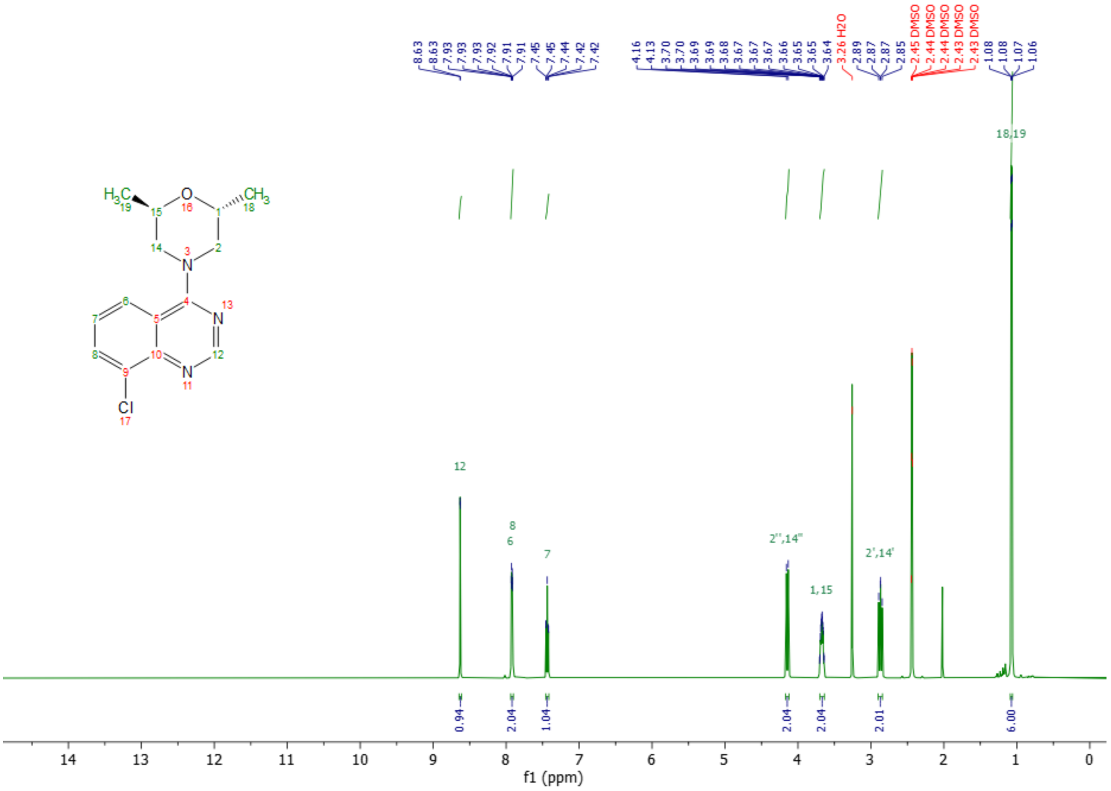

Figure S17a: ^{1}H NMR spectrum of (3q).

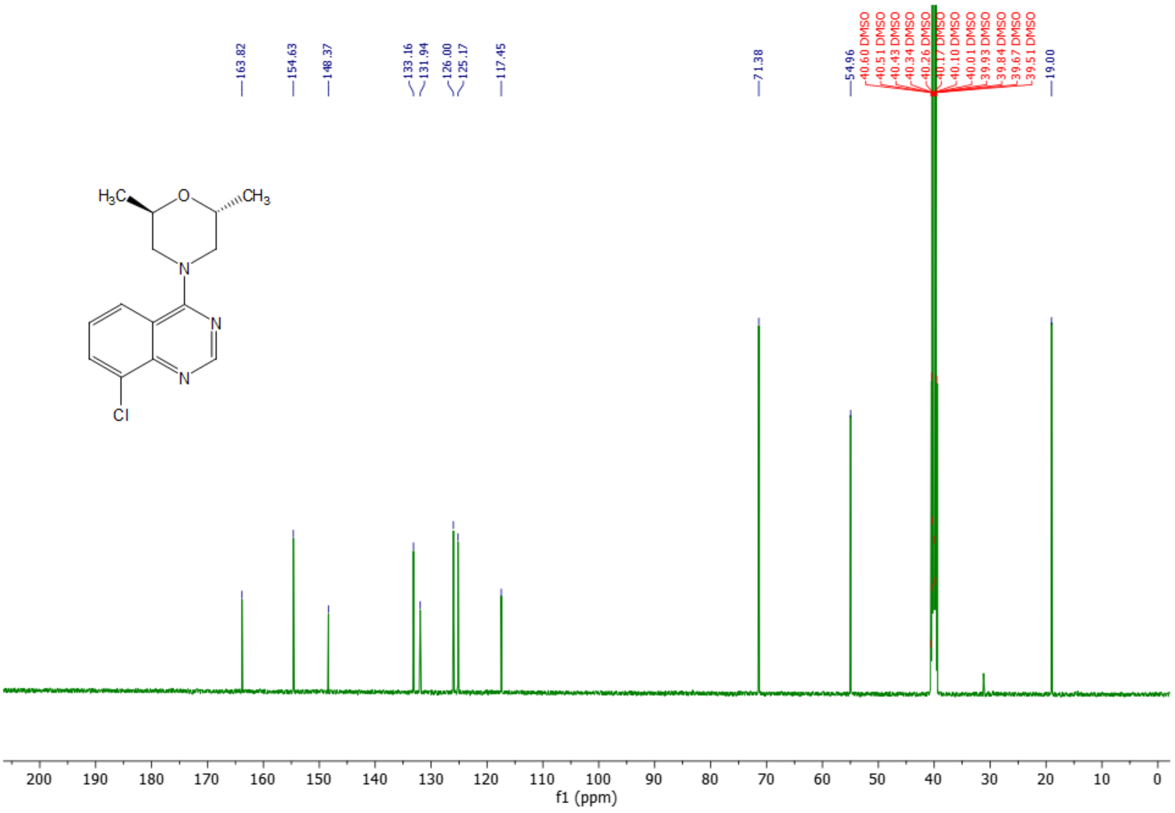

Figure S17b: ^{13}C NMR spectrum of (3q).

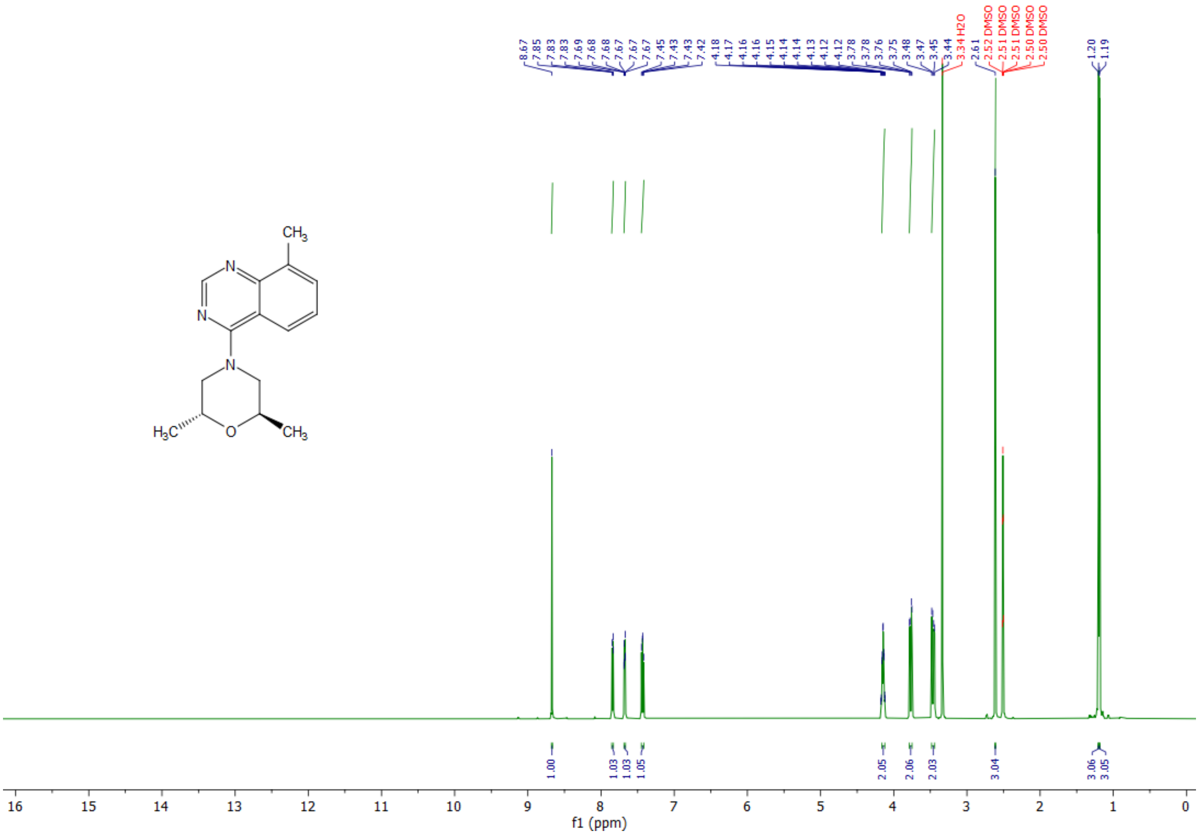

Figure S18a: ¹H NMR spectrum of (3r).

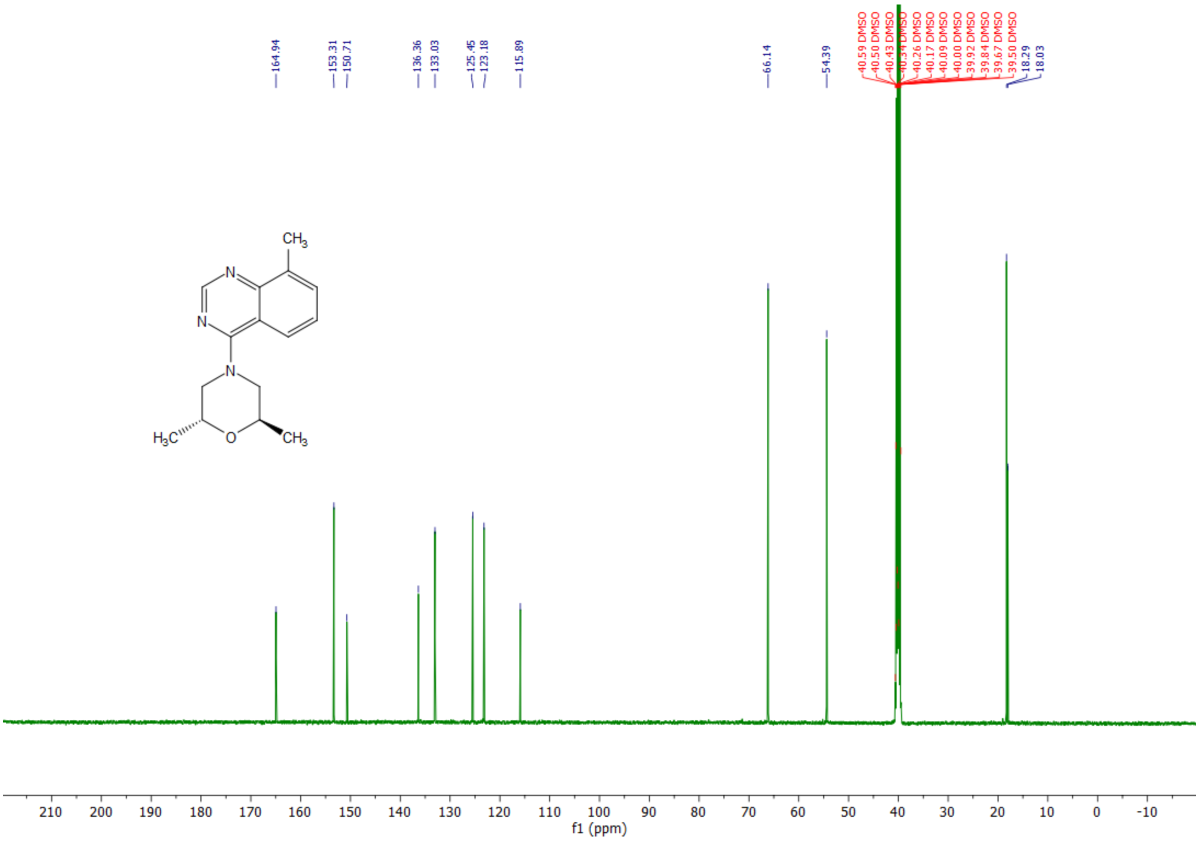

Figure S18b: ¹³C NMR spectrum of (3r).

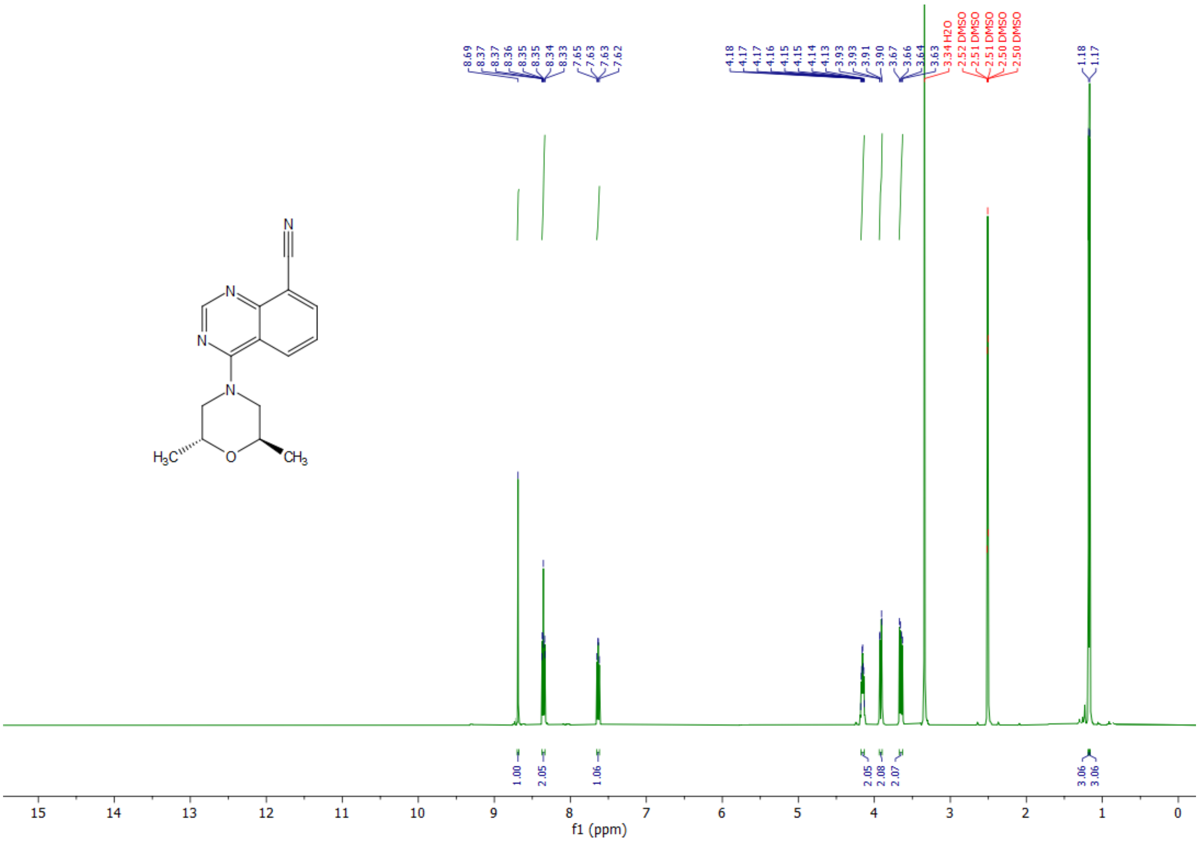

Figure S19a: ^{1}H NMR spectrum of (3s).

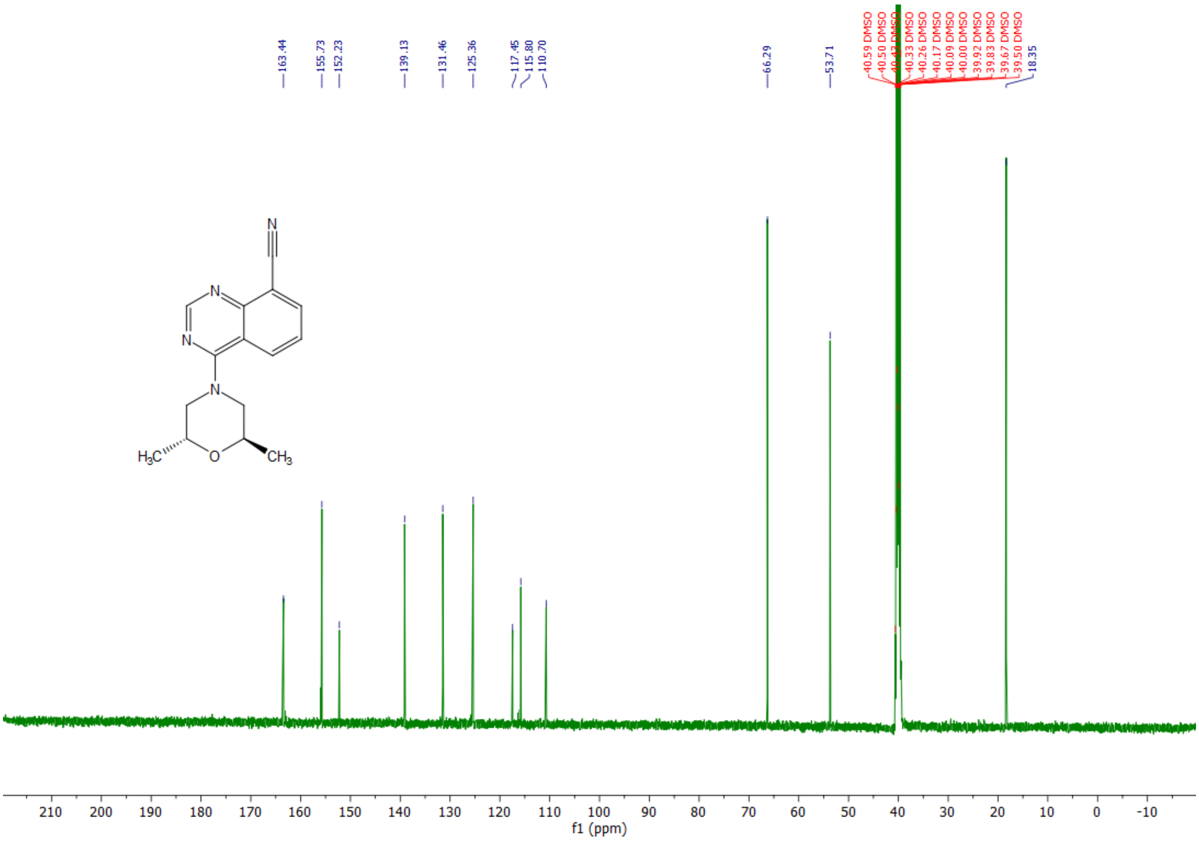

Figure S19b: 1H NMR spectrum of (3s).

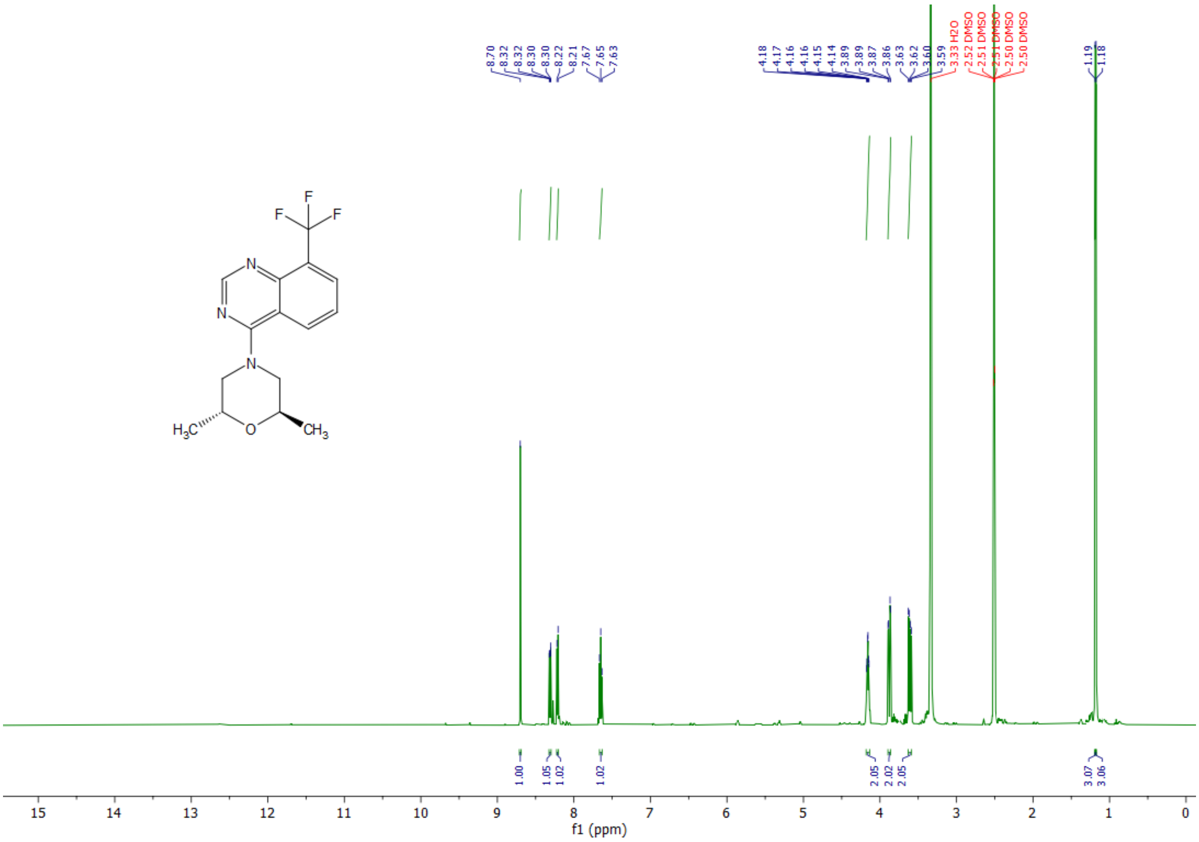

Figure S20a: ^{1}H NMR spectrum of (3t).

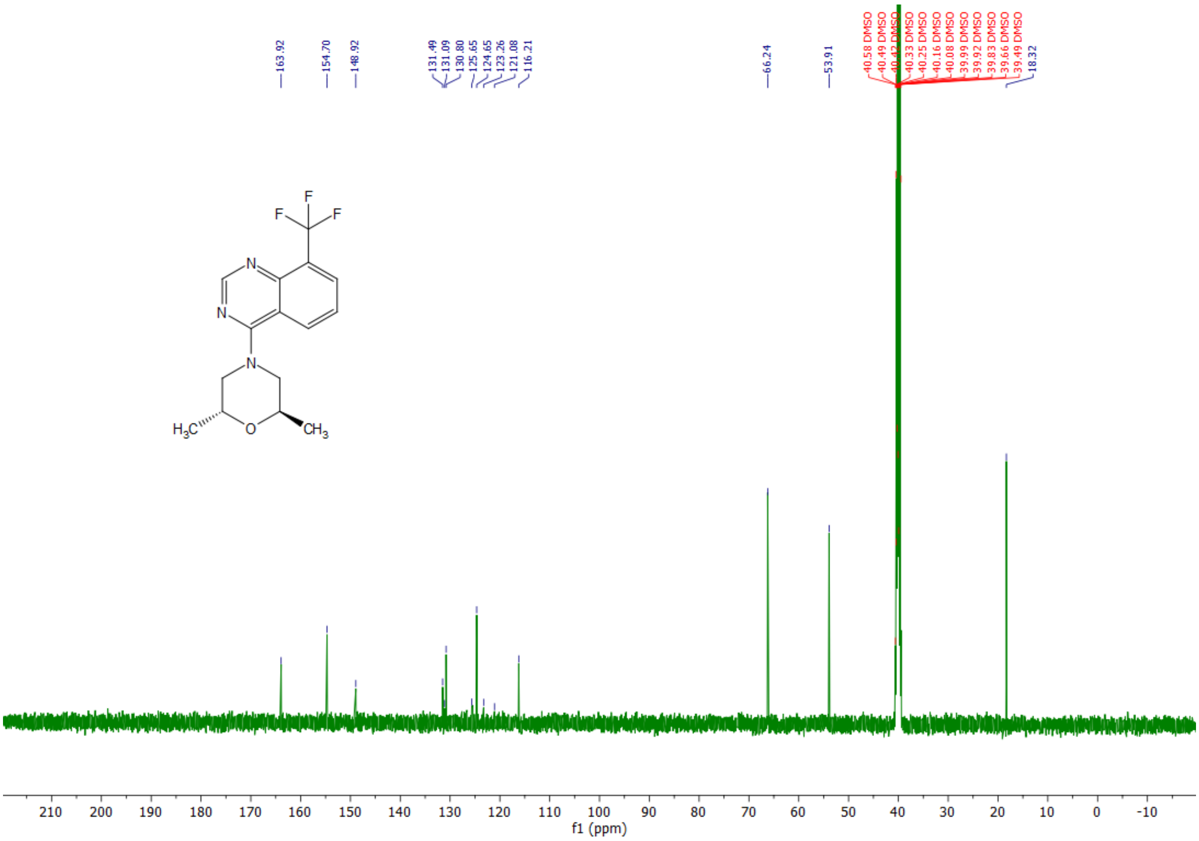

Figure S20b: 1H NMR spectrum of (3t).

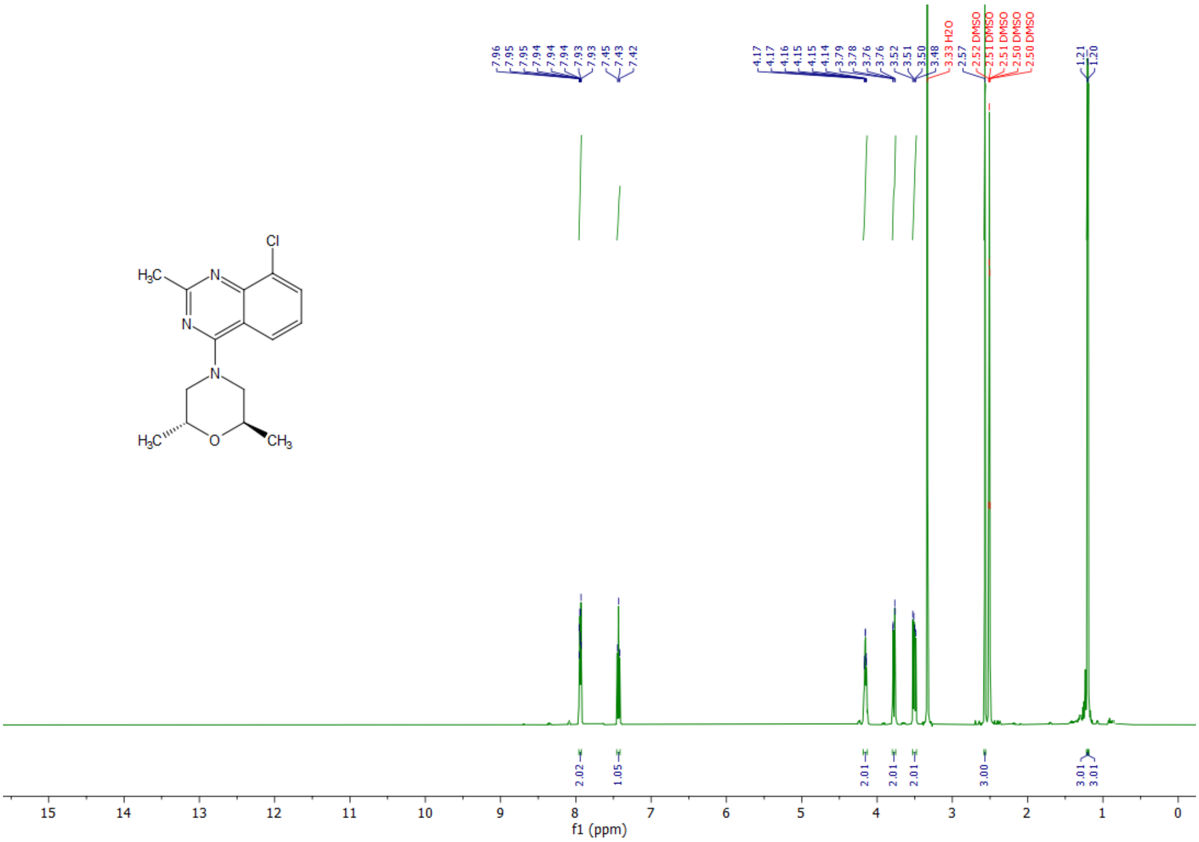

Figure S21a: ^{1}H NMR spectrum of (3u).

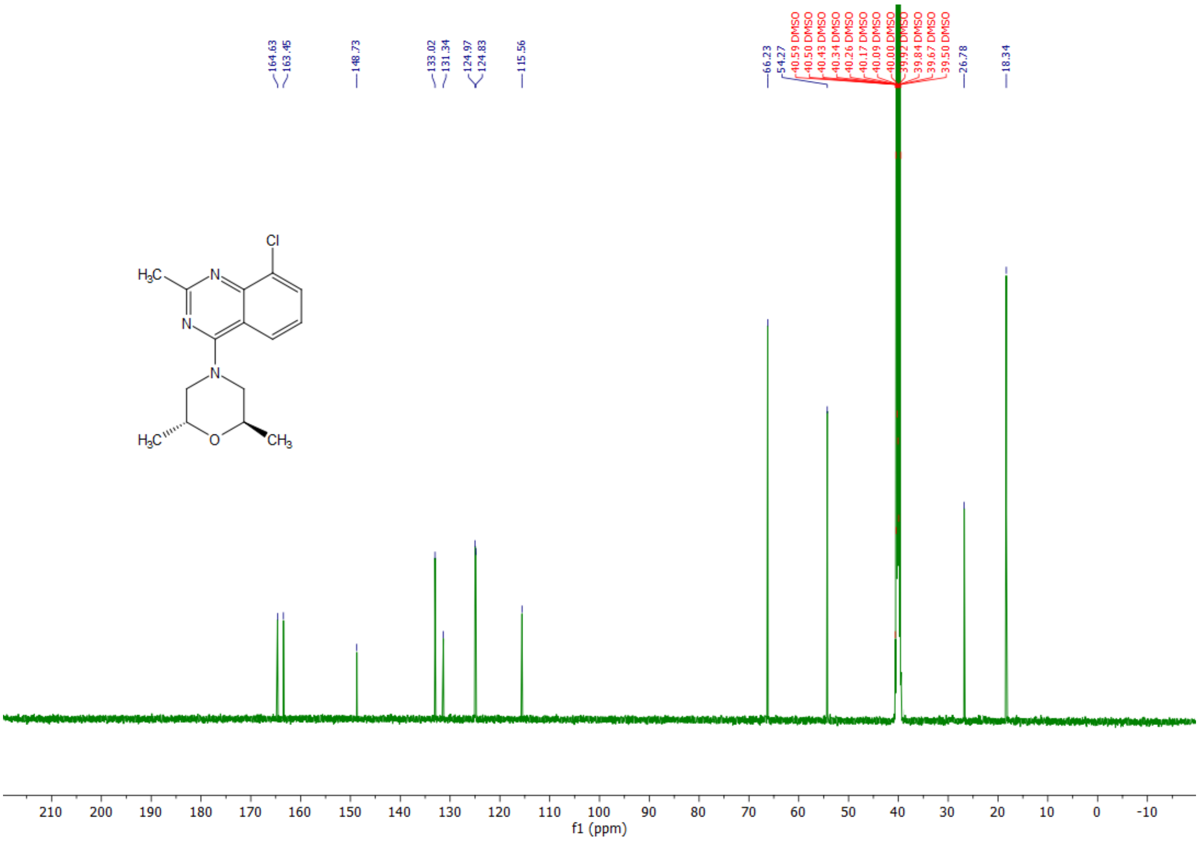

Figure S21b: ^{13}C NMR spectrum of (3u).

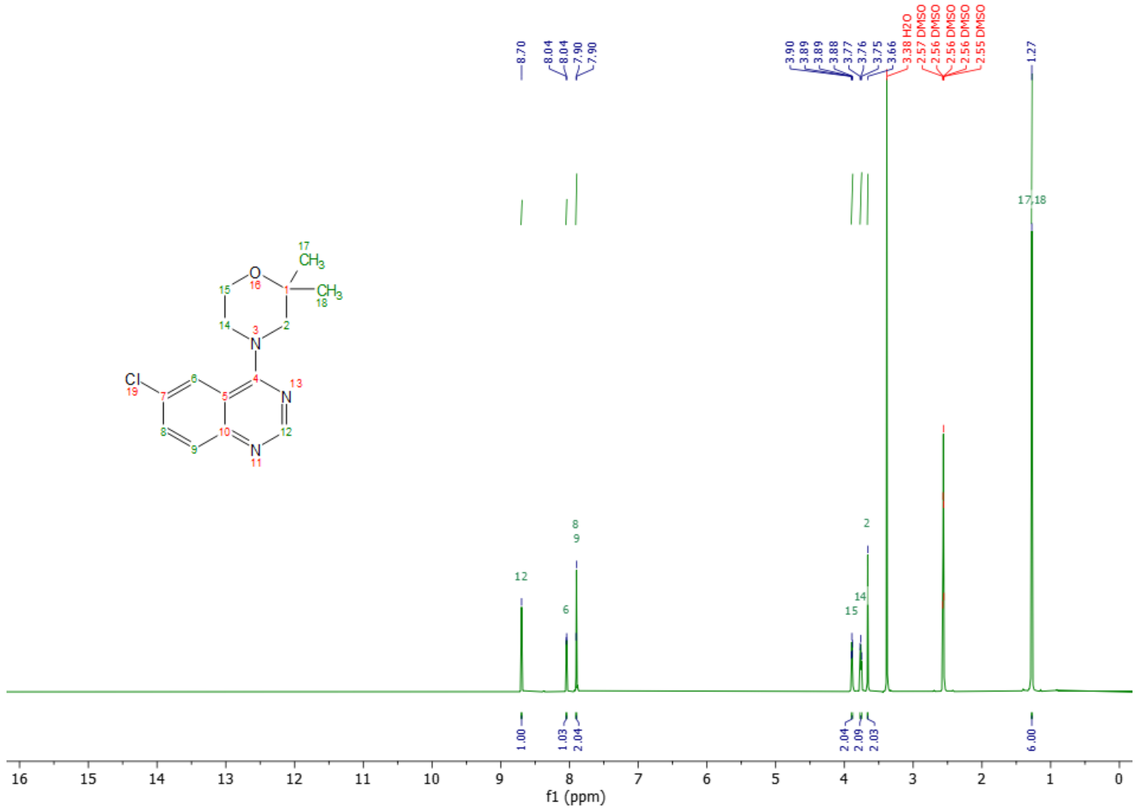

Figure S22a: ^{1}H NMR spectrum of (4a).

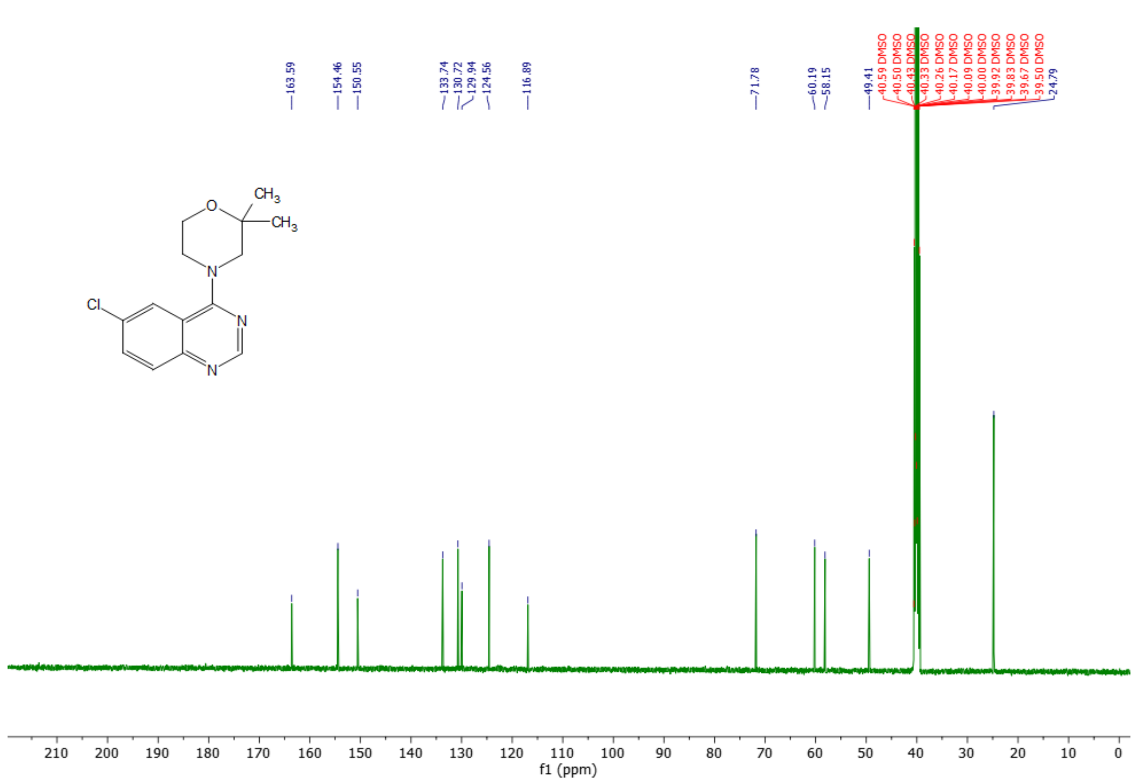

Figure S22b: ^{13}C NMR spectrum of (4a).

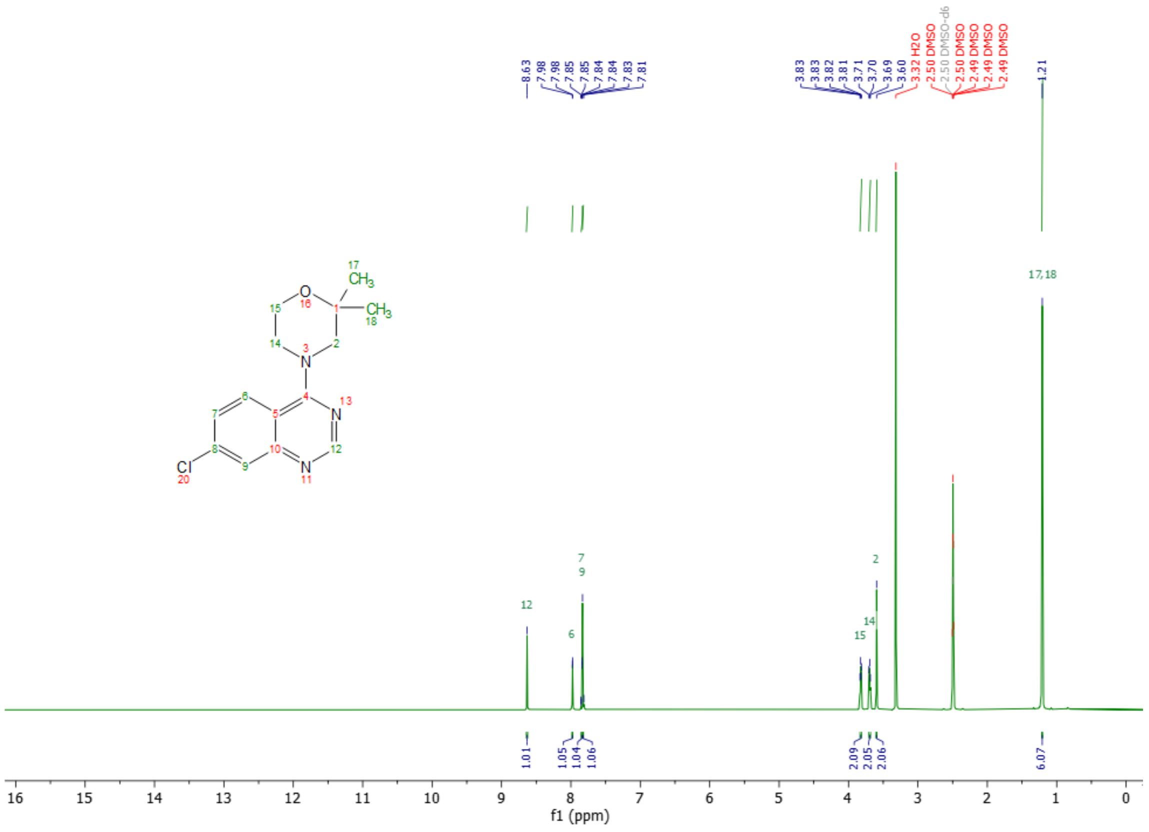

Figure S23a: ^{1}H NMR spectrum of (4b).

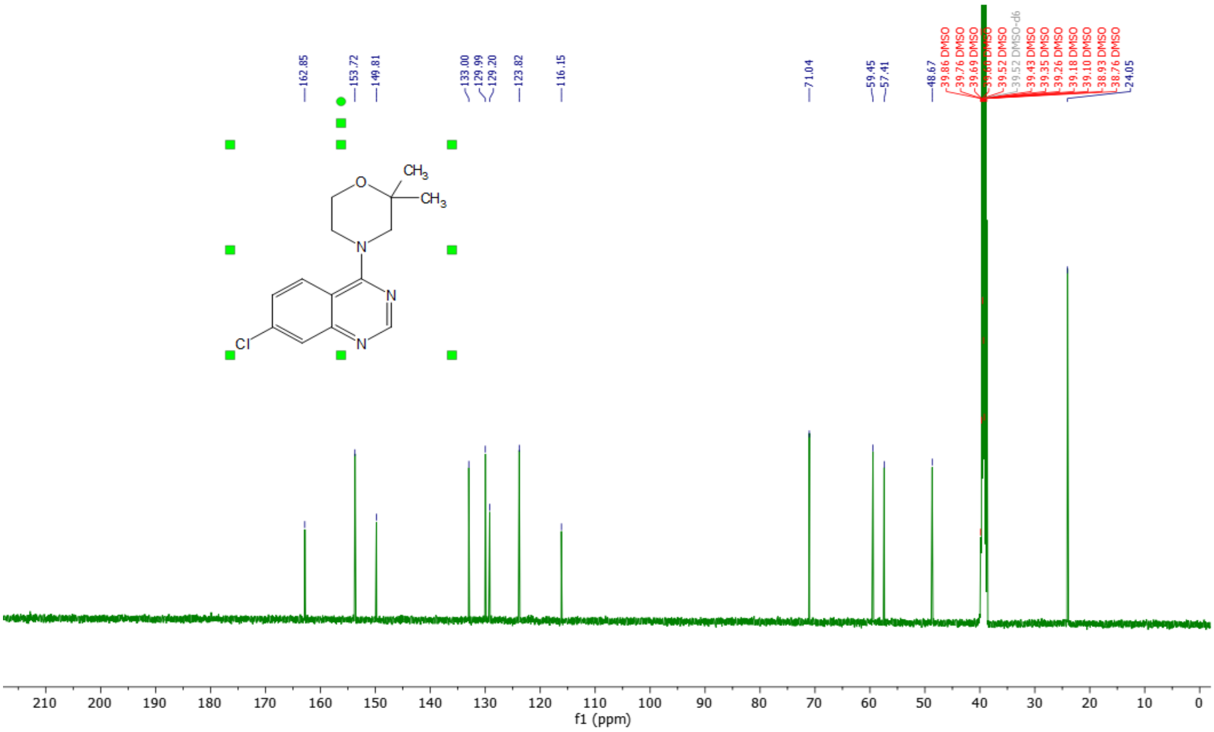

Figure S23b: ^{13}C NMR spectrum of (4b).

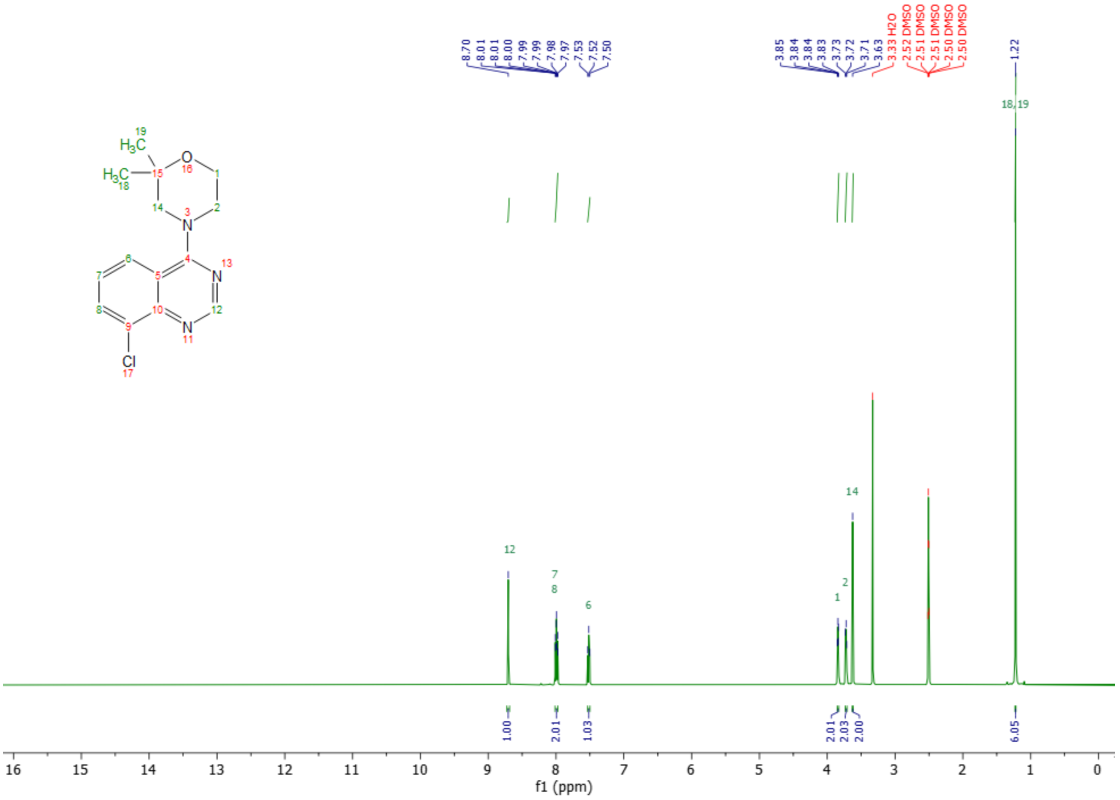

Figure S24a: ^{1}H NMR spectrum of (4c).

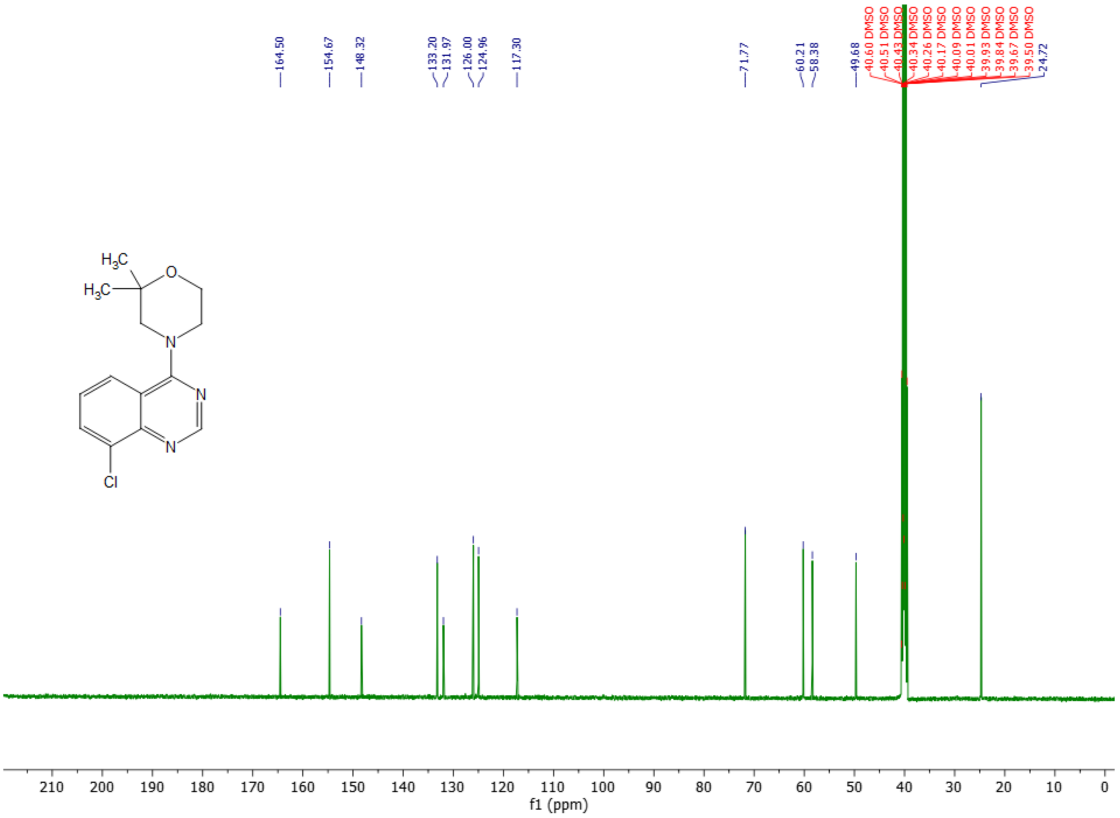

Figure S24b: ^{13}C NMR spectrum of (4c).

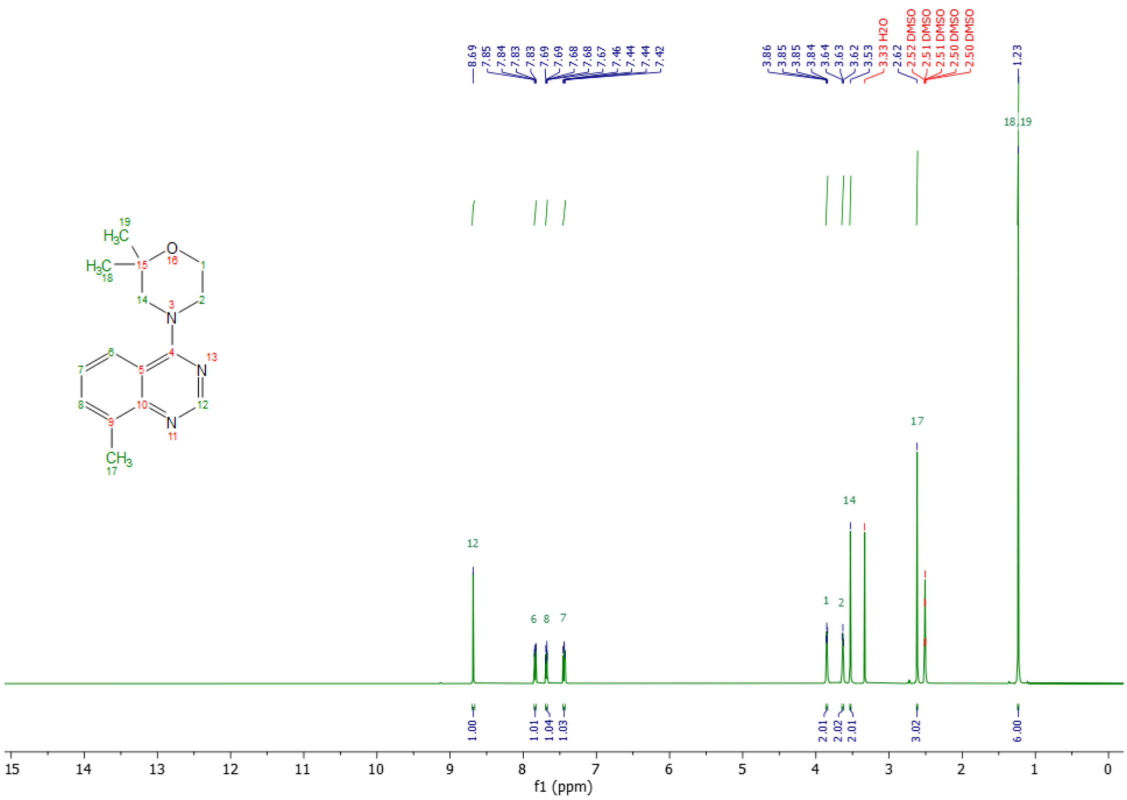

Figure S25a: 1H NMR spectrum of (4d).

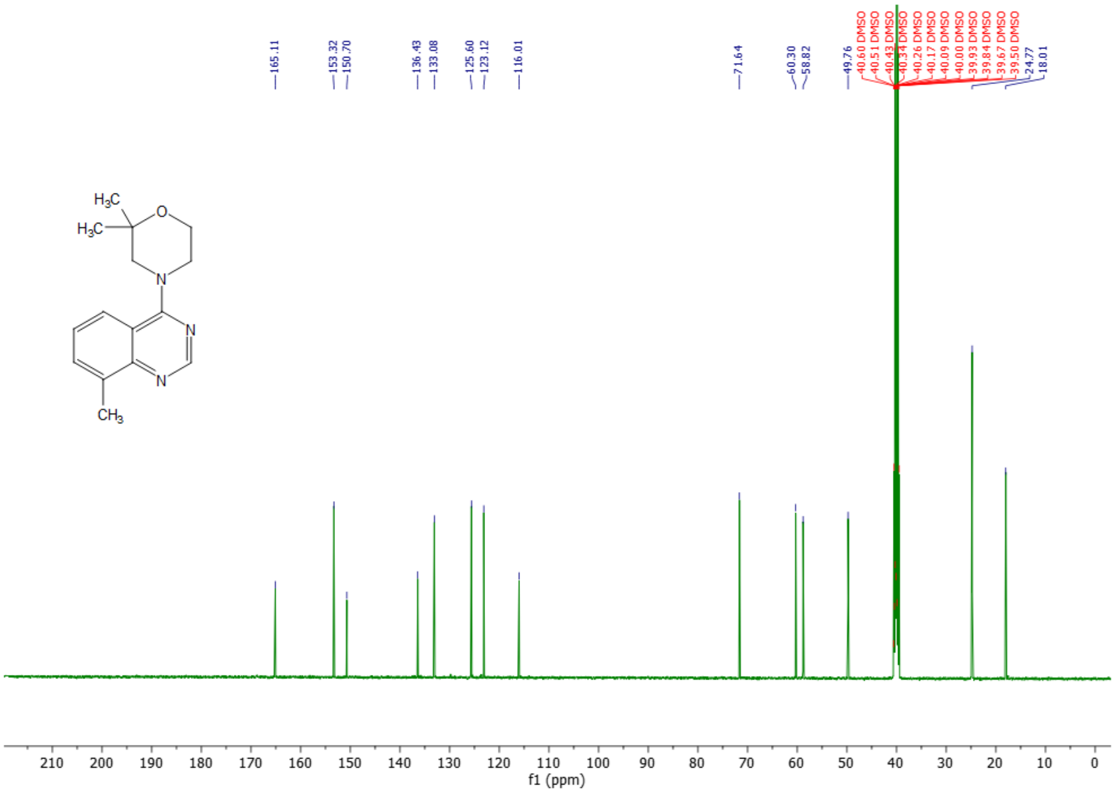

Figure S25b: ^{13}C NMR spectrum of (4d).

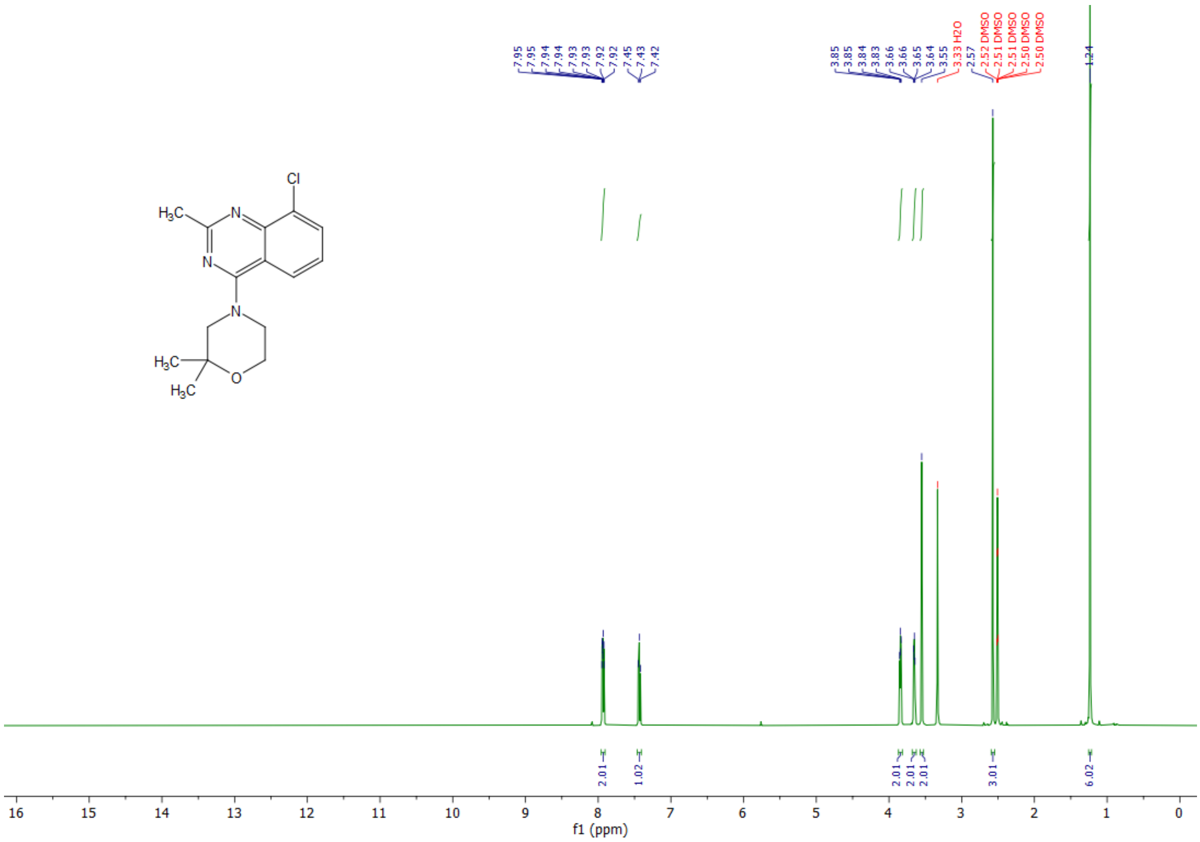

Figure S26a: ^{1}H NMR spectrum of (4e).

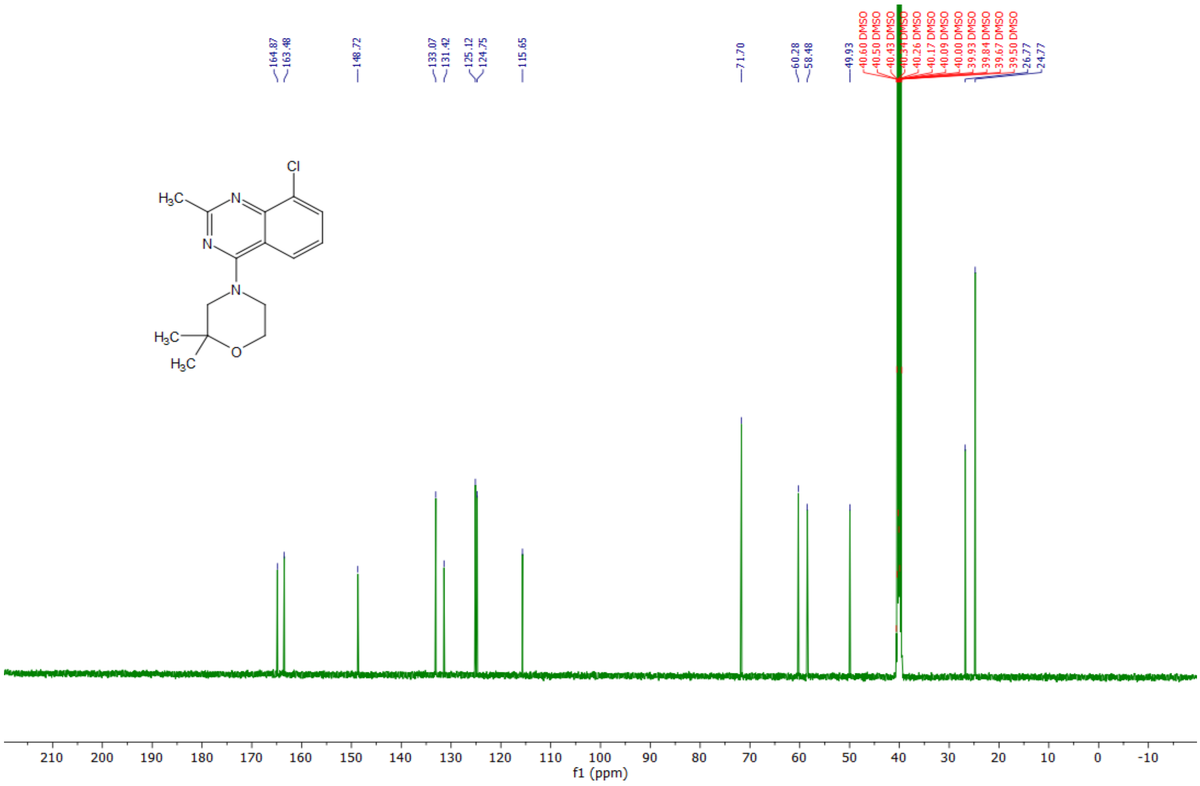

Figure S26b: ^{13}C NMR spectrum of (4e).

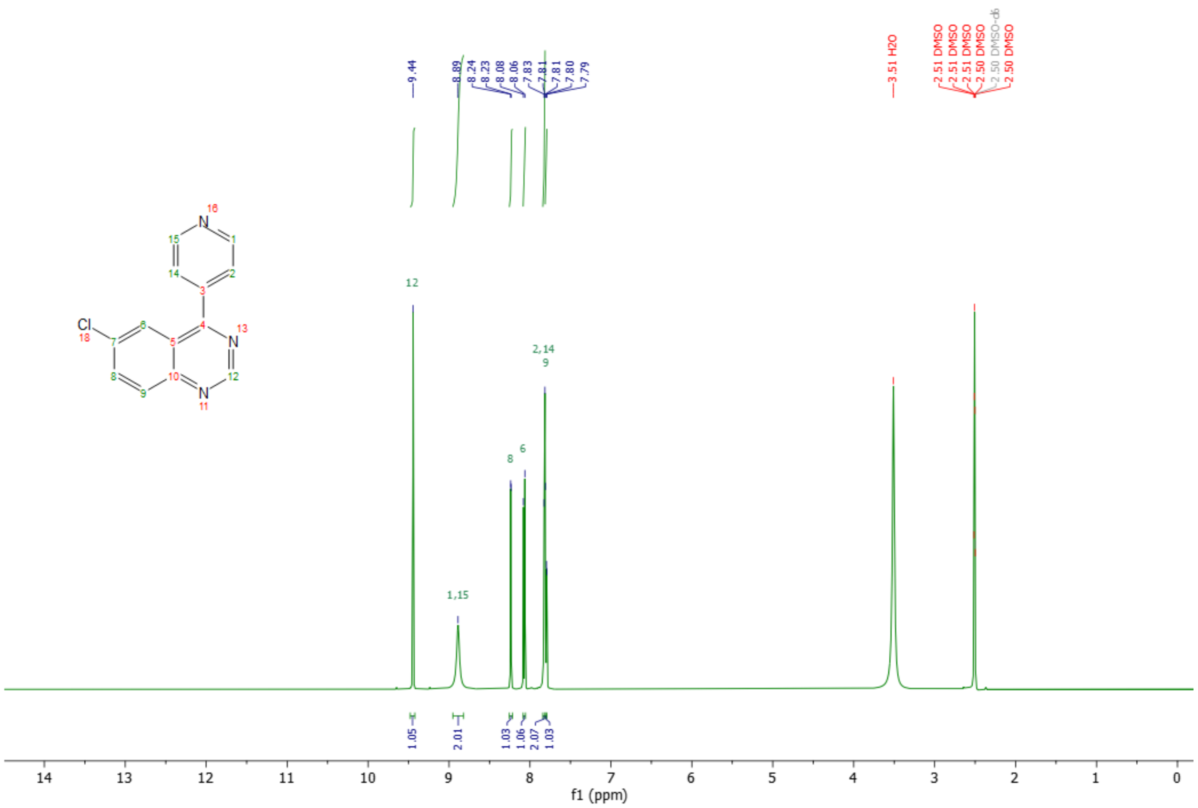

Figure S27a: ^{1}H NMR spectrum of (6a).

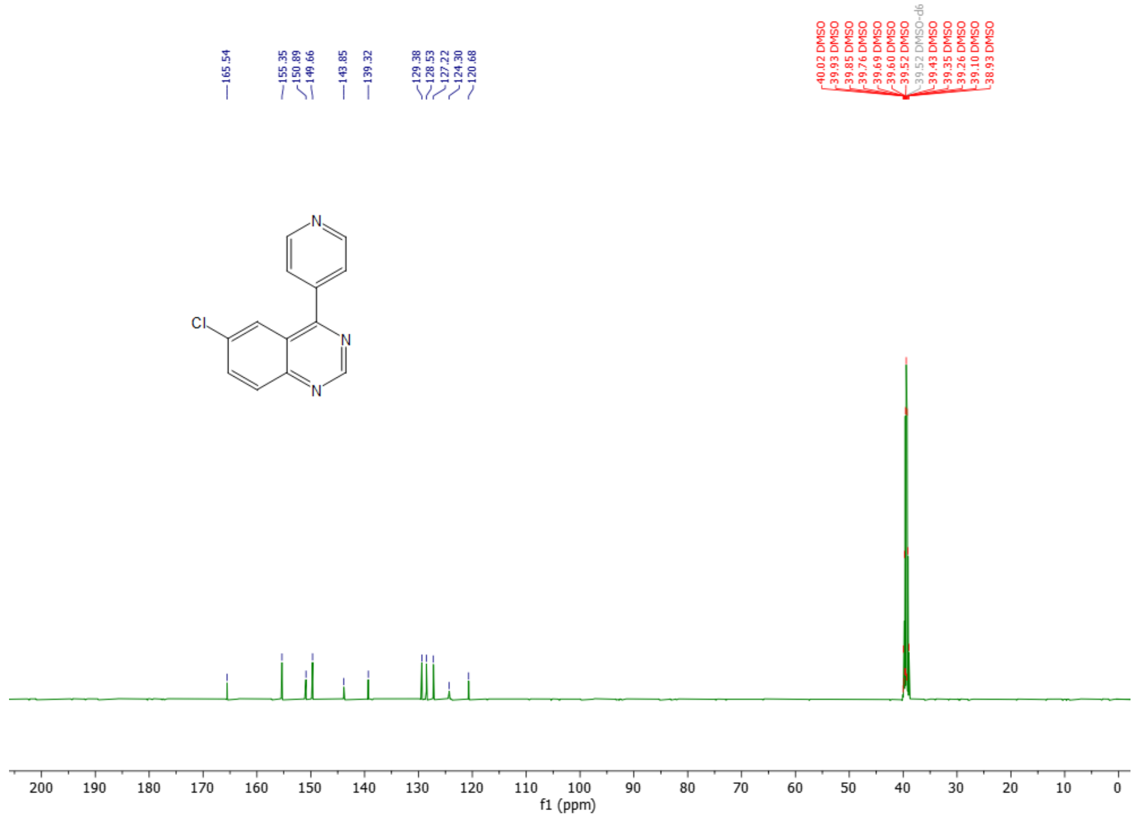

Figure S27b: ^{13}C NMR spectrum of (6a).

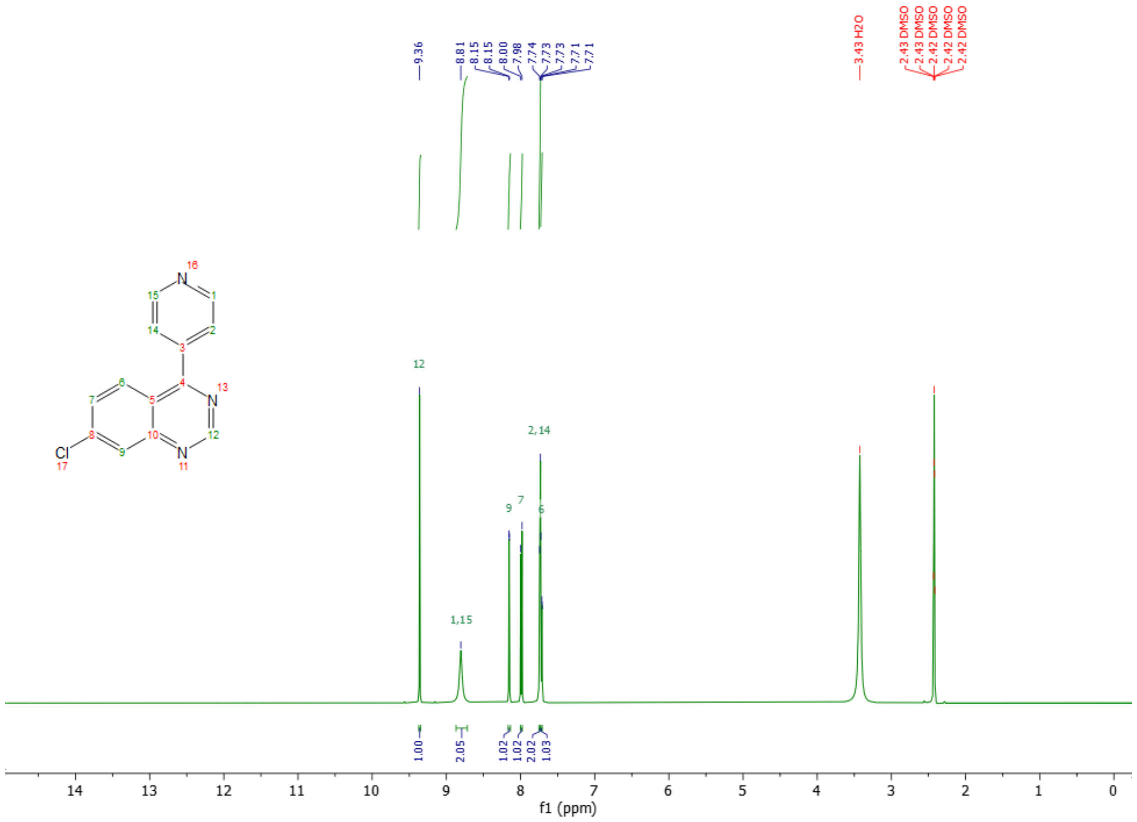

Figure S28a: ^{1}H NMR spectrum of (6b).

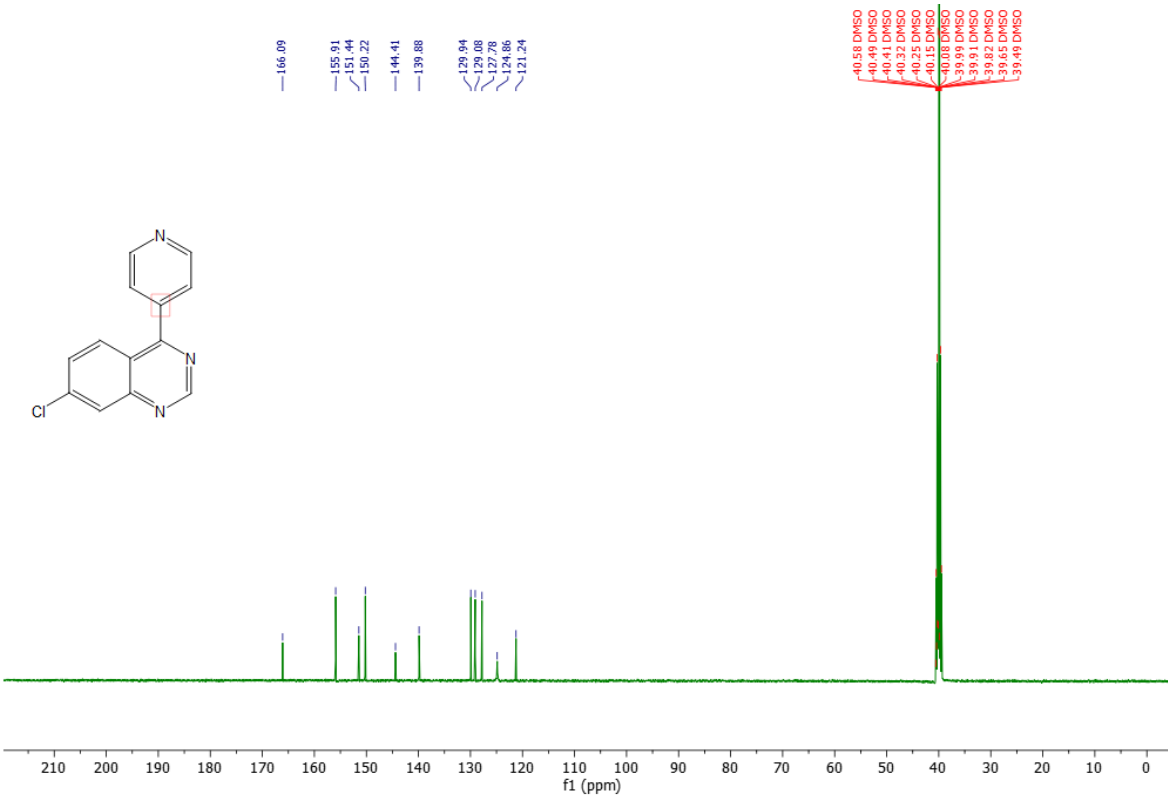

Figure S28b: ^{13}C NMR spectrum of (6b).

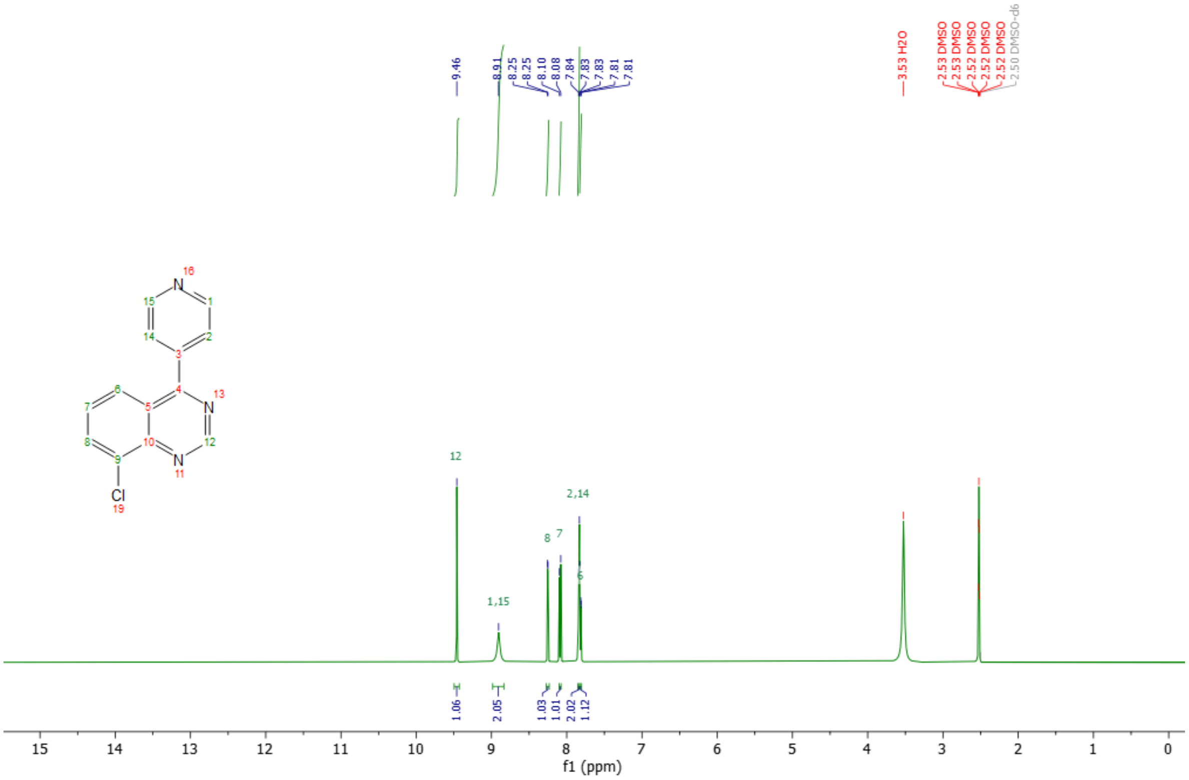

Figure S29a: ^{1}H NMR spectrum of (6c).

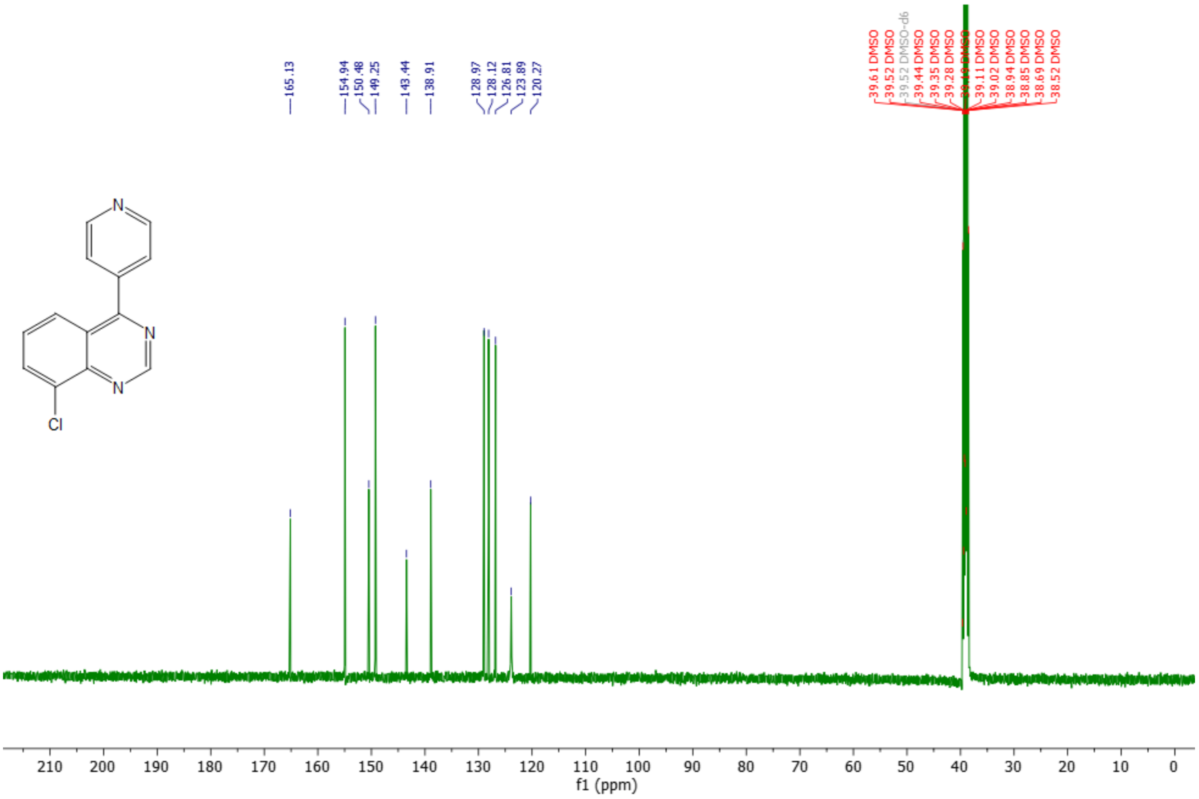

Figure S29b: ^{13}C NMR spectrum of (6c).

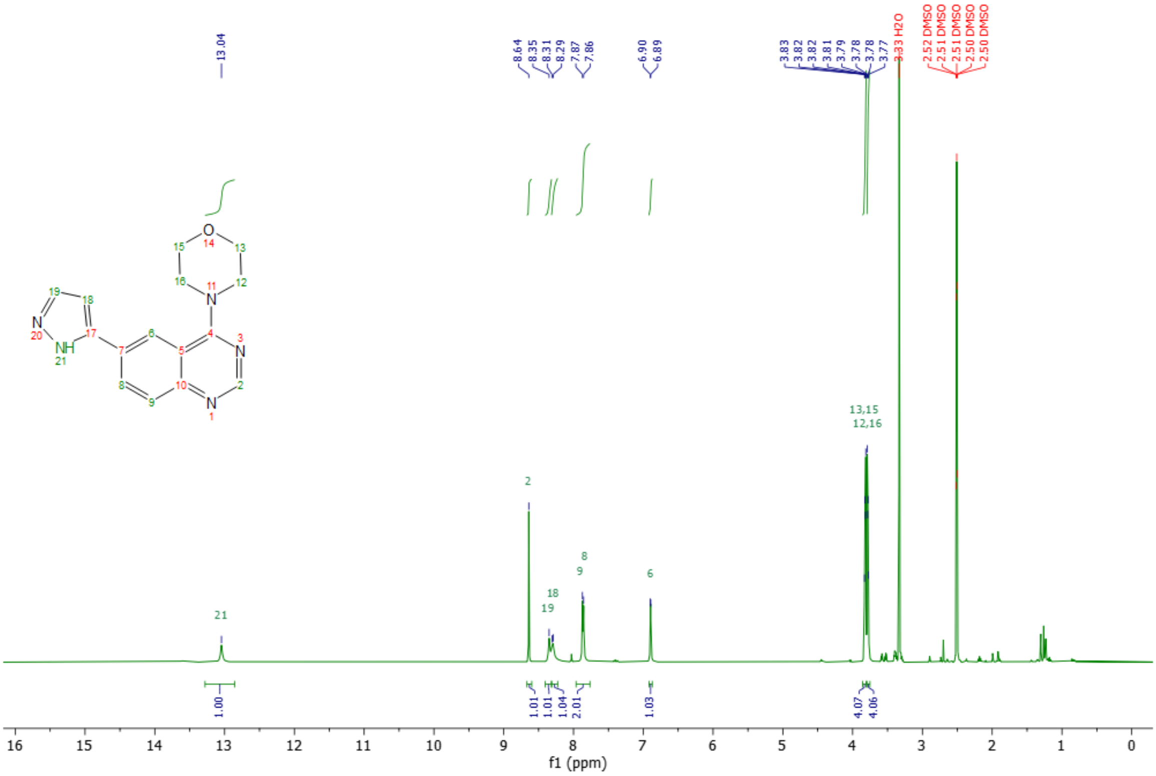

Figure S30a: ^{1}H NMR spectrum of (8a).

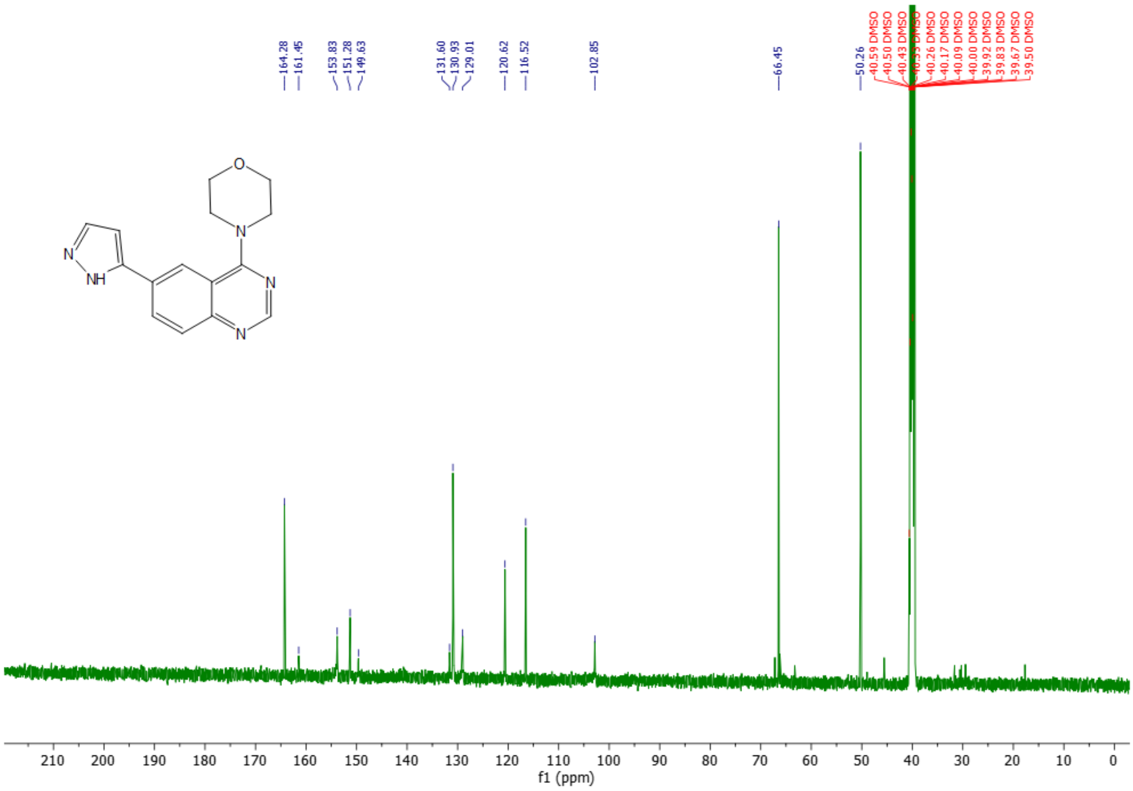

Figure S30b: ^{13}C NMR spectrum of (8a).

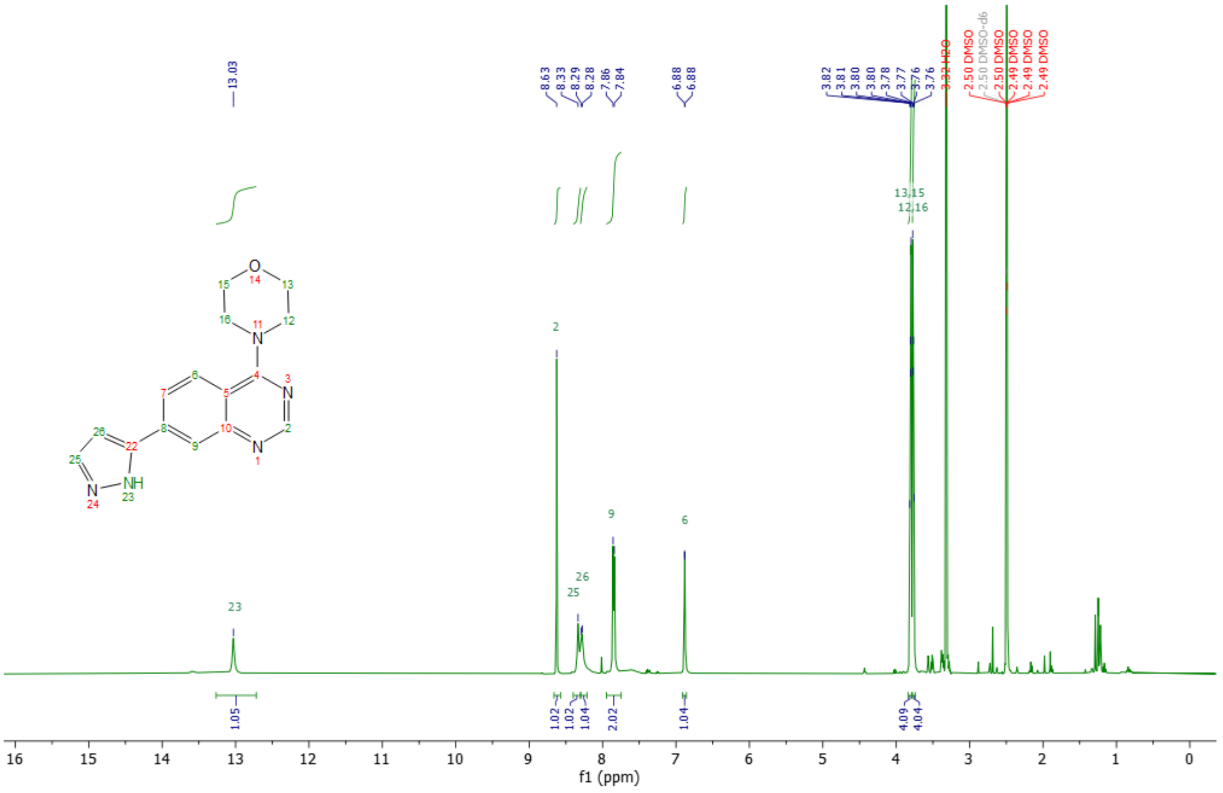

Figure S31a: ^{1}H NMR spectrum of (8b).

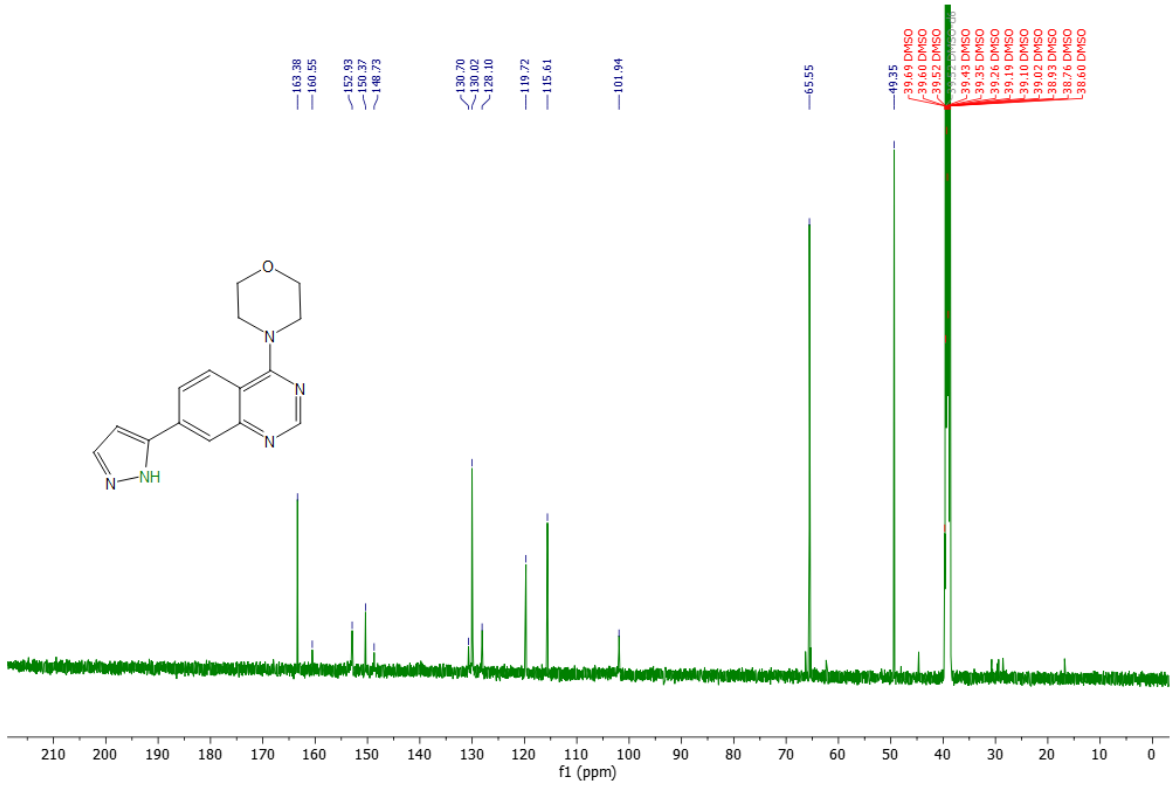

Figure S31b: ^{13}C NMR spectrum of (8b).

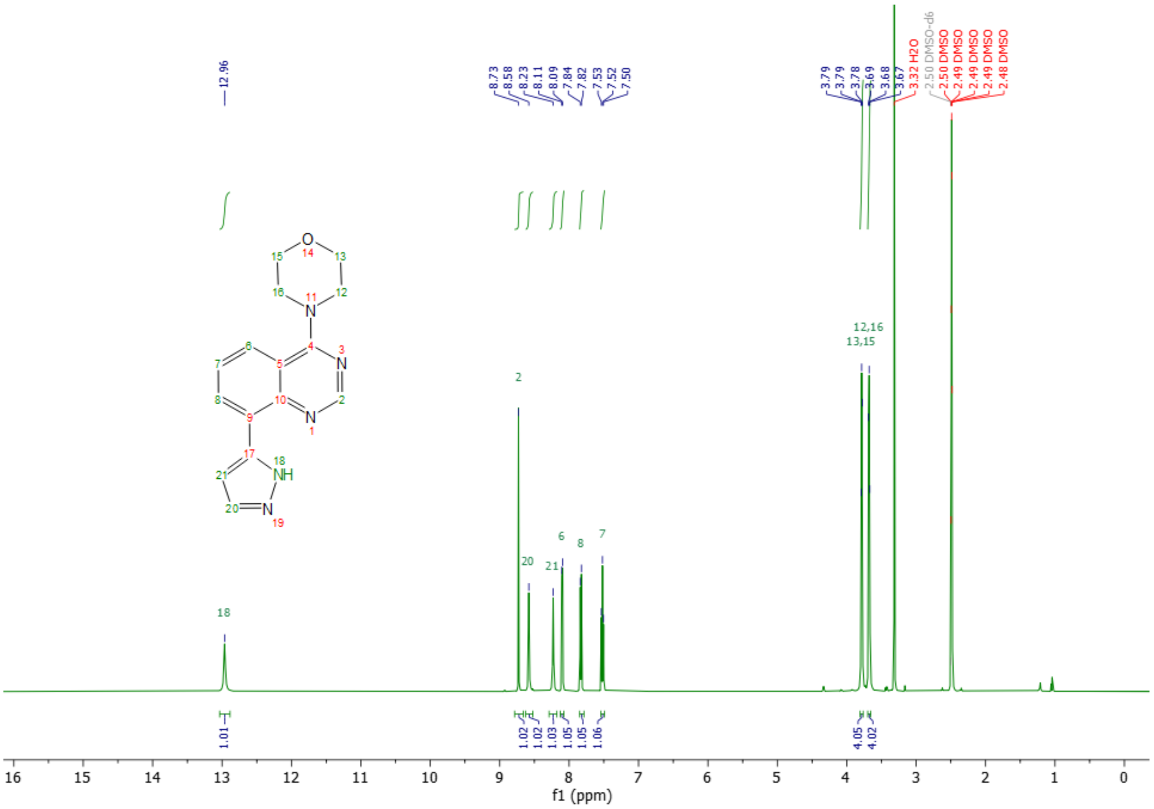

Figure S32a: ^{1}H NMR spectrum of (8c).

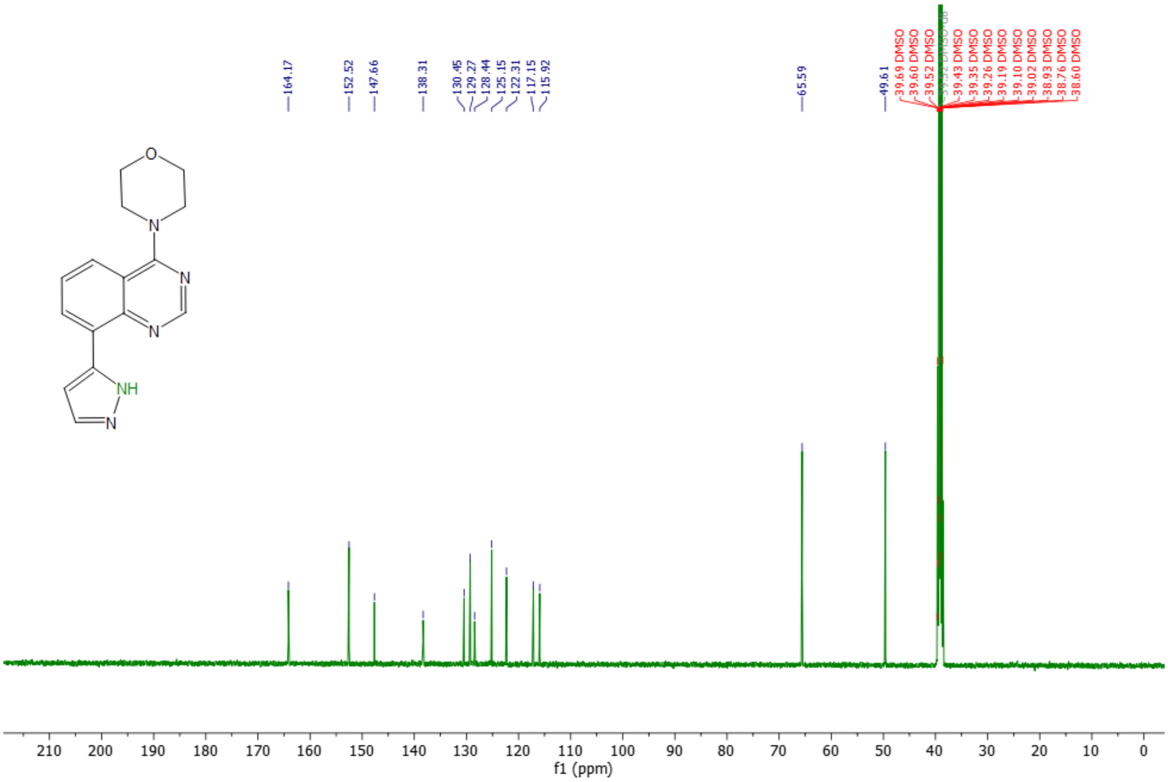

Figure S32b: ^{13}C NMR spectrum of (8c).

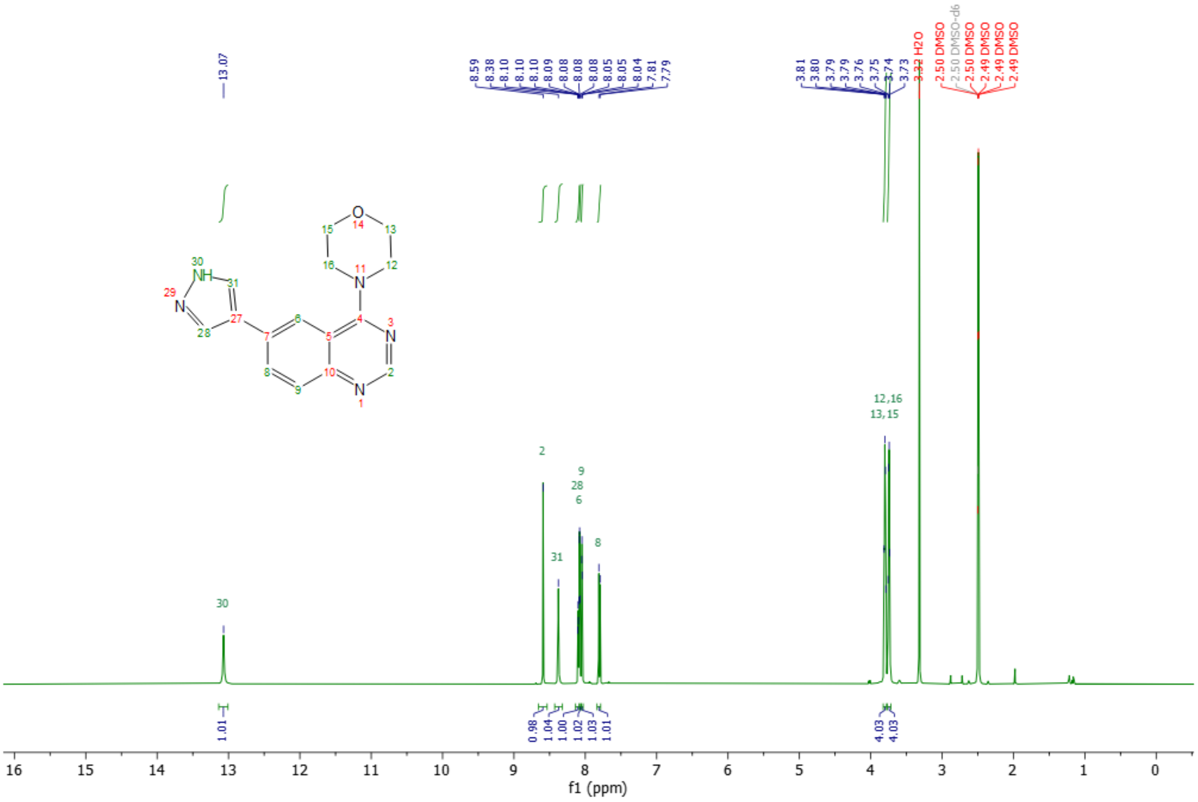

Figure S33a: 1H NMR spectrum of (8d).

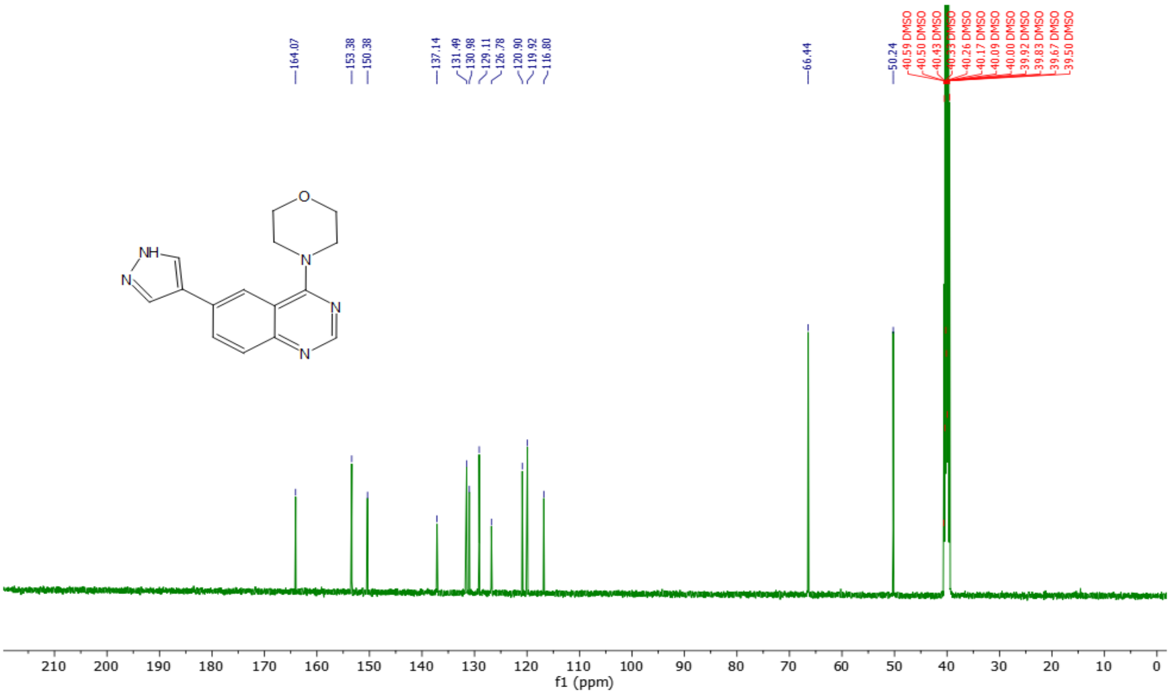

Figure S33b: ^{13}C NMR spectrum of (8d).

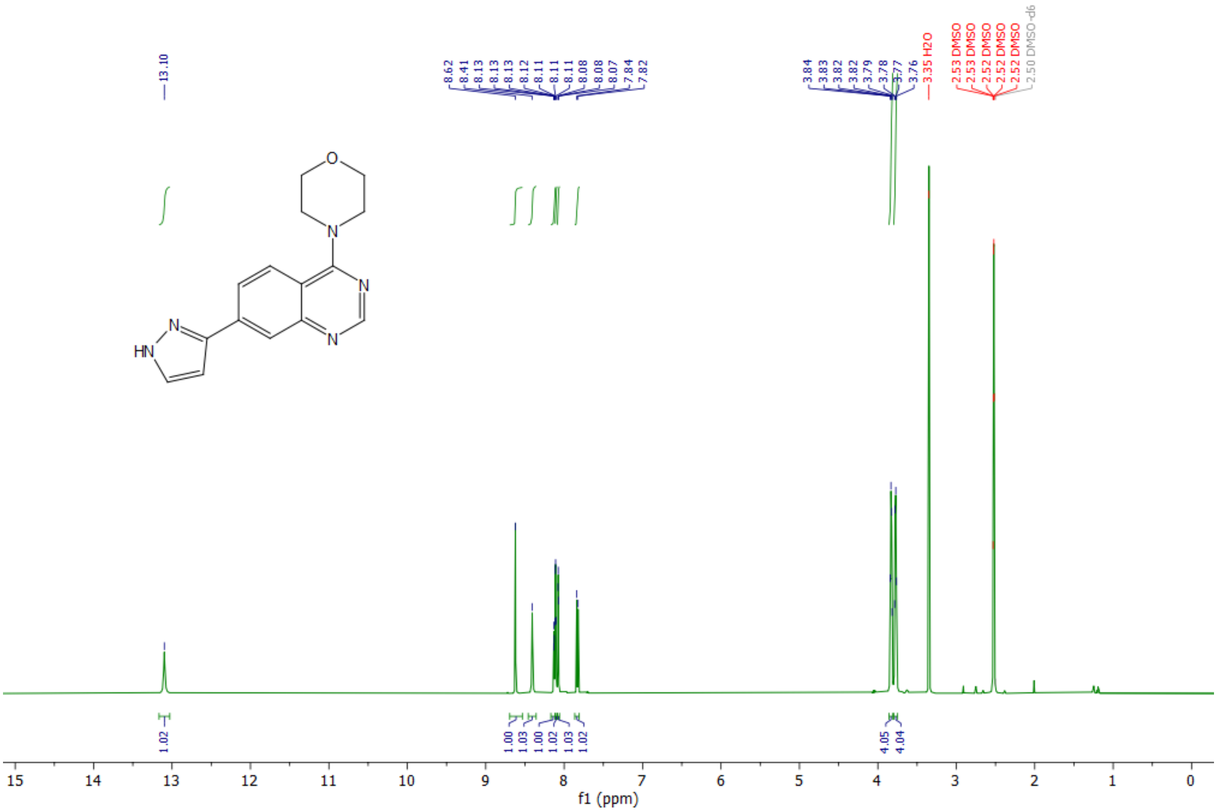

Figure S34a: ^{1}H NMR spectrum of (8e).

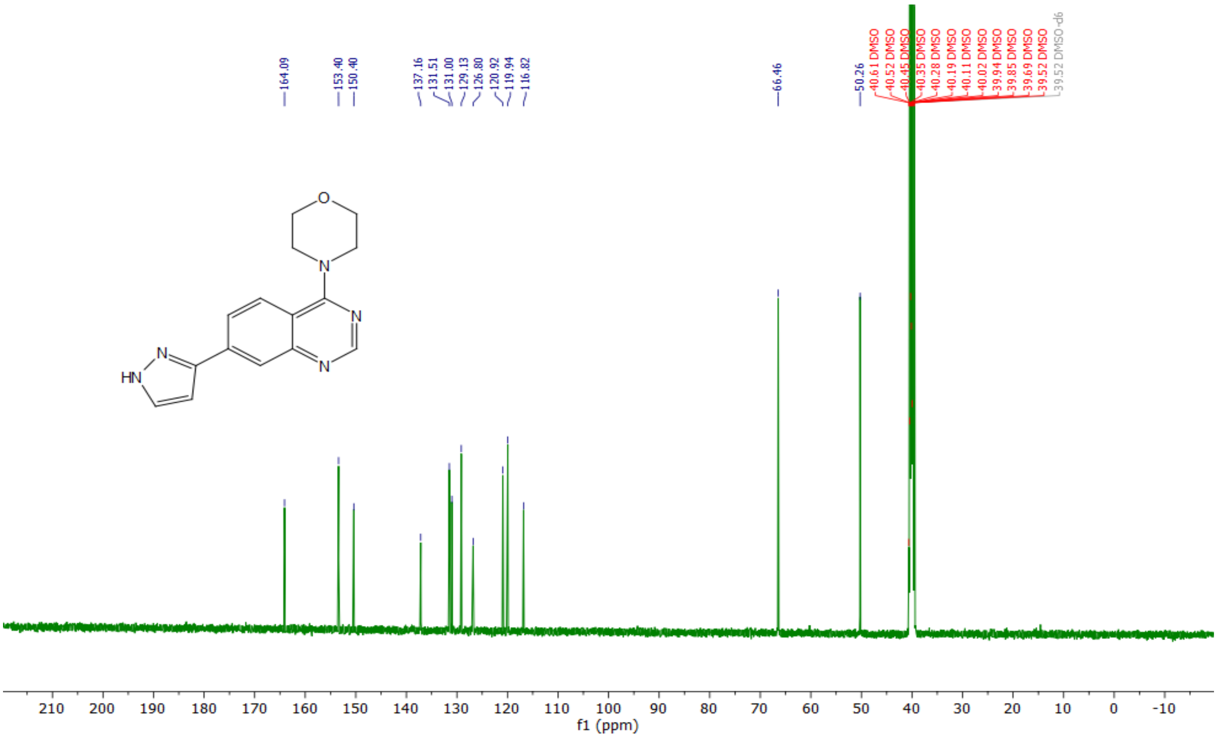

Figure S34b: ^{13}C NMR spectrum of (8e).

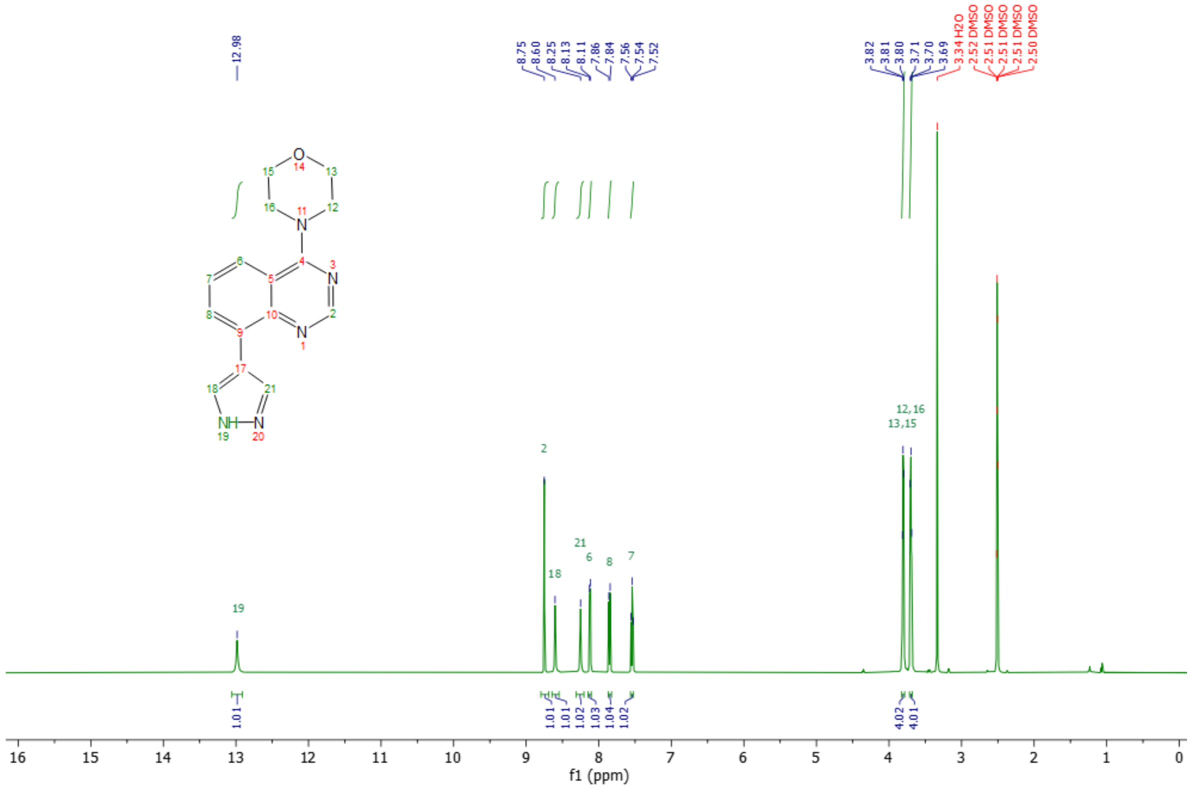

Figure S35a: ^{1}H NMR spectrum of (8f).

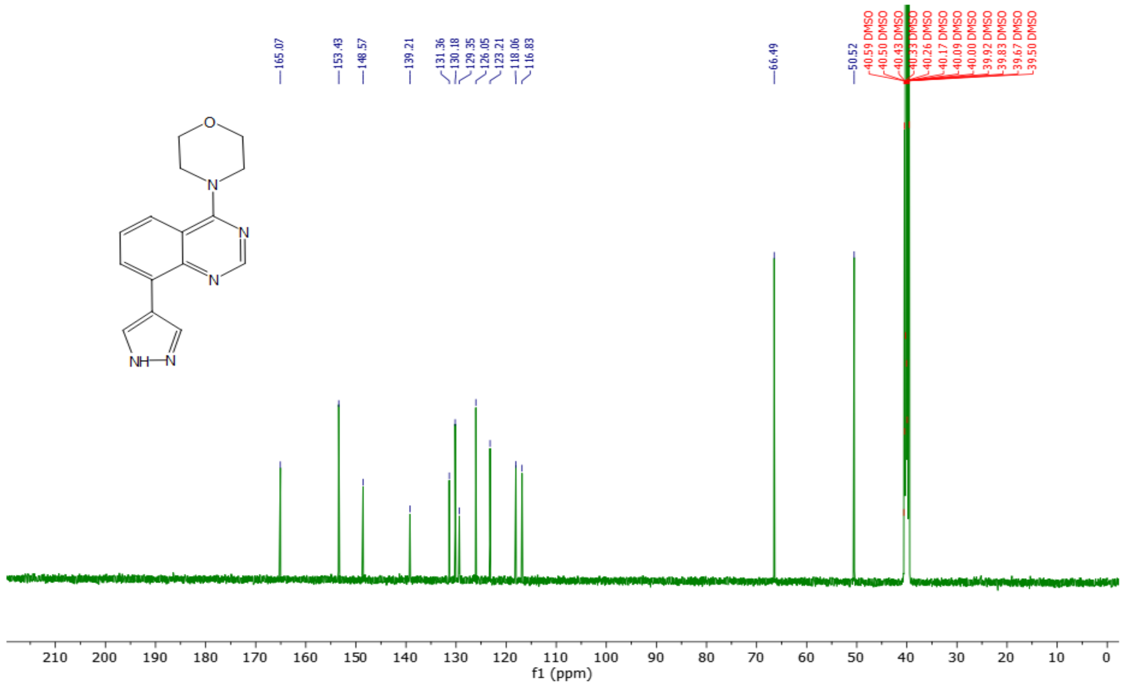

Figure S35b: ^{13}C NMR spectrum of (8f).

Mass spectrum of compounds (3a–3u), **(4a–4e)**, **(6a–6c)**, and **(8a–8f)**.

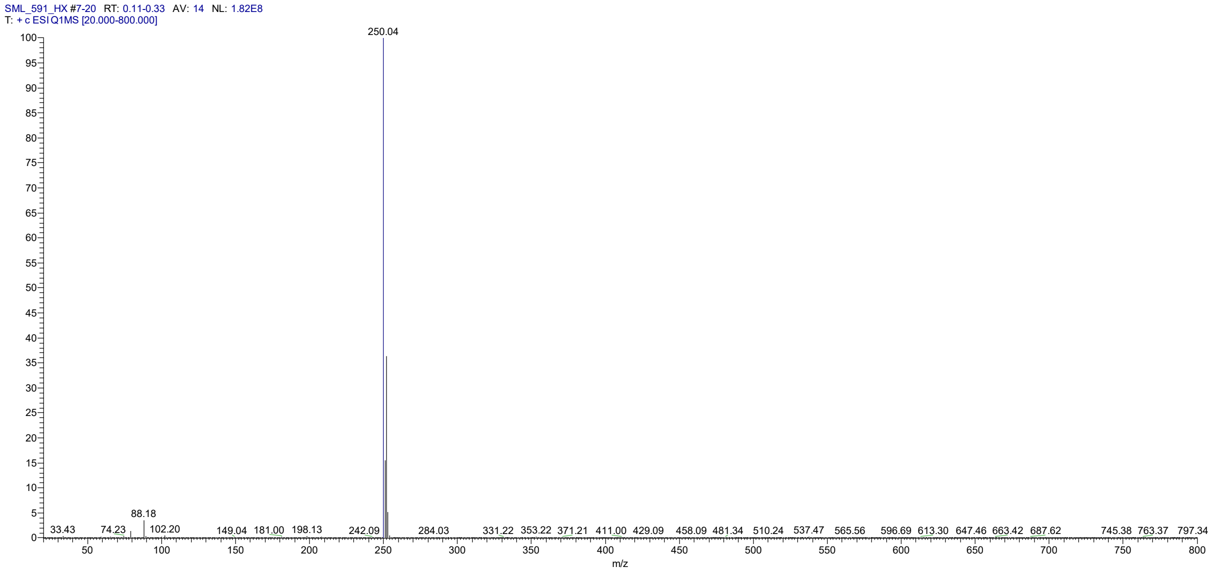

Figure S1c: MS spectrum of (3a).

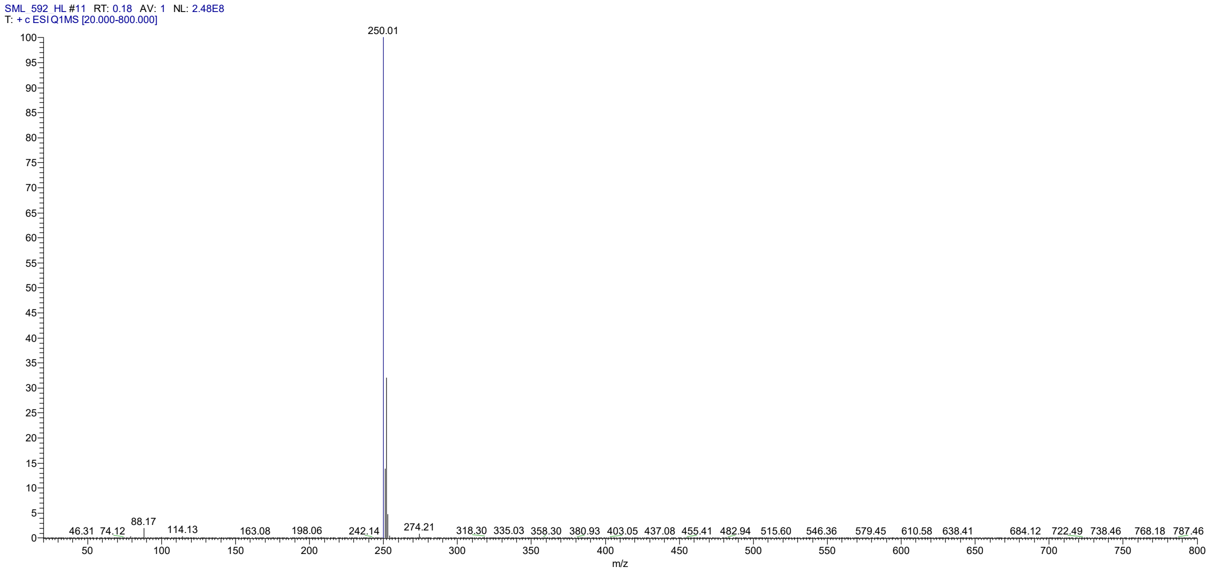

Figure S2c: MS spectrum of (3b).

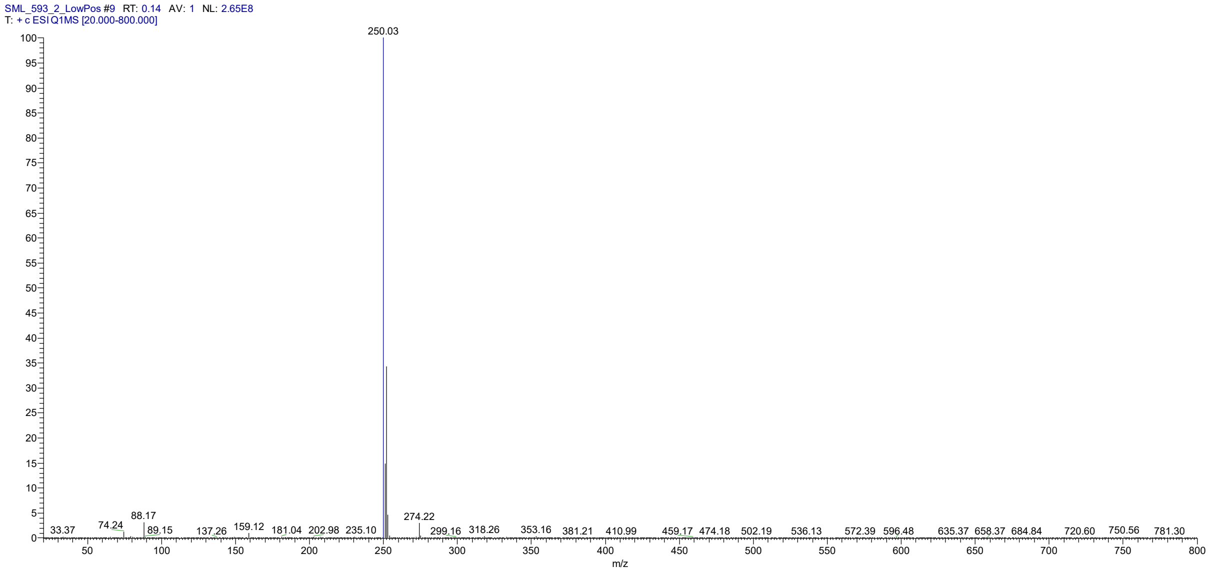

Figure S3c: MS spectrum of (3c).

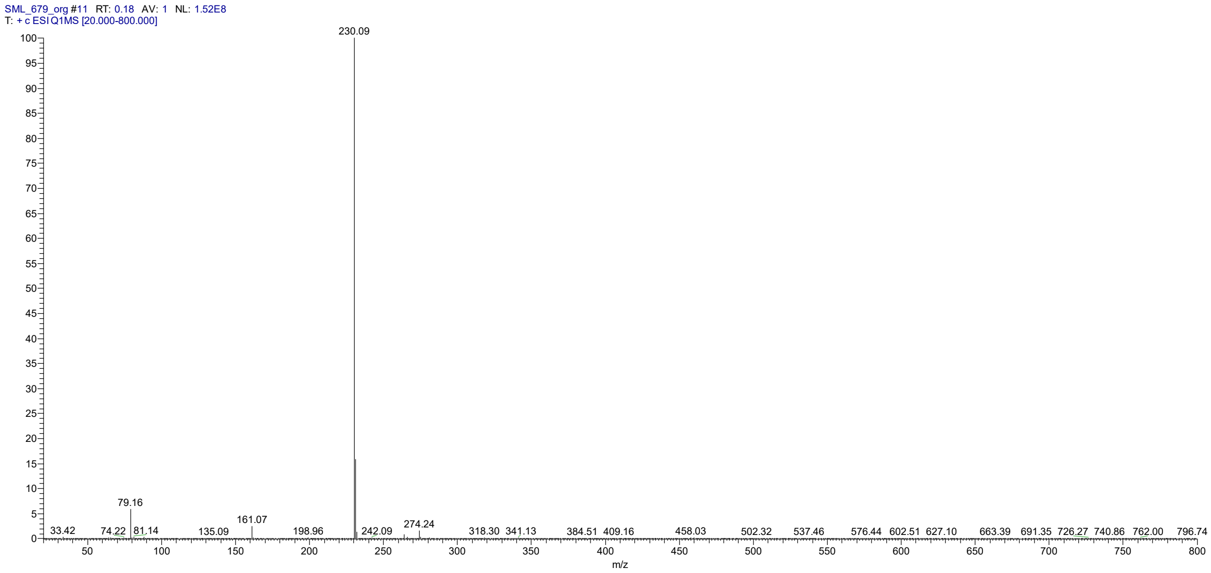

Figure S4c: MS spectrum of (3d).

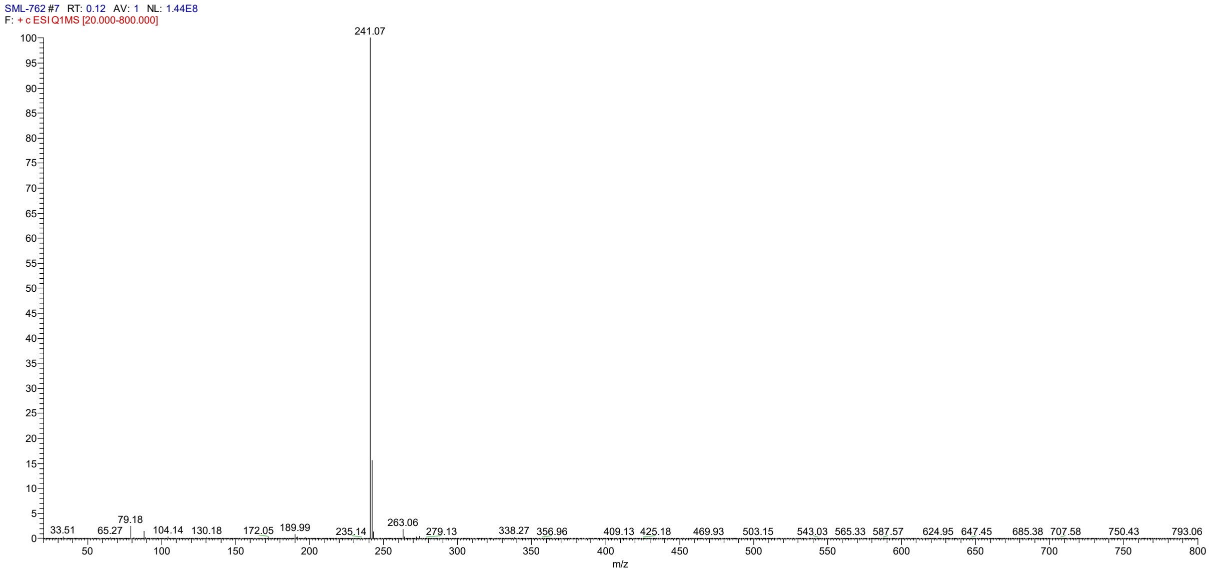

Figure S5c: MS spectrum of (3e).

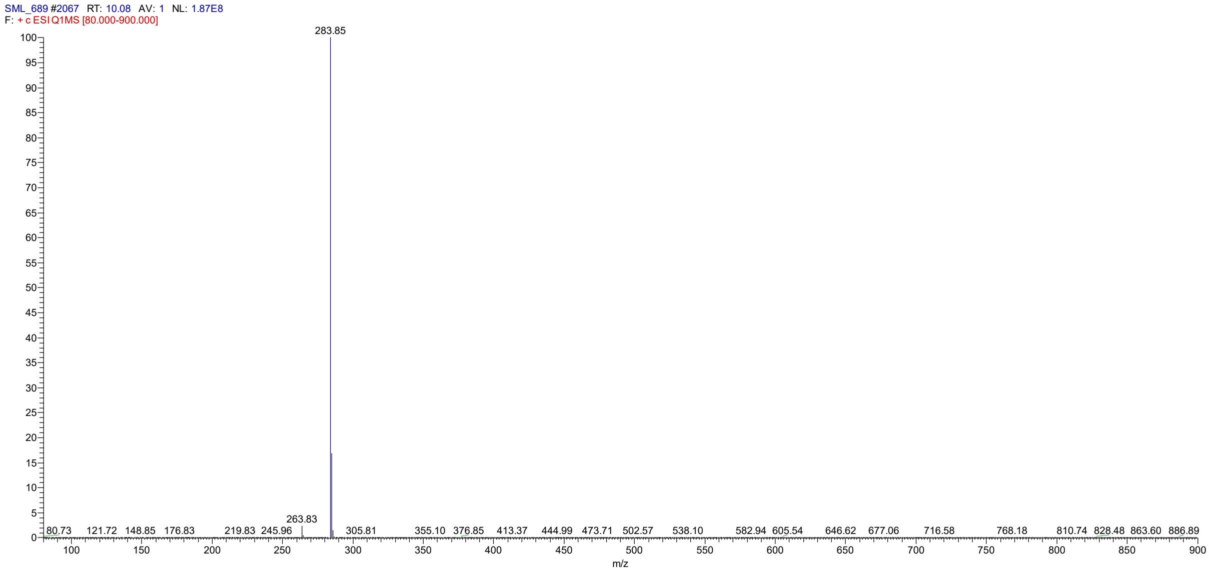

Figure S6c: MS spectrum of (3f).

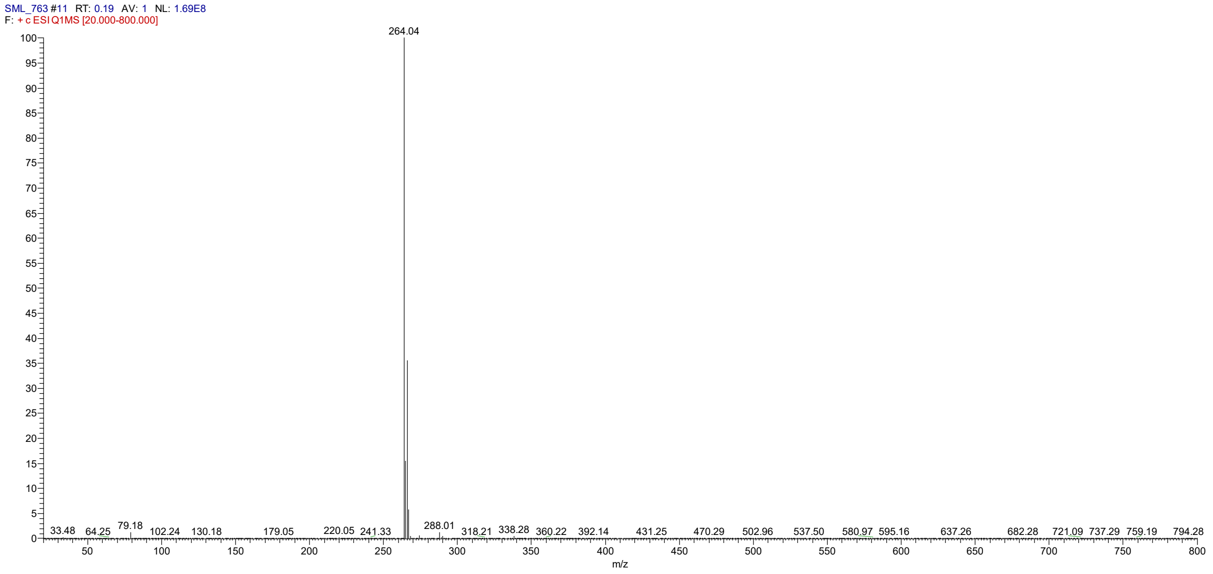

Figure S7c: MS spectrum of (3g).

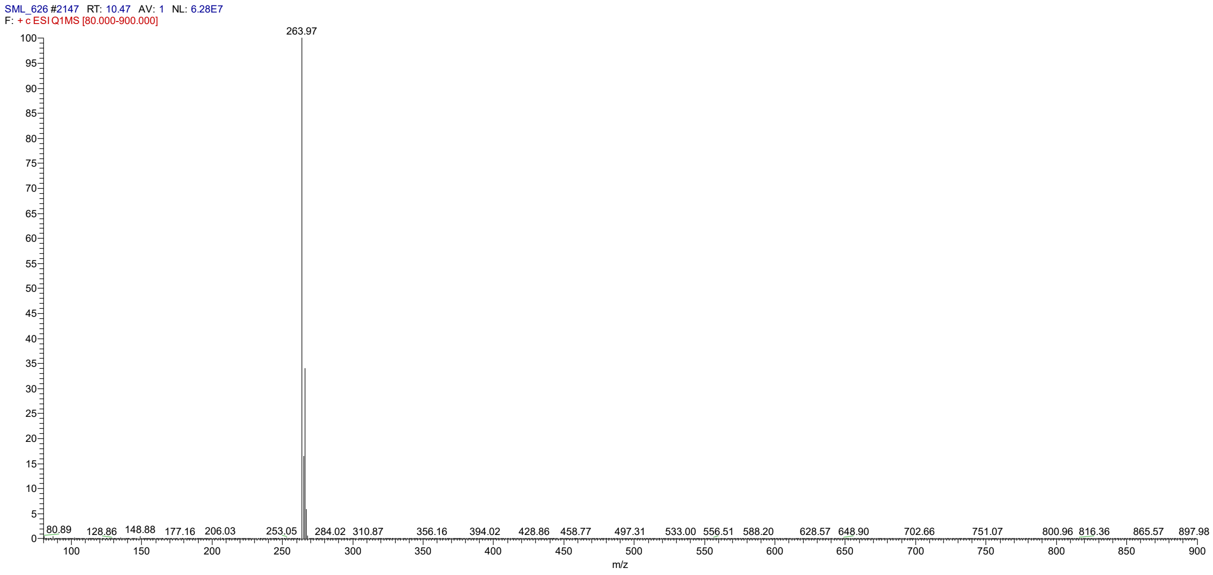

Figure S8c: MS spectrum of (3h).

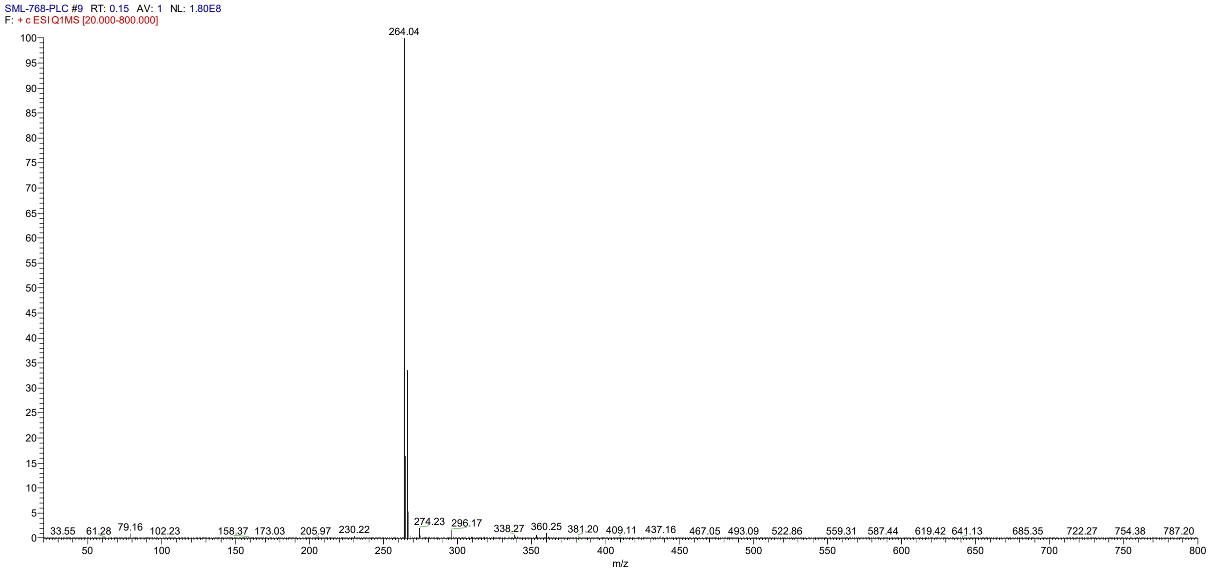

Figure S9c: MS spectrum of (3i).

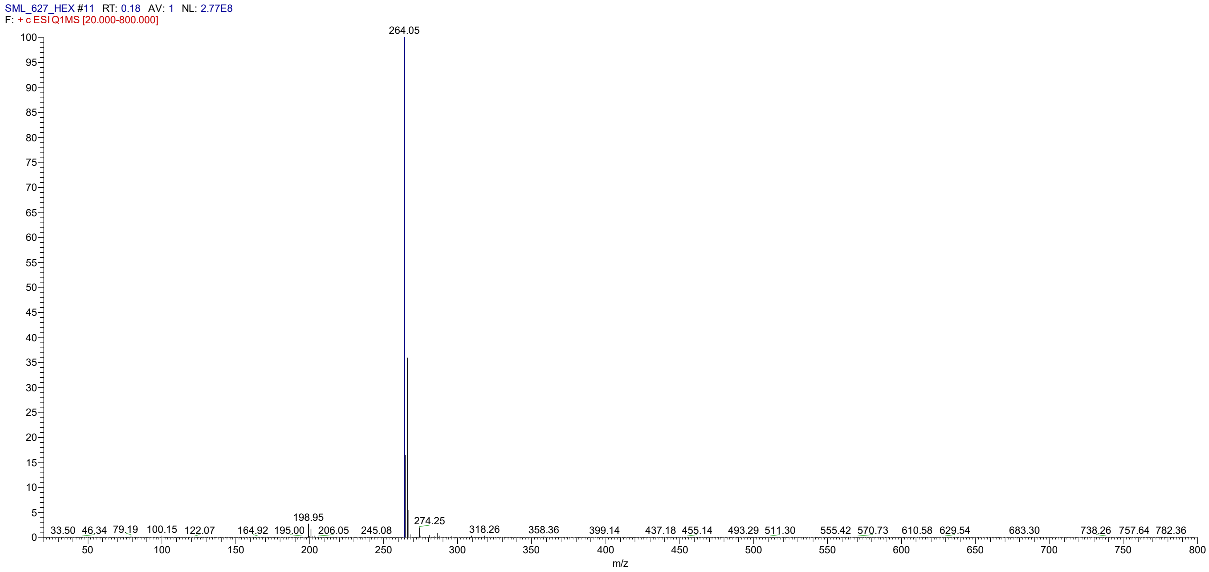

Figure S10c: MS spectrum of (3j).

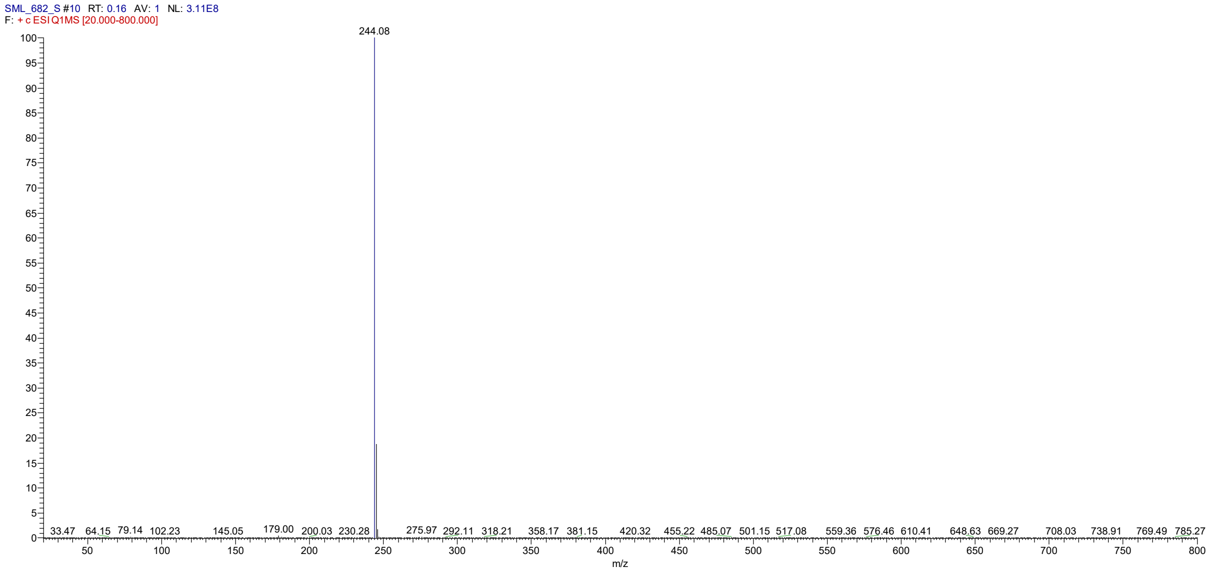

Figure S11c: MS spectrum of (3k).

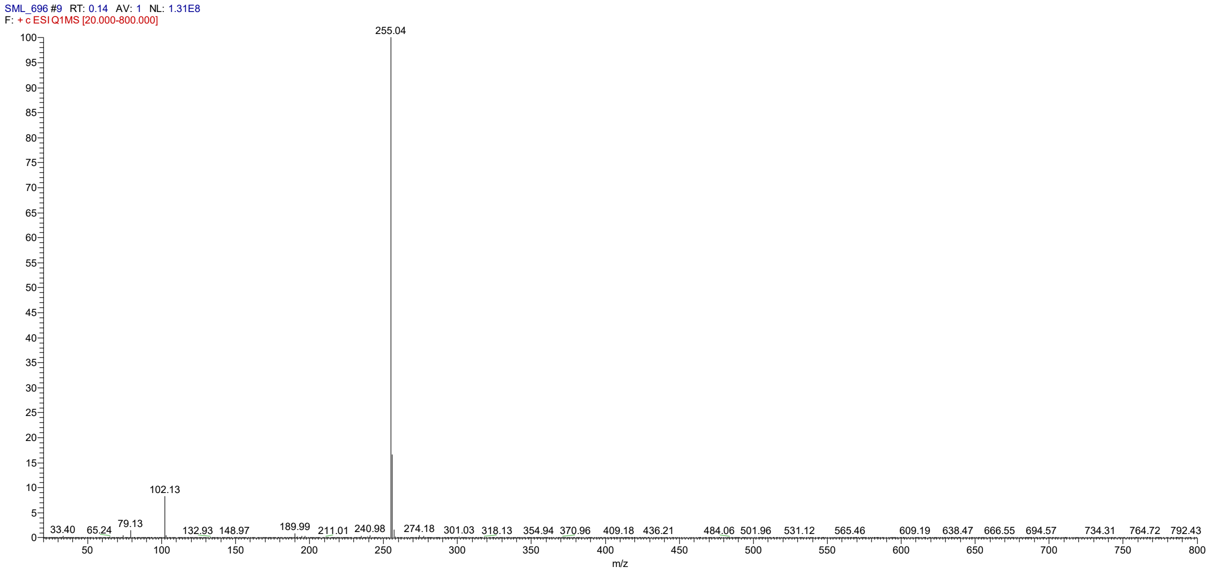

Figure S12c: MS spectrum of (3l).

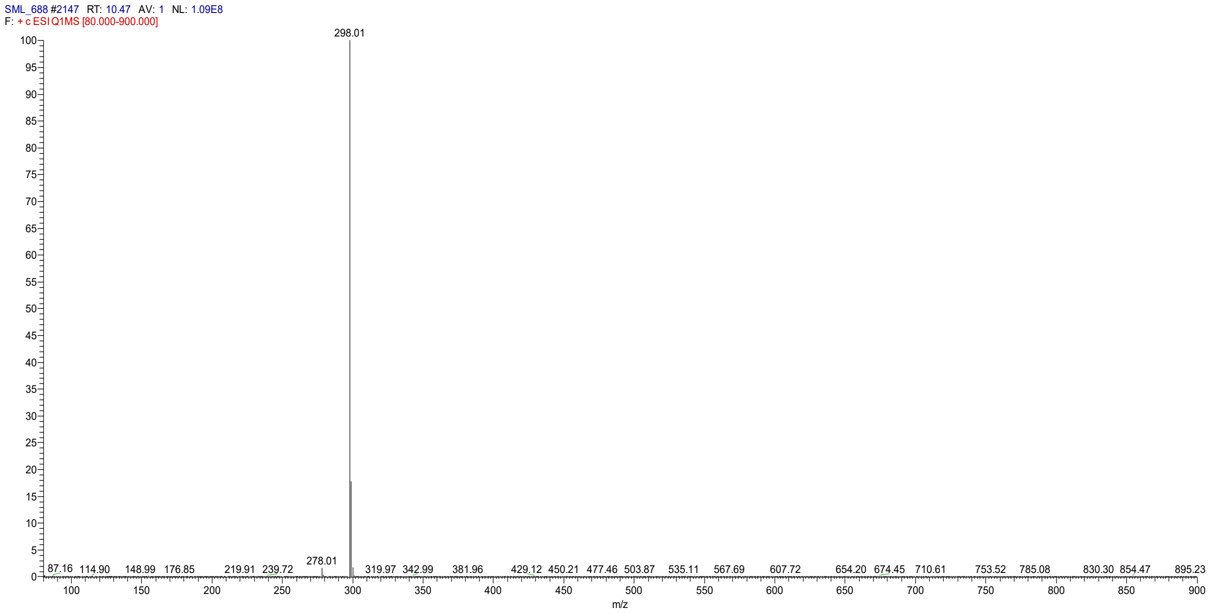

Figure S13c: MS spectrum of (3m).

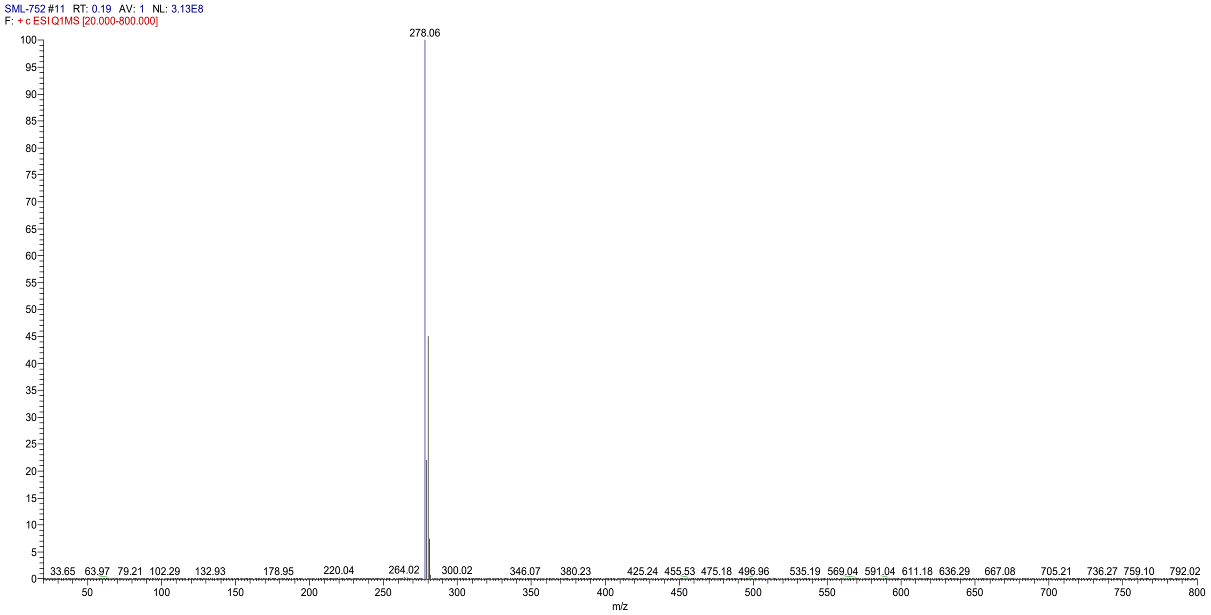

Figure S14c: MS spectrum of (3n).

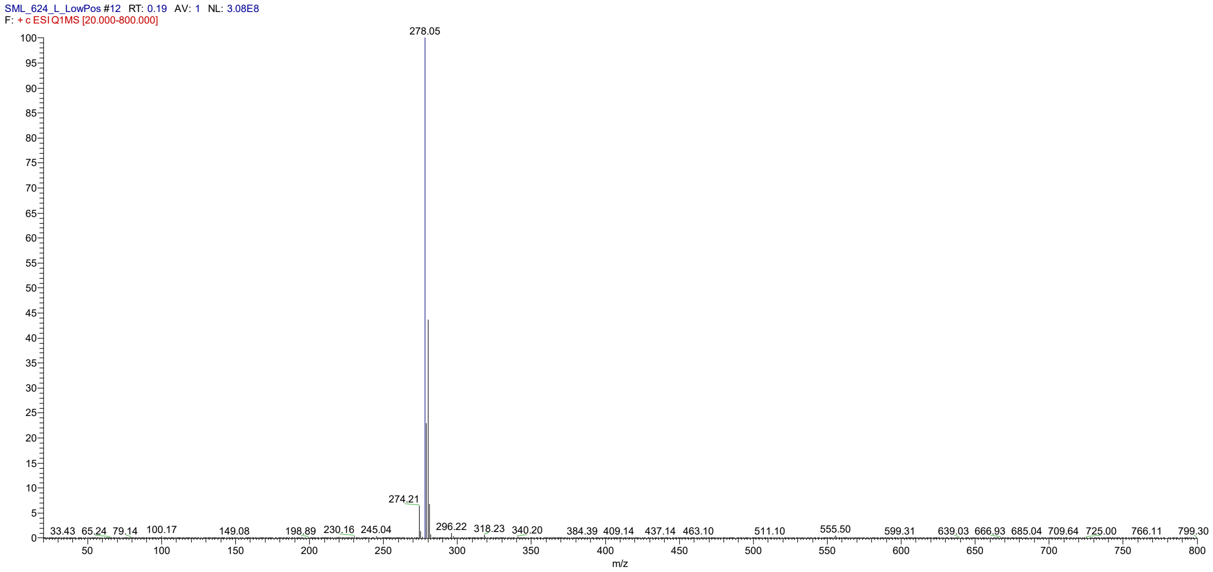

Figure S15c: MS spectrum of (3o).

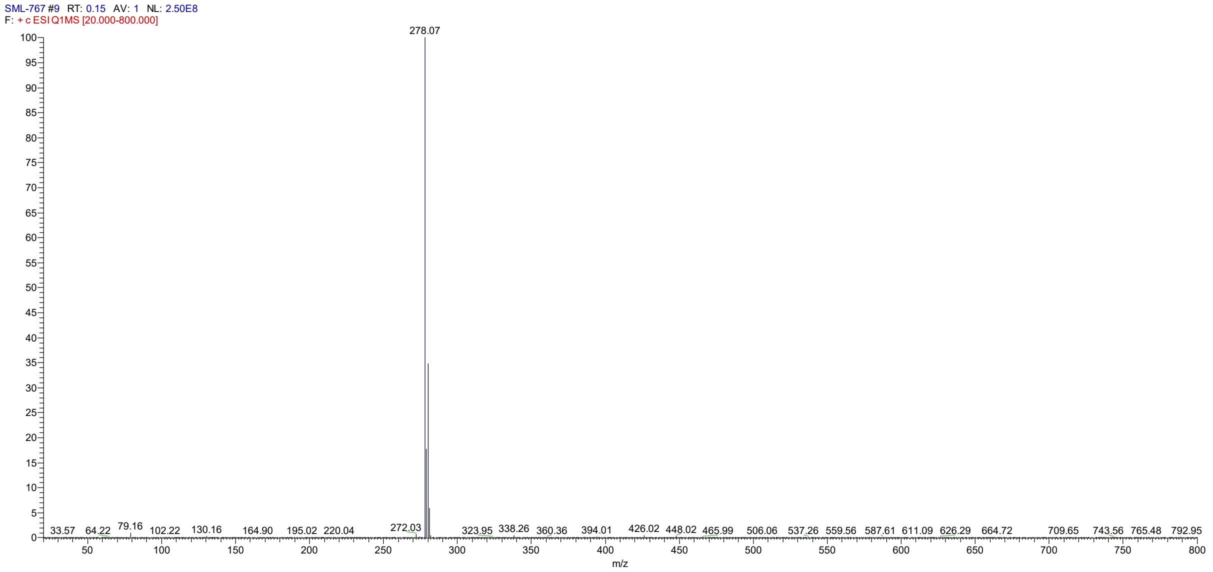

Figure S16c: MS spectrum of (3p).

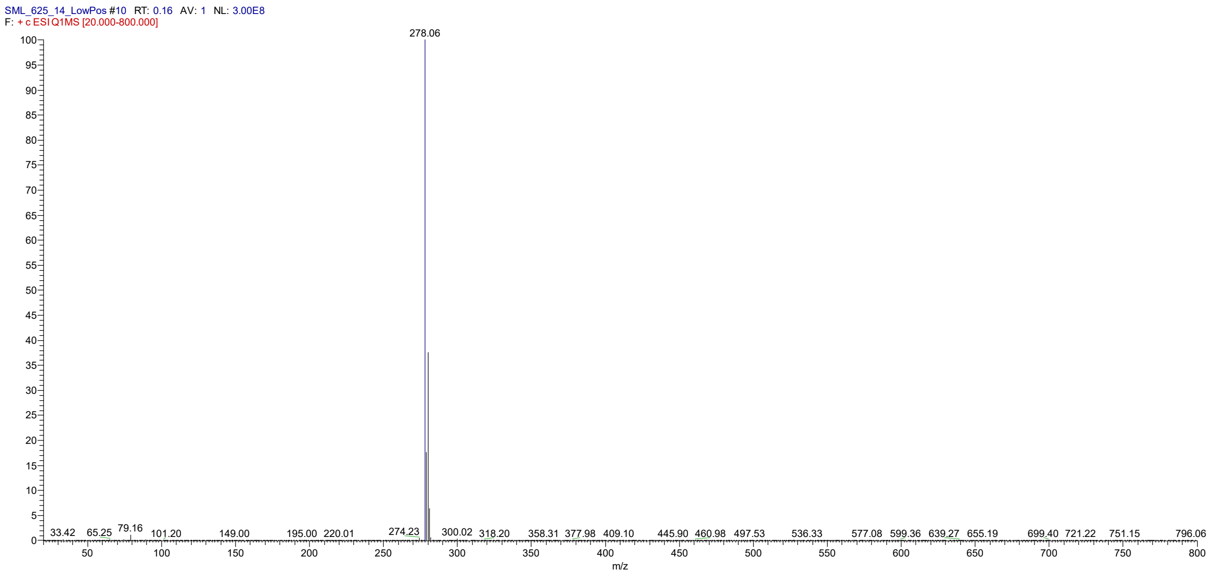

Figure S17c: MS spectrum of (3q).

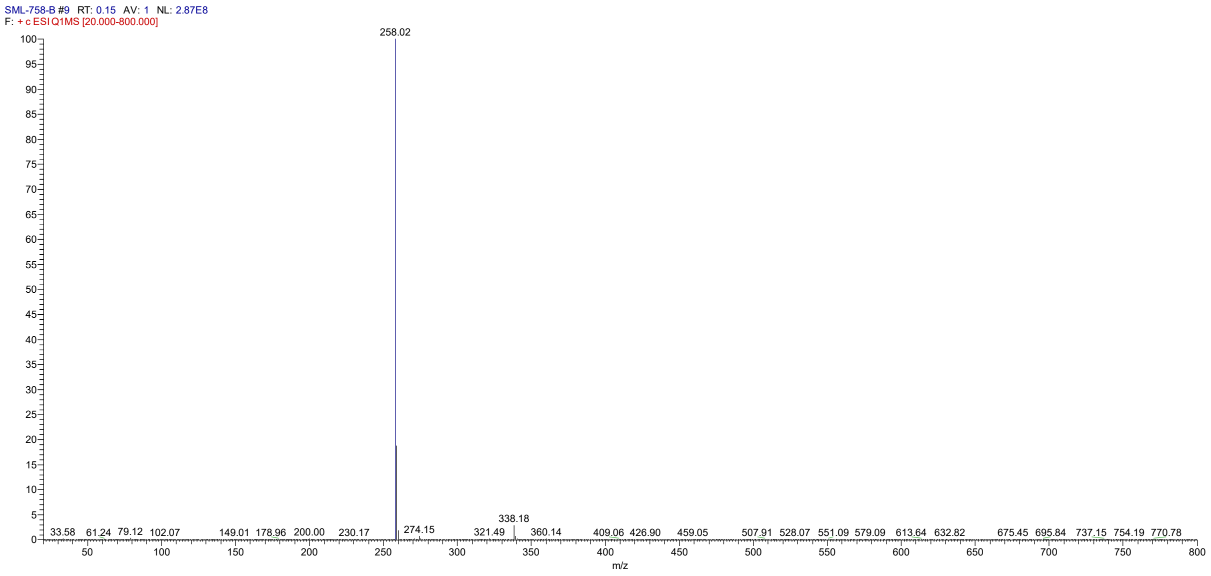

Figure S18c: MS spectrum of (3r).

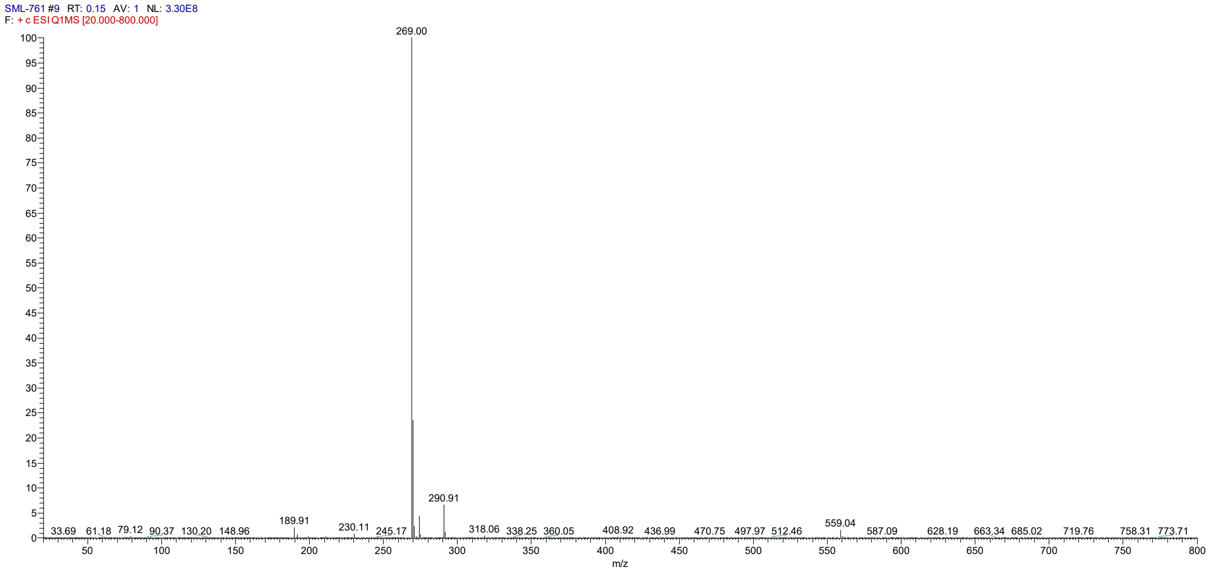

Figure S19c: MS spectrum of (3s).

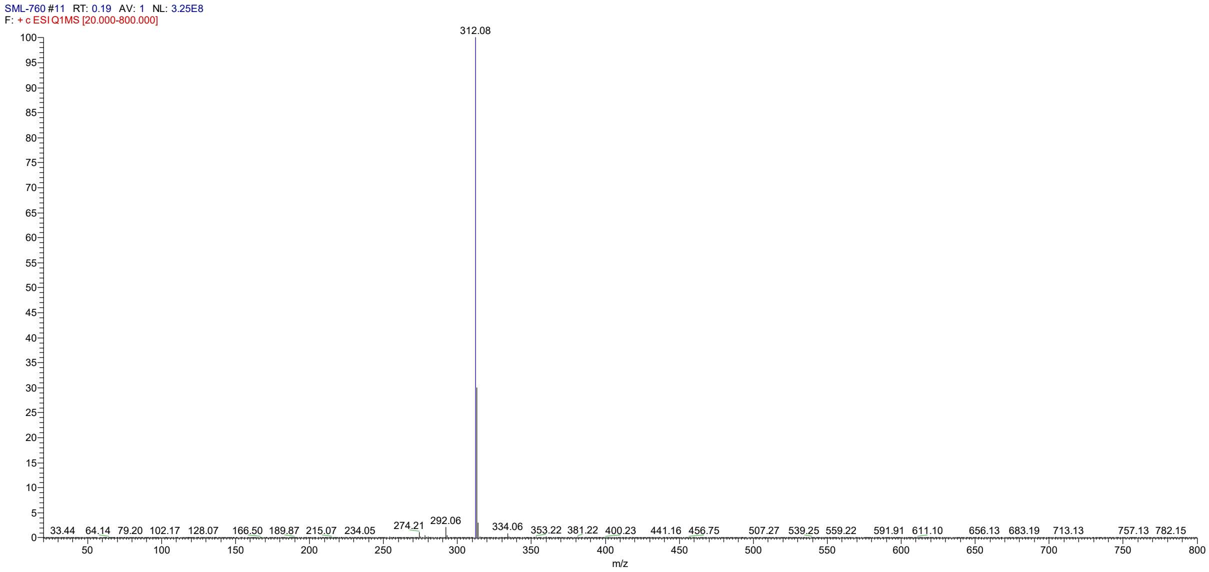

Figure S20c: MS spectrum of (3t).

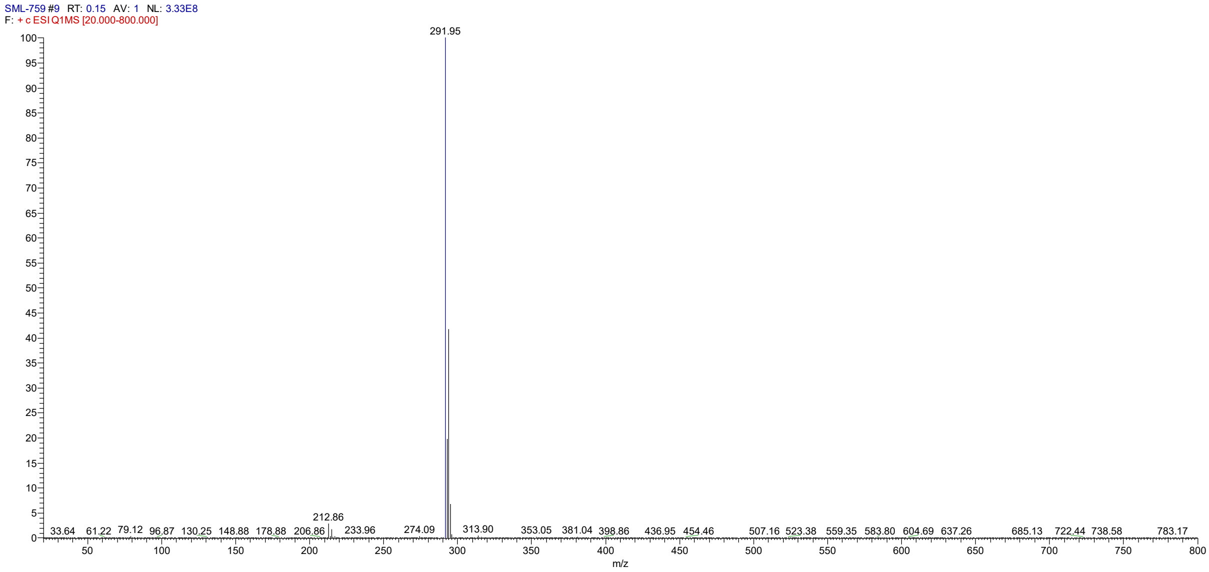

Figure S21c: MS spectrum of (3u).

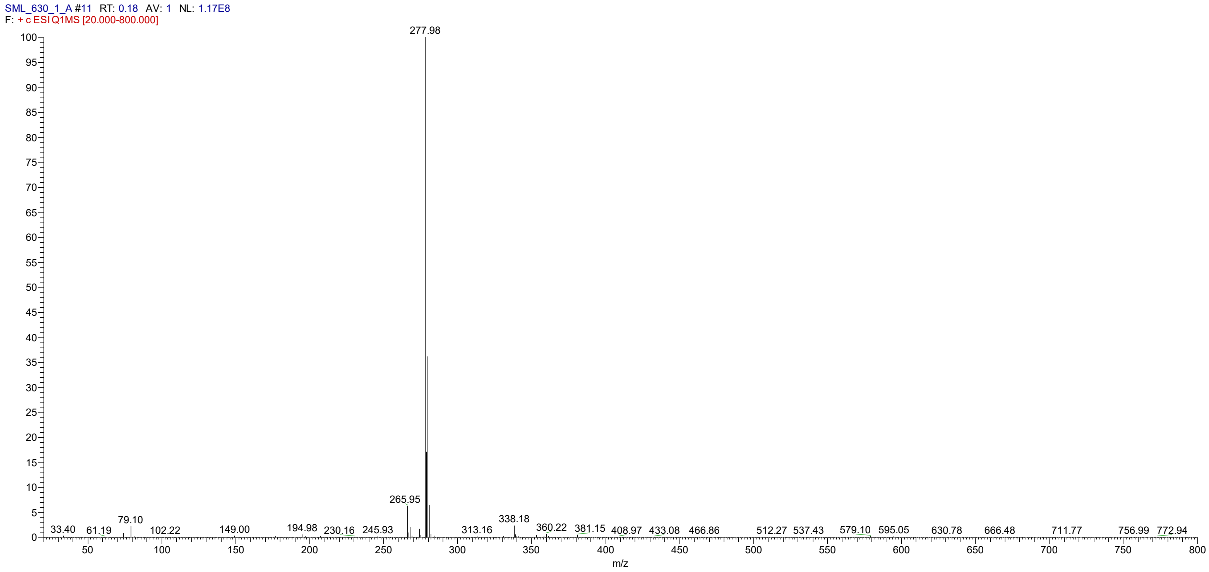

Figure S22c: MS spectrum of (4a).

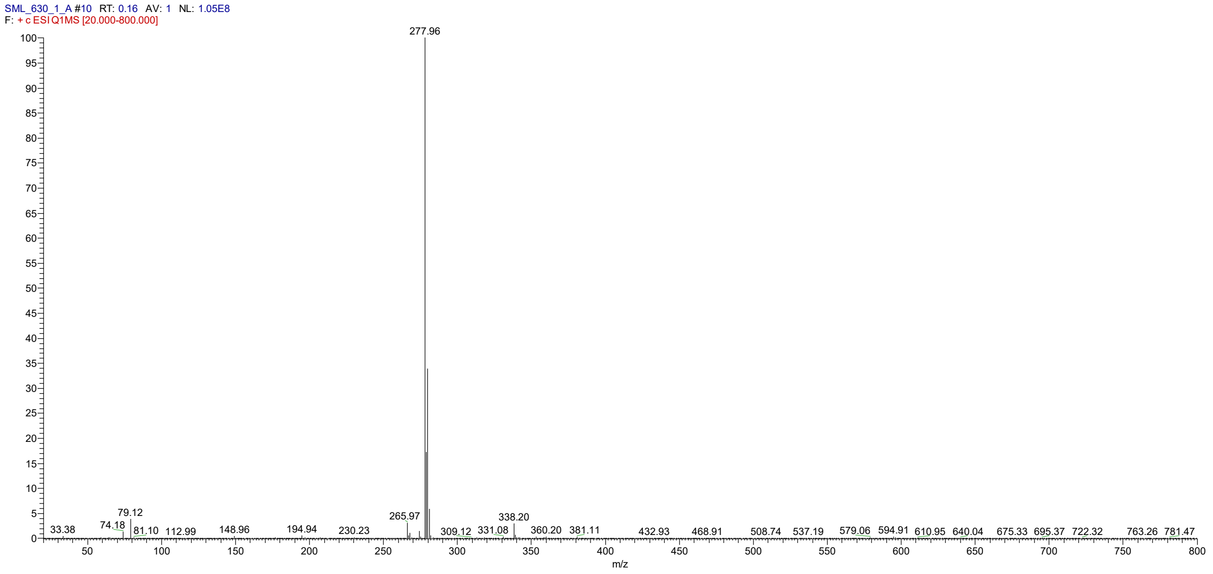

Figure S23c: MS spectrum of (4b).

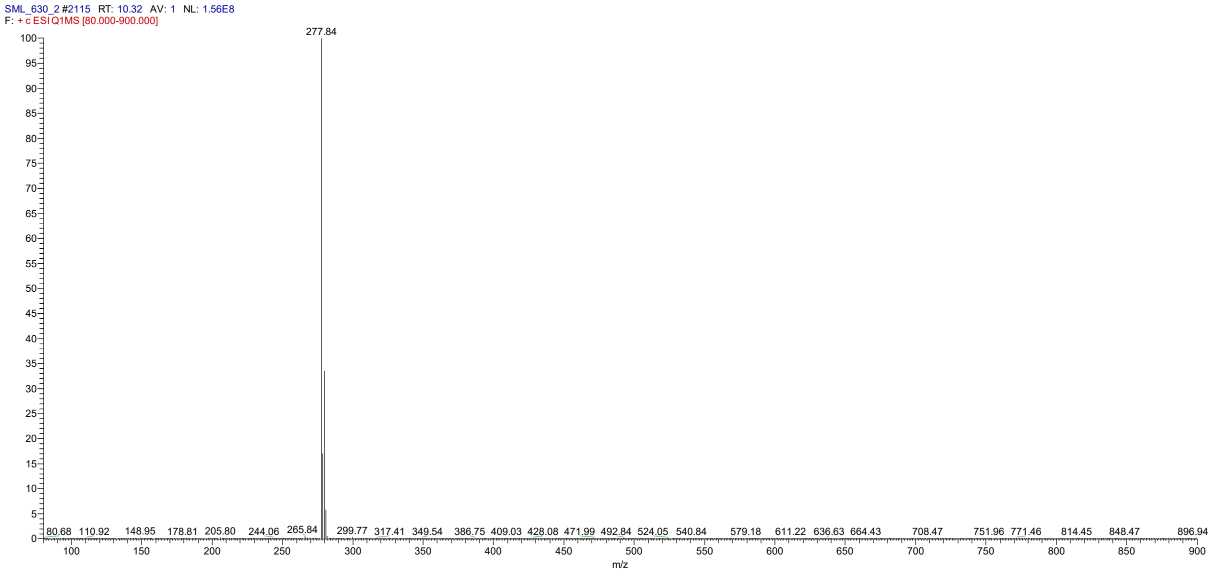

Figure S24c: MS spectrum of (4c).

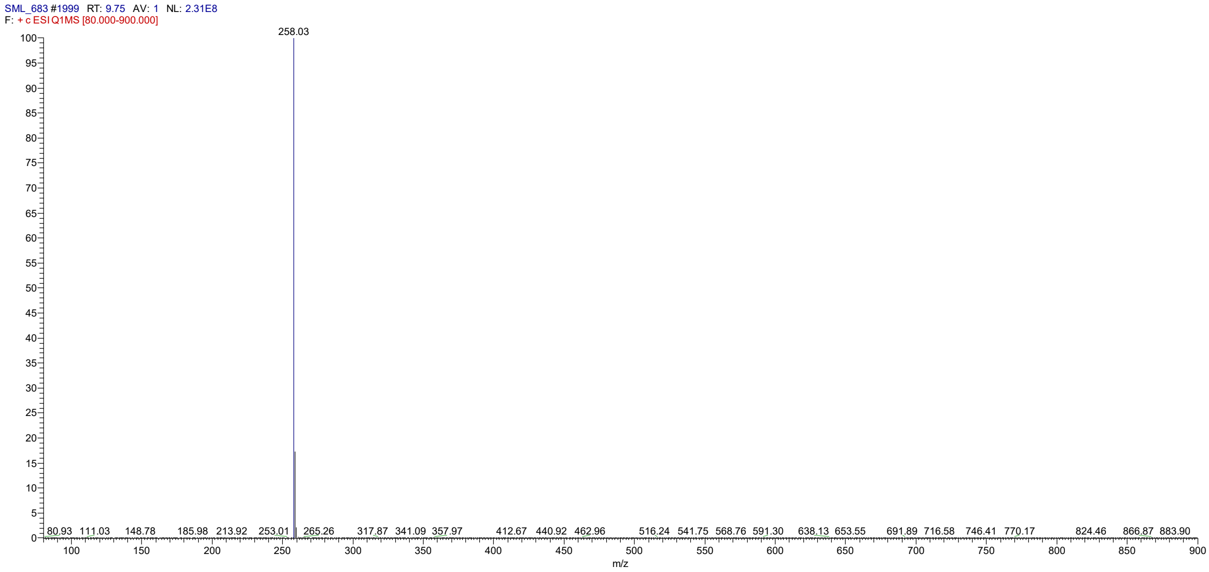

Figure S25c: MS spectrum of (4d).

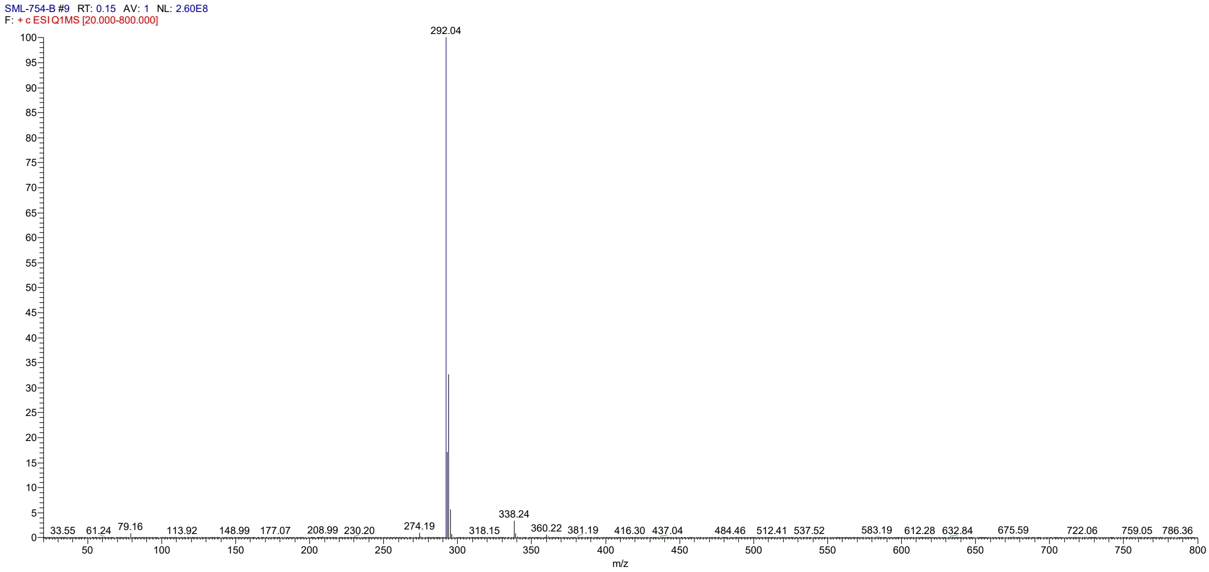

Figure S26c: MS spectrum of (4e).

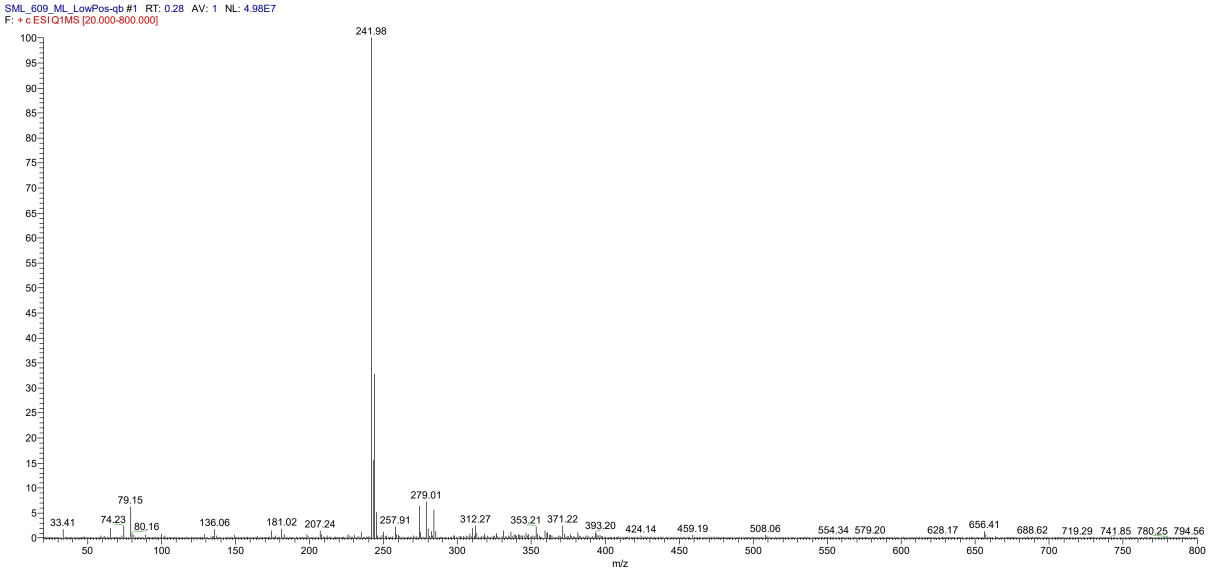

Figure S27c: MS spectrum of (6a).

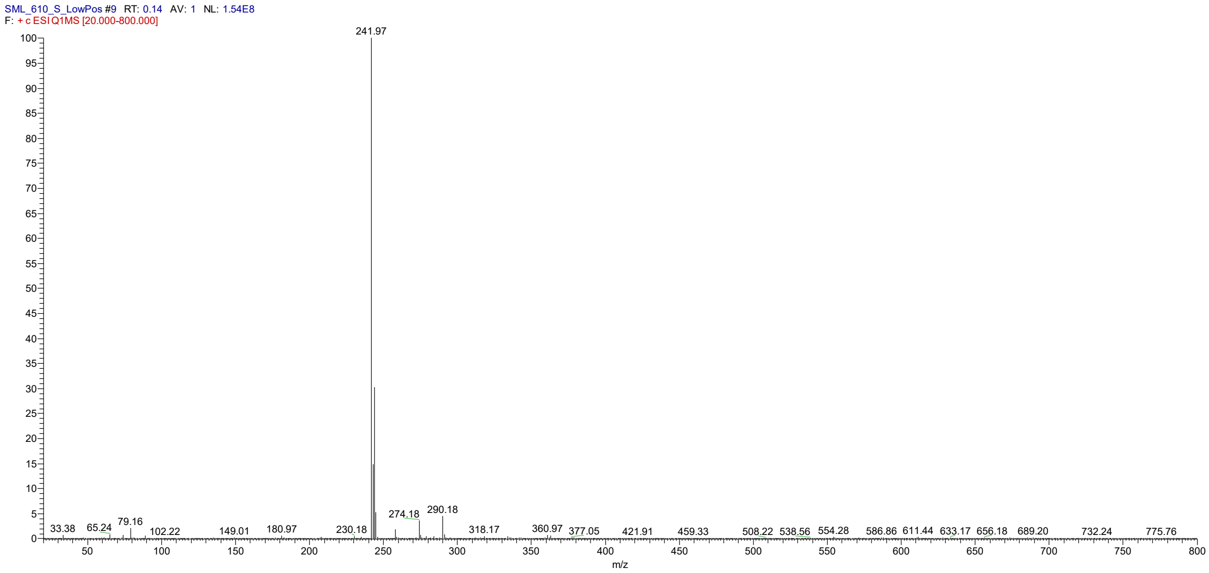

Figure S28c: MS spectrum of (6b).

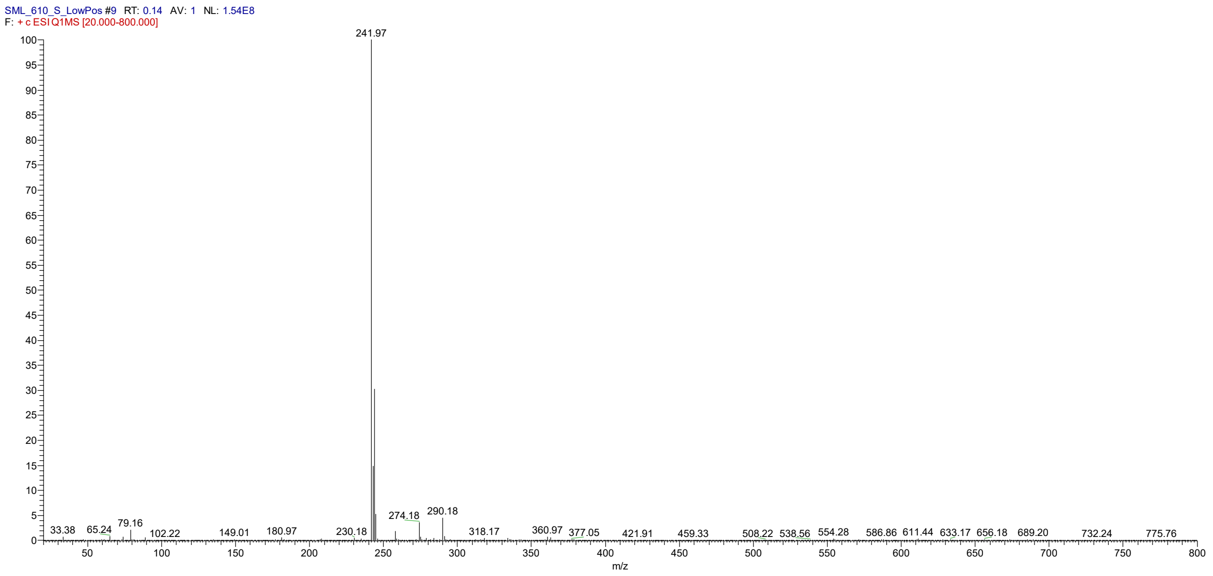

Figure S29c: MS spectrum of (6c).

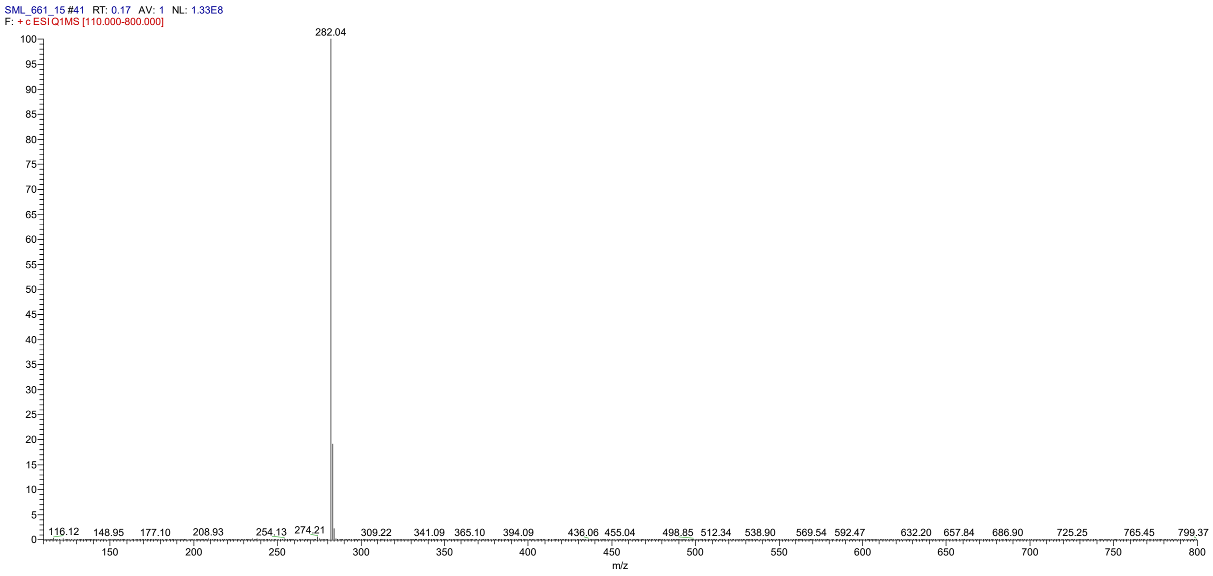

Figure S30c: MS spectrum of (8a).

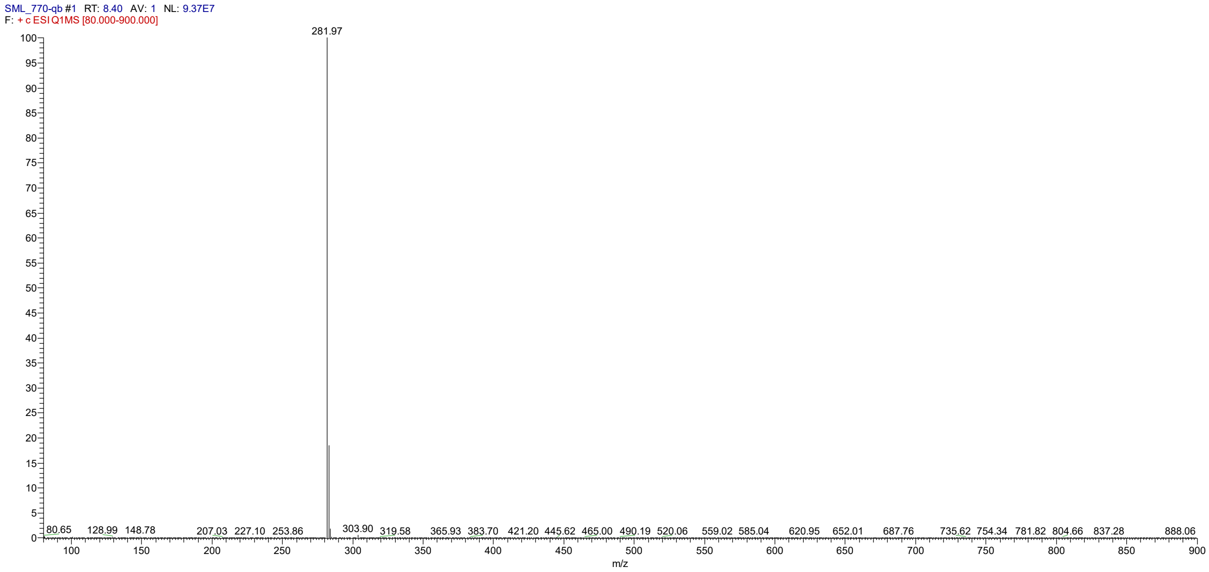

Figure S31c: MS spectrum of (8b).

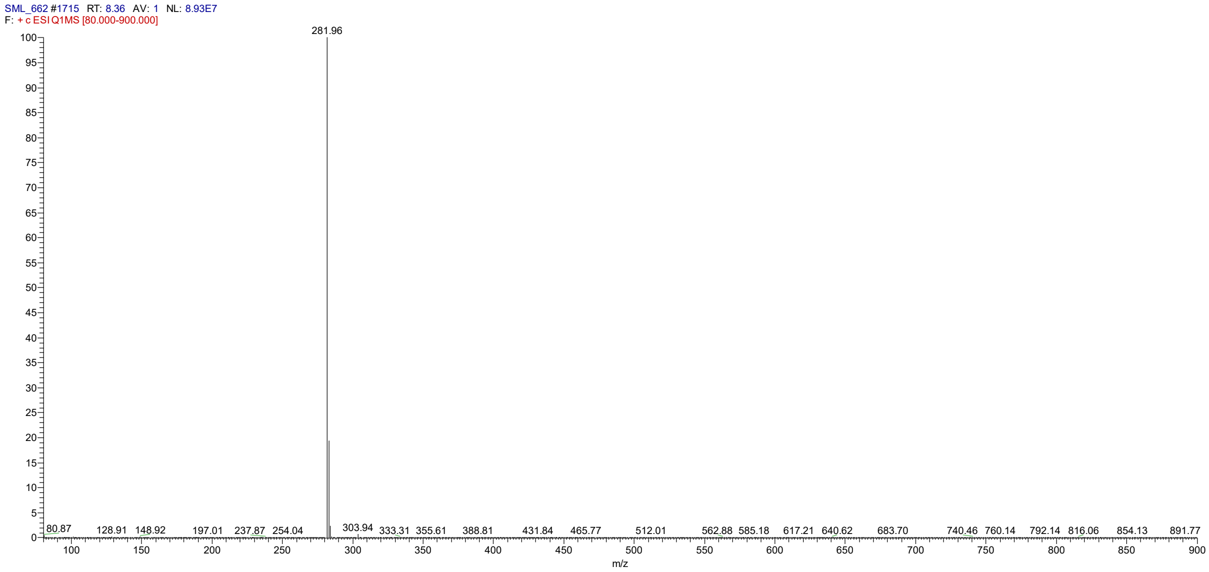

Figure S32c: MS spectrum of (8c).

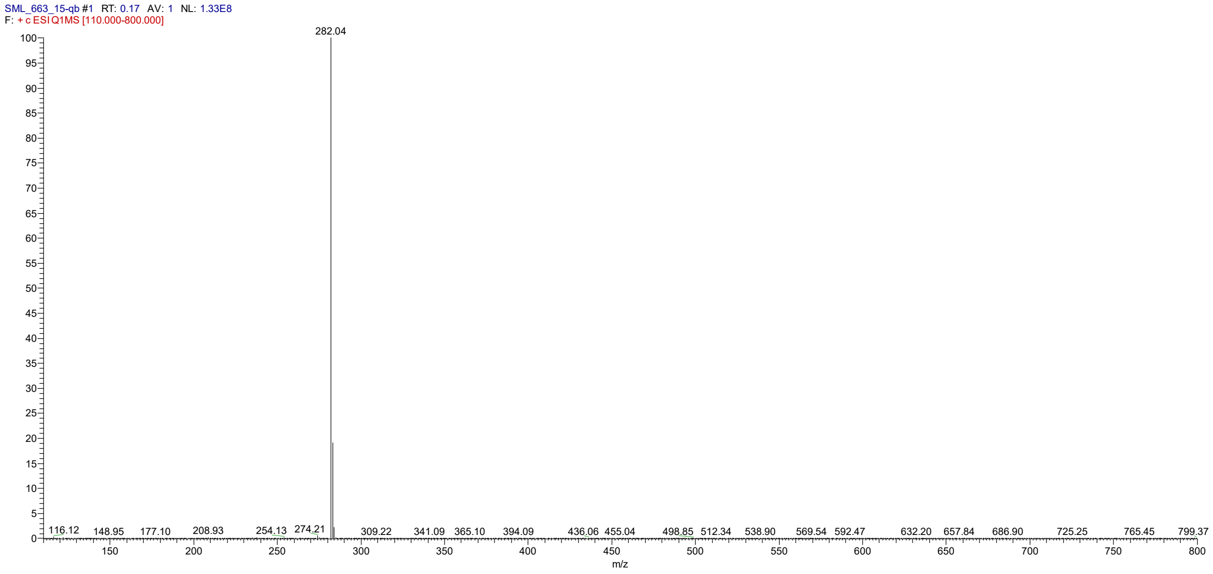

Figure S33c: MS spectrum of (8d).

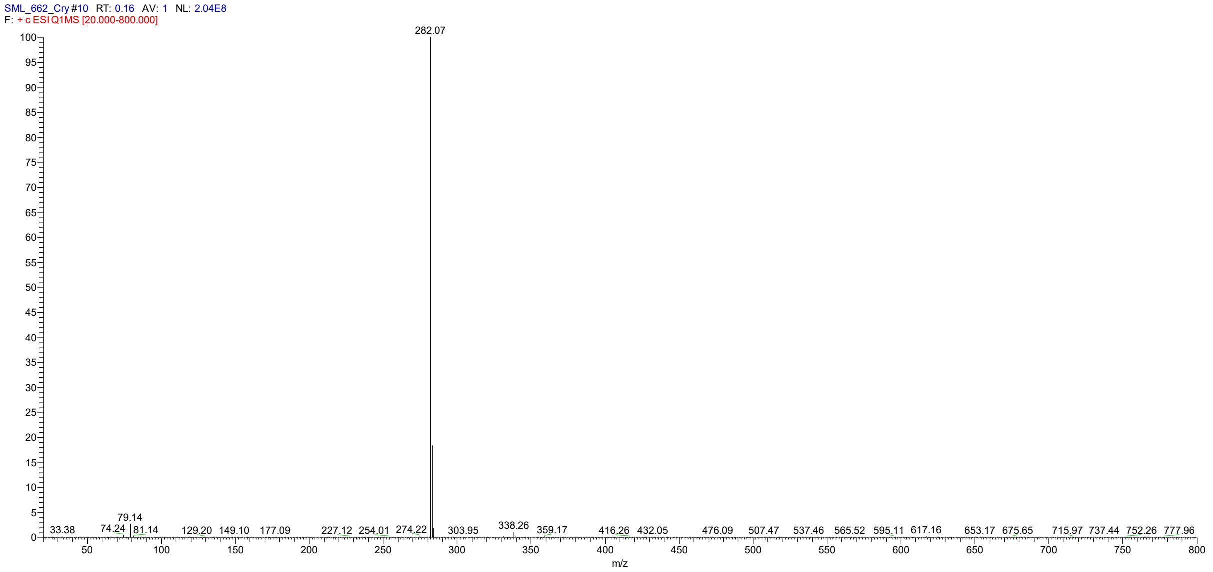

Figure S34c: MS spectrum of (8e).

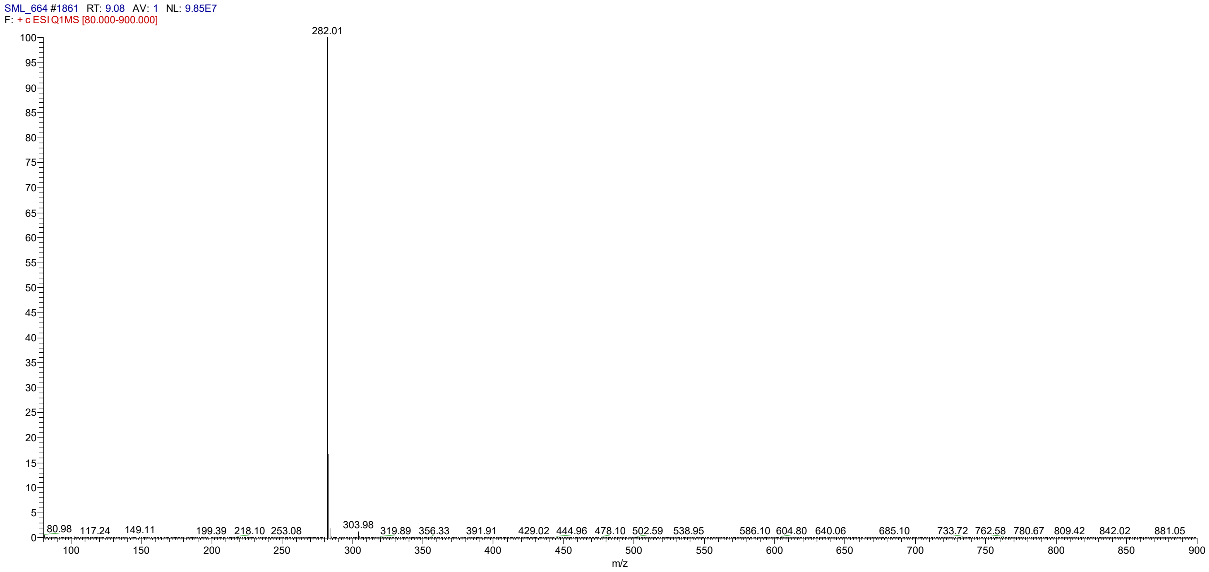

Figure S35c: MS spectrum of (8f).

Computational Data:

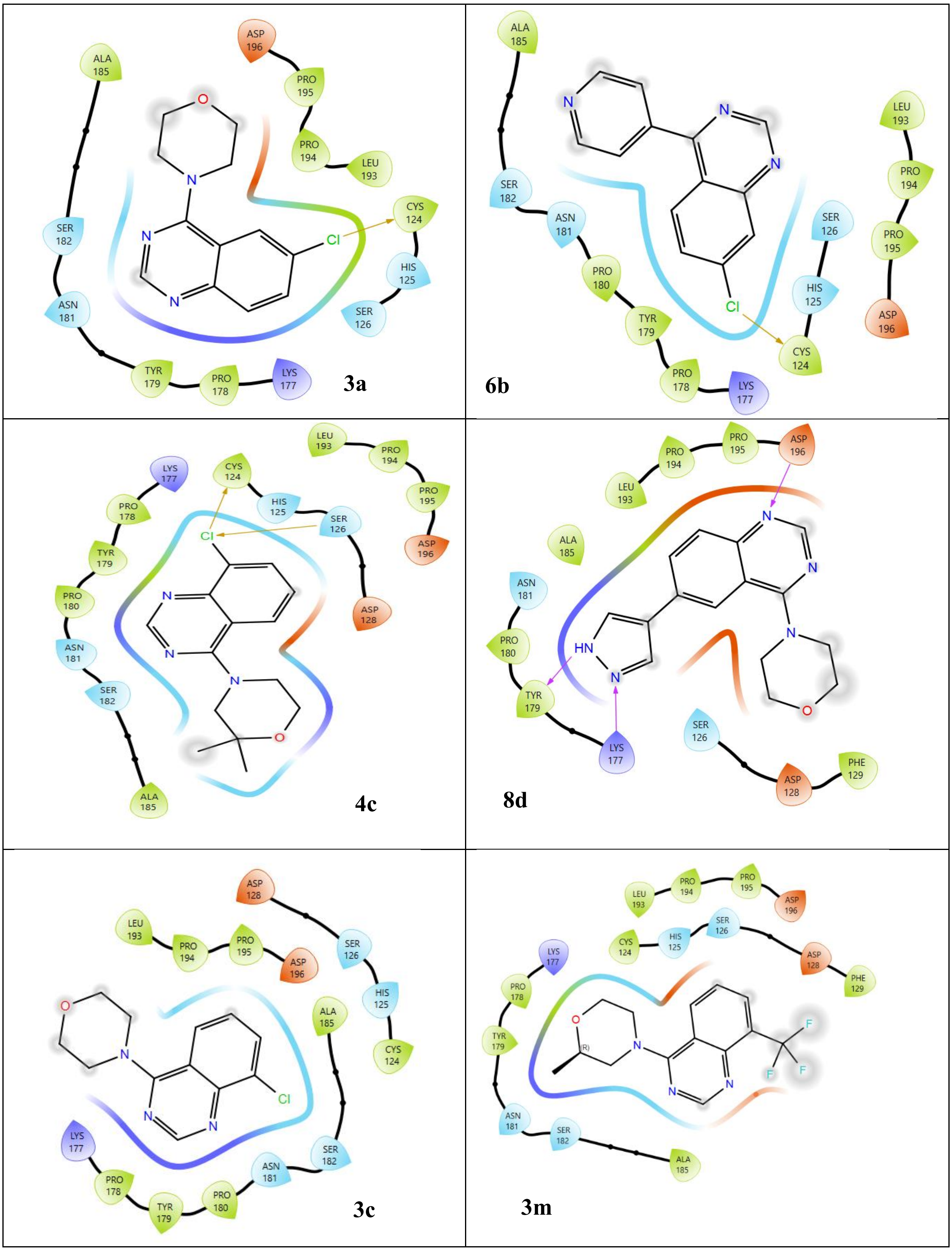

Figure S1d: 2D representation of the binding interactions between compounds 3a, 6b, 4c, 8d, 3c, and 3m of the ML-167 derivatives with Cyc1.

Table S1: Details of biological activity, docking scores, ligand efficiency, and key hydrophobic and hydrophilic interactions of ML-167 derivatives.

**Codes**	Cell Proliferation (%)	**Docking Score**	**glide ligand efficiency**	**H-bond/Halogen**	**Hydrophobic**
**(3a)**	115	-4.515	-0.266	Cys124(halogen)	His125, Ser126, Pro178, Tyr179, Leu193
**(3b)**	94	-4.965	-0.292	Tyr174	Ser126, Lys177, Pro178, Tyr179
**(3c)**	111	-4.300	-0.253		His125, Ser126, Pro178, Tyr179
**(6a)**	94	-5.274	-0.310	Lys118 (halogen)	His125, Ser126, Pro178, Tyr179
**(6b)**	116	-4.298	-0.253	Cys124(halogen)	Cys124, His125, Ser126, Pro178, Tyr179
**(6c)**	87	-4.098	-0.241		His125, Ser126, Pro178, Tyr179
**(3o)**	84	-4.590	-0.242	Cys124(halogen)	His125, Ser126, Pro178, Tyr179
**(3q)**	106	-4.815	-0.253		His125, Ser126, Pro178, Tyr179
**(3h)**	98	-4.712	-0.262		His125, Ser126, Lys177, Pro178, Tyr179, Leu193
**(3j)**	103	-4.835	-0.269		His125, Ser126, Pro178, Tyr179
**(4a)**	111	-4.669	-0.246	Tyr132	His125, Ser126, Pro178, Tyr179
**(4c)**	112	-4.196	-0.221	Cys124 (halogen)	His125, Ser126, Lys177, Pro178, Tyr179
**(8a)**	103	-5.605	-0.267	Lys177, Pro194	His125, Ser126, Pro178, Tyr179
**(8c)**	90	-5.443	-0.259	Phe157	His125, Ser126, Pro178, Tyr179
**(8d)**	114	-5.358	-0.255	Lys177, Tyr179, Asp196	His125, Ser126, Lys177, Pro178, Tyr179
**(8f)**	107	-4.983	-0.237	Cys124	His125, Ser126, Lys177, Pro178, Tyr179
**(3d)**	104				His125, Ser126, Pro178, Tyr179
**(3k)**	109	-4.632	-0.257		His125, Ser126, Pro178, Tyr179
**(4d)**	99	-4.222	-0.222		His125, Ser126, Pro178, Tyr179, Leu193
**(3m)**	112	-4.740	-0.226		His125, Ser126, Pro178, Tyr179
**(3f)**	103	-4.618	-0.231		His125, Ser126, Pro178, Tyr179
**(3l)**	83	-4.666	-0.246		His125, Ser126, Pro178, Tyr179
**(3n)**	91	-4.152	-0.219		His125, Ser126, Pro178, Tyr179
**(4e)**	99	-3.575	-0.179	Tyr179(halogen)	His125, Ser126, Pro178, Tyr179
**(3r)**	93	-4.882	-0.257	Lys177	His125, Ser126, Lys177, Pro178, Tyr179
**(3u)**	90	-4.501	-0.225		His125, Ser126, Pro178, Tyr179, Leu193
**(3t)**	89	-4.696	-0.213		His125, Ser126, Pro178, Tyr179
**(3s)**	97	-5.144	-0.257		His125, Ser126, Pro178, Tyr179, Leu193
**(3e)**	96	-4.677	-0.260		His125, Ser126, Pro178, Tyr179
**(3g)**	91	-4.727	-0.263		His125, Ser126, Pro178, Tyr179
**(3p)**	91	-4.815	-0.253		His125, Ser126, Pro178, Tyr179
**(3i)**	92	-4.649	-0.258		His125, Ser126, Pro178, Tyr179
**(4b)**	94	-4.669	-0.246	Cys124 (halogen)	His125, Ser126, Pro178, Tyr179

**(8b)**	95	-4.834	-0.230	Pro194	His125, Ser126, Pro178, Tyr179
**(8e)**	96	-5.175	-0.246		His125, Ser126, Pro178, Tyr179

Physicochemical properties:

Table S2. Physicochemical parameters of the tested synthesized ML-167 derivatives.

Entry	MW (g/mol)	Solubility	LogD	TPSA (Å²)
Metformin	129.16	299.14	< −2.41	91.49
ML-167	335.36	374	1.03	84.32
**(3a)**	249.70	316.20	2.11	38.25
**(3b)**	249.70	301.66	2.15	38.25
**(3c)**	249.70	148.60	1.89	38.25
**(3d)**	229.28	172.61	2.51	38.25
**(3e)**	240.27	190.45	0.91	62.04
**(3f)**	283.25	205.31	2.24	38.25
**(3g)**	263.73	241.44	2.20	38.25
**(3h)**	261.73	218.43	2.60	38.25
**(3i)**	263.73	273.85	2.00	38.25
**(3j)**	263.73	220.42	2.24	38.25
**(3k)**	243.31	244.09	2.31	38.25
**(3l)**	254.29	182.23	1.29	62.04
**(3m)**	297.28	184.03	2.40	38.25
**(3n)**	277.75	221.58	2.67	38.25
**(3o)**	277.75	193.04	2.78	38.25
**(3p)**	277.75	267.73	2.85	38.25
**(3q)**	277.75	260.64	2.48	38.25
**(3r)**	358.34	254.73	2.86	38.25
**(3s)**	268.32	159.72	1.46	62.04
**(3t)**	311.31	151.40	2.63	38.25
**(3u)**	291.78	195.97	2.78	38.25
**(4a)**	277.70	146.42	2.94	38.25

**(4b)**	277.70	257.12	2.84	38.25
**(4c)**	277.70	170.89	2.42	38.25
**(4d)**	257.34	198.58	2.68	38.25
**(4e)**	291.78	143.75	2.93	38.25
**(6a)**	241.68	149.56	2.17	38.25
**(6b)**	241.68	309.93	2.14	38.25
**(6c)**	241.68	157.56	2.15	38.25
**(8a)**	281.32	147.46	2.11	38.25
**(8b)**	281.32	240.72	2.04	38.25
**(8c)**	281.32	249.22	2.01	38.25
**(8d)**	281.32	222.38	1.87	38.25
**(8e)**	281.32	260.66	2.04	38.25
**(8f)**	281.32	105.66	2.16	38.25

In vivo Data:

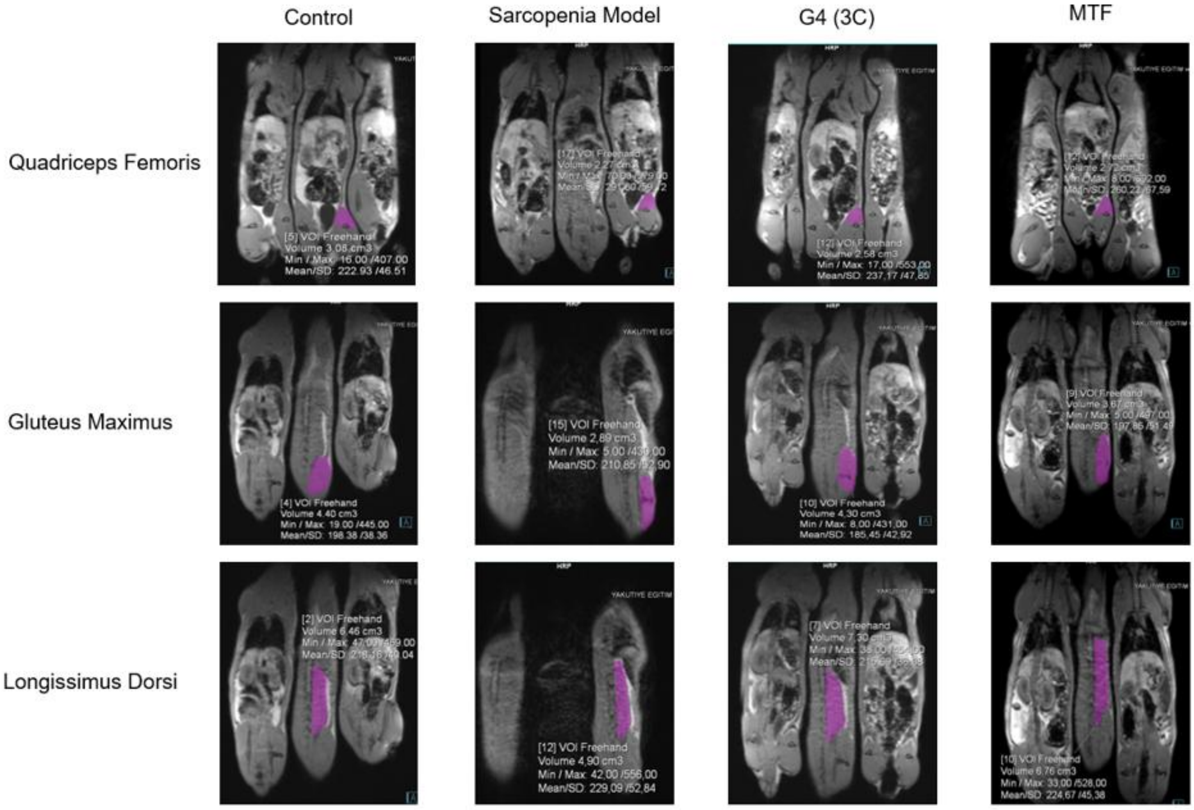

Figure S1e. Representative MRI images of skeletal muscles in experimental groups. Magnetic resonance imaging (MRI) of the quadriceps femoris, gluteus maximus, and longissimus dorsi muscles in the control, disease model, G4 (3c), and metformin (MTF) groups. Highlighted regions indicate the analysed muscle areas used for volumetric and signal intensity assessments, illustrating treatment-related changes in muscle morphology.

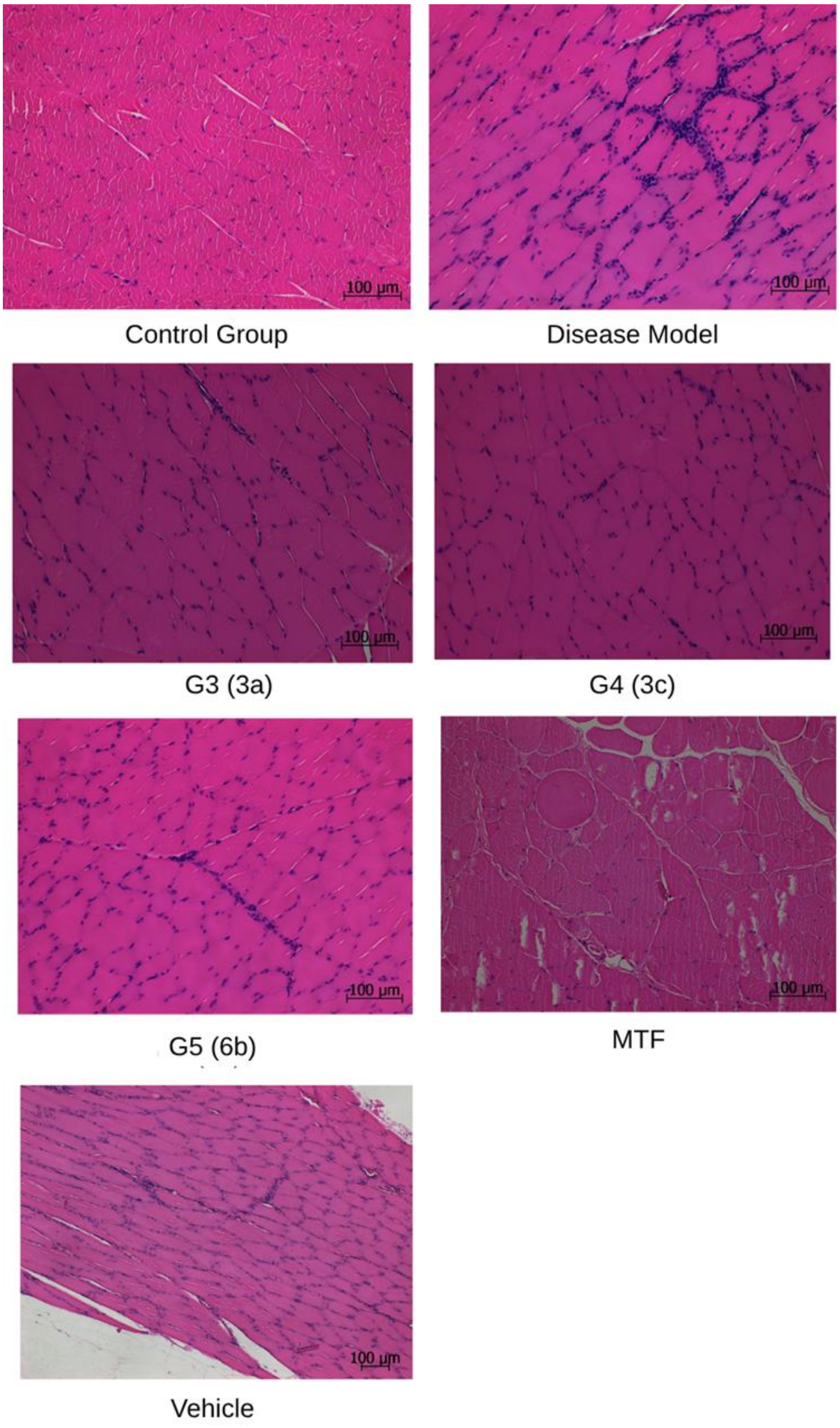

Figure S2e. Representative histopathological images of skeletal muscle tissues in the experimental groups. Sections from the Control, Disease model, G3 (3a), G4 (3c), G5 (6b), Metformin (MTF), and Vehicle groups demonstrate treatment-related atrophy and morphological changes.

Table S3. The Right and left skeletal muscle mass (mean ± SD) of the Gluteus Maximus, Quadriceps Femoris, and Longissimus Dorsi in experimental groups.

Groups	Mean Muscle Volumes (as cm^3)					
	GM Right (Mean ± SD)	GM Left (Mean ± SD)	QF Right (Mean ± SD)	QF Left (Mean ± SD)	LD Right (Mean ± SD)	LD Left (Mean ± SD)
**G1 Control**	4.57 ± 1.09	4.12 ± 0.25	3.12 ± 0.16	2.99 ± 0.42	5.93 ± 0.93	5.61 ± 0.77
**G2 SHAM**	3.91 ± 0.91	3.83 ± 1.04	3.21 ± 0.53	2.60 ± 0.29	5.61 ± 0.26	5.24 ± 0.39
**G3 (3a)**	4.46 ± 0.41	5.11 ± 0.73	3.03 ± 0.43	2.89 ± 0.30	5.67 ± 1.03	5.82 ± 1.56
**G4 (3c)**	4.62 ± 0.26	4.22 ± 0.15	3.07 ± 0.27	3.00 ± 0.55	6.58 ± 0.55	6.36 ± 0.82
**G5 (6b)**	5.43 ± 0.61	5.06 ± 0.40	3.34 ± 0.42	3.31 ± 0.71	6.83 ± 0.52	6.55 ± 0.49
**MTF**	5.12 ± 0.49	4.41 ± 1.04	3.10 ± 0.39	3.15 ± 0.38	7.51 ± 0.55	6.77 ± 0.56
**Vehicle**	4.39 ± 0.28	3.88 ± 0.34	2.46 ± 0.36	2.56 ± 0.07	5.27 ± 0.26	5.22 ± 0.38

Table S4. The average skeletal muscle mass (mean ± SD) of Gluteus Maximus, Quadriceps Femoris, and Longissimus Dorsi in experimental groups.

Groups	Mean Muscle Volumes (as cm^3)		
	Gluteus Maximus (Mean ± SD)	Quadriceps Femoris (Mean ± SD)	Longissimus Dorsi (Mean ± SD)
**G1 Control**	4.34 ± 0.67	3.06 ± 0.29	5.77 ± 0.85
**G2 SHAM**	3.87 ± 0.93	2.91 ± 0.41	5.43 ± 0.15
**G3 (3a)**	4.78 ± 0.51	2.96 ± 0.15	5.74 ± 1.30
**G4 (3c)**	4.42 ± 0.20	3.04 ± 0.39	6.47 ± 0.68
**G5 (6b)**	5.24 ± 0.47	3.32 ± 0.54	6.69 ± 0.43

**MTF**	4.77 ± 0.64	3.12 ± 0.34	7.14 ± 0.53
**Vehicle**	4.13 ± 0.12	2.51 ± 0.18	5.24 ± 0.32

Table S5. The total skeletal muscle mass (mean ± SD) in experimental groups.

Groups	Total Muscle Mass (Mean ± SD)
**G1 Control**	13.17 ± 1.62
**G2 SHAM**	12.20 ± 1.39
**G3 (3a)**	13.49 ± 1.71
**G4 (3c)**	13.93 ± 0.55
**G5 (6b)**	15.26 ± 0.80
**MTF**	15.03 ± 0.80
**Vehicle**	11.89 ± 0.19

Table S6. Combined biochemical parameters in experimental groups (Mean ± SD).

Parameter	G1 Control	G2 SHAM	G3 (3a)	G4 (3C)	G5 (6b)	MTF	Vehicle
Urea (mg/dl)	42.32 ± 7.29	43.25 ± 5.16	43.69 ± 3.01	38.46 ± 5.28	41.07 ± 4.71	41.77 ± 2.33	44.17 ± 3.32
BUN	19.78 ± 3.41	20.21 ± 2.41	20.16 ± 1.30	17.97 ± 2.47	19.19 ± 2.20	19.52 ± 1.09	20.64 ± 1.55
Creatinine	0.36 ± 0.06	0.31 ± 0.05	0.31 ± 0.03	0.30 ± 0.05	0.32 ± 0.05	0.32 ± 0.05	0.29 ± 0.04
AST	226.85 ± 62.07	182.49 ± 76.69	202.90 ± 138.60	196.05 ± 99.95	216.86 ± 149.41	216.61 ± 178.76	186.43 ± 56.80
ALT	63.08 ± 23.09	48.54 ± 15.28	50.70 ± 27.98	57.31 ± 21.51	58.06 ± 14.51	57.56 ± 20.91	47.10 ± 11.70
ALP	120.50 ± 44.20	105.00 ± 24.87	122.50 ± 93.11	135.75 ± 53.11	170.75 ± 101.34	144.43 ± 60.51	134.67 ± 48.34
GGT	0.88 ± 0.35	1.46 ± 1.24	1.28 ± 2.04	1.78 ± 2.29	0.78 ± 1.15	0.61 ± 0.48	0.10 ± 0.00
CK	913.29 ± 306.23	966.70 ± 640.07	732.57 ± 429.25	695.43 ± 305.62	679.86 ± 476.23	744.10 ± 902.38	801.33 ± 413.54
CK-MB	1100.49 ± 496.03	1033.81 ± 379.08	1059.80 ± 576.82	913.49 ± 405.13	813.20 ± 309.49	677.09 ± 533.71	496.70 ± 108.68
LDH	1441.25 ± 441.41	1216.85 ± 378.03	1651.61 ± 683.98	1458.64 ± 626.23	1243.45 ± 592.29	1096.18 ± 668.24	651.00 ± 118.50
Glucose	258.00 ± 74.42	271.86 ± 60.22	241.25 ± 79.62	245.88 ± 56.89	222.14 ± 39.83	261.86 ± 44.03	296.67 ± 30.02
Triglycerides	64.88 ± 20.15	96.86 ± 34.54	63.25 ± 25.59	50.00 ± 20.29	48.88 ± 22.60	66.71 ± 26.74	84.00 ± 6.08
Total Cholesterol	77.13 ± 6.92	68.43 ± 13.82	76.63 ± 14.75	77.25 ± 16.64	84.88 ± 21.70	64.71 ± 14.44	55.67 ± 1.53

HDL	50.25 ± 6.86	44.14 ± 7.47	49.38 ± 6.55	47.38 ± 8.31	55.00 ± 12.60	44.17 ± 7.47	36.33 ± 3.79
LDL	16.71 ± 6.10	22.20 ± 4.15	20.00 ± 8.55	20.71 ± 9.79	21.17 ± 8.52	19.67 ± 5.65	17.67 ± 1.15
Total Protein	6.93 ± 0.57	6.67 ± 0.38	6.81 ± 0.32	6.83 ± 0.32	6.98 ± 0.58	6.84 ± 0.28	6.47 ± 0.38
Albumin	3.14 ± 0.31	2.88 ± 0.25	3.21 ± 0.22	2.98 ± 0.19	2.98 ± 0.40	2.89 ± 0.19	2.70 ± 0.10
Total Bilirubin	0.22 ± 0.62*	0.05 ± 0.09	−0.02 ± 0.19	0.06 ± 0.15	0.11 ± 0.04	0.12 ± 0.04	0.08 ± 0.02
Direct Bilirubin	0.05 ± 0.01	0.04 ± 0.02	0.06 ± 0.04	0.04 ± 0.02	0.04 ± 0.01	0.04 ± 0.01	0.04 ± 0.00
Na	139.13 ± 1.36	138.86 ± 1.57	138.50 ± 1.77	138.63 ± 1.77	139.13 ± 1.96	139.43 ± 1.81	137.33 ± 3.06
K	5.72 ± 0.70	5.23 ± 0.45	6.03 ± 2.11	5.57 ± 0.91	4.98 ± 0.71	4.67 ± 0.74	5.67 ± 0.83
Cl	101.02 ± 1.77	102.08 ± 2.05	102.74 ± 1.68	101.71 ± 2.10	101.96 ± 1.76	103.27 ± 2.57	102.96 ± 1.21
Ca	10.21 ± 0.42	10.25 ± 0.56	10.30 ± 0.39	10.22 ± 0.40	10.13 ± 0.43	10.11 ± 0.26	10.11 ± 0.59
P	6.79 ± 1.43	6.10 ± 0.66	7.51 ± 3.18	6.86 ± 1.06	5.86 ± 0.68	5.78 ± 1.12	7.43 ± 1.43
Mg	2.61 ± 0.29	2.66 ± 0.20	2.73 ± 0.39	2.56 ± 0.16	2.47 ± 0.28	2.51 ± 0.13	2.67 ± 0.31
Iron	318.55 ± 58.30	268.72 ± 41.99	321.96 ± 33.54	297.31 ± 52.21	329.10 ± 36.34	324.71 ± 52.84	291.20 ± 24.13
Zinc	23.60 ± 2.41	20.14 ± 3.74	21.96 ± 3.99	19.34 ± 4.46	19.88 ± 4.34	16.76 ± 1.76	15.47 ± 1.88
Lipase	53.56 ± 31.19	31.86 ± 39.76	22.19 ± 18.99	36.61 ± 41.39	32.69 ± 37.43	7.87 ± 2.10	6.17 ± 1.72
CRP	0.14 ± 0.05	0.14 ± 0.05	0.15 ± 0.08	0.16 ± 0.08	0.16 ± 0.05	0.19 ± 0.04	0.20 ± 0.02

Table S7. Haematological parameters in experimental groups (Ort ± SD).

Parameter	G1 Control	G2 SHAM	G3 (3a)	G4 (3c)	G5 (6b)	MTF	Vehicle
**WBC**	5.34 ± 2.61	5.26 ± 1.66	3.94 ± 2.55	5.10 ± 4.65	4.76 ± 2.96	6.44 ±2.24	3.57 ± 0.82
**PLT**	964.50 ± 174.83	820.43 ± 154.08	894.00 ± 72.83	864.00 ± 140.08	900.50 ± 144.60	780.40 ± 147.57	714.67 ± 99.52
**RBC**	7.70 ± 0.33	7.57 ± 0.50	7.51 ± 0.39	7.66 ± 0.32	7.59 ± 0.33	7.63 ± 0.44	7.55 ± 0.31
**HGB**	14.94 ± 0.79	14.19 ± 0.78	14.39 ± 0.41	14.84 ± 0.42	14.61 ± 0.64	14.67 ± 0.78	14.33 ± 0.29
**HCT**	47.29 ± 4.51	43.00 ± 2.81	43.00 ± 1.27	44.98 ± 2.52	43.86 ± 2.99	42.37 ± 2.27	40.77 ± 0.86
**MCH**	19.41 ± 0.76	18.76 ± 0.53	19.19 ± 0.62	19.39 ± 0.68	19.24 ± 0.59	19.26 ± 0.95	19.00 ± 0.35
**MCV**	59.81 ± 2.31	56.80 ± 2.13	57.34 ± 2.67	58.71 ± 2.83	57.76 ± 2.54	55.57 ± 2.52	54.00 ± 1.55
**MCHC**	32.48 ±1.23	33.04 ± 1.31	33.47 ± 0.89	33.08 ± 1.63	33.39 ± 1.80	34.63 ± 0.24	35.17 ± 0.47

Table S8. Histopathological Scores of Skeletal Muscle (Ort ± SD).

Groups	Control G1	Sarcopenia Model G2	G3 (3a)	G4 (3C)	G5 (6b)	MTF	Vehicle
Score	0.125	3	1.5	1.375	1.5	1.71	3
